# 40th Annual CAPO Conference—Responding to the Human Experience of Cancer and Caring for the Soul: Building on 40 Years of Global Leadership in Psychosocial Oncology

**DOI:** 10.3390/curroncol32040241

**Published:** 2025-04-20

**Authors:** Peter Traversa, Doris Howell

**Affiliations:** 1 Canadian Association of Psychosocial Oncology, Toronto, ON M5A 1S2, Canada; 2 Princess Margaret Cancer Research Centre, Toronto, ON M5G 2M9, Canada

**Keywords:** psychosocial, oncology, cancer, research, accessible, sustainable, innovation

## Abstract

On behalf of the Canadian Association of Psychosocial Oncology, we are pleased to present the Abstracts from the 2025 Annual Conference, titled “Responding to the Human Experience of Cancer and Caring for the Soul: Building on 40 years of global leadership in psychosocial oncology”. The 40th Annual CAPO Conference was held in Toronto from 23 April 2025 to 25 April 2025. In an era marked by the rapid advancement of biologically focused precision medicine, it is imperative to redirect our attention towards the human experience of illness and the soul of medicine. Biomedicine has conceptualized illness in ways that have proved profoundly productive from a curative and biological point of view. But it cannot—and it does not pretend to—illuminate the experience of living with it. (Hurwitz 2009). This conference aims to delve into the intricate interplay between cutting-edge biomedical technologies inclusive of artificial intelligence and big data and the deeply personal narratives of individuals navigating illness. By shifting the focus from mere disease pathology to encompassing the holistic human experience, we aspire to foster a more compassionate and patient-centered approach to healthcare with psychosocial support at the core of humanistic care that can improve survival and well-being in all aspects of a whole-person approach to illness. Through interdisciplinary dialogue and introspection, we endeavor to illuminate the profound connection between mind, body, and spirit in the practice of medicine, reaffirming the timeless significance of empathy, understanding, and human connection in healing and psychosocial aspects of care as fundamental to living well with cancer. This conference brought together key stakeholders including multidisciplinary professionals from nursing, psychology, psychiatry, social work, spiritual care, nutrition, medicine, rehabilitation medicine, occupational health and radiation therapy for both adult and pediatric populations. Participants included clinicians, researchers, educators in cancer care, community-based organizations and patient representatives. Patients, caregivers and family members presented abstracts that speak to their role in managing cancer experiences and care. Over two hundred (200) abstracts were submitted for presentation as symposia, 20-minute oral presentations, 10-minute oral presentations, 90-minute workshops and poster presentations. We congratulate all the presenters on their research work and contribution.

**Abstract Themes:**
A. Adapting PSO care in LMI countries
B. Cancer care across the life span (children, adolescent & young adults, adults, and older adults)
C. Community-based and volunteer cancer care services
D. Complementary and integrative cancer care
E. Exercise/pre-habilitation and rehabilitation in cancer
F. Equity, diversity and inclusion (sociodemographic, culture, and sex/gender issues)
G. Health care provider wellness
H. High tech to high touch (digital health, value-based care, integrated PSO interventions)
I. Implementation science, knowledge translation and synthesis
J. Palliative and end-of-life care
K. Pandemics and cancer care issues
L. Patient oriented research approaches
M. Primary, secondary and tertiary cancer prevention
N. Novel interventions and clinical trials in PSO
O. Survivorship
P. Other
Symposia

## 1. S1-Improving Patient Navigation in Canadian Cancer Care

**Moderator**
Gilla Shapiro
**Summary**

Patient navigation programs seek to improve care by providing support to navigate complex health care systems, increase access to psychosocial and other supportive care services, and overcome health care system barriers. Navigation programs have been introduced across Canada and in global cancer care, providing assistance throughout the cancer continuum, from screening through all stages of disease and treatment. These programs vary substantially by who is targeted (patients, families, or caregivers), what supports are made available, how supports are provided, and the qualifications and training of navigators involved in these programs. Notably, different models (lay, peer and nurse-led models) of navigation have been implemented.

Emerging evidence has shown that navigation can improve outcomes of patients with cancer, facilitate access to services, and reduce health disparities. However, critical questions remain about the impact, cost-effectiveness, and equity of navigation program. This symposium will explore different models of navigation from these perspectives. We will highlight evidence on program effectiveness and research gaps, discuss implementation challenges, and identify opportunities for policy development. Presenters will also discuss contextual issues across different institutions and provinces/territories. Finally, this symposium will address a pressing question: is patient navigation doing enough to address the challenge of health care fragmentation?

**S1-71**

## 2. “Somebody That Can Meet You on Your Level:” Cancer Survivors’ Perspectives on the Role of Indigenous Patient Navigators in Cancer Care

Gary Groot, Maria Diaz

University of Saskatchewan, Saskatoon, Canada.

### 2.1. Background/Rationale or Objectives/Purpose

This study’s purpose was to explore whether First Nations and Métis cancer survivors in Saskatchewan perceive Indigenous patient navigators as a beneficial potential means to enhance their healthcare experiences by facilitating higher quality culturally appropriate care.

### 2.2. Methodology or Methods

Nineteen semi-structured interviews were conducted with First Nations and Métis cancer survivors between May 2022 and March 2023. Thematic analysis was performed to identify and develop themes, categories, and codes that captured participants’ experiences with patient navigators.

### 2.3. Impact on Practice or Results

Participants identified multiple supports that could assist patients in their cancer journey, such as family, community, traditional ways, and First Nations and Métis health support services. Obstacles to accessing care included communication and language barriers, logistical difficulties, cultural differences, financial constraints, and gaps in care. Indigenous patient navigators may play a crucial role in addressing these challenges by offering support in communication, translation, coordination, education, advocacy, and guidance for Indigenous cancer survivors. Indigenous patient navigators’ tasks range from helping schedule appointments to advocating for the patient’s treatment preferences. Additionally, Indigenous patient navigators may play a crucial role in integrating Western medicine with traditional healing practices to support patients pursuing cancer care.

### 2.4. Discussion or Conclusions

From the participants’ perspective, Indigenous patient navigators could offer significant benefits to Indigenous cancer survivors in Saskatchewan.

**S1-113**

## 3. Improving Patient Activation and Quality of Life in Men After Treatment for Prostate Cancer: A Randomized Controlled Trial of a Digital Peer Navigation Program

Jacqueline Bender ^1^, Arminée Kanzanjian ^2^, Robin Urquhart ^3^, Andrew Matthew ^1^, Logan Meyers ^1^, Andrea Vodermaier ^4^, Samantha Radford ^5^, Xiang Ye ^1^, Amy Zhihui Liu ^1^, Rebecca Hancock-Howard ^6^, Jennifer Jones ^1^, Nathan Perlis ^1^, Peter Chung ^1^, Michael McKenzie ^7^, Ryan Flannigan ^2^, Ricardo Renden ^5^, David Bowes ^5^, Antonio Finelli ^1^

1. Princess Margaret Cancer Centre, University Health Network, Toronto, Canada.
2. University of British Columbia, Vancouver, Canada.
3. Dalhousie University, Halifax, Canada.
4. Princess Margaret Cancer Centre, University Health Network, Vancouver, Canada.
5. Nova Scotia Health Authority, Halifax, Canada.
6. University of Toronto, Toronto, Canada.
7. BC Cancer, Vancouver, Canada.

### 3.1. Background/Rationale or Objectives/Purpose

Men diagnosed with prostate cancer lack access to support and face barriers to care when dealing with treatment side effects. Patient navigation may improve access to care and provide personalized support. We evaluated the impact of True North Peer Navigation—a digital peer navigation program for men after treatment for prostate cancer

### 3.2. Methodology or Methods

A pragmatic randomized controlled trial was undertaken. Patients with localized prostate cancer from cancer centres in Ontario, British Columbia, and Nova Scotia were randomized to 3-months of digitally enabled navigation from a trained cancer survivor peer navigator or web-based educational control. The primary outcome, patient activation (PAM), and secondary outcomes including quality of life (EQ5D-5L), were compared at baseline (T0) and at 3-months (T1) using student *t*-tests, chi-square tests and mixed-effects general linear models.

### 3.3. Impact on Practice or Results

172 eligible patients were randomized to intervention (86) and control (86) groups. Average age was 67 years, 65% were born in Canada, and 82% were White North American/European. Significant differences were observed in PAM (diff = 6.71, *p* = 0.004) and EQ5D-5L (diff = 0.04, *p* = 0.02) between intervention and control groups. Improvements in EQ5D-5L composite score may be largely due to a reduction in the anxiety/depression sub-scale score in the intervention compared to the control group (32% vs. 53%, *p* = 0.016).

### 3.4. Discussion or Conclusions

A digital peer navigation program can improve patient activation, quality of life, and distress in men after treatment for prostate cancer. Further work is needed to reach a more diverse, higher need patient population.

**S1-120**

## 4. Strengthening Patient Navigation in Cancer Care Alberta: A Programmatic Evaluation and Recommendations to Grow the Program

Linda Watson ^1,2^, Michelle Lack ^3^

1. Alberta Health Services, Calgary, Canada.
2. University of Calgary, Calgary, Canada.
3. Alberta Health Services, Edmonton, Canada.

### 4.1. Background/Rationale or Objectives/Purpose

Background: Cancer Care Alberta is a provincial ambulatory program with 17 facilities across Alberta. In 2012, Cancer Patient Navigators (CPNs) were implemented in all 15 rural and isolated urban centres to ensure rural Albertans with cancer had timely access to cancer services. In 2016 Indigenous and Adolescent and Young Adult navigator roles were added, with one of each being located at the cancer centres in Edmonton and Calgary. Over the decade, these navigators provided care to thousands of Albertans, however, programmatic coordination, supports and structures were lacking. To identify ways to strengthen the navigation capacity, a programmatic review was conducted to guide efforts to strengthen the navigation program.

### 4.2. Methodology or Methods

Methods: 121 interviews were conducted with Navigators, their managers and other staff who work closely with them. Qualitative data were analyzed using thematic analysis. Member checking of themes and recommendations occurred prior to the final report.

### 4.3. Impact on Practice or Results

Impact on Practice: As a result, navigation is now a provincial program, with a central manager, workflows, centralized referrals and bookings. Additionally, based on trends and unmet needs, two new specialized navigation streams have been added to the types of navigation offered. These include early palliative navigators and disease specific navigators.

### 4.4. Discussion or Conclusions

Discussion: Being a navigator is hard work. These nurses often are dealing with the sickest, most complex patients. Programmatic supports need to be put in place to enable this type of equity informed, person centred care. Next steps include developing caseload and staffing matrix to establish appropriate staffing, and key performance indicators for the program.

## 5. S2-Exploring Unique Approaches to Integrating Oncofertility Care for Adolescents and Young Adults with Cancer Across Canada

**Moderator**
Cheryl Heykoop
**Summary**

In 2022, the Canadian Partnership Against Cancer (CPAC) released a business case for oncofertility screening and care for adolescents and young adults (AYAs, ages 15 to 39) diagnosed with cancer in Canada. Oncofertility focuses on preserving the fertility of cancer patients at risk of infertility due to chemotherapy or radiotherapy. Despite guidelines from the American Society of Clinical Oncology (ASCO) and the Canadian Fertility & Andrology Society (CFAS) recommending that clinicians address the possibility of infertility as early as possible before treatment begins, studies in Canada and elsewhere show that these conversations are often not happening—and especially not before cancer treatment starts.

This gap is concerning, as it denies patients the opportunity to make informed decisions about their future reproductive options, undermining their autonomy and right to participate meaningfully in their care. This issue is particularly significant for AYAs, a vulnerable group whose life stage and social development make the impact of a cancer diagnosis even more profound.

This symposium will feature presentations from five groups across Canada (BC, Alberta, Saskatchewan, Manitoba, and the Atlantic provinces) that received CPAC funding to establish and integrate oncofertility care into clinical practice for AYAs in their jurisdictions. Differences in approaches will be highlighted.

**S2-133**

## 6. Using Participatory Action to Co-Develop a Roadmap for Adolescent and Young Adult Oncofertility Care in BC and the Yukon

Nathan Shen ^1^, Jonathan Avery ^2^, Kristin Marr ^1,3^, Alanah Smrke ^2^, Jennifer Wolfe ^4^, Cheryl Heykoop ^4^

1. University of British Columbia, Vancouver, Canada.
2. BC Cancer, Vancouver, Canada.
3. BC Women’s Hospital, Vancouver, Canada.
4. Royal Roads University, Victoria, Canada.

### 6.1. Background/Rationale or Objectives/Purpose

Adolescents and young adults (AYAs) with cancer face significant challenges in preserving fertility, an often-overlooked aspect of care. The YAC Prime Study (2020) found that only 52% of AYAs discuss fertility preservation with healthcare providers, and just 13% pursue these services. In British Columbia (BC) and the Yukon, oncofertility care is non-standardized, with inconsistent practices and no dedicated AYA program. Funding from the Canadian Partnership Against Cancer enabled a collaboration between BC Cancer, BC Children’s Hospital, and Yukon to develop resources and an implementation strategy for oncofertility.

### 6.2. Methodology or Methods

We used the participatory action research (PAR) approach and employed creative and collaborative methods to conduct multiple AYA working group sessions, individual interviews with AYAs and cancer care providers, and a collaborative session with both groups. Together, these activities helped design an ideal care pathway, develop informational resources, and create an implementation and educational strategy focused on sustainable oncofertility care.

### 6.3. Impact on Practice or Results

We are in the process of measuring the impact in practice. However, learnings to date illustrate the importance of a clear integrated care pathways; clinical guidelines and education; patient-centered educational materials; and a long-term strategy to incorporate tools into the provincial electronic medical record (EMR) system.

### 6.4. Discussion or Conclusions

This research provides a roadmap for transforming fertility preservation services, emphasizing holistic care tailored to the unique challenges AYAs face. By employing PAR, this initiative uniquely centers the voices of AYAs, ensuring that the proposed solutions are not only practical but also deeply informed by lived experiences.

**S2-139**

## 7. Saskatchewan Oncofertility Algorithm (SOFA)—Designing an Inclusive Pathway for Adolescents and Young Adults

Merrick Faulkner ^1^, Selene Daniel-Whyte ^2^, Mita Manna ^2,3^, Paul D’Alessandro ^1,2^

1. Jim Pattison Children’s Hospital, Saskatoon, Canada.
2. University of Saskatchewan, Saskatoon, Canada.
3. Saskatchewan Cancer Agency, Saskatoon, Canada.

### 7.1. Background/Rationale or Objectives/Purpose

Counseling on the potential impact of cancer treatments on future fertility is a standard of care but remains an unmet need for AYAs in Saskatchewan. Our group established a steering committee to develop a provincial oncofertility screening program, using insights from committee discussions and stakeholder engagement to inform a comprehensive AYA oncofertility strategy.

### 7.2. Methodology or Methods

Initial steering committee meetings were held provincially with clinical, managerial, and community stakeholders. Meeting minutes were reviewed to identify barriers and potential solutions to unmet AYA care needs in this domain. Preliminary steering committee recommendations and early implementation strategies will be reviewed.

### 7.3. Impact on Practice or Results

Preliminary steering committee discussions identified barriers to addressing unmet AYA needs, including funding challenges and fragmented care. Unique to our province were unresolved legal and logistic issues from the 2019 centralization and transition of pediatric/adolescent cancer care services to a new children’s hospital (Jim Pattison Children’s Hospital) in Saskatoon. A centralized EMR with IT support, operated by the Saskatchewan Cancer Agency, was used for outpatient pediatric and adolescent/young adult oncology programs, but two different health authorities (Saskatchewan Health Authority and Saskatchewan Cancer Agency) were custodians of personal health information data. Additional funding and data sharing agreements were required to navigate and bridge fragmentations in health systems.

### 7.4. Discussion or Conclusions

Future directions include establishment of an AYA working group as well as embedding screening prompts within existing clinical structures, such as forms filled out by patients during outpatient visits and a screening template for clinicians.

**S2-145**

## 8. Advancing Oncofertility Care for Patients with Cancer in Manitoba: Insights from Patient-Centered Oncofertility Initiative

Mackenzie Jansen ^1^, Madyson Shauman ^1^, Khulood Hasnain ^2^, Cielle Stapleton ^3,4,5^, Patricia Bocangel ^6^, Lorena Gerl ^7^, Sapna Oberoi ^4,5,8^

1. Department of Patient and Family Support Services, CancerCare ManitobaCancerCare Manitoba, Winnipeg, Canada.
2. Rady Faculty of Health Sciences, University of Manitoba, Winnipeg, Canada.
3. Department of Pediatric Hematology/Oncology, CancerCare Manitoba, Winnipeg.
4. Department of Pediatrics and Child Health, Max Rady College of Medicine, University of Manitoba, Winnipeg, Canada.
5. CancerCare Manitoba Research Institute, CancerCare Manitoba, Winnipeg, Canada.
6. Department of Community Oncology Program and Patient Experience, CancerCare Manitoba, Winnipeg, Canada.
7. Department of Patient and Family Support Services, CancerCare ManitobaCancerCare Manitoba, Winnipeg, Canada.
8. Department of Pediatric Hematology/Oncology, CancerCare Manitoba, Winnipeg, Canada.

### 8.1. Background/Rationale or Objectives/Purpose

Adolescent and young adult (AYA) cancer survivors are at risk of developing infertility, yet fertility preservation discussions and referrals remain inconsistent. Retrospective analysis of 263 AYAs diagnosed with cancer in Manitoba in 2022 revealed that only 42% had documented fertility discussions.

### 8.2. Methodology or Methods

In October 2023, CancerCare Manitoba (CCMB), in collaboration with the Canadian Partnership Against Cancer, launched a two-year initiative to enhance equitable access to oncofertility care in Manitoba. The initiative aims to integrate an oncofertility screening prompt, establish sustainable fertility referral pathways, and develop educational resources for healthcare providers (HCPs) and patients. Since initiation, we have integrated an oncofertility screening prompt into adult oncology clinics and childhood cancer survivorship clinic. A pediatric oncology oncofertility questionnaire and AYA fertility counselling have been implemented. To date, we have conducted twelve educational sessions with HCPs, established an oncofertility working task force, collaborated with the fertility clinic, and engaged eight diverse patient partners through ten engagement sessions.

### 8.3. Impact on Practice or Results

Initial steps have facilitated oncofertility screening for most patients and provided insights into current gaps in oncofertility care, needs, challenges associated with implementing screening in outpatient and inpatient settings, and perspectives of patients and HCPs on fertility educational resources.

### 8.4. Discussion or Conclusions

Future steps involve co-developing equitable, accessible, person-centred oncofertility resources for patients and HCPs through their active engagement, evaluating impact of screening and referral processes on fertility discussions/preservation, incorporating oncofertility screening into pediatric oncology settings, outreach to rural CCMB sites, and maintaining ongoing engagement with patient partners and HCPs.

**S2-155**

## 9. Oncofertility in Atlantic Canada

Carol Digout ^1^, Toni Leamon ^2^, Kim Vriends ^3^, Sarah Keeping ^1^

1. Atlantic Provinces Pediatric Hematology/Oncology Network, Halifax, Canada.
2. Atlantic Provinces Pediatric Hematology/Oncology Network, St. John’s, Canada.
3. Atlantic Provinces Pediatric Hematology/Oncology Network, Charlottetown, Canada.

### 9.1. Background/Rationale or Objectives/Purpose

In Atlantic Canada, fertility resources for oncology healthcare providers, patients, and families are limited. Currently, these provinces lack a provider-initiated prompt for fertility screening during cancer treatment. Most charting is not electronic, leaving no easily accessible record of fertility discussions. Funding was provided by the Canadian Partnership Against Cancer.

### 9.2. Methodology or Methods

We focused on three key themes to establish an approach for integrating provider-initiated fertility screening prompts and documenting these discussions:

1. Baseline fertility discussions with patients and families documented at diagnosis.
2. Conversations with pediatric and adult cancer survivors about fertility information they received.
3. Input from pediatric and adult cancer survivors on the fertility information they deemed essential.

We conducted retrospective chart audits across three of the four Atlantic provinces to assess baseline frequency of documented fertility discussions.

To explore the patient/family lived experience, a one-and-a-half-day session was held with 16 self-identifying participants. They provided their expertise and guidance on our proposed themes (as detailed above).

### 9.3. Impact on Practice or Results

Based on data collected and initial analysis, guidelines for oncofertility care have been appropriately updated, education materials for patients/families have been reviewed for adaptation, and new patient-centered prompts are being implemented.

### 9.4. Discussion or Conclusions

For healthcare providers, there is an identifiable lack of knowledge regarding fertility preservation options, funding availability for patients and families, and, most evidently, a lack of documented fertility discussions within a patient’s cancer treatment. It is discernable that documentation embedded prompts and quality indicators (for providers and patients) are a necessary next step.

**S2-153**

## 10. The Build and Integration of an Oncofertility Pathway in Cancer Care Alberta (CCA): Leveraging Connect Care and Change Management to Support Timely and Meaningful Conversations for Cancer Patients

April Wales ^1^, Claire Link ^1^, Andrea DeIure ^2^

1. Cancer Care Alberta, Calgary, Canada.
2. Cancer Care Alberta, Calgary.

### 10.1. Background/Rationale or Objectives/Purpose

Cancer Care Alberta (CCA) has integrated the use of a routine oncofertility screening question to identify and provide timely support for patients who wish to explore fertility preservation prior to starting their cancer treatment. Supported by the Canadian Partnership Against Cancer (CPAC), this work led to the development of a provincial Oncofertility Pathway which will be fully implemented in January of 2025.

### 10.2. Methodology or Methods

The integration of the pathway has required extensive engagement and collaboration across several clinical, operational and information technology teams. The technical build was embedded within Connect Care, CCA’s new provincial electronic health record (EHR), and includes digital alerts to help easily identify patients who would benefit from earlier oncofertility conversations and referrals. In addition, practice supports were developed to facilitate meaningful conversations about oncofertility between clinicians and their patients.

### 10.3. Impact on Practice or Results

Early data and results of the implementation will be shared in this presentation. Current evaluation plans for the oncofertility pathway include the routine monitoring of key metrics within Connect Care and evaluating its impact on clinical practice through a staff/physician survey.

### 10.4. Discussion or Conclusions

Key learnings related to this work will be discussed, including challenges with integration of the pathway into Connect Care and the success of utilizing change management supports with frontline nursing and prescribers. Future goals of this work include continued evaluation to measure effectiveness of the pathway, updating patient-facing resources, and exploring how integrated oncofertility conversations and the implementation of earlier referrals affect patient satisfaction, experience, and outcomes.

## 11. S3-Caring for Those Who Care for Adolescents & Young Adults (AYA): Navigating Grief and Loss in Cancer

**Moderator**
Pamela Mosher
**Summary**

Caring for Adolescents and Young Adults (AYAs) with cancer presents unique challenges and emotions for caregivers and clinicians, especially when AYAs face shortened lifespans or die from cancer. Grief caused by such experiences can be particularly difficult for caregivers and clinicians to navigate, yet hospital-based grief initiatives remain limited. This symposium highlights three such initiatives: two supporting AYA caregiver grief and one supporting AYA clinician grief.

The first details a remembrance service, created by AYA Program staff at a large provincial cancer centre, to offer caregivers/loved ones a communal space to honour AYAs who have died. The second explores meaning-making challenges for bereaved AYA caregivers, the primary features of Meaning-Centred Grief Therapy, and its application in clinical practice. The third addresses the use of Lavender Alerts—an intervention that provides clinicians opportunities to process stressful or emotional clinical experiences—in the AYA oncology setting.

The results will be reviewed in greater detail in individual presentations, but each initiative has evidenced a positive impact on participants and facilitators.

These presentations underscore the importance of addressing grief for both caregivers and clinicians in the context of AYA cancer care.

**S3-138**

## 12. Caring for the Soul: The Human Experience in Grief—A Remembrance Service for Adolescents and Young Adults (AYAs) with Cancer

Jennifer Catsburg, Sofia Canales Albarran

Princess Margaret Cancer Centre, Toronto, Canada.

### 12.1. Background/Rationale or Objectives/Purpose

Losing a young loved one poses tremendous challenges in bereavement for their caregivers, families, and community. In the Adolescent and Young Adult (AYA) Program at the Princess Margaret Cancer Centre, caregivers of AYAs were increasingly seeking comfort and community in their grief. To address this need, we held our inaugural Remembrance service, “AYA Remembers” in September of 2024, to provide a dedicated space for bereaved loved ones to come together in shared grief and remembrance.

### 12.2. Methodology or Methods

The experience of creating and hosting a service of remembrance for AYAs will be presented. The many considerations discovered in cultivating a grieving space specifically tailored for the grief of losing a young person to cancer will be shared. The how, who, and why of an AYA remembrance service will be articulated along with comments on the impact for its attendees and facilitators.

### 12.3. Impact on Practice or Results

The experience of hosting a remembrance service and feedback from attendees ranged from being described as “imperative” to “beautiful”. This event highlighted the need for support that extends beyond the cancer experience, emphasizing the importance of providing support for the caregivers of AYAs even after the death of their loved one. This has further demonstrated that beyond cancer care, in death and in grief, AYAs and their families are in desperate need of tailored services.

### 12.4. Discussion or Conclusions

This remembrance service for AYAs has highlighted the importance of prioritizing grief and bereavement. Feedback from attendees will inform the planning and execution of an annual remembrance service in our AYA programming.

**S3-122**

## 13. Supporting the Supporters: Lavender Alert as a Necessary Tool in Caring for AYA Oncology Patients

Sofia Canales Albarran, Jennifer Catsburg, Peggy Moore, Suraj George, Pamela Mosher

Princess Margaret Cancer Center, UHN, Toronto, Canada.

### 13.1. Background/Rationale or Objectives/Purpose

Caring for adolescents and young adults (AYA) with cancer is uniquely challenging for healthcare providers due to the developmental needs of AYA and the emotional toll of life-limiting illnesses. To support our AYA oncology team, we partnered with PM Spiritual Care to facilitate “Lavender Alert”, a wellness initiative offering emotional support during select monthly meetings.

### 13.2. Methodology or Methods

The intervention was piloted twice over six months, allowing in-person or virtual participation. Each Lavender Alert session followed the ORID methodology, incorporating a mindfulness exercise, a facilitated group debrief on managing work-related emotional challenges, time for reflection, and concluding music (once provided live by a music therapist). Feedback was gathered through informal conversations and a formal mixed method written questionnaire.

### 13.3. Impact on Practice or Results

Initial feedback has been positive, with 87.5% of participants finding the sessions “very helpful” to “extremely helpful”. A team member described the initiative as fostering “a sense of community, feeling held as a group in our challenges and grief”. The sessions helped normalize clinicians’ need for emotional care and strengthened team cohesion.

### 13.4. Discussion or Conclusions

Lavender Alerts provide a structured space to acknowledge and process grief and the emotional impact of AYA oncology care. This initiative highlights the importance of providing structured support for healthcare providers in emotionally demanding environments. Future plans include establishing quarterly sessions, formalizing feedback collection, and expanding the program across other teams in our cancer centre. Lavender Alert highlights how small, intentional interventions can sustain clinician well-being and promote compassionate care delivery to AYAs with cancer.

**S3-154**

## 14. Applications of Meaning-Centered Grief Therapy for Bereaved Caregivers of Adolescent & Young Adult Patients with Cancer

Aliza A. Panjwani ^1,2^, Wendy G. Lichtenthal ^3,4^

1. Princess Margaret Cancer Centre, Toronto, Canada.
2. University of Toronto, Toronto, Canada.
3. Center for the Advancement of Bereavement Care, Sylvester Comprehensive Cancer Centre, Miami, USA.
4. University of Miami Miller School of Medicine, Miami, USA.

### 14.1. Background/Rationale or Objectives/Purpose

For bereaved caregivers of adolescents or young adults with cancer (AYACs), grief may be experienced as particularly intense because the death of the AYAC occurs during a life stage typically associated with health, growth, and possibility. Yet tailored bereavement support for caregivers of AYACs is scarce. Initially developed for parents grieving the loss of a child to cancer, Meaning-Centered Grief Therapy (MCGT) is an evidence-based, 16-session intervention that utilizes a cognitive-behavioral-existential approach to address grief and meaning-related challenges. This paper aims to: (1) explore common meaning-making challenges experienced by bereaved caregivers of AYACs, including parents and partners; (2) outline MCGT’s core components; and (3) describe its application in clinical practice.

### 14.2. Methodology or Methods

We will present the unique challenges faced by bereaved caregivers of AYACs and synthesize the theoretical framework and core components of MCGT, including facilitating meaning reconstruction, connection to sources of meaning, and legacy work. Case illustrations will demonstrate the application of MCGT to the grief experiences of bereaved parents and partners of AYACs.

### 14.3. Impact on Practice or Results

MCGT principles hold relevance for different types of bereaved caregivers of AYACs and can inform clinical practice by offering evidence-based experiential strategies to help caregivers process grief, utilize sources of meaning to transcend suffering, and facilitate adjustment after loss.

### 14.4. Discussion or Conclusions

The use of MCGT in practice suggests the importance of identifying complementary resources to address concurrent challenges, such as sleep disturbance or social isolation. For providers engaged in bereavement work with this population, managing countertransference and accessing safe spaces for emotional support are essential for sustaining compassionate care.

## 15. S4-Comprehensive Approach to Managing Cancer-Related Stress in Individuals and Families to Help People Live, While Living with Cancer

**Moderator**
Elizabeth Dalgleish
Summary

### 15.1. Background

Gilda’s Toronto provides personalized psychosocial cancer support that blends a compassionate approach to evidence-based programming. The innovative online/virtual service model is a complement to medical treatments, offering comprehensive support for the whole person and the whole family in a community of shared experience, led by mental health professionals.

### 15.2. Methods 

The first presentation describes a validated cancer support screening tool that identifies key psychosocial concerns for patients and caregivers, enabling personalized support recommendations. The second presentation focuses on professionally facilitated support groups that foster community through shared experiences. The final presentation will explore the innovative approach to programming that fosters emotional resilience and healing for the whole family.

### 15.3. Impact on Practice 

Professionally facilitated support groups are essential for fostering connections and community. These therapeutic peer support programs empower patients and their families, reducing isolation and stress while enhancing their sense of control during the cancer experience.

### 15.4. Discussion 

Conducting thorough screenings to understand unique needs allows individuals to choose their interventions, contributing to improved mental health outcomes. Gilda’s Toronto will continue to enhance screening rates; assess barriers and needs among vulnerable communities; and, improve access to psychosocial cancer support services to improve psychosocial well-being for cancer patients and their families.

**S4-136**

## 16. Beyond the Diagnosis, Supporting the Whole Family: Holistic Psychosocial Programming and Empowering Families Through a Cancer Diagnosis

Tory Hagerman

Gilda’s Toronto, Toronto, Canada.

### 16.1. Background/Rationale or Objectives/Purpose

Cancer profoundly impacts not only the individual diagnosed but also the entire family unit. Gilda’s Toronto has expanded its psychosocial support services through compassionate, evidence-based programming to address the needs of families affected by cancer. By fostering a community of shared experiences, Gilda’s Toronto has become a cornerstone of support for families, promoting emotional resilience and healing.

### 16.2. Methodology or Methods

Gilda’s Toronto offers tailored programs to meet the unique needs of each family member, ensuring that support is available through shared experiences and a sense of belonging. Children, teen, and parent support groups play a vital role in fostering connections and mutual understanding. Specialized programs, such as Careful Steps, provide one-on-one support for parents concerned about the emotional impact of cancer on their children. Additional opportunities for relationship-building include regular family evenings and an enriching overnight family camp experience.

### 16.3. Impact on Practice or Results

Through a holistic approach, Gilda’s Toronto addresses cancer-related stress and isolation within families. Both virtual and in-person family programming has resulted in reported improvements in coping skills, reduced isolation, and a sense of empowerment in navigating grief and the cancer journey together.

### 16.4. Discussion or Conclusions

Gilda’s Toronto’s family programming highlights the importance of community-based support and opportunities for connection, for families impacted by cancer. Future efforts aim to expand psychosocial support for families of adult and pediatric patient families, addressing gaps in care by partnering and collaborating with healthcare teams and community organizations. Additionally, Gilda’s Toronto seeks to explore and address social determinants of health, barriers, and the needs of diverse communities by expanding family programs and connections within these groups.

**S4-144**

## 17. Comprehensive Cancer Support Through Distress Screening: How CancerSupportSource^®^ Helps Gilda’s Toronto Create a Customized Cancer Support

Nancy Hoang

Gilda’s Toronto, Toronto, Canada.

### 17.1. Background/Rationale or Objectives/Purpose

Cancer presents significant emotional, psychological, and practical challenges for those living with the diagnosis and their caregivers. Current research underscores the importance of identifying individual distress and unmet needs, as the failure to do so can hinder treatment adherence and overall well-being.

### 17.2. Methodology or Methods

The CancerSupportSource^®^ (CSS), an evidence-based distress screening tool proprietary to Gilda’s Toronto, is instrumental in the development of personalized psychosocial support plans for those living with cancer and their caregivers. This validated tool surveys the concerns commonly experienced by those affected by cancer, including but not limited to emotional well-being (risk of anxiety and depression), symptom burden, healthcare team communication and intimacy. The results of the survey are provided in a personalized report that identifies programs, services and resource recommendations that are customized for individuals and families with the facilitation of a mental health professional during support planning meetings.

### 17.3. Impact on Practice or Results

Through the CSS, Gilda’s Toronto supports individuals and families affected by cancer to access evidence-based internal programs and services, such as support groups, healthy lifestyle classes, educational webinars, and referrals to additional external community resources.

### 17.4. Discussion or Conclusions

Gilda’s Toronto has leveraged the use of CSS to streamline access to psychosocial care, reduce barriers, and provide comprehensive, no-cost support for individuals and families navigating the complexities of cancer. The use of this evidence-based tool will offer future opportunities to evaluate diverse needs within vulnerable communities, and program meaningful customized psychosocial programs that speak to identified needs.

**S4-197**

## 18. Connecting Through Care: The Impact of Virtual Professionally Led Peer Support Groups at Gilda’s Toronto

Katie Jacobs

Gilda’s Toronto, Toronto, Canada.

### 18.1. Background/Rationale or Objectives/Purpose

Gilda’s Toronto has been at the forefront of providing community-based support for individuals affected by cancer for over 20 years. The organization offers innovative, personalized, and evidence-based cancer support, blending compassionate care with mental health expertise. The virtual peer support groups, led by trained mental health professionals, play a pivotal role in addressing cancer-related stress and enhancing emotional well-being.

### 18.2. Methodology or Methods

A needs assessment revealed that cancer patients and their families often experience heightened stress, isolation, and emotional distress. To address these challenges, Gilda’s Toronto developed virtual, professionally facilitated peer support groups, designed using evidence-based practices and a compassionate, individualized approach. Outcomes were measured using CancerSupportSource (CSS) and post-group surveys to assess psychosocial impact, focusing on participants’ sense of community, emotional validation, and stress reduction.

### 18.3. Impact on Practice or Results

These virtual peer support groups have significantly impacted the practice of cancer care by expanding reach to individuals who may have otherwise lacked support. The results demonstrate a reduction in feelings of isolation and an overall improvement in emotional well-being for participants.

### 18.4. Discussion or Conclusions

Key lessons learned include the importance of group structure, facilitator involvement, and the integration of appropriate digital tools to optimize the virtual support experience. Future directions include reaching more diverse constituencies, offering more in-person program options, and understanding the psychosocial benefits of programming through enhanced reassessment process and evaluating cancer-related stress.

**S4-143**

## 19. S5—From Trauma to Meaning: Psychotherapy for Patients and Caregivers in the Context of Life-Threatening Cancer

**Moderator**
Gary Rodin
**Summary**

The acute onset and ongoing threat of life-threatening cancer may generate enormous distress in patients and caregivers who are affected. Traumatic stress symptoms (TSS) that meet criteria for acute stress disorder and post-traumatic stress disorder are common in this circumstance. Distress of this kind adversely affects physical and emotional wellbeing and is associated with an elevated suicide risk. Emotion and Symptom Focused Engagement (EASE) is a psychotherapeutic approach developed to prevent or alleviate TSS in the context of life-threatening disease and to facilitate a transition to reflection and meaning making. In this symposium, the nature of EASE will be described and data from two separate EASE trials will be reviewed to indicate the prevalence of TSS in patients and caregivers facing life-threatening cancer, and the feasibility and effectiveness of EASE in alleviating these symptoms. Presentations will include qualitative and quantitative data from a multi-site randomized control trial of EASE in adults with acute leukemia (AL) and from a feasibility trial of EASE in the parents of children with AL. The panel will reflect on their experiences delivering EASE and its role as a stand-alone intervention or as a phase of a reflective therapy, such as Managing Cancer and Living Meaningfully.

**S5-163**

## 20. The Prevalence of Traumatic Stress in Patients with Newly Diagnosed Acute Leukemia

Elizaveta Klekovkina, Maya Stern, Angela Mathews, Jay Hung, Anne Rydall, Breffni Hannon, Camilla Zimmermann, Gary Rodin

Princess Margaret Cancer Centre, Toronto, Canada.

### 20.1. Background/Rationale or Objectives/Purpose

Traumatic stress symptoms (TSS) frequently emerge in response to the diagnosis of acute leukemia (AL). This study explored the prevalence, severity, and factors associated with clinically relevant TSS in this population.

### 20.2. Methodology or Methods

Baseline data were collected from adult patients with newly diagnosed AL enrolled in an ongoing phase 3 randomized controlled trial (RCT) of the Emotion and Symptom-focused Engagement (EASE) intervention. Patients were recruited from four Ontario-based cancer centres.

Eligible patients were diagnosed with AL and receiving induction therapy with curative intent. Participants completed questionnaires capturing demographics; the severity and nature of TSS, using the Stanford Acute Stress Reaction Questionnaire-II (SASRQ-II); physical symptom burden, using the Memorial Symptom Assessment Scale. Descriptive and multivariate analyses were conducted to determine the prevalence and severity of TSS and explore potential risk factors for TSS in this cohort.

### 20.3. Impact on Practice or Results

Data were collected from 85 participants (mean age 51.3 years, 51.8% female) between January 2020 and February 2024. The mean SASRQ-II score was 39.8, with 48.2% scoring ≥ 40, indicating clinically significant TSS. Participants reported high physical symptom burden, with the most prevalent symptoms being lack of energy (86%) and drowsiness (67%).

### 20.4. Discussion or Conclusions

Clinically significant TSS are present in almost half of adults with newly diagnosed AL, highlighting the need for early psychosocial support. A brief and proactive intervention such as EASE that specifically targets TSS, may help to alleviate this distress, enhance coping, and reduce the risk of subsequent PTSD in individuals with the acute onset of a life-threatening condition.

**S5-164**

## 21. A Multi-Method Evaluation of the Feasibility and Acceptability of Emotion and Symptom-Focused Engagement (EASE) in the Parents of Children with Acute Leukemia

Maya Stern ^1^, Ally Yu ^2^, Argin Malakian Malakian ^2,3^, Laura Foran ^2^, Kate Hunt ^2^, Stephanie Nanos ^3,4^, Anne Rydall ^2^, Sarah Alexander ^4,3^, Lindsay Jibb ^4,3^, Gary Rodin ^1,3^

1. Princess Margaret Cancer Centre, Toronto, Canada.
2. Princess Margaret Cancer Centre, Toronto, Canada.
3. University of Toronto, Toronto, Canada.
4. Hospital for Sick Children, Toronto, Canada.

### 21.1. Background/Rationale or Objectives/Purpose

Acute leukemia (AL) in a child is associated with significant psychological distress in the parent. Emotion and Symptom-Focused Engagement (EASE) is a psychotherapeutic intervention designed to address trauma-related psychological distress in those affected by AL, incorporating four domains: relational support, affect regulation, problem-solving, and mentalization. This multi-method single-arm pilot trial evaluated the feasibility and acceptability of EASE prior to a Phase III randomized controlled trial (RCT).

### 21.2. Methodology or Methods

Parents of children (<18-years-old) with AL were recruited from the Hospital for Sick Children and engaged in EASE over approximately twelve weeks. We collected quantitative measures of feasibility (e.g., recruitment, retention, session completion rates) and used semi-structured interviews to explore perceptions of intervention acceptability. Quantitative data were analyzed descriptively, and qualitative interviews were analyzed using reflexive thematic analysis.

### 21.3. Impact on Practice or Results

Thirty-five of fifty-nine eligible parents (59%) consented. Thirty-two of thirty-five participants (28 mothers, 7 fathers) completed the minimum of three therapy sessions (median: 6; range: 3–19), with a 91% retention rate. Three parents withdrew. EASE was highly acceptable to parents, with perceived benefits related to emotional regulation, empowerment, and relational support. The flexibility, proactive scheduling, and virtual format of EASE facilitated retention. Participants expressed interest in additional psychoeducation on managing traumatic stress symptoms.

### 21.4. Discussion or Conclusions

This trial demonstrates that EASE is feasible and acceptable to parents of children with AL and suggests it may be suitable for parents of children with other cancers. Consequently, these findings informed the development of a CIHR-funded RCT to evaluate the efficacy of EASE in caregivers of children with any cancer.

**S5-165**

## 22. Emotion and Symptom-Focused Engagement (EASE): An Integrated Psychotherapeutic and Palliative Care Intervention in the Acute Phases of Life-Threatening Cancer

Kate Hunt ^1^, Laura Foran ^1^, Carmine Malfitano ^1^, Anne Rydall ^1^, Stephanie Nanos ^1,2,3^, Argin Malakian ^1,3^, Breffni Hannon ^1,3^, Camilla Zimmermann ^1,3^, Gary Rodin ^1,3^

1. Princess Margaret Cancer Centre, Toronto, Canada.
2. Hospital for Sick Children, Toronto, Canada.
3. University of Toronto, Toronto, Canada.

### 22.1. Background/Rationale or Objectives/Purpose

The prevalence of traumatic stress disorders in life-threatening cancer is well-documented. An integrated psychosocial and early palliative care intervention known as Emotion and Symptom-focused Engagement (EASE) has been developed to alleviate traumatic stress symptoms and comorbid physical symptoms. A phase II randomized control trial (RCT) demonstrated that EASE is feasible and acceptable for patients with newly diagnosed acute leukemia (AL), and offered preliminary evidence of efficacy in alleviating both traumatic stress symptoms and physical symptoms. A CIHR-funded multi-site phase III RCT with an embedded qualitative study is currently underway, comparing EASE + usual care (UC) to UC alone. 

### 22.2. Methodology or Methods

EASE is a novel, manualized intervention designed to alleviate traumatic stress and physical symptom burden in the acute stages of life-threatening disease. This trauma-focused intervention has two components: (1) EASE-psy, the psychological component, addresses four critical domains—relational support, affect regulation, problem-solving, and mentalization; and (2) EASE-phys, the physical component, includes weekly symptom screening triggering early palliative care.

### 22.3. Impact on Practice or Results

Qualitative data showed that participants in both groups expressed reluctance to rely on family caregivers for emotional support. Participants receiving UC viewed emotional suppression as an adaptive coping strategy. Conversely, participants receiving EASE + UC emphasized the importance of processing emotions with someone external and non-judgmental, highlighting the provider’s emotional presence as a key benefit of the intervention.

### 22.4. Discussion or Conclusions

Our findings illustrate the value of EASE in alleviating traumatic stress during the acute stages of a life-threatening cancer. This highlights the need for more routine access of patients in this circumstance to interventions such as EASE.

**S5-161**

## 23. Emotion and Symptom-Focused Engagement for Caregivers (EASE-CG): A Phase III RCT of an Intervention Targeting Traumatic Stress in Parents of Children with Cancer

Lindsay Jibb ^1^, Stephanie Nanos ^1,2^, Sarah Alexander ^3^, Anne Rydall ^2^, David Brownstone ^3^, Laura Foran ^2^, Kate Hunt ^2^, Argin Malakian ^2^, Elizaveta Klekovkina ^2^, Gary Rodin ^2^

1. Child Health Evaluative Sciences, The Hospital for Sick Children, Toronto, Canada.
2. Department of Supportive Care, Princess Margaret Cancer Centre, University Health Network, Toronto, Canada.
3. Division of Haematology/Oncology, The Hospital for Sick Children, Toronto, Canada

### 23.1. Background/Rationale or Objectives/Purpose

Childhood cancer is a singularly stressful life event for children and their families. We found that >50% of parents of children with cancer report clinically significant traumatic stress symptoms (TSS), which negatively impact physical/mental health, quality of life, and parents’ capacity to care for their ill child. However, psychological care for these caregivers is largely unstandardized. We developed a manualized psychotherapeutic intervention, called Emotion and Symptom-focused Engagement (EASE), for adult patients with acute leukemia (AL) and family caregivers. EASE integrates relational support, affect regulation, mentalization, and problem-solving. EASE pilot trials in both adult patients and parents of children with AL demonstrated its feasibility and acceptability, and we are now conducting an RCT to determine its effectiveness in mitigating TSS in parents of children with any cancer.

### 23.2. Methodology or Methods

This will be a 2-arm, parallel-group RCT with 1:1 allocation, enrolling 153 parents per arm at the Hospital for Sick Children (Toronto) with a nested qualitative study. Participants will be the primary parental caregiver of a child within 6-months of a new or relapse cancer diagnosis. *Treatment Arm.* Parents will receive usual care and EASE, with sessions delivered over 12-weeks by a trained clinician. *Control Arm*. Parents will receive usual care alone.

### 23.3. Impact on Practice or Results

Both arms will complete outcome measures at baseline, 4-, 8-, and 12-weeks, and 6-months, analyzed using area under the curve/regression models. Interviews will be conducted at 12-weeks and analyzed using content analysis.

### 23.4. Discussion or Conclusions

If shown effective, EASE has the potential to become a standard of psychosocial care to improve/maintain the well-being of parents of children with cancer.

## 24. Final CategoryA. Adapting PSO Care in LMI Countries

**88**

## 25. Psycho-Social Problems on Cancer in Rural India: A Comprehensive Study

Aditya Manna

MAS Clinic & Hospital, Tamluk, India.

### 25.1. Background/Rationale or Objectives/Purpose

This research aimed to investigate and analyze the psycho-social problems faced by cancer patients in rural India. The study focused on understanding the impact of cancer on individuals’ psychological well-being, social support systems, and overall quality of life.

### 25.2. Methodology or Methods

A mixed-methods approach was employed to gather comprehensive data on the psycho-social challenges faced by cancer patients in rural India. Quantitative surveys were administered to collect demographic information, assess psychological distress levels, and evaluate social support networks. In-depth interviews and focus group discussions were conducted to gain qualitative insights into the experiences and perceptions of cancer patients, their families, and healthcare providers.

### 25.3. Impact on Practice or Results

The findings revealed that cancer patients in rural India encountered numerous psycho-social challenges. Psychological distress was prevalent, with high levels of anxiety, depression, and emotional instability reported. Limited access to healthcare services, inadequate social support structures, and stigma surrounding cancer further exacerbated the psycho-social burden. Additionally, financial constraints, lack of awareness about available resources, and cultural beliefs influenced the coping mechanisms employed by patients and their families.

### 25.4. Discussion or Conclusions

The study underscores the urgent need for targeted interventions and support systems to address the psycho-social problems faced by cancer patients in rural India. Efforts should be made to enhance mental health services, raise awareness, and reduce the stigma associated with cancer. Moreover, fostering stronger social support networks and improving access to healthcare facilities can significantly alleviate the psycho-social burden and enhance the overall well-being of cancer patients in rural areas.

**200**

## 26. What Do Iranian Psychosocial Oncology Providers Need to Enhance Their Practice?

Baharan Ghavami ^1,2,3^, Fariba Zarani ^1^, Ladan Fata ^4^, Mohammadreza Sharbafchi ^5^, Jacqueline Bender ^2,3^

1. Shahid Beheshti University, Tehran, Iran, The Islamic Republic of Iran
2. University of Toronto, Toronto, Canada.
3. Princess Margaret Cancer Centre, Toronto, Canada.
4. Iran University of Medical Sciences, Tehran, Iran, The Islamic Republic of Iran.
5. Isfahan University of Medical Sciences, Isfahan, Iran, The Islamic Republic of Iran.

### 26.1. Background/Rationale or Objectives/Purpose

Psychosocial oncology providers (psycho-oncologists) play a crucial role in delivering supportive care to cancer patients and their caregivers. Iranian families value shared caregiving and view cancer as a family issue; therefore, psycho-oncologists provide support to families affected by cancer. Considering Iran as a low- and middle-income country with limited supportive care resources, little is known about the experiences and challenges of psychologists working in oncology settings. To address this gap, we explored the professional challenges faced by Iranian psycho-oncologists in working with cancer affected families and gathered their suggestions to improve their practice.

### 26.2. Methodology or Methods

Employing a qualitative descriptive study design, we conducted individual interviews and two focus groups with 15 psycho-oncologists in Iran. All interviews and focus groups were recorded and transcribed verbatim. Data analysis was performed using qualitative thematic analysis.

### 26.3. Impact on Practice or Results

The main professional challenge faced by psycho-oncologists is uncertainty in working with cancer patients and their caregivers. This stems from insufficient ongoing specialized supervision in psycho-oncology, lack of scientific professional network for knowledge sharing, and not being embedded into the circle of care. They require official recognition and integration within oncology settings, and the establishment of formal accreditation procedures by health policymakers.

### 26.4. Discussion or Conclusions

Uncertainty is a challenge for Iranian psycho-oncologists. In some countries, actions have been taken to address this issue by developing practicum and core-competency psychosocial oncology training. Interaction with international professional events can reduce isolation and facilitate knowledge exchange through training programs. Incorporating formal and culturally sensitive supervision and providing venues for accreditation can enhance the career development of psycho-oncologists and protect them against burnout in the emotionally intense field of cancer care.

## 27. Final Category: B. Cancer Care Across the Life Span (Children, Adolescent & Young Adults, Adults, and Older Adults)

**5**

## 28. A Refinement Study of Taking Back Control Together, an Intervention to Support Parents Confronted with Pediatric Oncology

Nikita Guarascio ^1,2^, Ariane Levesque ^1,2^, David Ogez ^1^, Valérie Marcil ^1,2^, Daniel Curnier ^1,2^, Émélie Rondeau ^2^, Serge Sultan ^1,2^

1. University of Montreal, Montreal, Canada.
2. Sainte-Justine University Health Center, Montreal, Canada.

### 28.1. Background/Rationale or Objectives/Purpose

A child’s cancer diagnosis profoundly impacts the psychological well-being of parents. To alleviate parental distress, researchers developed *Taking Back Control Together* (TBCT), a manualized six-session program to support parents by targeting individual problem-solving skills and dyadic coping. The current study aimed to refine TBCT for future implementation across Quebec (Phase 1b ORBIT model).

### 28.2. Methodology or Methods

We invited potential interventionists and local stakeholders from three pediatric oncology centers (CHU Sainte-Justine, CHU Sherbrooke, and CHU Quebec-University Laval) to join the refinement team. The final working team comprised 28 professionals, including social workers, psychologists, researchers, coordinators, and parent-partners.

The refinement group operated through dynamic discussions and iterative decision-making facilitated by regular meetings. This approach allowed the team to adapt and respond to emerging challenges and opportunities. The study included eight 50- to 90-min discussion sessions designed to stimulate conversation and facilitate the exchange of ideas and perspectives.

### 28.3. Impact on Practice or Results

Using the framework analysis method, we organized data into three categories: (1) Content modifications, including language simplification and visual enhancements; (2) Intervention description, addressing changes in personnel, resource disparities, and tailoring to accommodate different family structures; and (3) Factors influencing implementation, such as accessibility, participant satisfaction, clinician compensation, and flexibility in program delivery.

### 28.4. Discussion or Conclusions

The direct output of this research is a refined program with an updated manual, tools, and format adapted for the Province of Quebec. By involving local stakeholders in this early phase and addressing implementation barriers before expanding the program across centers, we expect TBCT to be ready for feasibility and pilot trials in Quebec.

**8**

## 29. Association of In-Hospital Oncology Camp Programming on the Psychosocial Health of Youth Who Have Lived with Cancer—A Pilot Study

Sarah O’Connell ^1^, Divine Udobor ^1^, Shannon Miller ^1^, Lauren Chisholm ^2^, Sarah L West ^1^

1. Trent University, Peterborough, Canada.
2. Campfire Circle, Muskoka, Canada.

### 29.1. Background/Rationale or Objectives/Purpose

Youth with cancer encounter significant adversity, therefore experiences promoting psychosocial health are necessary. This study investigated the association of in-hospital recreational oncology camp (ROC) participation with psychosocial health, including the resilience, hope, social support, and mental wellbeing, of youth with a history of cancer.

### 29.2. Methodology or Methods

Participants were recruited from the ROC program registration list (Campfire Circle). Youth were categorized into one of 4 groups: (1) currently in-hospital/on treatment and accessing OC programs; (2) currently in-hospital/on treatment and previously but not currently accessing OC programs; (3) not currently in-hospital/on treatment and previously accessed OC programs; (4) not currently in-hospital/on treatment and never accessed OC programs. Participants completed an online survey including the Children’s Hope Scale (CHS), Child and Youth Resilience Measure (CYRM-R), Social Provisions Scale (SPS-5), Warwick-Edinburgh Mental Wellbeing Scale (SWEMWBS), and open-ended questions regarding their experiences with ROC. T-tests and one-way ANOVAs evaluated differences in survey scores between groups. Thematic analysis was performed.

#### Impact on Practice or Results

Quantitative analyses included 28 participants (12.1 ± 6.2 years), with 21 participants (10.6 ± 1.5 years) included in qualitative analysis. Scores on CHS, CYRM-R, SPS-5, and SWEMWBS did not differ across groups. Three main themes emerged from qualitative data: positive emotions, positive experiences, and support & connection related to ROC participation.

### 29.3. Discussion or Conclusions

In-hospital ROC programming was not associated with higher hope, resilience, social support, or mental wellbeing in this pilot cross-sectional analysis; however, ROC provided positive experiences that elicited feelings of happiness, excitement, and perceived support in youth who have lived with cancer.

**17**

## 30. Diagnostic Delay Disparities of Breast Cancer in Young Women…An Emerging Issue of AYA Cancer Care Importance

Lorna Larsen

Team Shan Breast Cancer Awareness for Young Women (Team Shan), Huntsville, Canada.

### 30.1. Background/Rationale or Objectives/Purpose

Young women with breast cancer face unique challenges over the course of their cancer journey. Globally the higher incidence of cancer among young women is disproportionately driven by the incidence of breast cancer and the highest proportion of AYA cancer deaths and disability-adjusted life years (Dalys) are attributed to breast cancer. Delayed diagnosis has been identified as a pre-diagnosis issue for young women with breast cancer. Young women are also more likely to be diagnosed with distant involvement than older women and incur higher recurrence rates.

### 30.2. Methodology or Methods

Literature reviews have identified that a delay in diagnosis impacts treatment limitations and ultimately chances of survival. Due to patient or physician inaction, diagnostic delays can lead to increased distress for young women in an already stressful situation including impaired health related quality of life, anxiety and depression.

Primary Care Provider (PCP) breast cancer in young women knowledge gaps have been documented as a significant barrier to a timely breast cancer diagnosis. Clarifying misconceptions ‘too young for breast cancer’ and increasing PCP knowledge levels of breast cancer symptoms in young women can help expedite an accurate diagnosis and treatment pathway.

### 30.3. Impact on Practice or Results

Knowledgeable PCPs who ‘Consider Cancer’ have the potential to accurately diagnose, decrease distress, improve patient outcomes, and address morbidity and mortality age disparities. Psychosocial oncology professional AYA cancer advocacy can assist in encouraging health care provider awareness to help fill knowledge gaps in breast cancer in young women. Knowledgeable psychosocial oncology professionals are also in a position to support young women diagnosed late.

### 30.4. Discussion or Conclusions

This presentation will review the issue of late diagnosis in young women as an issue of public health and psychosocial oncology importance. Some documented health promotion strategies to address the knowledge gaps for the PCPs that service them will be shared. The impact on practice and the way forward to address the disparities for young women with breast cancer will be explored.

**21**

## 31. Lived Experience of a Mother to a Child with Pediatric Cancer by Nabaasa Jean from Uganda

Nabaasa Jean

Cancer care, Kampala, Uganda.

### 31.1. Background/Rationale or Objectives/Purpose

Lived Experience of a Mother to a Child with Pediatric Cancer by Nabaasa Jean from Uganda.

### 31.2. Methodology or Methods

My motivation to participate in this conference (Responding to the human experience of the cancer and caring for the soul) is informed by own lived experiences as a mother who was taking care of my own daughter battling Bilateral Wilms tumor for 4 years and 8 Months. My daughter was diagnosed in February 2020 at 1 year and 2 months.

### 31.3. Impact on Practice or Results

As a mother, I did all it takes to look for support besides acquiring a loan of worth USD 40,000 from my work to take this child to India. She underwent left nephroureterectomy and right partial nephrectomy in December 2020. Post-surgery chemotherapy was given for 6 months. In the year 2022, recurrence was detected, received treatment with ICE protocol chemotherapy till March 2023. She underwent right solidarity kidney-Wilms tumour excision in May 2023 and was put adjuvant oral metronomic chemotherapy. Unfortunately, she lost the battle on 17 September 2024. During this journey life has not been the same as a mother with other family responsibilities including taking care of the financial issues. These have been coupled with physical burden, psychological torture, disruptions of family routines and social life, inadequate support and isolation and disease related stigma.

### 31.4. Discussion or Conclusions

Given the experience I went through, I would like to request the partners in this conference to support my vision of setting up a community based organization in the memory of my daughter with the aim of cancer advocacy to increase awareness and support the young and vulnerable children to adhere to treatment.

**42**

## 32. A Preliminary Exploration of Health-Related Quality of Life in Children Treated with Reirradiation for Diffuse Intrinsic Pontine Glioma or Recurrent Brain Tumours and Their Families

Jenny Duong ^1^, Caitlin Forbes ^1^, Derek Tsang ^2,3,4^, Anirban Das ^4,5^, Fiona Schulte ^1,6^

1. Department of Oncology, Division of Psychosocial Oncology, Cumming School of Medicine, University of Calgary, Calgary, Canada.
2. Department of Radiation Oncology, University of Toronto, Toronto, Canada.
3. Radiation Medicine Program, Princess Margaret Cancer Centre, University Health Network, Toronto, Canada.
4. Division of Haematology & Oncology, Hospital for Sick Children, Toronto, Canada.
5. Department of Paediatrics, University of Toronto, Toronto, Canada.
6. Hematology, Oncology, and Transplant Program, Alberta Children’s Hospital, Calgary, Canada.

### 32.1. Background/Rationale or Objectives/Purpose

Reirradiation is a novel treatment option that may improve clinical outcomes for children with diffuse intrinsic pontine glioma (DIPG) and recurrent brain tumours. The potential impact on health-related quality of life (HRQOL) is not well studied. This study aimed to examine changes in HRQOL and assess the feasibility of longitudinal follow-up with these children and their families.

### 32.2. Methodology or Methods

Since 2021, 18 patients (*M_age_* = 10.03 ± 4.69 years, 50% female) re-irradiated for DIPG or recurrent brain tumours, and their caregivers, were recruited from hospitals across Canada. Self-report and caregiver-proxy PedsQL Generic and Brain Tumor modules were administered pre-reirradiation, post-reirradiation, 2 and 4 months after. Caregivers also completed the PedsQL Family Impact Module. Wilcoxon signed-rank tests compared HRQOL between timepoints.

### 32.3. Impact on Practice or Results

While most comparisons yielded statistically non-significant results, there were distinct trends. Patients’ HRQOL declined from pre-reirradiation to 4 months after (*Z* = −0.365, *p* = 0.715). Caregiver reports showed slight improvements at post-reirradiation for their own (*Z* = −0.297, *p =* 0.767) and child’s HRQOL (*Z* = −0.471, *p =* 0.638). Patients’ pain slightly improved up to 2 months post-reirradiation (*Z =* −0.477, *p* = 0.655) before declining (*Z =* −1.000, *p =* 0.317), while procedural anxiety worsened (*Z =* −1.414, *p =* 0.157) before improving after 2 months *(Z* = −1.000, *p* = 0.317). Movement and balance, along with worry, consistently declined (*Z* = −0.730, *p* = 0.465; *Z* = −1.000, *p* = 0.317). Patients’ self-reported cognition significantly improved from pre- to post-reirradiation (*Z* = −2.371, *p* = 0.018). The data completion rate was 56%.

### 32.4. Discussion or Conclusions

Interim trends indicate potential improved cognition but decreases in HRQOL following reirradiation. Extending this inquiry into earlier parts of treatment may highlight critical timepoints for intervention. Strategies for improving feasibility will be explored.

**46**

## 33. eLearning Modules: Connecting Families with Tools and Resources to Disclose and Support Children and Youth Through a Loved Ones Cancer Diagnosis Using Attachment Theory

Chelsea Butler, Ella Lingafelt, Laurence Gauthier Desrochers, Sandra Palaez

Colorectal Cancer Canada, Montreal, Canada.

### 33.1. Background/Rationale or Objectives/Purpose

A high number of Cancer patients and caregivers either have children of their own or know children that are close to them. Some supporting materials exist on how to disclose a Cancer diagnosis to a child. However, there is a lack of resources that address how to address behaviors that might be unhelpful or misleading after disclosing a Cancer diagnosis to a child or youth using the attachment theory. Furthermore, most resources are written instead of recorded through audio and visual means.

### 33.2. Methodology or Methods

Five video modules were created to address disclosing a Cancer diagnosis to a child or youth, help children and youth cope with a loved one’s Cancer diagnosis, explanation of developmental changes that may occur using the attachment theory, and supporting children and youth when they face grief and loss, whether it is through death or losses such as identity shifts and missed opportunities.

### 33.3. Impact on Practice or Results

The modules will provide Cancer patients, Caregivers, and loved ones with the tools and resources to support a child or youth throughout a loved ones Cancer diagnosis in a trauma-informed way. Attachment theory is an evidence-based practice which ensures that the approach to supporting a child or youth is grounded in research and clinically proven to be successful. These modules can be used by other practitioners for service users, as they are pan-Cancer.

### 33.4. Discussion or Conclusions

Next steps include holding live virtual events to engage families in reflective and fun activities and provide visually appealing written infographics that are easy to digest.

**52**

## 34. A Personalized Approach to Meet the Blood Cancer Community’s Information Needs

Daniela Sinisi ^1,2^, Nadine Prévost ^3^, Pascale Rousseau ^3^

1. Leukemia and Lymphoma Society of Canada, Toronto, Canada.
2. Leukemia and Lymphoma Society of Canada, Montreal, Canada.
3. Leukemia and Lymphoma Society of Canada, Chambly, Canada.

### 34.1. Background/Rationale or Objectives/Purpose

In 2017 and 2022, the Leukemia & Lymphoma Society of Canada (LLSC) conducted two market studies to better understand the experiences and needs of people touched by blood cancers. The studies found that the community faces three major barriers to learning: (1) the complexity of blood cancers, (2) the shock of a diagnosis, and (3) cognitive problems related to the cancer and treatments.

### 34.2. Methodology or Methods

Two important market research we conducted in 2017 and 2022.

### 34.3. Impact on Practice or Results

Our market research found that the community faces three major barriers to learning: (1) the complexity of blood cancers, (2) the shock of a diagnosis, and (3) cognitive problems related to the cancer and treatments.

### 34.4. Discussion or Conclusions

That’s why LLSC is proposing a personalized approach that respects the pace and literacy levels of the people we support.

**78**

## 35. A Multidisciplinary Healthcare Provider Perspective: A Qualitative Study of the Supportive Care Needs of Young Patients with Endometrial Cancer

Sharlane Lau ^1^, Megha Manoj ^1^, Sarah Ferguson ^2,3^, Madeline Li ^1,4^, Dionne Gesink ^5^, Shima Deljoomanesh ^6^, Stephane Laframboise ^2,3^, Karen Glass ^3^, Jackie Bender ^7^, Aliza Panjwani ^1,4^, Rachel Kim ^2,3^

1. Department of Supportive Care, Princess Margaret Cancer Centre, University Health Network, Toronto, Canada.
2. Department of Obstetrics and Gynaecology, Princess Margaret Cancer Centre, University Health Network, Toronto, Canada.
3. Department of Obstetrics and Gynaecology, University of Toronto, Toronto, Canada.
4. Department of Psychiatry, University of Toronto, Toronto, Canada.
5. Dalla Lana School of Public Health, University of Toronto, Toronto, Canada.
6. Princess Margaret Cancer Centre, University Health Network, Toronto, Canada.
7. Department of Supportive Care, Princess Margaret Cancer Centre, Toronto, Canada.

### 35.1. Background/Rationale or Objectives/Purpose

Supportive care improves patient-reported outcomes and quality of person-centred care. However, tailored supportive care services are lacking for patients of childbearing age with early endometrial cancer (EC), despite rising incidence in patients aged 30-49. While total hysterectomy is standard care, a subset of patients may be eligible for fertility-sparing treatment, though surgery may be ultimately required. Younger adults with EC (YAEC) may experience significant distress due to the dual threat of cancer and infertility, underscoring the importance of addressing their unmet psychosocial needs. Consequently, we explored the perspectives of multidisciplinary healthcare providers (HCPs) on the supportive care needs of YAEC, an underserved population.

### 35.2. Methodology or Methods

Spanning psychosocial oncology, oncology, and nursing disciplines, six HCPs providing direct care to YAEC (target *N* = 8–10) completed qualitative interviews. Additional interviews are planned with reproductive endocrinology and infertility specialists. HCPs were recruited from a cancer centre and oncofertility clinics in Toronto. Data collection and thematic analysis are ongoing and iterative.

### 35.3. Impact on Practice or Results

Preliminary themes reveal gaps in supportive care across multiple domains, including lack of emotional support (e.g., lack of tailored psychological interventions and peer support), key knowledge gaps (e.g., about diagnosis, treatment, and fertility) and health systems needs/challenges (e.g., coordinating multidisciplinary care). Across interviews, providers emphasized the importance of considering the developmental context of young adulthood in meeting the unique needs of YAEC patients.

### 35.4. Discussion or Conclusions

HCPs’ perspectives highlight the need for tailored supportive care for YAEC, including psychosocial services, informational resources, and patient navigation to improve care coordination. Findings may also inform the development of psychoeducational tools and resources.

**98**

## 36. The Next Chapter: Enhancing Transition Pathways from Pediatric to Adult Oncology Care

Samara Esack ^1^, Becca Cote ^2^, Chana Korenblum ^2,3,4^

1. Daphne Cockwell School of Nursing, Toronto Metropolitan University, Toronto, Canada.
2. The Hospital for Sick Children, Toronto, Canada.
3. Princess Margaret Cancer Centre, Toronto, Canada.
4. Temerty Faculty of Medicine, University of Toronto, Toronto, Canada.

### 36.1. Background/Rationale or Objectives/Purpose

The transition from pediatric to adult oncology care can present significant challenges for adolescents and young adults with cancer (AYA), including difficulty adhering to treatment or follow-up. Few formal transition pathways exist for AYA within five years of active cancer treatment across Canadian pediatric centres. This leaves many patients and families ill-prepared to handle the differences between the two healthcare systems.

### 36.2. Methodology or Methods

A needs assessment was conducted at a large pediatric hospital in Ontario as part of a broader initiative to improve the transition process for AYA. Twenty-one semi-structured interviews were conducted with healthcare professionals (physicians, nurses, nurse practitioners, social workers, neuropsychologists, administrators), 17 from the pediatric hospital and five from an adult cancer centre. Participants were asked about transition barriers, facilitators, and areas for improvement.

### 36.3. Impact on Practice or Results

Themes included: (1) supporting a gradual shift from family-centred to patient-centred care to prepare AYA to manage their health independently, (2) framing each clinical interaction as an opportunity to discuss transition, manage expectations, address uncertainty, and be mindful of language used to describe adult care, and (3) acknowledging system-level changes, including (a) availability of funding and supports, (b) shifting from hospital- to community-based resources, and (c) ensuring primary care provision.

### 36.4. Discussion or Conclusions

Findings will help inform the development of formalized transition pathways, including integrating transition discussions into routine care early on, leveraging the electronic health record to improve feasibility, and offering educational materials to staff. Next steps include interviews with patients and families, and healthcare professionals from other centres, as well as selection, implementation and evaluation of a formal transition-preparedness tool.

**104**

## 37. Health Interdependence in Children with Cancer & Their Parents: A Multimethod, Longitudinal Study

Stephanie Nanos ^1,2,3^, Gary Rodin ^1,2^, Lindsay Jibb ^1,3^

1. Temerty Faculty of Medicine, Institute of Medical Science, University of Toronto, Toronto, Canada.
2. Department of Supportive Care, Princess Margaret Cancer Centre, University Health Network, Toronto, Canada.
3. Child Health Evaluative Sciences, The Hospital for Sick Children, Toronto, Canada.

### 37.1. Background/Rationale or Objectives/Purpose

A childhood cancer diagnosis is disruptive to families, profoundly and negatively impacting their physical/psychosocial well-being. For children, initial disruptions may arise from intensive treatment, symptom burden, and disruption to normal childhood activities. Parents are increasingly expected to assume lead roles in complex care while managing existing responsibilities and faced with the constant threat that their child will suffer or die. These experiences are often separately assessed despite multiple theories of interdependence in close/familial relationships proposing that stress and coping in parent-child dyads are reciprocal and dynamic. Thus, we aim to holistically characterize the mutual experience of suffering in children with cancer and their parents.

### 37.2. Methodology or Methods

This will be a prospective, multimethod study conducted at the Hospital for Sick Children (Toronto). Participants will be parents (>18 yo) of a child (8–18 yo) within 6-months of a new or relapse cancer diagnosis (N = 120 dyads).

### 37.3. Impact on Practice or Results

Dyads will self-report valid and reliable measures of physical/psychosocial health (e.g., symptom burden, psychological distress) at baseline and 3-months. A subset (n = 20 dyads) varying in demographic and child disease characteristics will participate in semi-structured interviews about the perceived impact of cancer on the other. Quantitative data will be analyzed using Actor-Partner Interdependence Models and qualitative data with an established integrative technique.

### 37.4. Discussion or Conclusions

This will be the first study to describe the extent to which a child-parent dyad member’s health status and experiences affects the other. This understanding will provide needed guidance to the design of family-centered care for those affected by cancer, and shape future research and clinical practices.

**106**

## 38. Bringing Care Closer to Home: The Implementation of an Adolescent and Young Adult Program at a Regional Cancer Centre

Samantha Scime ^1,2,3^, Alisha Kassam ^2,3^

1. Princess Margaret Cancer Centre, Toronto, Canada.
2. Stronach Regional Cancer Centre, Toronto, Canada.
3. University of Toronto, Toronto, Canada.

### 38.1. Background/Rationale or Objectives/Purpose

Adolescents and young adults (AYA) need specialized care aimed at addressing the impact of cancer on their age and stage of development. While tailored resources can be available in urban centers, there is a critical need to expand programming to rural and community settings. Leveraging the Princess Margaret (PM) Cancer Network, the Stronach Regional Cancer Centre (SRCC) was well positioned to establish the first embedded AYA program at a regional cancer centre.

### 38.2. Methodology or Methods

Using a hub and spoke model, the program is led by a medical director at SRCC and clinical nurse specialist (CNS). The program includes an initial consultation and follow up with the CNS, as well as, the provision of tailored resources, psychosocial support and peer connection activities.

### 38.3. Impact on Practice or Results

Since the initiation of the program in fall 2023, over 100 patients have received tailored AYA care. Key innovations include an automatic referral process for patients (age 18–43), as well as, the development of a resource navigator, asset map, and standardized workflows in fertility preservation, sexual health and mental health. Increased awareness of AYA care domains has been established through staff education initiatives and peer connection is fostered through virtual and in-person AYA events.

### 38.4. Discussion or Conclusions

This presentation will provide an overview of the SRCC AYA program including metrics to date and lessons learned to guide AYA program development at other centres. Preliminary findings from patient and staff satisfaction surveys will be highlighted and future directions shared.

**108**

## 39. Evolution and Equity: Adapting an Existing AYA Program Through Education

Simone Kurup

Princess Margaret Cancer Centre, Toronto, Canada.

### 39.1. Background/Rationale or Objectives/Purpose

Since 2014, the Princess Margaret Cancer Centre (PMCC), located in Toronto, Ontario has provided specialized care for patients aged 18–39 through the Adolescent and Young Adult (AYA) Program. Historically, an AYA patient needed to be receiving care at PMCC to be eligible for the program. In 2023, the program expanded, accepting patients outside of PMCC through the creation of The Ontario Cancer Adolescent and Young Adult Program (OCAP). The driving force of this expansion is to reduce health inequities in accessing AYA-specialized care and services.

### 39.2. Methodology or Methods

In its first year, OCAP focused on the launch of a needs assessment and environmental scan, informing the adaptation of the existing program to serve a growing and diverse population and building relationships with healthcare providers (HCP) across various cancer centers. Through interviews with external HCP, the desire for more education to enhance competency in the AYA care domains was highlighted as a priority which led to OCAP hosting its inaugural AYA HCP Training Day.

### 39.3. Impact on Practice or Results

The AYA HCP Training Day consisted of 64 attendees in person and 86 attendees online from 18 different cancer centers across the country. A range of 16 different types of HCP attended the day with the goal of competency building in specialized AYA care.

### 39.4. Discussion or Conclusions

Evaluations of the AYA HCP Training Day were conducted by the attendees and presenters, indicating the interest in an annual conference as well as areas for growth for the day. Lessons learned will be highlighted.

**127**

## 40. Financial Impacts in Young Adults with Cancer

Reanna George ^1^, Krista Greeley ^1^, Pam Crotty ^2^, Geoff Eaton ^2^, Sheila Garland ^1,3^

1. Memorial University of Newfoundland, St. Johns, Canada.
2. Young Adult Cancer Canada, St. Johns, Canada.
3. Beatrice Hunter Cancer Research Institute, Halifax, Canada.

### 40.1. Background/Rationale or Objectives/Purpose

Young adults (YAs), defined as those between the ages of 18 and 39 years old, experience more financial stress as a consequence of cancer treatment. The current study examined the personal financial impacts of cancer on YAs in Canada.

### 40.2. Methodology or Methods

Young adults between the ages of 18-39 with cancer living in Canada were invited to complete a survey on the financial, psychological, social, and physical impact of cancer and quality of life. Select items of the Patient Self-Administered Financial Effects (P-SAFE) of Cancer measure was used to measure the financial burden of cancer. Descriptive statistics were used to quantify the financial impacts of cancer.

### 40.3. Impact on Practice or Results

Participants (N = 433, Mage = 33.53, 75% women) completed the survey. 48.4% of participants reported spending more than $100 per month out of pocket on cancer expenses, and 27.8% missed more than 16 weeks of work due to cancer. More than half (55.6%) reported missing less than 4 weeks of work due to cancer with 17.3% of YAs being responsible for 100% of their household income. To cover cancer-related costs, 35.6% reduced spending on entertainment, including family events, 23.1% reduced their monthly savings to accommodate cancer costs, 20.3% had to use their registered savings, 15% reduced spending on meals and accommodations, and 6.5% of YAs responded having to take a loan. To accommodate cancer related issues, 2.1% of YAs had to renovate their home.

### 40.4. Discussion or Conclusions

Overall, cancer costs are a burden on some young adults with cancer and the financial impacts need to be further explored.

**147**

## 41. Supportive Care Needs Among Individuals with Endometrial Cancer: A Systematic Review

Megha Manoj ^1^, Sharlane C.L Lau ^1^, Jennifer Yu ^1^, Sarah Daniels ^1^, Rouhi Fazelzad ^2^, Soyoun Rachel Kim ^3,4^, Sarah E. Ferguson ^3,4^, Shima Deljoomanesh ^3^, Stefane Laframboise ^3,4^, Madeline Li ^1,5^, Aliza A. Panjwani ^1,5^

1. Department of Supportive Care, Princess Margaret Cancer Centre, University Health Network, Toronto, Canada.
2. Library and Information Services, Princess Margaret Cancer Centre, University Health Network, Toronto, Canada.
3. Division of Gynecologic Oncology, Princess Margaret Cancer Centre, University Health Network, Toronto, Canada.
4. Department of Obstetrics and Gynecology, University of Toronto, Toronto, Canada.
5. Department of Psychiatry, University of Toronto, Toronto, Canada.

### 41.1. Background/Rationale or Objectives/Purpose

With increases in overall five-year survival rates, there is a growing population of patients with endometrial cancer (EC); however, little is known about their supportive care needs (SCNs). While the median age of diagnosis is 61, EC incidence is also increasing among patients younger than 45, who may experience additional unique challenges. This systematic review aims to identify the SCNs of patients with EC and, where available, delineate the distinct needs of younger adults within this population.

### 41.2. Methodology or Methods

Following the Joanna Briggs Institute guidelines for conducting mixed-methods reviews, a comprehensive search of seven bibliographic databases was conducted to identify literature on SCNs in adult EC patients/survivors, encompassing publications up to 2023. Peer-reviewed empirical articles in English were included without restrictions on country, date, disease stage, or treatment. Intervention studies and those involving mixed cancer samples without EC-specific analyses were excluded. Results were managed using Covidence. Abstracts were screened by three reviewers; full-text screening is ongoing. Eligible studies will undergo quality assessment, data extraction, and narrative and tabular synthesis.

### 41.3. Impact on Practice or Results

OF 11,002 reviewed abstracts, 1023 studies were selected for full-text screening. Of 224 full-text studies screened thus far, 77 are selected for data extraction. Preliminary findings highlight physical, psychological and interpersonal as the top unmet needs of individuals with EC.

### 41.4. Discussion or Conclusions

The results of this review will elucidate the SCNs of the growing EC population to identify potential gaps in supportive care. Findings will have important implications for the development of tools and resources to address unmet needs, including in younger patients with EC.

**148**

## 42. Adolescent and Young Adult Cancer Care: Insights from Patient Satisfaction Survey for Program Enhancement

Mackenzie Jansen ^1^, Madyson Shauman ^1^, Ian Scott ^1^, CindyMarie Mack ^1^, Caitlin Woodrow ^1^, Maryam Shamloo ^1,2^, Craig Harlos ^3,4^, Patricia Bocangel ^5^, Lorena Gerl ^1^, Sapna Oberoi ^6,7,2^

1. Department of Patient and Family Support Services, CancerCare Manitoba, Winnipeg, Canada.
2. CancerCare Manitoba Research Institute, CancerCare Manitoba, Winnipeg, Canada.
3. Department of Internal Medicine, University of Manitoba, Winnipeg, Canada.
4. Department of Hematology and Medical Oncology, CancerCare Manitoba, Winnipeg, Canada.
5. Departments of Community Oncology Program and Patient Experience, Winnipeg, Canada.
6. Department of Pediatric Hematology/Oncology, CancerCare Manitoba, Winnipeg, Canada.
7. Department of Pediatrics and Child Health, Max Rady College of Medicine, Winnipeg, Canada.

### 42.1. Background/Rationale or Objectives/Purpose

The Adolescent and Young Adult (AYA) Program at CancerCare Manitoba is dedicated to supporting individuals aged 15–39 who are navigating a cancer diagnosis. AYAs encounter unique medical, social, and developmental challenges that differ significantly from those faced by pediatric and older adult patients. To optimize care delivery, the program seeks to continuously evaluate its effectiveness and align services with the needs of its patients. This study will determine patient satisfaction with the program and explore the feasibility of using online surveys for ongoing feedback to enhance service delivery.

### 42.2. Methodology or Methods

A mixed-methods cross-sectional survey will be conducted between December 2024 to June 2025 utilizing the validated Client Satisfaction Questionnaire (CSQ) to evaluate participant satisfaction and identify opportunities for program improvement. Quantitative data from the CSQ will be supplemented with qualitative insights to comprehensively understand the program’s impact. Additionally, the study will examine the feasibility of implementing online surveys as an effective method for gathering feedback from the AYA population.

### 42.3. Impact on Practice or Results

The findings will help determine whether online satisfaction surveys are feasible for engaging AYAs in program evaluation. Results from this survey will identify the program’s strengths and opportunities to align program services with the evolving needs of AYAs, enhancing person-centred care.

### 42.4. Discussion or Conclusions

This study will explore the value of utilizing patient satisfaction surveys in identifying strengths and areas for growth within the AYA program and highlight the value of integrating patient feedback into service development and enhancement.

**151**

## 43. Where Cancer & Fertility Collide: Focus Group Insights into the Lived Experiences of Young Patients with Endometrial Cancer

Aliza Panjwani ^1,2^, Soyoun Rachel Kim ^1,2^, Sharlane Cheuk Ling Lau ^1^, Megha Manoj ^1^, Sarah Ferguson ^1,2^, Madeline Li ^1,2^, Dionne Gesink ^2^, Stephane Laframboise ^1,2^, Shima Deljoomanesh ^1^, Jackie Bender ^3^, Karen Glass ^2,4^

1. Princess Margaret Cancer Centre, Toronto, Canada.
2. University of Toronto, Toronto, Canada.
3. Department of Supportive Care, Princess Margaret Cancer Centre, Toronto, Canada.
4. CREATE Fertility, Toronto, Canada.

### 43.1. Background/Rationale or Objectives/Purpose

Endometrial cancer (EC) is projected to become the third most common cancer in women in developed countries by 2030, with early-onset cases rising in those aged 30–45. While fertility-sparing therapy is an option for some young patients with EC or atypical hyperplasia (YAEC/AH), 25–40% may have an incomplete response and ultimately require hysterectomy. The cancer diagnosis, treatment risks, and threat to fertility result in compounding psychological vulnerability. Through focus groups, this study explores the lived experiences of YAEC/AH to better understand the sources and nature of fertility distress in this population.

### 43.2. Methodology or Methods

A descriptive focus group design using purposive sampling was employed to recruit a diverse participant pool. Three focus groups have been conducted to-date (age_range_ = 29–43; 44% Asian, 22% White, 22% multi-racial; 11% Hispanic), including YAEC/AH at varying illness stages and a caregiver group, with recruitment completed for two additional focus groups. Focus groups were approximately two hours, recorded, and transcribed verbatim. Utilizing thematic analysis (to be completed by March 2025), preliminary themes were identified in an iterative process.

### 43.3. Impact on Practice or Results

Preliminary themes contributing to sources of fertility distress include sociocultural pressures and norms (e.g., gender roles), impacted self-concept and identity, disconnect/isolation from same-age peers, and social comparisons and triggers. Themes related to the nature of fertility distress include uncertainty, anxiety, and [anticipatory] grief and loss.

### 43.4. Discussion or Conclusions

Findings will inform a theoretical model of fertility distress, laying the foundation for developing a tailored group psychosocial intervention. The results may be applicable to other young adult patients at risk of impaired fertility due to cancer.

**166**

## 44. A Comprehensive Examination of Social Information Processing on Social Adjustment in Survivors of Pediatric Brain Tumor

Hailey Zwicker ^1^, Courtney Tromburg ^2^, Mehak Stokoe ^1^, Caitlin Forbes ^1^, Heide Adham ^1^, Gregory Guilcher ^1^, Taryn Fay-McClymont ^3^, Oluwaseyi Lawal ^1^, Lucie Lafay-Cousin ^1^, Keith Yeates ^1^, Kevin Krull ^4^, Fiona Schulte ^1^

1. University of Calgary, Calgary, Canada.
2. University of Alberta, Edmonton, Canada.
3. University of British Columbia Okanagan Campus, Kelowna, Canada.
4. St. Jude Children’s Research Hospital, Memphis, USA.

### 44.1. Background/Rationale or Objectives/Purpose

SURVIVORS of pediatric brain tumor (SPBT) often experience late effects including challenges in social adjustment. This study explored whether components of social information processing (SIP; i.e., executive and affective processes) are associated with social adjustment, and if these associations differ between SPBT and healthy children.

### 44.2. Methodology or Methods

Thirty-five SPBT (M_age_ = 11.61 ± 2.96, 51.4% male), sex—and age-matched controls, and their caregivers were recruited and completed a one-time research session at Alberta Children’s Hospital.

Participants completed the *Pediatric Evaluation of Emotions, Relationships, and Socialization* tool to assess affective processing and the Wechsler Intelligence Scale for Children Fifth Edition to assess domains of executive processing. Caregivers reported on parent-proxy executive processing by completing the *Behavior Rating Inventory of Executive Functions* and the *ADHD Rating Scale-5 for Children and Adolescents*, and rated social adjustment using the *Adaptive Behaviors Assessment System* (ABAS-3) social subscale. Exploratory factor analyses and theoretical knowledge were used to identify SIP factors to be included in the linear regression analyses predicting social adjustment.

### 44.3. Impact on Practice or Results

Three SIP factors were identified: (1) global executive functioning, (2) processing speed, and (3) affective processing. The ABAS-3 linear regression including the three SIP factors and group (SPBT vs. controls) identified that SPBT is a significant predictor of poorer social adjustment (β = −7.88, *p* < 0.05). Also, poorer global executive functioning (β = −0.51, *p* < 0.005) is significantly associated with poorer social adjustment. Other SIP factors were not significant.

### 44.4. Discussion or Conclusions

This study is the first to identify specific aspects of SIP associated with the social adjustment of SPBT. The results may guide future research and interventions to improve the social well-being of these survivors.

**177**

## 45. Life Redefined: Understanding the Young Adult Brain Tumour Experience Through Digital Storytelling

Michael Lang ^1,2,3^, Natasha Bilau-Howie ^3^, Mani Prabhu ^3^

1. University of Calgary, Calgary, Canada.
2. Mike Lang Stories, Calgary, Canada.
3. Common Language Digital Storytelling, Calgary, Canada.

### 45.1. Background/Rationale or Objectives/Purpose

Over the past 15 years there has been increasing recognition of the differential psychosocial impact of cancer by age, with the young adult age-range experiencing elevated levels of clinical distress, depression, and anxiety relative to all other age groups of cancer survivors. Being an young adult at the time of diagnosis and treatment is a significant risk factor for poor health-related quality of life and post-traumatic stress in cancer survivorship. Young adult-focused interventions delivered on platforms that are acceptable and accessible to this population are urgently needed and one such intervention could be Digital Storytelling (DST). Over the past three years, 18 young adults with brain tumours have participated in an online Digital Storytelling workshop to create short-films about their brain tumour experiences. These stories are then shared publicly online and in other advocacy and educational contexts by the storytellers themselves.

### 45.2. Methodology or Methods

This presentation will begin by providing a very brief overview of the young adult brain tumour digital storytelling workshop process and will then screen two digital stories with the storytellers in attendance. After each story, the storyteller will talk about the main themes in their story as well as their experiences of creating the story and how it has impacted their healing process.

### 45.3. Impact on Practice or Results

Attendees will gain a greater understanding of the young adult brain tumour experience and how Digital Storytelling could be used as a developmentally-appropriate, meaning-making activity for young adults with cancer. They will leave with direct access to the Life Redefined series of 18 young adult brain tumour survivor-created digital stories that can be shared with other young adults in clinical practice contexts.

### 45.4. Discussion or Conclusions

This presentation will focus on the lived-experiences of two storytellers as they share directly with the audience the impact of the digital storytelling experiences on their lives. Through the digital stories and commentary, the audience will see how an appropriately designed and facilitated DST experience can support young adult brain tumour survivors to cultivate understanding of, and craft meaning from their cancer experience.

**185**

## 46. Empowering Lives Across Cancer Trajectories: Evaluating the Impact of the Cancer Patient Empowerment Program (CancerPEP) on Psychological Distress in a Multicancer Population

Gabriela Ilie, Robert Rutledge

Dalhousie University, Halifax, Canada.

### 46.1. Background/Rationale or Objectives/Purpose

Psychological distress is a common and debilitating consequence of a cancer diagnosis, adversely affecting patients’ quality of life, treatment adherence, and overall health outcomes. Despite advances in oncology care, integrating psychosocial interventions into standard cancer treatment remains limited. To address this gap, the Cancer Patient Empowerment Program (CancerPEP) was developed as a home-based, multimodal intervention incorporating physical activity, dietary guidance, and social support. Building on the success of the Prostate Cancer-Patient Empowerment Program (PC-PEP), CancerPEP aims to reduce psychological distress across various cancer types and stages. This study evaluates the program’s effectiveness in a diverse cancer population, including patients with breast, colorectal, bladder, and kidney cancers, where unmet psychosocial needs remain significant.

### 46.2. Methodology or Methods

This single-site, crossover randomized clinical trial enrolled 104 cancer patients diagnosed with breast, bladder, GU, or kidney cancers. Participants were randomized to receive CancerPEP, with or without a Heart Rate Variability (HRV) biofeedback monitor, stratified by metastatic status and baseline psychological distress. Psychological distress was assessed using the Kessler Psychological Distress Scale (K10) at baseline, 6 months, and 12 months. Key covariates included age, comorbidities, cancer stage, treatment type, and mental health medication use. An exploratory sub-analysis was conducted for the breast cancer subgroup based on available sample size. The trial is registered at ClinicalTrials.gov (NCT05508412).

### 46.3. Impact on Practice or Results

The HRV biofeedback monitor did not significantly affect psychological distress outcomes in the full cohort or the breast cancer subgroup. However, participation in CancerPEP resulted in a significant reduction in psychological distress over time. In the full cohort, distress was significantly reduced from baseline at 6 months (adjusted Odds Ratio [aOR] = 2.64, 95% Confidence Interval [CI]: 1.53–4.56) and at 12 months (aOR = 2.94, 95% CI: 1.62–5.30). Similar reductions were observed in the breast cancer subgroup at 6 months (aOR = 2.25, 95% CI: 1.24–4.08) and 12 months (aOR = 2.73, 95% CI: 1.35–5.52). Integrating CancerPEP into standard oncology care has the potential to bridge the gap between medical treatment and psychosocial support. Its home-based, scalable design addresses the practical challenges of in-person interventions. By significantly reducing psychological distress in diverse cancer populations, CancerPEP provides a replicable and sustainable model that can be adapted across cancer care settings worldwide. Its integration could improve patient outcomes, enhance quality of life, and support long-term survivorship care.

### 46.4. Discussion or Conclusions

CancerPEP significantly reduced psychological distress in cancer patients, demonstrating consistent improvements across different cancer types and stages. Its success builds on the established effectiveness of PC-PEP and highlights the feasibility of expanding such interventions beyond prostate cancer. By addressing psychosocial needs through a holistic, multimodal approach, CancerPEP presents a valuable, evidence-based model for global cancer care. Future evaluations at 24 months will provide deeper insight into its long-term benefits and potential role in routine oncology practice worldwide.

**186**

## 47. Unlocking the Black Box of Psychosocial Interventions: A Realist Evaluation of the Prostate Cancer-Patient Empowerment Program (PC-PEP)

Robert Rutledge, Gabriela Ilie

Dalhousie University, Halifax, Canada.

### 47.1. Background/Rationale or Objectives/Purpose

Men diagnosed with prostate cancer face significant psychological distress due to the physical, emotional, and social challenges associated with their diagnosis and treatment. While randomized clinical trials (RCTs) establish the efficacy of psychosocial interventions, they often fail to explain *why* and *how* certain outcomes occur. To address this “black box” problem, this study applied a realist evaluation framework to the Prostate Cancer-Patient Empowerment Program (PC-PEP), investigating the contextual factors and mechanisms driving mental health outcomes among participants in the Phase 3 PC-PEP RCT. The study explored heterogeneity in outcomes by examining key research questions:

1. What patient-related contexts and mechanisms explain differences in mental health outcomes between intervention and control groups at six months post-randomization?
2. What contextual factors and mechanisms account for differences in outcomes between early intervention and delayed (control) participants?

### 47.2. Methodology or Methods

This study integrated quantitative and qualitative data from the PC-PEP Phase 3 RCT, which enrolled 128 men aged 50–82 randomized to an immediate intervention or waitlist control group. Psychological distress, measured using the Kessler Psychological Distress Scale (K10), was assessed at baseline, six months, and 12 months. To explore mechanisms underlying quantitative outcomes, 43 participants (33%)—25 from the early intervention group and 18 from the delayed group—engaged in semi-structured qualitative interviews. These interviews explored participants’ lived experiences, identifying facilitators and barriers to program engagement. Context-mechanism-outcome (CMO) configurations were developed by triangulating findings from the RCT with thematic qualitative analysis.

### 47.3. Impact on Practice or Results

#### 47.3.1. Impact on Practice

The realist evaluation approach highlights how PC-PEP can be tailored to optimize its implementation and effectiveness in clinical settings. Key practice recommendations include: Early Intervention: Offering PC-PEP at the point of diagnosis may prevent distress escalation. Family and Partner Involvement: Incorporating partners and family members enhances emotional support and adherence. Cultural and Social Adaptation: Culturally sensitive adaptations can improve uptake among diverse populations. Flexible Delivery Models: Hybrid (in-person and digital) program delivery can ensure accessibility for rural, working, and minority populations.

#### 47.3.2. Results

The evaluation identified several key mechanisms influencing mental health outcomes: Psychological Contexts: Fear of diagnosis, intimacy education, multidimensionality (better fit), length of program (6 months plus ongoing monthly support) and communication style played critical roles in reducing mental distress. Social Support: Partner involvement, family engagement, and buddy systems strengthened emotional resilience and program adherence. Structural Barriers: Employment status, time constraints, cultural beliefs, and the COVID-19 pandemic significantly shaped participation and outcomes. Retired or unemployed participants demonstrated higher engagement, while working individuals faced logistical challenges. Clinical Credibility: The involvement of clinical experts fostered trust, motivation, and consistent program engagement.

Discussion or Conclusions

Findings emphasize that tailoring psychosocial interventions requires addressing the complex interplay of individual, social, and environmental contexts. Future iterations of PC-PEP should include enhanced flexibility, cultural sensitivity, and early engagement strategies. Policymakers should consider integrating PC-PEP into routine prostate cancer care, leveraging remote and digital delivery to expand access. These findings also suggest that PC-PEP could be adapted for other cancer types, ensuring its broader applicability and public health impact. Ongoing research will focus on expanding the program’s reach to underserved populations and refining its delivery in diverse healthcare settings.

## 48. Final Category: C. Community-Based and Volunteer Cancer Care Services

19

## 49. Participants’ Perspectives of “Drop-In” Compared to Closed-Group Community-Based Bereavement Support Programs

Yoojung Kim ^1^, Aditi Nanjappa ^2^, Carmen Loiselle ^1,2,3^

1. Division of Experimental Medicine, Faculty of Medicine and Health Sciences, McGill University, Montreal, Canada.
2. Segal Cancer Centre, CIUSSS du Centre-Ouest-de l’Île-de Montréal, Montreal, Canada.
3. Department of Oncology, Faculty of Medicine and Health Sciences, McGill University, Montreal, Canada.

### 49.1. Background/Rationale or Objectives/Purpose

Having opportunities to access bereavement support programs tailored to individual needs and preferences is an important aspect of person-centred cancer care. *Hope & Cope*, a community-based organization in Montreal, Quebec, supports individuals across the cancer trajectory from diagnosis to bereavement. Led by professionals and volunteers, these programs are offered as “drop-in” or closed groups. This qualitative study sought to explore how participants perceived two types of bereavement support programs: *Mourning Walk* (where participants can join at any time and indefinitely) or *Living with Loss* (an 8-week closed-group program).

### 49.2. Methodology or Methods

Participants (N = 18) were either enrolled in *Mourning Walk* (n = 7) or *Living with Loss* (n = 11), at least 18 years old, lost a loved one to cancer, and communicated in English. The study was conducted online. Program registrants were asked for permission to be contacted for research purposes. Interested participants were recruited by phone and screened for eligibility. E-questionnaires gathered information on socio-demographics, participation history and program modality preferences. Semi-structured Zoom interviews were conducted at program completion to explore affective attitude, potential burden and perceived effectiveness.

### 49.3. Impact on Practice or Results

Data collection will be completed by December 2024. Thematic analysis will be used to analyze verbatim transcripts and determine preferences related to program formats and features by bereaved individuals.

### 49.4. Discussion or Conclusions

Study findings provide opportunities to document and adapt key features of these bereavement programs to better support program users. Characterizing the strengths and gaps in open and closed group formats can enhance our understanding of their significance according to users’ needs and preferences.

**20**

## 50. Participants’ Evolving Perceptions While Attending a Bereavement Program: Qualitative and Quantitative Findings

Yoojung Kim ^1^, Carmen Loiselle ^1,2^

1. Division of Experimental Medicine, Faculty of Medicine and Health Sciences, McGill University, Montreal, Canada.
2. Department of Oncology, Faculty of Medicine and Health Sciences, McGill University, Montreal, Canada.

### 50.1. Background/Rationale or Objectives/Purpose

Psychosocial support provided by community-based organizations can be a significant contributor to the wellbeing of individuals who are navigating cancer. One such organization is *Hope & Cope* (in Montreal, Quebec) which provides support for individuals diagnosed with cancer and their informal caregivers. Herein, a comprehensive evaluation over program delivery timelines was undertaken to document bereaved individuals’ perceptions of “*Living with Loss*” as it evolved across time. *Living with Loss*, an 8 bi-weekly in-person program, is open to individuals who have lost a loved one to cancer within the past 2 years.

### 50.2. Methodology or Methods

Participants (n = 11) who were at least 18 years old and registered for the *Living with Loss* program were invited to participate in the study. The study was completed online. A mixed-method design with self-report e-questionnaires was used to measure levels of hopefulness (Herth Hope Index) and coping (Brief Cope Scale) pre- and post-program completion. Semi-structured interviews were also conducted before, during and after program completion. E-questionnaires were completed on participants’ home computers or smart phones. In-depth interviews took place virtually using the Zoom platform.

### 50.3. Impact on Practice or Results

Data collection will be completed by December 2024. Paired sample t-tests will comparatively evaluate participants’ levels of hopefulness and coping pre- and post-program attendance. Qualitative data from semi-structured interviews will be analyzed using thematic analysis.

### 50.4. Discussion or Conclusions

Study findings can comprehensively document contributions of this community program to hope and coping. The exploration of participants’ views over time can further inform how the program can be adjusted to truly meet users’ needs and preferences.

**141**

## 51. Lost but Found—Finding Sense and Meaning Trough Participation in an Online Art Therapy Support Group

Iulia Udrea ^1,2^, Mara Oprea ^2^, Camelia Moraru ^1^

1. Medisprof Cancer Center, Cluj-Napoca, Romania.
2. Asociatia Proiect A Zece, Cluj-Napoca, Romania.

### 51.1. Background/Rationale or Objectives/Purpose

Art-therapy groups have a proven impact on reducing distress and improving the oncological patients’ quality of life [Collie&all, 2006, Ennis&all, 2018, Bosman&all, 2021]. Therefore, we conducted an online art therapy support group over 2 years, studying the group impact on patients and the community—previously analyzed and presented (CAPO 2023, IPOS 2022)—and, more recently, the patients’ journeys in the process of rediscovering sense and meaning for their life after an oncological diagnosis.

### 51.2. Methodology or Methods

The online group started in 2023 with monthly art therapy activities facilitated by 2 architects, 2 psychologists, and 1 artist, for 15 voluntary participants, male and female. The growing cohesion and openness of the group revealed one major theme—finding new sense and meaning in their experience with oncological diagnosis—expressed through artistic objects. 5 participants’ evolution is documented through interviews, artwork photos, and video narratives presenting an interpretation of their journey and the groups’ contribution.

### 51.3. Impact on Practice or Results

Participating in artistic-based support groups empowered participants to become resources and promote the need for this emerging practice in Romania.

### 51.4. Discussion or Conclusions

Specific art themes could reinforce the construction of meaning and finding hope in groups. Post-group follow-up could support participants in a constant commitment to their discovered sense and meaning and empower them to support these activities for other patients. More qualitative and quantitative research is needed to find the best moment for group completion and long-term support. Keeping the group informed regarding participants’ evolution and possible recurrence or death of members should be addressed.

**156**

## 52. How Healing and Well-Being Are Enhanced on Retreat: Brooksong’s Innovative Approach to Psychosocial and Supportive Care

Barb Smith-Morrison, Nancy Gosse

Brooksong Retreat and Cancer Support Centre, Haliburton, Canada.

### 52.1. Background/Rationale or Objectives/Purpose

Brooksong Retreat and Cancer Support Centre addresses a critical gap in psychosocial cancer care by offering an immersive community retreat experience that includes evidence-informed well-being modalities for both the person with a diagnosis and a caregiver or key support partner.

The components of the retreat serve as supportive adjuncts to medical treatment and provide an introduction to a diverse set of holistic practices and tools to address psychological, emotional, physical and spiritual needs of cancer patients and their loved ones.

### 52.2. Methodology or Methods

An early partnership with the Patterson Institute for Integrative Oncology Research guided the choices of modalities included in retreats. Modalities included are found to have a positive impact on either the side effects of cancer treatment and/or the side effects of the trauma of being ill.

Retreat modalities include expressive arts, yoga, meditation, guided nature connection, music, massage, facilitated and informal group sessions (together and separately for those with a diagnosis and caregivers) and a plant-based menu.

### 52.3. Impact on Practice or Results

Brooksong’s programs are unique across Canada because of the inclusion of caregivers and because retreats are provided at no cost to participants. Participants report that one of the most beneficial impacts is the intentional care paid to the creation of an enduring and compassionate community in which to explore modalities; one which they have reported leaves them with a sense of safety and empowerment relating to how they approach their healing experience.

### 52.4. Discussion or Conclusions

We have a robust evaluation process that consistently demonstrates that retreat participation leads to experiences of belonging, meaningful connection, renewal, and empowerment.

Future directions would include an expanded evaluation process that also monitors ongoing engagement with modalities and community several months past the retreat experience.

**158**

## 53. A Community-Based Approach to Supporting Patients with Incurable/Chronic Cancer

Judi Perry Brinkert, Gloria Ghabeli

Wellspring Cancer Support, Toronto, Canada.

### 53.1. Background/Rationale or Objectives/Purpose

Responding to feedback from both healthcare partners and patients diagnosed with an incurable cancer, Wellspring Cancer Support identified the need to recognize the uniqueness of these patients within it’s program roster. Patients coping with an incurable cancer felt a need to censor what they shared in groups when individuals on a curative path were present. They did not feel ready to participate in programming that was focused on discussions related to later stage illness. A specialized stream of programming would address this gap in supportive programming.

### 53.2. Methodology or Methods

Two patient focus groups were held to understand their experience at Wellspring and identify unique needs. A program stream was created including a professionally led bi-weekly virtual support group, specialized peer support, and coaching. Volunteer training modules were also created to promote awareness of the unique needs of this population. Metrics collected included the number of unique participants and attendance rates. An electronic survey was distributed to participants (n = 85) from January 2023 to November 2024 to assess satisfaction and program impact.

### 53.3. Impact on Practice or Results

The new programs incorporated discussion related to adapting to fluctuating symptoms and emotional challenges, navigating changes in relationships, and coping with grief and loss. Since its launch (February 2021), 221 patients have participated, for a total of 2328 visits. Survey respondents (response rate = 51.8%) reported feeling understood (98%), less alone (93%), more confident (89%), less anxious or overwhelmed (91% and 86% respectively). Additionally, 92% indicated they felt more positive as a result of participating.

### 53.4. Discussion or Conclusions

The new program stream was found to address a critical gap in care for individuals living with incurable cancer, offering a supportive space tailored to their unique needs over a prolonged period. By meeting patients where they are, Wellspring is fostering connection, resilience and hope within its community.

**169**

## 54. Expanding Community-Based Pancreatic Cancer Support Through Partnership

Susy Santos, Gloria Ghabeli

Wellspring Cancer Support, Toronto, Canada.

### 54.1. Background/Rationale or Objectives/Purpose

Pancreatic cancer is a rare aggressive disease; most diagnoses occur at advanced stages when patients are in crisis. Recognizing the need for psychosocial support, Pancreatic Cancer North America (PCNA) and Wellspring Cancer Support (Wellspring) entered a partnership to provide specialized programs and raise awareness of available services. Wellspring designed and piloted new supportive care programs, PCNA contributed disease expertise, funding and promotional support.

### 54.2. Methodology or Methods

The following specialized programs were launched between 2018–2024: Peer Support, specialized Volunteer Training, Counselling, Support Groups, Educational Presentations/Videos and Bereavement Support (MAID). Program impact was assessed through the following metrics: new memberships, attendance in pancreatic and general programs, video views and volunteers recruited and trained. Annual surveys measured program quality, impact and participant satisfaction.

### 54.3. Impact on Practice or Results

The annual number of individuals reaching out to Wellspring for support increased by 83% (58% pancreatic patients, 42% caregivers). 31 volunteers facilitated 1160 peer support appointments, and 1009 professional counselling appointments were completed. Participants reported feeling heard and understood (98%), less alone (98%) and less anxious (91%). Pancreatic patients also accessed other support like Relaxation and Visualization, Exercise, and Yoga.

### 54.4. Discussion or Conclusions

The partnership effectively expands care for individuals with pancreatic cancer, allowing the two organizations to focus on their strengths. Strong collaboration built on trust, resource sharing, and a shared vision has proven instrumental. Further expansion of the partnership will include an early-onset support group and resources for children. The model’s success has inspired replication with other national cancer organizations.

**203**

## 55. Evaluating the Feasibility and Acceptability of a Community-Based, Co-Created Yoga Program for Women with Gynecologic Cancer: A Series N-of-1 Feasibility Study

Jenson Price ^1^, Brooklyn Westlake ^1^, Jennifer Brunet ^2,3,4^

1. School of Human Kinetics, University of Ottawa, Ottawa, Canada.
2. School of Human Kinetics, University of Ottawa, Ottawa, Canada.
3. Cancer Therapeutic Program, Ottawa Hospital Research Institute, The Ottawa Hospital, Ottawa, Canada.
4. Institut du savoir Montfort, Hôpital Montfort, Ottawa, Canada.

### 55.1. Background/Rationale or Objectives/Purpose

Current yoga programs for cancer survivors do not meet participants’ needs and are rarely implemented in community-based settings, despite reported benefits. The aim of the current study was to implement a co-created 12-week bi-modal Hatha-based yoga program for adults diagnosed with gynecologic cancer in the community and assess the feasibility and acceptability of the program and study methods.

### 55.2. Methodology or Methods

Using a mixed methods series N-of-1 A_1_BA_2_ research design, participants were recruited from The Ottawa Hospital. Participants self-selected a morning or evening program, completed surveys 9 to 11 times and were interviewed post-program. The yoga instructor was interviewed post-program about their experience delivering the program. Quantitative feasibility outcomes were tracked throughout the study. Qualitative acceptability outcomes were explored during post-program semi-structured interviews. Audio- and video-recordings of the yoga classes and data from the instructor interview were used to assess fidelity outcomes to determine whether the protocol could be adhered to consistently.

### 55.3. Impact on Practice or Results

Forty-one individuals were screened for eligibility and 20 consented (48.7%). Seventeen participants (85.0%) completed the final survey. Participants attended 83.1% (19.9/24) of classes with varied engagement with optional features. The instructor was 61.3% adherent to the prescribed protocol, using recommended behaviours 44.6% of the time. Participants shared barriers and facilitators that influenced the success of the trial methods and program.

### 55.4. Discussion or Conclusions

The program was well-received and trial methods were moderately successful but refinements are warranted before a large-scale trial. Community-based yoga programs could be feasible and acceptable for women with gynecologic cancer.

## 56. Final Category: D. Complementary and Integrative Cancer Care

**29**

## 57. Impact of Yoga Therapy on Autonomous Nervous System Functioning and Heart Rate Variability Among Cancer Survivors: A Single-Subject Exploratory Experimental Study

Jennifer Brunet ^1^, Julia Hussien ^1^, Anne Pitman ^2^, Christophe Henry ^3^, Nadia Polskaia ^4^, Amanda Wurz ^5^, Ellen Conte ^6^, Andrew Seely ^3,7^, Dugald Seely ^6,2^

1. University of Ottawa, Ottawa, Canada.
2. The Centre for Health Innovation, Ottawa, Canada.
3. The Ottawa Hospital Research Institute, Ottawa, Canada.
4. Bruyère Health Research Institute, Ottawa, Canada.
5. University of the Fraser Valley, Chilliwack, Canada.
6. The Patterson Institute for Integrative Oncology Research, Ottawa, Canada.
7. The Ottawa Hospital, Ottawa, Canada.

### 57.1. Background/Rationale or Objectives/Purpose

We previously showed a yoga therapy (YT) intervention can improve specific patient-reported outcomes (PROs) in adults after cancer treatment. The mechanisms through which YT improves PROs are unclear. In this single-subject exploratory experimental study, we evaluated the effects of the YT intervention on autonomic nervous system functioning (ANS) and heart rate variability outcomes in adults after cancer treatment.

### 57.2. Methodology or Methods

Data from 20 adults (*M*_age_ = 55.7, 85% female; *M*_years from diagnosis_ = 2.8) recruited from *The Centre for Health Innovation* were analyzed. Participants received one 1:1 YT session followed by 6 weekly small group YT sessions. Outcomes were assessed before, during, and after the intervention via self-report (trait ANS, state ANS) and Hexoskin Smart Shirts (HRV outcomes [mean heart rate, standard deviation, coefficient-of-variation, RMSSD, sample entropy, Low Frequency power, High Frequency power]). Hierarchical linear models were tested with fixed effects to assess average baseline level (*Intercept*), linear slope (*Time*), YT initiation effect on level (*Phase*), and YT intervention effect on linear slope (*Time-by-Phase*).

### 57.3. Impact on Practice or Results

Significant (*p* < 0.05) small *Time*, *Phase*, and *Time-by-Phase* effects were found for state ANS, coefficient-of-variation and High Frequency power. A significant (*p* < 0.05) small *Time-by-Phase* effect was found trait ANS, notably with a greater rate of change after YT initiation. Significant (*p* < 0.05) small *Time* and *Phase* effects were found for sample entropy. No other effects were significant.

### 57.4. Discussion or Conclusions

Results require confirmation but support continued investigation of YT to gain insight into the practical significance that can be attributed to HRV changes after cancer treatment.

**51**

## 58. Embedding the Patient Voice in the Research Process: Collaborative Qualitative Analysis of Cancer Interviews

Yasmin Lalani, Danielle Cameron

Humber River Health, Toronto, Canada.

### 58.1. Background/Rationale or Objectives/Purpose

Without clearly defined roles, patient involvement in research may sometimes lack meaningful engagement. This presentation demonstrates how involving a patient partner in qualitative analysis enriches interpretation, providing insights grounded in lived experience. We outline our collaborative analysis process of interviews with cancer patients, highlighting the value of including the patient voice at this stage.

### 58.2. Methodology or Methods

As part of a larger project to develop a digital meditation program for cancer patients, 15 participants were recruited from Humber River Health’s cancer clinic for interviews. Interviews explored stress-coping strategies, perceptions of meditation, and preferences for meditation content. Using Interpretive Phenomenological Analysis (IPA), themes were developed from individual and group-level interpretations. The process extended IPA’s “double hermeneutics” by incorporating a patient partner’s lived experience, offering unique interpretations. Regular meetings allowed us to explore findings collaboratively, adding an enriched layer of insight that might otherwise have been overlooked

### 58.3. Impact on Practice or Results

Themes revealed that participants felt a drive to “stay positive” as a way to counteract fear and uncertainty about their future. Many were open to using meditation to cultivate calm, with some already practicing meditation. A shared theme was participants’ determination to maintain control in their lives. We anticipate that a tailored digital meditation program would benefit cancer patients who see themselves as having the mental and emotional strength to find moments of calm and control.

### 58.4. Discussion or Conclusions

Involving a patient partner at the analysis stage deepened understanding of patient narratives, with IPA serving as an effective framework for this collaborative approach.

**58**

## 59. Development of Psychoeducational Interventions for Early-Age-Onset Colorectal Cancer (EAO CRC) Patients, Caregivers and Healthcare Providers Using the Social Cognitive Theory (SCT) Approach

Camelia Tavakoli ^1^, Chelsea Butler ^2^, Laurence Gauthier Desrochers ^2^, Sandra Pelaez ^2^

1. Colorectal Cancer Canada, Maple Ridge, Canada.
2. Colorectal Cancer Canada, Montreal, Canada

### 59.1. Background/Rationale or Objectives/Purpose

Early-age-onset colorectal cancer (EAO CRC) patients and caregivers face unique psychosocial challenges requiring tailored interventions.

### 59.2. Methodology or Methods

A comprehensive needs assessment identified gaps in EAO CRC psychosocial care using Colorectal Cancer Canada resources and available literature. A psychoeducational intervention based on the social cognitive theory (SCT) is developed. The intervention equips patients and caregivers with self-management tools to cope with psychosocial issues such as stress and anxiety. The intervention is made of interactive exercises and activities that use observational learning as a starting point to enhance self-management through personal agency and self-efficacy.

### 59.3. Impact on Practice or Results

THIS intervention seeks to empower patients and caregivers by providing actionable tools for self-managing psychosocial challenges. While tailored to address the unique needs of EAO CRC patients and caregivers, the principles and strategies outlined in these interventions can be adapted for individuals affected by any cancer type. By promoting healthcare provider awareness of psychosocial challenges across diverse cancer populations, this intervention fosters integrated care and enhances the quality of life for patients and their caregivers, regardless of the specific diagnosis.

### 59.4. Discussion or Conclusions

Psychoeducational interventions that enhance self-management by building personal agency and self-efficacy are essential for addressing diverse patient needs and integrating psychosocial care into standard practice. However, implementing these interventions requires overcoming challenges like healthcare provider engagement barriers and resource accessibility. Future efforts should focus on evaluating interventions’ long-term effectiveness, expanding digital platforms for broader reach, and refining resources through stakeholder feedback to enhance support for patients, caregivers, and healthcare providers.

**89**

## 60. A Survey of Patients’ Insomnia Treatment Preferences Across the Cancer Care Continuum

Nicole Carmona ^1,2^, Lucas Norton ^1^, Karen Fergus ^1,2^

1. York University, Toronto, Canada.
2. Odette Cancer Centre, Toronto, Canada.

### 60.1. Background/Rationale or Objectives/Purpose

Insomnia is a significant problem across the cancer care continuum and is effectively treated with cognitive behavioural therapy for insomnia (CBT-I). Studies have identified barriers that limit access to CBT-I in oncology settings, but have yet to assess patient preferences regarding insomnia treatment, such as *when is the best time to intervene, how, and in what format?* Our objective is to answer these questions using a virtual survey to inform the design of patient-centred care pathways that focus on getting the right treatment into the right hands, at the right time.

### 60.2. Methodology or Methods

Participants with cancer (N = 80), ranging from diagnosis to survivorship, will be recruited from the Sunnybrook Odette Cancer Centre. Consenting participants will respond to a survey describing insomnia treatment options and asked to rate these in terms of level of interest at various points in the cancer trajectory and preferred format (e.g., info sheet, self-help, online or face-to-face—individual or group).

### 60.3. Impact on Practice or Results

We anticipate an interest in effective, nonpharmacological insomnia treatments such as CBT-I, in formats that do not increase appointment burden, especially during active treatment. Participants will have the opportunity to identify and explain their reasoning behind their preferred treatment formats.

### 60.4. Discussion or Conclusions

By engaging patient stakeholders, the results of this study will inform the creation of a suite of patient-centred insomnia resources and care pathways that strive to match patients to interventions based on needs and preferences, as well as consideration of *when* they are most likely to benefit.

**115**

## 61. Feasibility and Acceptability of a Creative Art Workshop as an Innovative and Complementary Approach to Conventional Treatment of Psychiatric Comorbidities in Oncology: Preliminary Results of a Single-Center Study

Jacynthe Rivest ^1^, Julie Haslam ^1^, Francine Aubin ^1^, Christophe Longpré-Poirier ^2^, Véronique Desbeaumes-Jodoin ^1^, Joé T. Martineau ^3^

1. University of Montreal Hospital Center, Montreal, Canada.
2. Institut de Cardiologie de Montreal, Montreal, Canada.
3. Hautes Etudes Commerciales de Montréal, Montreal, Canada.

### 61.1. Background/Rationale or Objectives/Purpose

People with cancer are at greater risks for affective and anxiety disorders. Art-based interventions have been found to be beneficial as a complementary approach to conventional treatments in mental health and cancer care. Recent advances in virtual care have improved accessibility to specialized services. However, no studies have examined the use of a virtual creative arts workshop for people with both cancer and psychiatric disorders. This pilot study aims to determine the feasibility and acceptability of such an intervention for this comorbid population.

### 61.2. Methodology or Methods

Sample and setting: This is a single-center pilot study with no control group. Participants will be adults receiving care at an academic consultation-liaison psychiatry service. The convenience sample size is estimated at 30 participants.

Procedures: Participants will engage in an 8-week virtual creative arts workshop facilitated by a professional artist. Data will be collected before, immediately after, and four weeks post-workshop using attendance logs, a visual analog scale of satisfaction, surveys and questionnaires on psychological symptoms, well-being and quality of life. Descriptive statistical analysis will be carried out.

### 61.3. Impact on Practice or Results

Feasibility will be demonstrated if participants complete at least half of the sessions, while acceptability will be assessed based on satisfaction rates and the highlighting of facilitators that might influence implementation.

### 61.4. Discussion or Conclusions

This pilot study could inform about the feasibility and acceptability of such intervention before considering a future randomized controlled trial. These findings could also help clinicians to better understand the barriers and facilitators in implementing complementary approaches in psychosocial oncology.

**162**

## 62. Cancer and the Arts: Restoring the Soul of Medicine for Patients, Caregivers, and Staff Through Art

Miah Zammit ^1^, Meena Merali ^1^, Patricia Ritacca ^1^, Malka Greene ^2^, Hanae Davis ^1^, Gary Rodin ^1^

1. Princess Margaret Cancer Centre, Toronto, Canada.
2. Princess Margaret Cancer Foundation, Toronto, Canada.

### 62.1. Background/Rationale or Objectives/Purpose

The Cancer and the Arts Program at the Princess Margaret aims to enhance the cultural safety and healing environment of the cancer centre through curated visual art and musical experiences. Prior to the launch of this program, the visual art and musical offerings at the hospital were not under a central governance structure that put IDEAA (inclusion, diversity, equity, access, and anti-racism) at the forefront. Upon establishing a modern curatorial process and key partnerships, the main areas of the hospital have been revitalized to resonate with the diverse community that we serve. The visual arts stream features site-specific installations of contemporary artworks from local, regional, and national artists. The musical stream hosts performances for patients, staff, and families, with diverse local musicians.

### 62.2. Methodology or Methods

This poster presentation will showcase the curatorial process, community partnerships, and clinic engagement involved. The Cancer and the Arts Committee provides curatorial oversight, a collaborative effort comprising staff, patient partners, arts professionals, and volunteers. Patient feedback is being collected through a survey-based evaluation, and preliminary results demonstrate positive experiences with these offerings.

### 62.3. Impact on Practice or Results

By integrating art and music, Cancer and the Arts promotes a sensory-rich environment that nurtures well-being while cultivating a more empathic, equitable, and supportive atmosphere for everyone at the Princess Margaret.

### 62.4. Discussion or Conclusions

Cancer and the Arts will continue to promote psychosocial well-being through culturally diverse artwork and music at the Princess Margaret. New partnerships and continued stewardship that will ensure that the arts remain a core feature of the ambience at the cancer centre.

## 63. Final Category: E. Exercise/Pre-Habilitation and Rehabilitation in Cancer

**12**

## 64. Work in Progress: Development of a Prehabilitative Yoga Intervention (“ACE-Stem Yoga”) for Individuals with Acute Leukemia Undergoing an Allogeneic Stem Cell Transplant

Jocelyn L. Cannon ^1^, Julia T. Daun ^1^, Meghan H. McDonough ^1^, Sarah Perry ^2^, S. Nicole Culos-Reed ^1,3^

1. Faculty of Kinesiology, University of Calgary, Calgary, Canada.
2. Division of Hematology and Hematologic Malignancies, Calgary, Canada.
3. Department of Psychosocial Resources, Tom Baker Cancer Centre, Alberta Health Services, Calgary, Canada.

### 64.1. Background/Rationale or Objectives/Purpose

The Alberta Cancer-Exercise (ACE)-Stem Yoga study is tailoring and assessing the feasibility of a prehabilitation yoga intervention for individuals preparing for an allogeneic stem cell transplant (alloHSCT) to support patients’ physical and psychosocial well-being prior to alloHSCT.

### 64.2. Methodology or Methods

Sample and setting: A convenience sample of individuals 18+ with acute leukemia eligible for an alloHSCT in Calgary, AB are drawn from the larger trial.

Procedures: Phase 1 involved a literature review identifying psychosocial and functional challenges for patients awaiting alloHSCT. In Phase 2, a multidisciplinary team—including Clinical Exercise Physiologists, a yoga therapist, and clinicians—developed the intervention to address gaps in literature to date. Phase 3 (ongoing) is a pilot feasibility trial (n = 5–8 participants) of a brief yoga intervention, assessing intervention adherence, fidelity, participant satisfaction, and engagement. Exploratory examination of patient-reported outcomes pre-/post-intervention includes cancer-related fatigue (CRF), exercise self-efficacy, and health-related quality of life (HRQL). Post-intervention interviews will capture feedback on barriers and facilitators to engagement.

### 64.3. Impact on Practice or Results

Results: The development process resulted in a 4–6-week gentle yoga protocol, with 15–20-min classes offered twice weekly. One class per week is live, and one is pre-recorded, each focusing on weekly themes that incorporate movement, breath, and relaxation techniques. Team insights ensured the program minimizes risk and addresses common treatment side-effects. Data will be analyzed descriptively to assess feasibility and inform future trials, and qualitative data obtained from interviews will be analyzed using content analysis.

### 64.4. Discussion or Conclusions

Conclusions and Clinical Implications: Prehabilitative yoga may enhance HRQL for patient’s pre-transplant. This protocol contributes to oncology-specific yoga intervention development, evaluation, and implementation in clinical oncology settings.

**32**

## 65. Facilitating Access to Cancer Rehabilitation—Refining the Cancer Rehabilitation and Exercise Screening Tool (CREST)

Lauren Capozzi, S. Nicole Culos-Reed, Julia Daun, Hannah Cripps, David Langelier, Khara Sauro

University of Calgary, Calgary, Canada.

### 65.1. Background/Rationale or Objectives/Purpose

Despite over 65% of patients reporting functional impairments related to their cancer or associated treatments, screening of physical function and referral to appropriate rehabilitation services is often inadequate. Therefore, the Cancer Rehabilitation and Exercise Screening Tool (CREST) was proposed to identify impairment and inactivity through a 22-item patient reported questionnaire, tailored for use in an outpatient oncology setting. This in-progress study aims to refine the CREST to be used to facilitate screening for physical impairment and inactivity among those living with and beyond cancer.

### 65.2. Methodology or Methods

The CREST will be refined by a group of national knowledge users (KUs, n = 10–14), including physiatrists, oncology providers, rehabilitation providers, patient partners, and oncology researchers. Using a modified Delphi framework, the KUs will participate in a series of surveys and focus groups, whereby consensus (70% agreement) will be reached on the final version of CREST. Consensus on clarity, quality, and importance of each item will be assessed.

### 65.3. Impact on Practice or Results

Anticipated Results: A final version of the CREST will be developed and ready for validation and implementation.

### 65.4. Discussion or Conclusions

This project has the potential to better support patients, healthcare providers, and systems in reducing the burden of functional impairment and connecting patients with resources. Following refinement of the tool, Phase 2 will include a validation study, and Phase 3 will assess facilitators and barriers to implementation.

Questions for Consideration:

1. What are barriers and facilitators to a physical function screening tool in your clinic setting?
2. How would the implementation of functional screening impact your practice?

**50**

## 66. “If I’m Feeling Good Physically, That’s an Impact on How I’m Feeling Emotionally”: A Qualitative Analysis of Mental Health in Women with and Beyond Breast Cancer After an Exercise Program

Natalie Cuda, Kelly Arbour-Nicitopoulos, Catherine Sabiston, Michelle Ha, Hui Xiao, Linda Trinh

University of Toronto, Toronto, Canada.

### 66.1. Background/Rationale or Objectives/Purpose

The purpose of this qualitative analysis was to understand the impact of a breast cancer diagnosis on the mental health of women with and beyond breast cancer (WBBC) post-chemotherapy treatment following participation in a remotely-delivered exercise program.

### 66.2. Methodology or Methods

Women WBBC were recruited between February and July 2023 across Canada to participate in a remotely delivered, supervised exercise intervention. Women WBBC were randomized 1:1 into a combined exercise (i.e., aerobic + resistance; n = 10) or active control (i.e., balance + flexibility; n = 11) program. Semi-structured, 1-on-1 interviews were conducted at post-intervention (i.e., 8-weeks). Women WBBC were asked about the influence of their breast cancer diagnosis and treatment on their mental health, and how exercise impacted their mental health. Interviews were transcribed verbatim and analyzed using Braun & Clarkes (2006) six phases of thematic analysis. The study employed an inductive, data-driven approach inspired by Grounded Theory principles.

### 66.3. Impact on Practice or Results

Women WBBC (N = 21; M_age_ = 51.6 ± 7.2 years) were 22.9 ± 13.8 months since diagnosis and 11.8 ± 12.9 months since treatment. Four main themes emerged: (1) Identity disruption: Navigating self-perception post-breast cancer; (2) Living with uncertainty in the breast cancer journey; (3) The physical impact of breast cancer and chemotherapy, and (4) Exercise as a source of empowerment. These results demonstrate the relationship between mental health, identity, and perceived capability among women WBBC post-breast cancer treatment, and the positive influence of exercise on mental health.

### 66.4. Discussion or Conclusions

This study demonstrates the benefit of incorporating supportive care strategies such as exercise to improve mental health among women WBBC.

**74**

## 67. Factors Influencing Healthcare Provider Referrals to a Physical Activity Intervention and Trial

Hannah Cripps ^1^, Emma McLaughlin ^1^, Laura St. John ^1^, Fiona Schulte ^2,3^, Amanda Wurz ^4^, Nicole Culos-Reed ^1,3,5^

1. Faculty of Kinesiology, The University of Calgary, Calgary, AB, Canada.
2. Department of Oncology, Division of Psychosocial Oncology, Cumming School of Medicine, Calgary, AB, Canada.
3. Department of Psychosocial Resources, AE Child Comprehensive Cancer Centre, Calgary, AB, Canada.
4. School of Kinesiology, University of the Fraser Valley, Chilliwack, BC, Canada.
5. Department of Oncology, Cumming School of Medicine, University of Calgary, Calgary, AB, Canada.

### 67.1. Background/Rationale or Objectives/Purpose

Physical activity (PA) is beneficial for children and adolescents on treatment for cancer. To support PA, the IMPACT intervention and trial were developed. Recruitment for IMPACT occurs primarily through healthcare provider (HCP) referral (n = 84/93; 90%). To date, only 17 (18%) children and adolescents have consented and enrolled, which is lower than expected. To enhance HCP referrals and support processes to enhance recruitment and enrollment, the purpose of this study was to understand pediatric oncology HCPs’ barriers and facilitators for referral to IMPACT and explore strategies to facilitate more effective consenting processes.

### 67.2. Methodology or Methods

HCPs were recruited via email and invited to complete a demographics survey and 1:1 semi-structured interview, following the capability, opportunity, motivation-behavior (COM-B) model. Interviews were audio recorded, transcribed verbatim, and analyzed using conventional content analysis.

### 67.3. Impact on Practice or Results

16 HCPs from two children’s hospitals in Alberta completed the survey, and 15 HCPs completed the interview (Zoom n = 13, phone n = 1, in-person n = 1). Interviews ranged from 15.43–28.24 min. HCPs’ experiences with referral were captured across 4 main themes: (1) Benefits and Beliefs about PA; (2) Families are in Survival Mode; (3) Healthcare Provider Role Discussing PA; (4) Pathways to Participation-Referral and Consent to IMPACT.

### 67.4. Discussion or Conclusions

Findings suggest that HCPs believe PA is beneficial for children and adolescents on treatment, however, PA referral and discussions are not always a priority. HCPs have competing demands and shared that families are often overwhelmed. Understanding HCPs’ experience with referral and unique factors in this clinical setting will inform ongoing efforts to enhance recruitment and enrollment within IMPACT.

**105**

## 68. Understanding Participant Dropouts in the Exercise for Cancer to Enhance Living Well (EXCEL) Study to Support Exercise as Supportive Cancer Care

Jonathan Low ^1^, Julianna Dreger ^1^, Chad Wagoner ^2^, Emma McLaughlin ^1^, Jocelyn Cannon ^1^, Margaret McNeely ^3^, Melanie Keats ^4^, Daniel Santa Mina ^5^, Linda Trinh ^5^, Kristin Campbell ^6^, Isabelle Doré ^7^, Colleen Cuthbert ^8^, Lauren Capozzi ^9^, Daniel Sibley ^5^, Thomas Christensen ^4^, Alexia Piché ^7^, Kelly Mackenzie ^6^, Sean Sawer ^6^, Nicole Culos-Reed ^1^

1. Faculty of Kinesiology, University of Calgary, Calgary, Canada.
2. Department of Kinesiology, Recreation, and Sport Studies, University of Tennessee—Knoxville, Knoxville, USA.
3. Department of Physical Therapy, University of Alberta, Edmonton, Canada.
4. Faculty of Health, School of Health and Human Performance, Dalhousie University, Halifax, Canada.
5. Faculty of Kinesiology and Physical Education, University of Toronto, Toronto, Canada.
6. Department of Physical Therapy, Faculty of Medicine, University of British Columbia, Vancouver, Canada.
7. School of Kinesiology and Physical Activity Sciences, Faculté de médecine, Université de Montréal, Montreal, Canada.
8. Faculty of Nursing, University of Calgary, Calgary, Canada.
9. Cumming School of Medicine, University of Calgary, Calgary, Canada.

### 68.1. Background/Rationale or Objectives/Purpose

Exercise studies provide valuable insights into the physical and psychosocial benefits for people with cancer, yet adherence to community-based exercise programs can be variable. Understanding ‘dropout’ reasons and timing that dropouts occur can provide insight that study-designs can address to promote better adherence.

### 68.2. Methodology or Methods

We explored dropout timing and reasons that occurred after consent to the EXercise for Cancer to Enhance Living Well (EXCEL) study, a 12-week exercise trial delivered online and in-person to Canadians living with and beyond cancer in rural/remote/underserved communities. Dropout numbers, when dropouts occurred, and reasons for dropouts were tabulated across program delivery from 2020–2024.

### 68.3. Impact on Practice or Results

Of the 1002 participants, 19% (n = 278) have dropped out, with 28% (n = 77) occurring in the first 2 weeks between consent and EXCEL start. Most dropouts (72%, n = 201) occurred within 4.8 (SD ± 4.7) weeks after starting EXCEL. Reasons for dropout included medical (treatment reasons, recurrence, death; n = 83, 30%), lack of interest or motivation (n = 154, 55%), scheduling/time constraints (n = 23, 8%), administrative/logistical issues (n = 13, 5%), and ‘other’ reasons (n = 5, 2%).

### 68.4. Discussion or Conclusions

Participants in exercise oncology studies often cite lack of interest and treatment-related issues when dropping out. The gap from consent to study start, as well as addressing barriers during study delivery, may require greater exercise intervention flexibility to meet participants’ needs. Future work will explore dropout reasons in relationship to participant health and demographics, as well as delivery options to address needs.

**121**

## 69. Trajectories of Physical Activity After Curative Cancer Surgery and Their Association with Fear of Cancer Recurrence

Juliette Picard ^1,2,3^, Claudia Mc Brearty ^1,2,3^, Josée Savard ^1,2,3^

1. School of Psychology, Université Laval, Québec, Canada.
2. CHU de Québec-Université Laval Research Center, Québec, Canada.
3. Université Laval Cancer Research Center, Québec, Canada.

### 69.1. Background/Rationale or Objectives/Purpose

Fear of cancer recurrence (FCR) is frequent in cancer survivors, affecting their quality of life. Individuals experiencing FCR often adopt control-seeking behaviours to reduce the perceived risk of recurrence. Given the benefits of physical activity (PA) in decreasing the likelihood of cancer recurrence, this study aimed to identify trajectories of self-reported PA over 18 months following surgery for nonmetastatic cancer and evaluate their association with FCR.

### 69.2. Methodology or Methods

962 patients with mixed cancer types scheduled for curative surgery were recruited. Self-reported PA (number of episodes > 20 min/month) and FCR (Fear of Cancer Recurrence Inventory-Severity) were assessed at six time points: perioperative phase (T1), 2 (T2), 6 (T3), 10 (T4), 14 (T5), and 18 (T6) months post-surgery. Group-based trajectory modelling (GBTM) was used to identify distinct PA trajectories, including FCR as a time-varying variable. Trajectory groups were categorized by perioperative PA levels (high, medium, low) and patterns of change (increaser, decreaser, maintainer).

### 69.3. Impact on Practice or Results

Analyses included 879 patients who provided PA data at ≥2 time points. GBTM identified four trajectories of PA: low maintainer (22.2%), low increaser (41.5%), medium increaser (29.4%) and high maintainer (6.9%). A reduction in PA was associated with increased FCR severity in the high maintainer group (β = −3.196, *p* = 0.014), but not in the other trajectory groups.

### 69.4. Discussion or Conclusions

Four distinct PA trajectories were identified among nonmetastatic cancer patients over 18 months post-surgery. The association between FCR and PA was significant in the high maintainer group, indicating that reduced PA levels may increase FCR severity in this subgroup of patients.

**179**

## 70. Inclusion of Exercise as a New Standard of Supportive Prostate Cancer Care: A Transdisciplinary Prehabilitation Program

Julia T. Daun ^1^, Jamie Filatoff ^2^, Jessica Danyluk ^1^, Arjuni Seevaratnam ^2^, Taylor Travis ^2^, Zoë Keefe ^2^, Lin Yang ^3,4^, John Dushinski ^2^, Viktoriia Shcherbachuk ^2^, S. Nicole Culos-Reed ^1,3,5^

1. Faculty of Kinesiology, University of Calgary, Calgary, Canada.
2. Prostate Cancer Centre, Calgary, Canada.
3. Department of Oncology, Cumming School of Medicine, University of Calgary, Calgary, Canada.
4. Department of Community Health Sciences, University of Calgary, Calgary, Canada.
5. Department of Psychosocial Resources, Tom Baker Cancer Centre, Alberta Health Services, Calgary, Canada.

### 70.1. Background/Rationale or Objectives/Purpose

Prehabilitation (i.e., Prehab) is an evidence-based intervention for supporting preparation for and recovery from radical prostatectomy. Given the potential to optimize patient outcomes, the Calgary Prostate Cancer Centre (PCC) developed and is implementing the PCC Prehab program.

### 70.2. Methodology or Methods

Patients’ pre-radical prostatectomy are referred by urologists to the Prehab program via the electronic medical record. Patients complete baseline patient-reported outcomes, attend a medical assessment, receive access to a patient portal for wellness resources, and are triaged based on their preferences to one or a combination of Prehab pathways including exercise, psychosocial and sexual health, pelvic floor, and nutrition. Exercise participants complete a baseline functional fitness assessment, receive up to three one-on-one exercise and health coaching sessions, and are provided a tailored exercise program and/or twice weekly group exercise sessions. Programming is available in-person or online, and participants attend up to 12 weeks pre surgery. Post-program assessment of patient-reported outcomes and functional fitness occurs approximately one week prior to surgery.

### 70.3. Impact on Practice or Results

The Prehab program has enrolled 100 patients over nine months and triaged 53 patients to exercise (mean patient age: 63 years). Average time until surgery has been 6.59 weeks (range 0.4–18 weeks) and patients have most commonly been diagnosed with intermediate risk (i.e., localized) prostate cancer. Interim analyses examining implementation and effectiveness metrics will occur in March 2025 at the one-year mark.

### 70.4. Discussion or Conclusions

With an integrated clinical approach, the transdisciplinary PCC Prehab program is designed to optimize patient wellness prior to surgery. Future work will examine potential impact on surgical outcomes and will evaluate cost-effectiveness.

**194**

## 71. Enhancing Psychological Health in Prostate Cancer Patients: The Mediating Roles of Self-Efficacy and Illness Perceptions Through the Prostate Cancer Patient Empowerment Program

Cody MacDonald ^1,2^, Gabriela Ilie ^1,2^, George Kephart ^1^, Ricardo Rendon ^1,2^, Ross Mason ^1,2^, Greg Bailly ^1,2^, David Bell ^1,2^, Nikhilesh Patil ^1,2^, David Bowes ^1,2^, Derek Wilke ^1,2^, Andrea Kokorovic ^1,2^, Rob Rutledge ^1,2^

1. Dalhousie University, Halifax, Canada.
2. Nova Scotia Health, Halifax, Canada.

### 71.1. Background/Rationale or Objectives/Purpose

Empowering prostate cancer patients through structured self-management interventions can improve mental health, yet the mechanisms driving these improvements remain underexplored. This secondary analysis of the Prostate Cancer Patient Empowerment Program (PC-PEP) randomized trial examined the intervention’s effects on self-efficacy and illness perceptions, exploring their role in helping patients cope with their diagnosis and mediating reductions in psychological distress.

### 71.2. Methodology or Methods

A total of 128 patients with localized prostate cancer scheduled for curative-intent treatment were randomly assigned to PC-PEP or standard care (waitlist control). Over six months, PC-PEP participants received daily emails, weekly adherence surveys, and monthly live sessions promoting healthy habits related to physical activity, nutrition, relaxation, pelvic floor muscle training, and strategies to enhance relationships and intimacy. Questionnaires measuring psychological distress, self-efficacy, and illness perceptions were administered at baseline and six months. Linear mixed models and mediation analysis (PROCESS macro) evaluated the intervention’s direct effects and mediating pathways.

### 71.3. Impact on Practice or Results

PC-PEP significantly improved self-efficacy (*p* = 0.023) and illness perceptions, including personal control (*p* = 0.032) and emotional impact (*p* = 0.026), compared to standard care. These improvements mediated the intervention’s effects on psychological distress, with self-efficacy as the strongest mediator, accounting for 52% of the effect. Mediation effects were consistent irrespective of treatment (surgery or radiotherapy).

### 71.4. Discussion or Conclusions

PC-PEP fosters empowerment and activation through a structured, patient-centered approach, equipping patients with confidence, motivation, and a sense of control to enhance their mental health. Integrating PC-PEP into clinical practice offers an effective model to improve well-being and address the psychosocial burden faced by this population.

## 72. Final Category: F. Equity, Diversity and Inclusion (Sociodemographic, Culture, and Sex/Gender Issues)

**2**

## 73. It Is More than Just a Tattoo—Restorative Areola Tattooing

Alexandra Ginty ^1,2,3^

1. Halton Healthcare, Oakville, Canada.
2. University of Toronto, Toronto, Canada.
3. McMaster University, Hamilton, Canada.

### 73.1. Background/Rationale or Objectives/Purpose

The highest complaints in Survivorship are fear of recurrence, often manifesting in depression (23%) and anxiety (30%), followed by sexuality and body image changes (85%). Closure of the cancer journey with reconstruction has high quality of life results. The additional role of restorative tattooing distracts from scars, deformities and mismatch further increasing satisfaction scores dramatically. Typically, good quality 3D tattooing costs $1000–$2500. Most patients after loss of work during treatment and low SES cannot complete this. This gap in equity was identified by Dr Ginty during breast reconstruction as a patient and physician in leadership.

Trained over 200 h by world tattooists, Dr Ginty passionately opened the Restore-me clinic, supported by Halton Healthcare with no cost to the hospital or the patient. It was designed on a referral system available on a self-developed website and a secure fax from their physician. Once the referral is received, patients are invited to self-book using tags by procedure. 60% of patient’s self-book and even higher for their first visit. This allows patients to access booking or moving their appointment around treatments after office hours and at their convenience. It innovatively also reduces overhead. Reminders from the system address the cognitive fatigue experienced after treatment and has resulted in 0 no-shows which is important for the 1–2 h procedures. The OHIP code is now flagged as a non-negatable code for primary care referrals. Consultations are dictated on the hospital line to align the medical team. Inks are available in all Fitzpatrick skin types. Patients of black, middle and south Asian skin types are included. The realistic art of creating a 3D nipple-areola tattoo as well as understand the psychology and surgical procedures undertaken is part of closure for all patients and universal access in the safety of the hospital should be offered everywhere.

We aim to train physicians from all areas in this program that does not rely on hospital funding and improve the quality of life for all patients regardless of color or economic status.

### 73.2. Methodology or Methods

With the engagement of physicians from London to Toronto, patients who have finished breast reconstruction are now routinely offered OHIP-covered restorative areola tattooing with 50% now outside our Halton region. This is a change of 100% in just 12 months. Most patients agree that they would not consider going outside the hospital and could not afford to pay.

OMA announcements and patient-driven requests (50%) are a powerful reminder of the advocacy of patient awareness and education.

The restore-me clinic presence on media such as CHCH news, City TV news, Local news station and 30 min interview on local TV as well as special guest interviews have helped patients, friends or family become aware of this unique and equitable program. Talks at Wellspring, one of three booths at Breast reconstruction day Toronto, the biggest event of the year have been patient-engaging.

Totalling 280 new referrals, this program is where art and medicine meet to psychologically make a difference. As restorative 3D areola tattooing has historically been in the community, there is little data to compare. We now have the opportunity to study this in our Regional Cancer Centres.

### 73.3. Impact on Practice or Results

The quality-of-life impact of restorative areola tattooing is measured in an overwhelming, emotional patient response. This is a completely OHIP-covered clinic by a physician cancer-survivor and artist who takes the time to 3D tattoo realism over the scars of mastectomies and breast reconstruction. Such high-quality tattoos are $1000–2500—inaccessible for most after financial burdens of cancer treatments.

This program innovatively uses self-booking from physician referrals with no nurse and reduces overhead using a hospital clinic based in a positive outpatient environment. A patient is provided a journey from surgery to treatment to reconstruction and finally restoration. The impact of this clinic is immeasurable.

I cannot put into numbers, the tears of joy and gratitude at closure of years of pain when the patient feels whole again. Loving your body again after cancer surgery is confidence and completeness.

### 73.4. Discussion or Conclusions

Starting as a project of need and compassion in a part-time clinic, it has grown 600% with waiting list and high satisfaction. Despite dramatic scars, patients are so grateful for the tattoo of restoration to emotionally complete the complications and pain.

The long-term sustainability is robust in this program. It does not rely on hospital budget or funding. The physician is independent within the hospital. Initial costs for tattoo machine, inks and equipment of high quality is $1500 and low ongoing costs. A small room, initially shared with other specialties was required only.

Call of interest resulted in 44 physicians with hospital affiliation interested in training in restorative areola tattooing and setting up a clinic in their hospitals. This would improve the 50% of patients that are coming from outside the Halton region and as far away as Sudbury, Winsor and London. The goal of training is also to recognize the medical, surgical and psychological benefits and have CME accreditation for the first time. The course will work with a highly qualified tattooist, bridging the gap to bring this back into the hospital. It will have an asynchronous component and an intensive on-site weekend that can be booked independently.

This program is awaiting CME accreditation as an important part of the team of breast cancer treatment.

**28**

## 74. Exploring Online Support Group (OSG) Use Among Sexual and Gender Diverse (SGD) People Diagnosed with Cancer in Canada

Lauren Squires ^1,2^, Lori Ross ^1^, Aisha Lofters ^3^, Jackie Bender ^1,2^

1. Dalla Lana School of Public Health, University of Toronto, Toronto, Canada.
2. Department of Supportive Care, University Health Network, Toronto, Canada.
3. Women’s College Hospital, Toronto, Canada.

### 74.1. Background/Rationale or Objectives/Purpose

Online support groups (OSGs) are a convenient way to access information and peer support. Sexual and gender diverse (SGD) people diagnosed with cancer often struggle to find relevant supports, contributing to high levels of unmet needs. OSGs may be a way they can find much-needed support and community with similar others, yet little is known about OSG use/non-use among this population.

### 74.2. Methodology or Methods

In-depth interviews were conducted with SGD people who were 19 or older, diagnosed with cancer, and living in Canada recruited using targeted maximum variation sampling. Data were analyzed using reflexive thematic analysis guided by an intersectional theoretical framework.

### 74.3. Impact on Practice or Results

Twenty interviews are planned, and nine have been completed to date (average age = 49, 6 trans or non-binary, 8 white, 7 breast/chest cancer). Through preliminary data analysis we have generated the following broad themes related to participants’ experiences using OSGs: reasons for use/non-use, the ability of OSGs to meet their needs, openness to SGD users, engagement and posting behaviours, and overall group culture/dynamics. Different interrelated contextual factors influence these experiences, such as experiences with healthcare providers, own/healthcare provider knowledge of SGD-specific resources, demographic and clinical characteristics, and confidence in self-advocating.

### 74.4. Discussion or Conclusions

For many, relevant SGD-specific OSGs are difficult to locate, contributing to some choosing to leave their SGD identities out of their OSG use to ensure their cancer-related needs are met. We anticipate that insight gained from our findings will contribute to more equitable cancer support services to address the diverse needs of SGD communities across Canada.

**36**

## 75. “Calling In” Practices of Equitable Psychosocial Support for Structurally Underserved Communities

Paula Holmes-Rodman, Michelle Audoin, Kathy Barnard, Louise Binder, Martin Dawes, Amy Rosvold, Michael Smylie, Leah Stephenson, Jennifer Rayner, Antonella Scali, Martine Elias, Fred Horne, Josee Pelletier, Tina Sahay, Rebecca Turner

All.Can Canada.

### 75.1. Background/Rationale or Objectives/Purpose

Equitable cancer diagnosis and care in Canada remains a significant challenge for structurally underserved populations, including FNIM, LGBTQ2S+, refugees, immigrants, racialized communities, and those living with pre-existing functional and cognitive challenges.

### 75.2. Methodology or Methods

At its third Roundtable, “Optimizing Equitable Cancer Diagnoses in Canada” (2024), All.Can Canada invited a wide range of participants from groups who identified extensive and diverse health inequities during cancer diagnosis, including the lack of culturally responsive psychosocial supports.

The resulting “What We Heard” Reports underscored multiple and intersecting challenges that inform the entire healthcare experience, including the availability, accessibility, and appropriateness of psychosocial supports grounded in cultural humility and safety.

### 75.3. Impact on Practice or Results

Our workshop is a “calling in” of Canadian practices of equity-oriented psychosocial supports. The first part will outline what ACC has learned about (in)equitable cancer diagnoses, followed by a “Morbidity and Mortality Rounds” where participants share cases that provide lessons in terms of cognitive and structural errors while identifying systemic issues. The second part will be small group “Lighting Rounds” of practices that can be emulated, spotlighted and adapted to new contexts.

### 75.4. Discussion or Conclusions

Participants will learn in “rounds” settings about:

1. specific cases where the psychosocial supports offered were refused or deemed inappropriate for equity-based issues.
2. how organizations and colleagues are adapting diversity, equity and inclusion work to speak directly to psychosocial needs of equity-deserving and rights-holding groups.
3. Canadian “bright spots” that can be highlighted and adapted to better meet the needs of structurally underserved populations.

**53**

## 76. Algonquins of Pikwakanagan Ways of Knowing and Doing in Cancer Care

Mohamad Hamze Al-Chami ^1^, Wendy Gifford ^1^, Peggy Dick ^2^, Maggie Benoit ^2^, Ellen Gandy ^1^

1. University of Ottawa, Ottawa, Canada.
2. Algonquins of Pikwakanagan First Nation—Family Health Team—Minopimàdiz-i Gamik, Pikwakanagan, Canada.

### 76.1. Background/Rationale or Objectives/Purpose

First Nations people in Canada have higher cancer rates relative to the general population with distinct transitional care needs based on First Nations’ ways of knowing and doing. Ongoing legacies of colonization continues to create structural racism and erase First Nations knowledges, values and practices in dominant models of cancer care.

In this study we explored a culturally safe cancer care strategy with the Algonquins of Pikwakanagan First Nations community to support cancer care throughout the care continuum.

### 76.2. Methodology or Methods

A participatory mixed methods approach was used. We worked with an Advisory Group from the community of knowledge keepers, Elders and health care providers. We held one sharing circle and 6 individual interviews with cancer survivors and family members (n = 12) and one focus group with health and social service providers (n = 6) for a total of 18 participants. Thematic analysis was done inductively and final categories were validated and refined based on community feedback.

### 76.3. Impact on Practice or Results

Two areas emerged as important to cancer care: (1) Information about cancer, treatment options and how to safely navigate the health care system; and (2) Cultural knowledges and practices that included spirituality as healing, and family and community members “being there”. Participants emphasized these areas help bridge traditional and western approaches while confronting mistrust in settler biomedical healthcare systems.

### 76.4. Discussion or Conclusions

Findings confirm that cultural practices are crucial in any cancer care strategy to support First Nations people and are necessary to prevent the perpetuation of neocolonial models of care that continue to harm Indigenous communities.

**56**

## 77. Building Cancer Care with Inuit in Inuvialuit Settlement Region

Wendy Gifford ^1^, Reyna Uriarte ^1^, Liquaa Wazni ^1^, Karis Gruben ^2^, Ellen Gandy ^1^, Tapisa Kilabuk ^2^

1. University of Ottawa, Ottawa, Canada.
2. Pauktuutit Inuit Women of Canada, Ottawa, Canada.

### 77.1. Background/Rationale or Objectives/Purpose

Inuit have significantly higher prevalence and poorer survival for almost all cancers relative to the Canadian population. While causes are complex and include low screening rates and late-stage diagnoses, more pervasive reasons stem from ongoing legacies of colonization and residential schools that have created structural racism, social exclusion, and erasure of Inuit knowledges, values and practices in settler health care. Inuit have not been meaningfully included in research to address their cancer care needs.

### 77.2. Purpose/Objectives

Researchers from University of Ottawa and Pauktuutit Inuit Women of Canada are conducting a study to understand *how cancer care can be strengthened for Inuit living in Inuvialuit Settlement Region.* Specific objectives include understanding what good cancer care looks like, and how it can be integrated into social and health care systems.

### 77.3. Methodology or Methods

We held focus group discussions with Inuit cancer survivors and family members in Inuvik NWT in November 2023 (n = 8) and July 2024 (n = 8). Qualitative thematic analysis as outlined by Braun & Clarke (2006; 2020) was conducted and preliminary findings taken back to the community for refinement and validation.

### 77.4. Impact on Practice or Results

Participants revealed that community members were reluctance to discuss cancer, holding an enduring fear associated with the word cancer. Themes for good cancer care involved: cancer care as a reflective and transformative journey; upholding Inuit ways of knowing and doing; and self-determination.

### 77.5. Discussion or Conclusions

Developing support systems founded on Inuit values, knowledge, cultural norms and practices within all stages of the cancer trajectory is essential for cancer care to be strengthened for Inuit.

**62**

## 78. Cross-Sectional and Qualitative Assessment of Sexual Health in Patients with a Vagina Receiving Pelvic Radiotherapy

Kaviya Devarajah ^1,2^, Anjali Sachdeva ^3^, Karina Gandhi ^4^, Abha A. Gupta ^5,6^, Jennifer Croke ^7^

1. Adolescent and Young Adult Program, Department of Supportive Care, Princess Margaret Cancer Centre, University Health Network, Toronto, Canada.
2. Institute of Medical Science, University of Toronto, Toronto, Canada.
3. Temerty Faculty of Medicine, University of Toronto, Toronto, Canada.
4. Department of Psychology, University of Guelph, Guelph, Canada.
5. Division of Medical Oncology and Hematology, Princess Margaret Cancer Centre, University Health Network, Toronto, Canada.
6. Adolescent and Young Adult Program, Department of Supportive Care, Princess Margaret Cancer Centre, University Health Network.
7. Radiation Medicine Program, Princess Margaret Cancer Centre, University Health Network, Toronto, Canada.

### 78.1. Background/Rationale or Objectives/Purpose

Pelvic radiotherapy (PRT) is an effective cancer treatment for pelvic malignancies but often leads to sexual dysfunction and long-term complications, particularly for patients with a vagina. These challenges are amplified in adolescent and young adult (AYA) patients due to unique developmental milestones, significantly affecting relationships, self-esteem, and quality of life. This study investigates the sexual health experiences of AYAs undergoing PRT to identify care gaps and inform tailored support and resources.

### 78.2. Methodology or Methods

Participants included 58 female AYA patients (mean age: 32.8) undergoing PRT at Princess Margaret Cancer Centre.

This mixed-methods study employed surveys to assess changes in sexual health and care satisfaction and conducted interviews to explore sexual dysfunction and needs. Descriptive statistics summarized survey data, while thematic analysis, guided by Braun and Clarke’s framework, identified key themes. Triangulation compared survey and interview findings for a comprehensive understanding.

### 78.3. Impact on Practice or Results

Participants reported significant challenges, including pain during intercourse, loss of libido, diminished intimacy, communication barriers, fertility difficulties, and psychosocial distress. Thematic analysis revealed three main themes: (1) managing the impact of changes in sexual function on relationships and intimacy, (2) navigating sexual health changes affecting fertility and family planning, and (3) understanding the emotional and psychosocial toll of physical discomfort and dysfunction.

### 78.4. Discussion or Conclusions

The findings underscore the urgent need for comprehensive, developmentally tailored interventions to support the sexual health of AYA patients undergoing PRT, addressing both physical and psychosocial dimensions.

**77**

## 79. Coming Out in Cancer Care: Understanding Sexual Identity Disclosure Experiences for Young Lesbian, Gay, Bisexual and Queer Cancer Patients

Whitney Qualls ^1^, Jonathan Avery ^2^, Helen McTaggart-Cowan ^2,1^, Travis Salway ^1^, Will Small ^1,3^, Stuart Peacock ^1,2^

1. Simon Fraser University, Vancouver, Canada.
2. BC Cancer, Vancouver, Canada.
3. British Columbia Centre on Substance Use, Vancouver, Canada.

### 79.1. Background/Rationale or Objectives/Purpose

The objective of this study is to explore the sexual identity disclosure experiences of queer adolescent and young adult (AYA) cancer patients and survivors during cancer care. Specifically, the study aims to examine how queer AYAs navigate disclosure of their sexual identity during active treatment and survivorship care, as well as how cancer care providers facilitate these conversations.

### 79.2. Methodology or Methods

Sample and setting: 25 cancer care providers and 25 queer AYA cancer patients and survivors who live in Canada.

Procedures: 45–90 min semi-structured qualitative interviews held via Zoom or in person.

Impact on practice or Results

This study is ongoing but preliminary findings include the following emerging themes:

1. Providers want to be told; patients want to be asked: Cancer care providers prefer that patients initiate conversations about sexual identity, while AYAs often wait for providers to take the lead.
2. Additional mental load during cancer care: Queer AYAs report that concealing their sexual identity is exhausting. However, when they disclose, they frequently face the burden of educating providers about queer health issues.
3. Expectations of queer competency: While acknowledging challenges in the Canadian healthcare system, queer AYAs expect providers to be knowledgeable and competent in queer-specific health issues.

### 79.3. Discussion or Conclusions

The findings can guide the development of tailored oncological health education modules, focusing on the unique needs of queer AYAs. The insights gained can support the creation of institutional-level inclusivity initiatives tailored for queer AYAs in cancer care. Additionally, this research highlights the need to expand disclosure models, which predominantly reflect women’s experiences, to better encompass the diverse experiences of queer AYAs.

**112**

## 80. Supportive Care Needs of Rural Canadian Cancer Patients

Alyssa Dalinda, Samantha Mayo

Princess Margaret Cancer Centre, Toronto, Canada.

### 80.1. Background/Rationale or Objectives/Purpose

Rural oncology patients often encounter significant barriers to accessing specialized cancer care due to their geographic isolation, logistical challenges of traveling long distances for care and the social and financial burdens associated with relocating for care. As a result, addressing the comprehensive supportive care needs of these patients, including physical, emotional, social, spiritual and psychological domains, is essential to ensure they can continue to receive their oncology care. However, rural patients may face challenges in accessing supportive care that is both geographically convenient and culturally sensitive. This presentation aims to explore the question: “How can oncology nurses effectively address the supportive care needs of rural Canadian cancer patients?”. Their proximity to patients, combined with their leadership and advocacy roles, positions nurses to identify and address supportive care needs of cancer patients in a timely and culturally appropriate manner. Nurses can play a pivotal role in improving care for rural cancer patients.

### 80.2. Methodology or Methods

A narrative literature review will be conducted to synthesize the most current and relevant data on this topic. The review will focus on peer-reviewed articles published in Canada from 2014 onward. An equity-focused approach will guide the critical analysis of the literature to better understand the unique supportive care needs and experiences of rural Canadian cancer patients, and the opportunities for oncology nurses to provide tailored support based on their scope of practice.

### 80.3. Impact on Practice or Results

Preliminary findings indicate that rural Canadian cancer patients face a range of unique supportive care challenges related to their geographic location, leaving many of their needs unmet. These individuals often experience worsened symptoms, heightened social isolation, and limited access to culturally safe support. Transportation and limited access to specialized care are significant barriers to addressing these needs. Integrating digital health technologies into care plans could enhance the ability of healthcare providers, particularly nurses, to better meet the supportive care needs of rural cancer patients by improving access to essential services.

### 80.4. Discussion or Conclusions

The findings from the study will provide valuable insights into the distinctive supportive care needs of rural cancer patients compared to their urban counterparts. The research will offer recommendations for nurses and other healthcare providers on how to effectively support rural oncology patients, emphasizing nursing strategies that consider both equity and patient-centered care.

**116**

## 81. Understand the Importance of Integrating Indigenous Coping Mechanism When Working with Chinese Cancer Patients

Evaon Wong

Portland State University, Portland, USA.

### 81.1. Background/Rationale or Objectives/Purpose

Clinicians working with oncology patients need to understand how to value their indigenous practice of healing and coping with their cancer diagnosis, especially those who are newly diagnosed with an advance stage of the disease. Being able to understand the patients’ use of cultural relevant coping mechanisms will help build a respectful relationship and foster trust with them.

### 81.2. Methodology or Methods

Needs assessment: Key informants’ studies were conducted with patients who volunteers with nonprofit organizations conducting Cantonese speaking support groups. These volunteers are valuable assets to bridge clinicians understanding of minoritized patients use of alternative coping mechanisms such as fortune telling, Feng Shui, or geomancy. One of the examples of popular fortune telling is shortage, a Daoist practice of prediction that many Chinese patients use to predict the success of their treatment for their cancer diagnosis.

### 81.3. Impact on Practice or Results

These informants explained that they do not expect social workers, or psychologists to understand how they use these indigenous practices; however, they do hope that clinicians will be willing to ask questions and understand their emotional reaction to these practices, that sometimes may help with their emotional well beings, or sometimes negative affect their mood and depressive symptoms when these predictions turn out to be negative.

### 81.4. Discussion or Conclusions

More trainings for clinicians to understand these practices will help them utilize patients’ internal and cultural resources. Clinicians’ curiosity about these practices will help Chinese patients feel more supported and less ridiculed or alienated when working as a team.

**137**

## 82. Palliative Care for People with Severe and Persistent Mental Illness

Alan Bates ^1,2^, John-Jose Nunez ^1,2^, Anne Woods ^3^

1. BC Cancer, Vancouver, Canada.
2. UBC Psychiatry, Vancouver, Canada.
3. Division of Palliative Care, McMaster University, Hamilton, Canada.

### 82.1. Background/Rationale or Objectives/Purpose

People with severe and persistent mental illness have unique needs that create challenges in implementing equitable palliative care. Factors related to ease of access, setting of care, advanced care planning, interdisciplinary care, and patient decision-making capacity and autonomy must be considered.

### 82.2. Methodology or Methods

We conducted an extensive literature review of palliative care for people with severe and persistent mental illness.

### 82.3. Impact on Practice or Results

Palliative care for people with severe and persistent mental illness needs to be tailored to each mental health syndrome. While medication interactions and assessment of decision-making capacity might be particularly relevant in schizophrenia, a trauma-informed approach to care might be a greater focus for someone with a personality disorder and comorbid substance use disorder. Syndromes such as anorexia nervosa emphasize the need for interdisciplinary care and the overlap between mental health care and palliative care.

### 82.4. Discussion or Conclusions

Cross-disciplinary training and interdisciplinary care settings that include staff with expertise in both mental health care and palliative care will be key to advancing palliative care for people with severe and persistent mental illness. Existing healthcare settings may not be adequate for some populations and ethical considerations that affect care trajectories will also likely continue to evolve.

**157**

## 83. Challenges and Opportunities in Supporting Family Caregivers of Advanced Cancer Patients with Limited Language Proficiency: A Focus Group Study

Paige Chu, Shabbir Alibhai, Marianna Calamia, Sarah Hales, Breffni Hannon, Madeline Li, Martine Puts, Rinat Nissim

Princess Margaret Cancer Centre, Toronto, Canada.

### 83.1. Background/Rationale or Objectives/Purpose

Family caregivers supporting individuals with advanced cancer provide essential support, and often face significant distress themselves. Emerging literature shows that caring for a loved one with Limited Language Proficiency (LLP) can present additional, unique challenges that further caregiving burden despite the availability of interpretation services offered in many healthcare settings. In this study, we seek to understand the experience of such caregivers, examine the unique challenges and barriers they face and identify potential solutions to improve support for both patients with LLP and their caregivers.

### 83.2. Methodology or Methods

We are currently conducting focus groups and one-on-one qualitative interviews with family caregivers who are supporting patients with advanced cancer and LLP and who are receiving treatment at the Princess Margaret Cancer Centre in Toronto, Ontario. Focus groups will be formed based on participant ethnicity such that cultural impacts on their experiences can be highlighted and factored into this exploration. Questions asked will focus on the following broad categories: healthcare navigation; understanding medical information; use of interpreters; cultural differences; support and resources; and suggestions for improvement. Responses will be analysed using thematic analysis.

### 83.3. Impact on Practice or Results

Recruitment opened in November 2024; we plan to conduct four focus groups, each including approximately 6-8 caregivers, with the first group scheduled for January 2025. Preliminary results will be presented, with a focus on identifying unique challenges, barriers and solutions for this caregiver population.

### 83.4. Discussion or Conclusions

Findings will help contribute toward intervention development and improved language-and culture-based policies and programs to better support diverse cancer patient populations and their families.

## 84. Final Category: G. Health Care Provider Wellness

**16**

## 85. Narrative Prescriptions: Nurturing Wellness for Psychosocial Care Providers

Paula Holmes-Rodman ^1^, Mireille de Reland ^2^

1. Courtenay, Canada.
2. Toronto, Canada.

### 85.1. Background/Rationale or Objectives/Purpose

Narrative medicine (NM) integrated with mindfulness is a valuable and accessible tool for psychosocial healthcare providers, offering a source of healing, nourishment, and resilience. Individual and collective practices include reflective writing, storytelling, and NM informed mindfulness techniques. These modalities support providers to process the emotional demands of their work, alleviate compassion fatigue and foster a sense of purpose and resilience.

### 85.2. Methodology or Methods

Our program, “Narrative Prescriptions” was created for hospice clinicians, staff and volunteers. It has been adapted for use in the psychosocial oncology space.

Narrative Prescriptions uses practical, evidence-based NM theory and mindfulness approaches in an inclusive space, available online or in person nationwide. Program methods include (1) expressive writing, (2) attentive listening and reading, (3) group discussion and sharing, (4) mindfulness practices, and (5) other creative modalities.

### 85.3. Impact on Practice or Results

Engaging in NM and mindfulness within peer groups fosters community support, reduces isolation, and creates a shared sense of purpose. By reflecting on their experiences within a collective, providers learn to adapt to professional challenges with an open, growth-oriented mindset. Our program benefits those working in psychosocial care to reconnect with the values that brought them to their profession, deepening empathy and strengthening patient-provider relationships.

### 85.4. Discussion or Conclusions

The potent combination of NM and mindfulness, developed specifically for psychosocial healthcare providers, not only supports their mental well-being but also enhances the quality of care, as professionals engage more fully with their patients. Narrative Prescriptions cultivates a sustainable, caring, compassionate approach to psychosocial care, enabling providers to continue their essential work with renewed energy, heart and compassion.

## 86. Final Category: H. High Tech to High Touch (Digital Health, Value-Based Care, Integrated PSO Interventions)

**11**

## 87. Patients’ Attitudes Toward Artificial Intelligence in Cancer Care: A Scoping Review

Daniel Hilbers ^1^, Navid Nekain ^1^, Alan Bates ^2,3^, John-Jose Nunez ^2,3^

1. Faculty of Medicine, University of British Columbia, Vancouver, Canada.
2. Department of Psychiatry, University of British Columbia, Vancouver, Canada.
3. BC Cancer, Vancouver, Canada.

### 87.1. Background/Rationale or Objectives/Purpose

To explore the attitudes of patients with cancer toward the integration of artificial intelligence in their medical care and identify knowledge gaps.

### 87.2. Methodology or Methods

We followed the PRISMA-ScR guidelines and applied a conceptual framework by Richardson et al. to develop a comprehensive search strategy. We searched MEDLINE (OVID), EMBASE, PsycINFO, and CINAHL for peer-reviewed primary research articles. Studies with quantitative or qualitative data on patients with cancer and their attitudes toward AI were included. Two independent reviewers screened the articles, with a third resolving disagreements. Data were synthesized into tabular and narrative summaries.

### 87.3. Impact on Practice or Results

Our search yielded 1093 citations, of which 15 articles met the inclusion criteria. The studies, published from 2019 onward, involved adult patients with cancer in middle to late adulthood, across clinical settings in North America, Europe, and Asia. They focused on prostate, melanoma, breast, and colorectal cancers. Most studies were qualitative, using interviews, surveys, or focus groups for data collection. We categorized the findings, highlighting key attitudes toward artificial intelligence in medical care, including optimism, concerns, preferences for AI-based care versus traditional care, and measures of trust, satisfaction, and fear.

### 87.4. Discussion or Conclusions

This is the first scoping review to map the attitudes of patients with cancer toward artificial intelligence in medical care. Gaps in the literature highlight the need for future research on younger adults and patients with cancer at different stages and types. Clinically, understanding patient attitudes toward AI can guide the integration of these technologies into cancer care, ensuring alignment with patient preferences and enhancing both acceptance and effectiveness.

**18**

## 88. Building an AI-Powered Personalized Cancer Care Navigation Assistant

John-Jose Nunez ^1,2^, Jon Avery ^1^, Alan Bates ^1,2^

1. BC Cancer, Vancouver, Canada.
2. University of British Columbia, Vancouver, Canada.

### 88.1. Background/Rationale or Objectives/Purpose

Navigating the different types of support available to patients with cancer—such as mental health, palliative care, or nutritional advice—can be challenging. This project aims to develop an AI-powered cancer care navigation assistant that to offers personalized recommendations by predicting supportive care needs, clearly explain these recommendations, and facilitates timely actions like initiating self-referrals or alerting care providers.

### 88.2. Methodology or Methods

We recently developed AI tools that analyze oncologists’ consultation notes to predict supportive care needs. The proposed navigation assistant will use these predictions to explain recommendations to patients and offer actions, such as triggering self-referrals or notifying care providers. A pilot study will test the feasibility of this approach. This workshop will include a discussion to gather feedback from attendees on features they consider most helpful in such a navigation assistant.

### 88.3. Impact on Practice or Results

This assistant could help patients navigate resources during their cancer journey by improving access to care, empowering discussions with their care team, and reducing feelings of being overwhelmed at the start of treatment.

### 88.4. Discussion or Conclusions

This project aims to develop an AI-powered navigation assistant to guide patients through their cancer journey, building on prior work predicting supportive care needs. We hope our presentation will engage the audience in refining the assistant’s design to ensure alignment with the needs of research users including cancer clinicians, researchers, and patients.

**35**

## 89. Youth and Young Adult-Driven Augmented Reality for Vaping Cessation: An Interpretive Description Study

Karlee Fonteyne, Laura Struik, Elizabeth Keys

University of British Columbia Okanagan, Kelowna, Canada.

### 89.1. Background/Rationale or Objectives/Purpose

This study aimed to explore youth and young adults’ (YYAs) experiences with vaping cessation and their perceptions of augmented reality (AR) as a tool for supporting quitting efforts. The goal was to inform the design of AR-based interventions that address YYA-specific motivations, barriers, and preferences. Vaping carries a known cancer risk and increases the likelihood of transitioning to smoking, the leading preventable cause of cancer. Early cessation interventions are critical to reducing long-term health risks.

### 89.2. Methodology or Methods

#### 89.2.1. Sample and Setting

Participants were 12 YYAs aged 18–24, recruited using purposive sampling. Eligibility required current vaping, motivation to quit, and residency in Canada. Data collection occurred virtually through Zoom.

#### 89.2.2. Procedures

The study used qualitative interpretive description methodology. Participants engaged in semi-structured interviews discussing their quitting journeys and preferences for prospective AR features in a mobile app. Data were analyzed using Braun and Clarke’s thematic analysis framework, supported by NVivo software, to identify patterns and themes.

### 89.3. Impact on Practice or Results

Findings revealed that quitting vaping is a deeply individualized process influenced by health concerns, financial burdens, and social pressures. Participants favoured app features like behaviour tracking, goal setting, and anonymous peer support but expressed concerns about privacy and app costs. AR features, including health visualizations, gamification, and real-time support, were identified as promising tools to sustain engagement and manage cravings. Participants also highlighted the need for interventions to be nonjudgmental and highly personalized.

### 89.4. Discussion or Conclusions

This study highlights the potential of AR-based interventions to bridge gaps in vaping cessation support for YYAs. By addressing individual and social needs, AR can enhance engagement and empower users, offering an innovative pathway to reducing vaping prevalence. Supporting improved cessation at an early age can significantly reduce cancer risk, fostering the health and well-being of future generations.

**73**

## 90. Lay Navigation Interventions for Patients with Cancer: A Review of the Literature

Sharlane C. L. Lau ^1^, Hanae Davis ^1^, Richa Srivastava ^1^, Megan Wexler ^1^, Jackie Bender ^1,2^, Alana Changoor ^1,3^, Rochelle Kahn ^1,3^, Natasha Leigh ^1,2^, Gary Rodin ^1,2^, Gilla K. Shapiro ^1,2^

1. Princess Margaret Cancer Centre, Toronto, Canada.
2. University of Toronto, Toronto, Canada.
3. Patient Partner Program, University Health Network, Toronto, Canada.

### 90.1. Background/Rationale or Objectives/Purpose

Patient navigation can address barriers to care and improve outcomes for patients with cancer by providing informational, practical, and emotional support. Distinct navigation models have been implemented, but available literature reviews have not disaggregated evidence from clinical (professional) navigation and lay navigation (LN) programs. The objective of this review is to synthesize the published literature on LN in patients with cancer and identify what outcomes have been examined, the measures used, and their impact.

### 90.2. Methodology or Methods

This narrative review included studies conducted in English on LN in patients with cancer, without restrictions on care setting, cancer type, or stage. We synthesized evidence from relevant studies including study details (authors, publication date), LN program, study design (sample, outcomes, measures), and findings.

### 90.3. Impact on Practice or Results

We reviewed 41 studies. LN programs varied in their goals, operations, whether the navigator had cancer (patient/peer), and navigator training. We grouped study outcome measures into four categories: 1. treatment participation; 2. experience of care; 3. quality of life and symptoms; and 4. costs. While findings were mixed, in many studies LN reduced time to treatment, healthcare utilization, and medical costs; improved access to quality care and clinical trials; and addressed practical barriers to care.

### 90.4. Discussion or Conclusions

LN programs in cancer care hold strong potential to be a cost-effective solution to improve patient outcomes and health care access. Given mixed findings, future research should identify the settings, target populations, program components, and practice domains to maximize LN benefits. This review offers recommendations to guide the implementation of LN in oncology settings.

**90**

## 91. Exploring the Feasibility, Acceptability, and Impact of iCANSleep: A Mobile App for Insomnia in Cancer

Katherine-Ann Piedalue, Krista Greely, Rachel Lee Dauz, Sheila Garland

Memorial University of Newfoundland, St. John’s, Canada.

### 91.1. Background/Rationale or Objectives/Purpose

Individuals who have been diagnosed with cancer, experience insomnia rates 2–3 times higher than the general population, which is a significant barrier to returning to usual functioning after cancer. Cognitive behavioural therapy for insomnia (CBT-I) is the recommended treatment for insomnia, but it is largely inaccessible due to a shortage of providers. iCANSleep is a smartphone app that was co-designed with patients to deliver CBT-I, is one way to increase the accessibility of CBT-I. This study presents interim feasibility, acceptability, and efficacy outcomes of the iCANSleep app.

### 91.2. Methodology or Methods

Individuals with a history of cancer and insomnia disorder were recruited to test the app’s the feasibility and acceptability. The Acceptability E-Scale (total scores range from 5–30) examined the apps acceptability, while the Insomnia Severity Index measures insomnia symptoms. The Patient-Reported Outcome Measurement Information System (PROMIS) was used to measure fatigue, cognitive function, anxiety, and depression before and after the program. Descriptive statistics and paired sample t-tests were used to examine differences before and after CBT-I.

### 91.3. Impact on Practice or Results

To date, 54 participants have been recruited (M_age_ = 54.56 years, 94% women). Twenty participants have finished the program, 25 are currently using the app, 5 are in the onboarding process, and 4 have been lost to contact. Completers reported a high Acceptability E-Scale score (*M* = 27.5). Participants reported a significant reduction in insomnia [(19) = 7.92, *p* < 0.001], fatigue, [t(19) = 3.76, *p* < 0.001], cognitive impairment, [t(19) = 3.53, *p* = 0.002], and anxiety, [t(19) = 5.01, *p* < 0.001]. There was no significant difference in depression.

### 91.4. Discussion or Conclusions

The iCANSleep app may be a promising tool to provide CBT-I to individuals with a history of cancer who are experiencing insomnia. After completing feasibility testing, the app will be modified as needed, and a randomized controlled trial will formally assess its efficacy.

**118**

## 92. Innovation of the Care and Connect Program: Using Lay Navigators to Deliver Virtual Support

Richa Srivastava ^1,2^, Hanae Davis ^1,2^, Megan Wexler ^1,2,3^, Gilla Shapiro ^1,3,4^, Gary Rodin ^1,2,3^

1. Cancer Experience Program, Princess Margaret Cancer Centre, Toronto, Canada.
2. Care and Connect, Princess Margaret Cancer Centre, Toronto, Canada.
3. Department of Supportive Care, Princess Margaret Cancer Centre, Toronto, Canada.
4. Dalla Lana School of Public Health, University of Toronto, Toronto, Canada.

### 92.1. Background/Rationale or Objectives/Purpose

Launched in 2007, Care and Connect (CC) is a patient navigation program at Princess Margaret Cancer Centre that provides psychosocial support, information, and system navigation through lay navigators. In March 2023, CC innovated its in-person support model to a virtual and longitudinal delivery to ensure equitable patient access. Preliminary program growth, utilization, and patient/navigator engagement results will be shared.

### 92.2. Methodology or Methods

CC manages a cohort of highly skilled and multilingual navigators who are proactively referred and matched to patients/caregivers based on patient preference and sociodemographic factors (e.g., age, sex, gender, language). The navigators provide psychosocial support and navigation through phone/video for over six months. Data collection is ongoing, focusing on program utilization, access to services, patient/navigator engagement metrics, and demographics. In 2024, a quality improvement evaluation was initiated to support iterative program growth through qualitative interviews (n = 10).

### 92.3. Impact on Practice or Results

Preliminary findings show increases in program utilization, navigator caseloads, and improved ability to meet the diverse needs of patients/caregivers. The shift to a virtual care model has strengthened support for patients/caregivers by fostering long-term relationships, detecting clinical complexities, and empowering patients/caregivers. Early quality improvement findings suggest streamlined referrals, technological infrastructure, sustained funding, and clinician stakeholder engagement as key drivers of program growth.

### 92.4. Discussion or Conclusions

This quality improvement evaluation has demonstrated CC’s ability to provide continuous and personalized support to patients/caregivers while highlighting its potential to scale virtual psychosocial support and provide equitable navigation through lay navigators. Future directions involve a formal evaluation of CC’s efficacy and its impact on health system utilization.

**128**

## 93. Implementing REmote SymPtom mOnitoring and maNagement (RESPONd) in Cancer Care Alberta: Using Patient-Reported Outcomes to Personalize Patient Care

Claire Link ^1^, April Wales ^1^, Andrea DeIure ^1^, Linda Watson ^2^

1. Cancer Care Alberta, Calgary, Canada.
2. Cancer Care Alberta, Alberta Health Services, Calgary, Canada.

### 93.1. Background/Rationale or Objectives/Purpose

In Cancer Care Alberta (CCA), Patient-Reported Outcomes (PROs) are collected at clinic appointments as part of standard practice, using a routine questionnaire to assess symptoms and supportive care needs common for patients with cancer. PROs help care teams personalize symptom management, resources, and support to meet each patient’s needs. Recently, CCA received funding to implement a digital remote symptom monitoring and management program called RESPONd, providing patients the opportunity to complete PROs from home between appointments and receive tailored advice from a nurse.

### 93.2. Methodology or Methods

RESPONd will be implemented over 1.5 years in six clinics, spanning tertiary, regional, and community cancer centres. Study nurses will monitor PROs questionnaires completed between clinic visits and contact patients to provide personalized support. PROs information will also be utilized during weekly triage discussions in each clinic, where the route of care will be tailored for patients based on their symptom complexity.

### 93.3. Impact on Practice or Results

The study will explore a person-centered, proactive approach to care and the potential benefits to patient experience, outcomes, and clinical teams. RESPONd represents an innovative opportunity to shift the model of care in oncology clinics to optimize efficiency and utilization of limited clinic resources and create a responsive care team that is ready and able to provide personalized symptom management and supportive care.

### 93.4. Discussion or Conclusions

This presentation discusses details of the RESPONd study, focusing on the rationale and anticipated outcomes for patients and care teams, and potential implications for the broader health system. Key learnings from the initial study phases will be shared.

**192**

## 94. Going Above and Beyond: Integrating the Patient Experience to Humanize Digitalization Projects in Cancer Care

Virginia Lee ^1,2,3^, Lauryn Santiago ^2^, Malcolm Baird ^2^, Anna Burgos ^2^

1. McGill University Health Centre, Montreal, Canada.
2. Cedars CanSupport, Montreal, Canada.
3. McGill University Ingram School of Nursing, Montreal, Canada.

### 94.1. Background/Rationale or Objectives/Purpose

As cancer patient volumes soar, the benefits of digitalization (namely cost savings, accessibility to information and efficiency of workflow processes) continue to drive technology into cancer care delivery systems at an accelerated pace. Unfortunately, the increased reliance on digitalization can also unintentionally dehumanize care. Automated activities that reduce in-person interactions can interfere with the capacity to offer and integrate fundamental values of respect, autonomy and individuality, and compassion. Yet, as clinical and complementary service delivery models become increasingly digitalized, examples of health care providers who have “gone above and beyond” continue to shine through.

### 94.2. Methodology or Methods

This presentation will describe how the humanization of care is prioritized at the McGill University Health Centre Cedars Cancer Centre in partnership with Cedars CanSupport, a non-profit organization that offers emotional, informational, practical, and financial support. We will describe innovative approaches used to uncover congruence or incongruence in terms of discerning what matters most between the sponsors and the patients of large organizational digitalization projects. We will present data and testimonials from health care providers, patient partner workgroups and patient experience surveys.

### 94.3. Impact on Practice or Results

Empowering patients to contribute their voice in digitalization projects is critical to leveraging high tech innovations that will be consistent with whole person cancer care.

### 94.4. Discussion or Conclusions

Ultimately, technology offers an indispensable tool to improve the patient experience if patients themselves are recognized as valuable stakeholders in the transformation of care.

## 95. Final Category: I. Implementation Science, Knowledge Translation and Synthesis

**7**

## 96. Mapping and Evaluating Measures of Self-Management (SM) and SM Support in Oncology: A Scoping Review in Progress

Sitara Sharma, Jennifer Brunet

University of Ottawa, Ottawa, Canada.

### 96.1. Background/Rationale or Objectives/Purpose

Given the burgeoning focus on self-management (SM) in oncology, a scoping review and evaluation of relevant measures is key to guiding its assessment in future research and practice. Thus, we aim to: (1) synthesize and evaluate the quality of measures used to assess SM and SM *support* by individuals living with and beyond cancer or their healthcare providers (HCPs), and (2) identify gaps in SM/SM support measurement in oncology.

### 96.2. Methodology or Methods

This scoping review will follow Arksey and O’Malley’s (2005) five-step framework, enhanced by Levac et al. (2010). Four electronic databases were searched to identify relevant articles in July 2024. Currently, two independent reviewers are screening > 6500 retrieved titles/abstracts; full-text screening of retained articles will follow using two inclusion criteria: (1) peer-reviewed English articles, and (2) ≥1 measure assessing SM/SM support components [e.g., patient/provider activation, self-efficacy, decision-making] in individuals living with and beyond cancer or oncology HCPs. Extracted data will include study/sample/intervention characteristics, along with SM/SM support frameworks and measures. Measures will be classified (e.g., by scope, focus, dimensionality, intended use), and appraised using the *COnsensus-based Standards for the selection of health Measurement Instruments* (COSMIN) checklist. Findings will be mapped narratively/tabularly, shared with an Advisory Council of HCPs and patient representatives for review, and refined based on their input.

### 96.3. Impact on Practice or Results

N/A—protocol

### 96.4. Discussion or Conclusions

Results will offer a comprehensive overview of existing SM/SM support measures used in oncology, highlighting their methodological strengths/weaknesses and any critical gaps. This will enable more informed measure selection for SM/SM support by researchers and practitioners moving forward.

**67**

## 97. IMplementation of Physical Activity for Children and Adolescents on Treatment (IMPACT): Adapting to British Columbia and Ontario for Site Uptake

Amanda Wurz ^1^, S. Nicole Culos-Reed ^2^, Emma McLaughlin ^2^, Caron Strahlendorf ^3^, Kristin Marr ^4^, Hanna Lotocka-Reysner ^4^, Stuart Peacock ^5^, Iris Lesser ^1^, Sharon Hou ^5^, Fuchsia Howard ^6^, Kristin Campbell ^6^, Brianna Empringham ^7^, Raveena Ramphal ^7^, Lillian Sung ^8^, Donald Mabbott ^8^, Anna Janzen ^6^, Eden Baerg ^1^, Hayley Milburn ^1^, Megan Filiatrault ^1^

1. University of the Fraser Valley, Abbotsford, Canada.
2. University of Calgary, Calgary, Canada.
3. British Columbia Children’s Hospital, Vancouver, Canada.
4. Surrey Memorial Hospital, Surrey, Canada.
5. Simon Fraser University, Burnaby, Canada.
6. University of British Columbia, Vancouver, Canada.
7. Children’s Hospital of Eastern Ontario, Ottawa, Canada.
8. Hospital for Sick Children, Toronto, Canada.

### 97.1. Background/Rationale or Objectives/Purpose

Physical activity (PA) is beneficial for pediatric cancer patients. We developed IMPACT (IMplementation of Physical Activity for Children and adolescents on Treatment), a PA intervention delivered by Zoom that is being evaluated in Alberta. To adapt IMPACT for implementation, evaluation, and uptake, we will: (1) identify barriers/enablers to implementing and evaluating IMPACT in British Columbia (BC) and Ontario (ON), and (2) process map site-specific implementation strategies to develop an Implementation Research Logic Model (IRLM).

### 97.2. Methodology or Methods

A pragmatic orientation will be adopted, and interviews will be conducted with pediatric cancer patients (5–18 years) and their parents (n = 15 dyads/site) and healthcare providers (HCP) and staff (n = 15/site) in BC and ON. The interview guide follows from the Consolidated Framework for Implementation Research and Theoretical Domains Framework, and questions explore site-specific barriers/enablers to IMPACT across individual- and system-levels. Data will be transcribed and analyzed using thematic and framework analyses. Next, the same participants will engage in iterative process mapping to develop site-specific implementation strategies and an IRLM depicting pathways between implementation determinants, strategies, mechanisms, and outcomes.

### 97.3. Impact on Practice or Results

Data will be collected Spring 2025.

### 97.4. Discussion or Conclusions

Findings will reflect collaboration between patients and their parents, HCP, staff, and researchers in BC and ON. Site-specific implementation strategies and engaging ‘champions’ across sites will further drive implementation momentum. This critical phase of work that places patients, families, and clinical teams at the ‘centre’ of the research will ensure successful implementation, evaluation, and uptake and enhance the likelihood of future integration of PA within pediatric cancer patients’ clinical care.

**110**

## 98. Feasibility and Timing of Managing Cancer and Living Meaningfully (CALM) in Newly Diagnosed and Recurrent Ovarian Cancer

Megan George ^1,2^, Stephanie Lheureux ^3^, Paige Gibbins ^3^, Lauren Philp ^3^, Gary Rodin ^1^

1. Department of Supportive Care, Princess Margaret Cancer Centre, University Health Network, Toronto, Canada.
2. Institute of Medical Science, Temerty Faculty of Medicine, University of Toronto, Toronto, Canada.
3. Division of Gynecologic Oncology, Princess Margaret Cancer Centre, University Health Network, Toronto, Canada.

### 98.1. Background/Rationale or Objectives/Purpose

Traumatic stress symptoms (TSS) is common in women with both newly diagnosed and recurrent ovarian cancer (OC). Interventions such as Managing Cancer and Living Meaningfully (CALM) are effective in advanced cancer but their feasibility and acceptability at the time of OC diagnosis and recurrence have not been demonstrated.

### 98.2. Methodology or Methods

Patients > 18 years with newly diagnosed or recurrent OC recruited from the Gynecologic Oncology Clinic at Princess Margaret Cancer Centre in Toronto, Canada. Patients who provide informed consent are offered 3-6 sessions of CALM over 3-6 months. Validated patient-reported measures administered at baseline and at 3 and 6 months assess symptoms of traumatic stress, depression, death anxiety and perceived benefit of clinical care. Feasibility criteria include: >30% accrual of newly diagnosed and recurrent OC patients approached over 12-months; ≥64% of participants completing > 3 sessions over 6 months; ≥64% completion of outcome measures at each time point; >50% of participants report perceived benefit based on score ≥ 14 on the Clinical Evaluation Questionnaire (CEQ).

### 98.3. Impact on Practice or Results

Of the patients approached, 14/23 (60%) participants enrolled in the recurrent disease arm and 7/33 (21%) enrolled in the newly diagnosed cohort. Of the 7 participants who reached the second time point assessment, all have completed their outcome evaluations.

### 98.4. Discussion or Conclusions

Preliminary data indicate that it is feasible to initiate CALM at the time of OC recurrence, but more challenging at the time of new diagnosis. The high retention rate in these preliminary results speaks to the acceptability of the intervention. This study will inform the design of a future larger RCT.

**126**

## 99. Implementation of the Fear of Recurrence Therapy (FORT) Across Canada: Implementation Strategies and Tools

América Prudent ^1^, Alanna Chu ^1^, Florence Gourgues ^1^, Emma Kearns ^1^, Sara Beattie ^2^, Andrea Feldstain ^3^, Sheila Garland ^4^, Cheryl Harris ^5^, Jennifer Jones ^6^, Christine Maheu ^7^, Linda Carlson ^3^, Sophie Lebel ^1^

1. University of Ottawa, Ottawa, Canada.
2. Tom Baker Cancer Center, Calgary, Canada.
3. University of Calgary, Calgary, Canada.
4. Memorial University of Newfoundland, St John’s, Canada.
5. The Ottawa Hospital Research Institute, Ottawa, Canada.
6. Princess Margaret Cancer center, Toronto, Canada.
7. McGill University, Montreal, Canada.

### 99.1. Background/Rationale or Objectives/Purpose

Fear of cancer recurrence (FCR) is the fear, worry, or concern that cancer may come back or progress. It is the number one unmet need of cancer survivors, with up to 59% reporting clinical levels of FCR. FORT is an evidence-based intervention that consists of six weeks of cognitive-existential group therapy, based on a fear of recurrence theoretical model, with standardized manuals for survivors and clinicians. We are currently implementing FORT in five Canadian cancer centers and the next step is to establish the best implementation plan for each site. This study collected qualitative data from January-October 2023 from clinicians and decision-makers to identify implementation strategies and tools that were provided to each institution to ensure the successful implementation of FORT.

### 99.2. Methodology or Methods

A comparative approach employing the *Consolidated Framework for Implementation Research* (CFIR) and the *Expert Recommendations for Implementing Change* (ERIC) frameworks was used to assess key strategies and tools for FORT’s implementation in five Canadian clinical settings (i.e., The Ottawa Hospital, Princess Margaret Cancer Center, NL Health Services, McGill University Health Centre, Tom Baker Cancer Center). Semi-structured individual interviews of 90–120 min were administered to clinicians and decision-makers to enable comparisons between institutions.

### 99.3. Impact on Practice or Results

The study provides preliminary data on similarities (i.e., training, supervision) and specificities (i.e., modality, population variety) of implementation solutions and tools across sites. Analysis is expected to conclude in early 2025 and will be presented.

### 99.4. Discussion or Conclusions

Implementation outcome data will allow evaluations of findings such as: effectiveness, sustainability, implementation expansion and standard of care potential.

**130**

## 100. A Scoping Review of Psychosocial Distress Screening Tools for Pediatric Cancer Patients and Survivors: Identifying Optimal Instruments for Clinical Integration

Harsimronjoot Sidhu ^1^, Brianna Henry ^2^, Lindsay Jibb ^3^, Leandra Desjardins ^4^, Kyobin Hwang ^5^, Yustine Carruyo Soto ^6^, Victoria Forster ^7^, Chantale Thurston ^8^, Paul Nathan ^5,9^, Sapna Oberoi ^10,11^, Fiona Schulte ^12^

1.   University of Calgary, Calgary, Canada.
2.   Department of Psychology, University of Calgary, Calgary, Canada.
3.   Lawrence S. Bloomberg Faculty of Nursing, University of Toronto, Toronto, Canada.
4.   Charles-Bruneau Cancer Center, Sainte-Justine University Health Center, Montreal, Canada.
5.   The Hospital for Sick Children, Toronto, Canada.
6.   Charles-Bruneau Cancer Centre, CHU Sainte-Justine Research Centre, Montreal, Canada.
7.   Women’s College Hospital, Toronto, Canada.
8.   AYA Can-Canadian Cancer Advocacy, Winnipeg, Canada.
9.   University of Toronto, Toronto, Canada.
10. Max Rady College of Medicine, University of Manitoba, Winnipeg, Canada.
11. CancerCare Manitoba, Winnipeg, Canada.
12. Department of Oncology, Division of Psychosocial Oncology, Cumming School of Medicine, University of Calgary, Calgary, Canada.

### 100.1. Background/Rationale or Objectives/Purpose

Psychosocial distress is prevalent among children and adolescents during and after cancer therapy, yet no standardized evaluation exists. This study synthesizes literature on instruments for screening psychosocial distress in pediatric cancer care.

### 100.2. Methodology or Methods

This scoping review included English-language primary research published from 1990 to 2024, involving children and adolescents (0–18 years) diagnosed with cancer or survivors of childhood cancer. Studies from any geographic location and cancer care setting (e.g., hospital, clinic, home) were considered. Utilizing the Levac et al. (2010) framework, comprehensive searches were conducted in Medline, PubMed, and PsycINFO. Study selection followed the Population, Concept, Context (PCC) framework. Two reviewers independently screened titles and abstracts, and eligible full-text articles were further assessed in duplicate.

### 100.3. Impact on Practice or Results

Out of 8416 titles and abstracts and 1472 full-text articles screened, 496 studies met inclusion criteria. The review identified 410 unique instruments for psychosocial distress screening. The most frequently cited were the Pediatric Quality of Life Inventory (PedsQL), the Patient-Reported Outcomes Measurement Information System (PROMIS), and Memorial Symptom Assessment Scale (MSAS), accounting for 62.3% of studies (n = 309).

### 100.4. Discussion or Conclusions

There is an urgent need to integrate validated, clinically practical instruments for routine psychosocial distress screening in Canadian pediatric oncology settings. This scoping review maps existing instruments, evaluates their clinical applicability, and identifies optimal instruments for adoption. Implementing these evidence-based tools will enable timely detection of psychosocial distress, support targeted clinical interventions, and enhance both care quality and long-term psychological outcomes for pediatric cancer patients and survivors.

**173**

## 101. Enhancing the Human Experience: Implementation of a Novel Patient-Reported Experience Measures (PREMs) Program for Quality Improvement

Abigail Forbes, Alyssa Macedo, Shay Kittuppanantharajah, Mike Lovas, Janet Papadakos, Colleen Dunphy, Tran Truong, Meena Merali, Jennifer Catton, Neesha Dhani

Princess Margaret Cancer Centre, Toronto, Canada.

### 101.1. Background/Rationale or Objectives/Purpose

What happens when a Cancer Centre truly listens to what matters most to the patient? This abstract outlines an easy-to-implement step-wise process for actioning patient-reported experience feedback to advance patient experience.

### 101.2. Methodology or Methods

The Princess Margaret (PM) Patient and Family Experience Committee applied the Consolidated Framework for Implementation Research to implement an innovative Patient-Reported Experience Measures (PREMs) program inclusive of a novel role of a Patient Experience Quality Coordinator. Utilizing the Your Voice Matters survey, a validated, outpatient oncology patient experience measure, a dashboard was developed and real time data disseminated to leadership and local teams. Key performance indicators were developed, aligned to large scale change initiatives. Unique to PM, an open text question “What is one thing we could improve?” was asked and five recommendations were actioned monthly. Patients with poor experience were contacted for feedback to guide improvement initiatives.

### 101.3. Impact on Practice or Results

Over 100 large and small-scale quality improvement (QI) initiatives have been implemented since 2022. Our PREMs response rate increased from 1–2% to 30% (1100/3700) from 2020–2024. Our PREMs program was recognized as an Accreditation Canada Leading Practice. The overall patient experience improved from 74% to 83%; validation is underway.

### 101.4. Discussion or Conclusions

This landmark initiative driven solely by the patient voice places the human experience at the centre of QI initiatives to address holistic needs effectively, while focusing on sustainability and stewardship of resources. What resonates most? How have you advanced the patient experience? Are there other emerging trends or practices that should be considered in the development of a PREMs program?

**178**

## 102. Patient, Community and Health System Engagement to Inform the Development of a Framework for a Provincial Psychosocial Oncology Program

K. Julia Kaal ^1,2^, Jennifer Boone ^1^, Anahita Hollenhorst ^1^, Leslie Hill ^1^, Marianne Arab ^1^, Jill Flinn ^1^, Joy Tarasuk ^1^, Janice Howes ^1^, Mark Embrett ^3^, Logan Lawrence ^3^, Belinda Smith ^1^, Kenneth Jacquard ^1^, Allie Campbell ^1^, Helmut Hollenhorst ^1^, Archie Stewart ^1^

1. NSH Cancer Care Program, Halifax, Canada.
2. Dalhousie University, Halifax, Canada.
3. Nova Scotia Health, Halifax, Canada.

### 102.1. Background/Rationale or Objectives/Purpose

An embedded research project was conducted within the Nova Scotia Health Cancer Care Program to inform the development of a provincial psychosocial oncology program framework to organize and deliver psychosocial oncology (PSO) services across Nova Scotia. Two virtual workshops were held in July 2024 that engaged patients, community, and health system stakeholders and solicited feedback on the gathered evidence informing the framework.

### 102.2. Methodology or Methods

The workshops entailed a presentation on the background and context of the project and an overview of findings of the scoping literature review and stakeholder interviews. Attendees discussed the presentation before being assigned to two breakout rooms for topic-specific discussion regarding two factors that stood out in the gathered data. Breakout rooms followed the Nominal Group Technique (NGT), a structured method that facilitates equal participation, problem identification, solution generation and decision making.

### 102.3. Impact on Practice or Results

A total of 32 health system professionals and 11 patients attended the workshops. Statements generated in the breakout rooms were categorized by topic and included PSO support for family and/or caregivers, standard of care, PSO care across the cancer continuum, communication, equitable, diverse, inclusive, and accessible PSO care, program reporting structure and a provincial central PSO referral intake process.

### 102.4. Discussion or Conclusions

Learnings/results of the workshops are part of the evidence base that will directly inform the framework’s development. To support knowledge mobilization the workshop summary has been distributed to diverse groups/organizations who were not able to attend the workshop to ask for their feedback; a policy brief outlining the gathered evidence/resulting recommendations was presented at a CIHR Roundtable event. Collaborative framework development is ongoing.

**188**

## 103. Cost-Effectiveness of the Prostate Cancer Patient Empowerment Program (PC-PEP): A Health Promotion Strategy for Improving Outcomes in Prostate Cancer

Gabriela Ilie, Robert Rutledge

Dalhousie University, Halifax, Canada.

### 103.1. Background/Rationale or Objectives/Purpose

Prostate cancer is the most prevalent cancer among males in Canada, leading to significant side effects, mental distress, and increased healthcare costs. Health promotion strategies are essential for improving outcomes and managing these costs. The Prostate Cancer Patient Empowerment Program (PC-PEP) is a home-based intervention designed to reduce mental distress, enhance physical and urinary functions, and improve quality of life. This study evaluates the cost-effectiveness of PC-PEP, hypothesizing that its early implementation reduces healthcare spending and improves mental health and health-related quality of life (HRQoL).

### 103.2. Methodology or Methods

In a six-month crossover randomized trial, participants were randomized to the PC-PEP intervention or a waitlist control group. The PC-PEP program included daily activities such as stress reduction techniques, physical fitness routines, stress management strategies, pelvic floor exercises, healthy habit formation, intimacy training, social support, and dietary recommendations. Medical costs and health outcomes were assessed using data from Nova Scotia Medical Services Insurance and self-reported participant data. Incremental cost-effectiveness ratios (ICERs) were calculated, and bootstrapping was used to assess statistical uncertainty in the findings.

### 103.3. Impact on Practice or Results

#### 103.3.1. Results

PC-PEP demonstrated significant cost-effectiveness, improving mental health, modestly enhancing HRQoL, and reducing healthcare costs. Key findings include:

- At six months, PC-PEP saved 411.53 CAD per patient **and prevented** 30% of psychological distress cases requiring clinical treatment, **while improving** 0.0134 QALYs.
- At 12 months, cost savings increased **to** 660.89 CAD per patient, **preventing** 31% of psychological distress cases requiring clinical treatment, **and improving** 0.0344 QALYs.
- These results support the program’s substantial financial and psychosocial benefits.

#### 103.3.2. Impact on Practice

The findings of this study highlight PC-PEP as a scalable, evidence-based model for integrating health promotion into prostate cancer care. Its demonstrated cost-effectiveness supports broad implementation in clinical practice, particularly at the time of diagnosis, where early intervention can prevent distress escalation and improve quality of life. Policymakers and healthcare providers can benefit from adopting PC-PEP to optimize resource use, reduce healthcare costs, and improve patient outcomes. The program’s success underscores its potential to transform routine prostate cancer care globally, with opportunities for adaptation across diverse healthcare settings and patient populations.

### 103.4. Discussion or Conclusions

PC-PEP is a cost-effective, health-promoting intervention for prostate cancer patients. It significantly reduces healthcare costs, enhances mental health, and modestly improves HRQoL. The observed cost savings and health benefits highlight the importance of early implementation, advocating for the integration of PC-PEP into routine prostate cancer care. These findings have critical implications for healthcare policy, demonstrating PC-PEP’s potential to enhance patient outcomes and improve resource allocation in prostate cancer care globally.

## 104. Final Category: J. Palliative and End-of-Life Care

**30**

## 105. Tending to Soul: End-of-Life Practices with Integrative Yoga Therapy

Anne Pitman

The Centre for Health Innovation, Ottawa, Canada.

### 105.1. Background/Rationale or Objectives/Purpose

Long known for the practice of savasana (corpse pose), yoga has been subtly teaching us about impermanence, necessary endings, and dying at the conclusion of every yoga class. Yoga Therapy at end-of-life offers savasana as a central practice to foster wonder and wrestle with mortality. While evidence suggests yoga can shift patterns of anxiety, depression, fear and fatigue in late-stage disease, the tending to the soul is foremost when considering one’s dying time. An unhurried and accessible practice of subtle movement, meditation and soft breath work can help people release tension and pain, whilst cultivating acceptance, yield and surrender. Adaptable to diverse religious, spiritual or secular paths, yoga encourages engagement in the work of dying. Reflecting on Dharma, or our soul’s purpose, brings self-compassion, meaning and a tender humanity to the ending of days. Individualized yoga therapy works with the prevalent anxiety and abinevesha (fear of death) in palliative care and can assist palliative teams with non-pharmacologic and adjunctive practices for patients and their families. In this session, practice with corpse pose differently, and explore recent research that calls for an increased presence of yoga therapy in integrative palliative care, providing complementary care to those at the end-of-life, and deep support for our colleagues in palliative care.

### 105.2. Methodology or Methods

We will begin with a lecture on yoga therapy and dharma (soul’s purpose) at end of life, including research findings. We’ll then look at a few case studies of yoga therapy in integrative palliative care, common yoga practices at end-of life and finish with a practice of savasana (corpse pose).

### 105.3. Impact on Practice or Results

Yoga is practice. We can talk about research, theory and philosophy, but at some point, we must put it all down to relate to the soul through interoceptive practice.

### 105.4. Discussion or Conclusions

We’ll look at future direction on integrative yoga therapy for palliative care.

**83**

## 106. Rethinking the Management of Total Pain in Palliative Oncology Care: Toward an Integrative Approach

Emile Abou Chaar ^1,2^, Elaine Champagne ^3^, Sophie Lauzier ^1^, Mylène Brunet ^3,4^, Jacques Cherblanc ^5^, Pierre Gagnon ^6^, Thierry Collaud ^2^, Aline Hajj ^1,7^

1. Faculté de Pharmacie, Université Laval, Québec city, Canada.
2. Département de Théologie morale et d’Éthique, Université de Fribourg, Fribourg, Switzerland.
3. Faculté de théologie et de sciences religieuses, Université Laval, Québec city, Canada.
4. CSSanté, Québec city, Canada.
5. Unité d’enseignement en études religieuses, éthique et philosophie, Université du Québec à Chicoutimi, Chicoutimi, Canada.
6. Faculté de médecine, Université Laval, Québec city, Canada.
7. Institut National de Santé Publique, d’Épidémiologie Clinique et de Toxicologie-Liban (INSPECT-LB), Beirut, Lebanon.

### 106.1. Background/Rationale or Objectives/Purpose

This study aims to explore the lived experiences of patients with metastatic cancer, focusing on their capacities, coping strategies, and unmet needs in managing “total pain”. This concept, rooted in Cicely Saunders’ work, covers physical, psychological, social, and spiritual dimensions of suffering. Due to limited data in this area, this research aims to understand these multifaceted experiences and seeks to inform innovative and integrative strategies to alleviate suffering and enhance the quality of life in early palliative oncology care.

### 106.2. Methodology or Methods

This study was designed with patient-partners and will use a qualitative phenomenological design. We will recruit 20–30 adult patients diagnosed within three months with stage IV metastatic cancer from several hospitals affiliated with the CHU de Québec-Université Laval (Canada). Participants will be selected to capture diverse perspectives, and recruitment will continue until data saturation is achieved. Semi-structured interviews will explore patients’ experiences of total pain, and their views on integrative care approaches, involving pharmaceutical and spiritual interventions. A continuous thematic analysis assisted by NVivo will be conducted on the integral transcripts.

### 106.3. Impact on Practice or Results

Preliminary findings will shed light on how patients perceive and manage total pain. We expect key themes to include the interaction between the different dimensions of suffering, the role of coping strategies, and patient receptiveness to integrative pharmaceutical-spiritual care models.

### 106.4. Discussion or Conclusions

This research seeks to underscore the understanding of early palliative oncology care by integrating interdisciplinary pharmaceutical and spiritual strategies—a largely unexplored approach –, potentially challenging current practices and promoting personalized interventions to improve patient’s quality of life.

**84**

## 107. Students’ Perspectives on Socioeconomic Factors Affecting Cognitive Impairment in Advanced Cancer and Their Link to the Sustainable Development: Towards Promoting Sustainable Practice

Sarah Harb ^1^, Sophie Lauzier ^1^, Hermann Nabi ^2^, Aline Hajj ^1,3^

1. Faculté de Pharmacie, Université Laval, Québec city, Canada.
2. Faculty of Medicine, Université Laval, Québec city, Canada.
3. Institut National de Santé Publique, d’Épidémiologie Clinique et de Toxicologie-Liban (INSPECT-LB), Beirut, Lebanon.

### 107.1. Background/Rationale or Objectives/Purpose

Although socioeconomic factors play a critical role in cognitive impairment (CI) among patients with advanced cancer, research on university students’ understanding of these factors and their connection to sustainable healthcare remains limited. This study aimed to explore students’ perspectives on these determinants, aiming to address knowledge gaps and enhance awareness of their relevance to sustainable healthcare systems.

### 107.2. Methodology or Methods

Eligible participants were university-level students across Québec (Canada) conducting oncology-related research. Recruited through a convenience sampling via regional networks and student committees, participants completed an online questionnaire. This exploratory study included open- and closed-ended questions to examine participants’ understanding of CI, its links to socioeconomic status, key determinants, impacts on advanced cancer patients, perceptions of health inequities, and familiarity with Sustainable Development Goals (SDGs; scored 0–10) and sustainable healthcare. Data were thematically analyzed using NVivo.

### 107.3. Impact on Practice or Results

A total of 19 students took part in this study. Students acknowledged the socioeconomic impact of CI, primarily linking it to financial barriers and limited access to care. Their perceptions of CI’s consequences were framed within biomedical and psychosocial contexts, with minimal mention of employment or economic sustainability. Most believed CI could be integrated into the SDGs framework, primarily SDG3 (health and well-being), with little focus on SDG8 (employment and economic sustainability). The study highlighted gaps in students’ knowledge of sustainable health and SDGs (scores ranging from 0 to 5/10).

### 107.4. Discussion or Conclusions

Our findings revealed a limited understanding of sustainable development and health concepts among participants. This underscores the need to enhance awareness and education to promote holistic and sustainable approaches to patient care, especially for vulnerable populations such as patients with advanced cancer.

**119**

## 108. Patient Perceptions of Healthcare Worker Support During Medical Assistance in Dying: A Qualitative Study

Chunlin Liu ^1^, Eryn Tong ^2^, Sarah Hales ^2^, Rinat Nissim ^2^

1. Temerty Faculty of Medicine, University of Toronto, Toronto, Canada.
2. Department of Supportive Care, Princess Margaret Cancer Centre, University Health Network, Toronto, Canada.

### 108.1. Background/Rationale or Objectives/Purpose

Since the legalization of Medical Assistance in Dying (MAID), more Canadians have chosen MAID. To date, many studies have focused on HCWs’ attitudes towards MAID. However, little is known about patient’s perception of support from HCWs during MAID.

### 108.2. Methodology or Methods

Between February 2019 and June 2021, semi-structured interviews were conducted with 32 patients who requested MAID. Transcripts were thematically analyzed. Recurring themes were identified and developed iteratively by constantly comparing within and between transcripts.

### 108.3. Impact on Practice or Results

Three key themes emerged: (1) Communication and Information, highlighting the importance of clear, informative discussions on MAID and other end-of-life options. (2) Emotional Connectedness, demonstrating the patient’s need for HCWs to build trust, actively listen, and care with empathy. (3) Respect for Patient Autonomy, focusing on the need for HCWs to respect patient’s decision-making process.

### 108.4. Discussion or Conclusions

As more patients choose MAID, it is important to better understand clinical practices which patients value. HCWs should facilitate clear communication, build emotional connectedness, and respect the patient’s autonomy. These three themes can also inspire future policies, guidelines, and medical education to deliver better MAID care. Future research could explore the impact of HCW practices on family members’ perceptions of support during the MAID process.

**123**

## 109. What Led to the Introduction of Medical Assistance in Dying? Perspectives from Canadian Health Leaders

Amanda Yee ^1,2^, Eryn Tong ^2^, Rinat Nissim ^2,3^, Camilla Zimmermann ^2,3,4^, Sara Allin ^5^, Jennifer L. Gibson ^5,6^, Madeline Li ^2,3,4^, Gary Rodin ^2,3,4^, Gilla K. Shapiro ^2,3,4^

1. Leslie Dan Faculty of Pharmacy, University of Toronto, Toronto, Canada.
2. Department of Supportive Care, Princess Margaret Cancer Centre, Toronto, Canada.
3. Department of Psychiatry, Faculty of Medicine, Toronto, Canada.
4. Global Institute of Psychosocial, Palliative and End-of-Life Care (GIPPEC), University of Toronto and Princess Margaret Cancer Centre, Toronto, Canada.
5. Institute of Health Policy, Management and Evaluation, Dalla Lana School of Public Health, Toronto, Canada.
6. Joint Centre for Bioethics, Dalla Lana School of Public Health, University of Toronto, Toronto, Canada.

### 109.1. Background/Rationale or Objectives/Purpose

Cancer is the most common underlying medical condition among individuals who request and receive Medical Assistance in Dying (MAiD) in Canada. The legalization of MAiD in 2016 shaped healthcare practices around dying, but few research has examined its emergence in Canada. The purpose of this study was to explore health leaders’ perspective on the factors that led to the emergence of MAiD and on their attitudes towards MAiD.

### 109.2. Methodology or Methods

A qualitative descriptive study was conducted. We performed online semi-structured interviews with 36 Canadian health leaders from 2021 to 2022. We recruited participants using purposive and snowball sampling. Interviews were analyzed using inductive thematic analysis.

### 109.3. Impact on Practice or Results

Participants discussed six factors that they believed shaped the emergence of MAiD in Canada: public advocacy and influence; judicial system and notable MAiD legal cases; political ideology and landscape; policy diffusion; health system emphasis on a patient-centred care approach; and changes in societal and cultural values. Participants expressed wide-ranging attitudes on the legalization of MAiD. Some described overall agreement with the introduction of MAiD, while still raising concerns regarding vulnerability. Others held neutral attitudes and indicated that their attitudes changed on a case-by-case basis.

### 109.4. Discussion or Conclusions

This study highlights the wide-ranging and complex attitudes health leaders held towards MAiD and identifies the convergence of multiple factors that led to the legalization of MAiD in Canada. Ultimately, understanding health leaders’ attitudes and perspectives about the legalization of MAiD can guide policymakers in other countries considering assisted dying.

**150**

## 110. Exploring the Meaning of Research Participation for Bereaved Caregivers of Patients Who Died in Hospital During the COVID-19 Pandemic: A Qualitative Study

Thisha Ravindran ^1^, Junhee Baek ^1^, Sally Bean ^2^, Breffni Hannon ^1^, Elie Isenberg-Grzeda ^2^, Madeline Li ^1^, Debbie Selby ^2^, Camilla Zimmermann ^1^, Sarah Hales ^1^, Rinat Nissim ^1^

1. Princess Margaret Cancer Centre, Toronto, Canada.
2. Sunnybrook Health Sciences Centre, Toronto, Canada.

### 110.1. Background/Rationale or Objectives/Purpose

While motivations for research participation have been explored, little research examines its meaning for those who are grieving. In this exploratory study, we examine the significance of research participation for caregivers who lost family members during the COVID-19 pandemic, offering insights into how grief influences engagement with research.

### 110.2. Methodology or Methods

Sample and Setting: To ensure a diverse demographic representation, a subset of 29 interview transcripts were selected from a large mixed-method study with bereaved caregivers of patients who died in one of two large hospitals in Toronto, Canada, between March 2020 and May 2022.

Procedures: Transcripts were analyzed using grounded theory. Categories were created by conceptualizing meaning in the transcripts and repeated comparison with new and existing data.

### 110.3. Impact on Practice or Results

We identified two main themes: (1) advocacy for change, with subthemes of (a) the need for policy changes to support the bereavement process, and (b) moral duty; and (2) interviews as a therapeutic space, with subthemes of (a) emotional catharsis, and (b) empowerment.

### 110.4. Discussion or Conclusions

Our findings provide valuable insights for research design and conduct by highlighting how qualitative interviews can serve as both advocacy tools and therapeutic outlets for participants, particularly in the context of grief.

**160**

## 111. Intranasal Ketamine in Palliative Care: Secondary Outcome Analysis from the INKeD-PC Trial

Mary Makarious ^1^, Stefan Aguiar ^1^, Joshua D Rosenblat ^2^, Froukje deVries deVries ^3^, Zoe Doyle ^2^, Roger S. McIntyre ^2^, Gary Rodin ^1^, Camilla Zimmermann ^1^, Ernie Mak ^1^, Breffni hannon ^1^, Christian Schulz-Quach ^1^, Aida al Kindy ^1^, Zeal Patel ^1^, Madeline Li ^1^

1. Princess Margaret Cancer Centre, Toronto, Canada.
2. University Health Network, Toronto, Canada.
3. Netherlands Cancer Institute, Amsterdam, Netherlands.

### 111.1. Background/Rationale or Objectives/Purpose

The trial demonstrated strong efficacy in improving depression in patients with advanced cancer. Here we report secondary trial outcomes including changes in the Edmonton Symptom Assessment Scale-revised (ESAS-r), death anxiety (Death Anxiety and Distress Scale, DADDS), and quality of life (McGill Quality of Life, MQOL).

### 111.2. Methodology or Methods

The study involved 20 patients with advanced cancer recruited from oncology clinics at the Princess Margaret Cancer Centre. Secondary outcomes were available for n = 15 subjects.

Participants received three flexible doses of intranasal (IN) ketamine (50–150 mg) over a one-week period. Paired t-tests were conducted to assess differences between baseline and primary endpoint (Day 8), and Cohen’s d was used to evaluate effect size for each outcome. Qualitative feedback from participants confirms the reduction in death anxiety and improvement in well-being.

### 111.3. Impact on Practice or Results

IN ketamine significantly reduced ESAS-r physical symptoms (t(28) = 2.35, *p* = 0.013, Cohen’s d = 0.86) and emotional symptoms (t(28) = 2.39, *p* = 0.022, Cohen’s d = 0.88), as well as death anxiety (t(28) = 2.18, *p* = 0.019, Cohen’s d = 0.78). No significant change was observed in quality of life (MQOL, t(28) = 1.11, *p* = 0.277, Cohen’s d = 0.41), but the ESAS-r well-being item improved significantly (t(28) = 1.98, *p* = 0.029, Cohen’s d = 0.72).

### 111.4. Discussion or Conclusions

IN ketamine was associated with reductions in physical symptoms, emotional distress, death anxiety and well-being at the end of life. These findings suggest additional rapid benefits of treating depression with IN ketamine in patients with advanced cancer.

**171**

## 112. Hard Choices, Lots of Love: Exploring the Experiences of MAiD Family Caregivers for People Through Digital Storytelling

Michael Lang ^1,2,3^, Katherine Kortes-Miller ^4^

1. University of Calgary, Calgary, Canada.
2. Mike Lang Stories, Calgary, Canada.
3. Common Language Digital Storytelling, Calgary, Canada.
4. Lakehead University, Thunder Bay, Canada.

### 112.1. Background/Rationale or Objectives/Purpose

Over 60,000 Canadians have utilized Medical Assistance in Dying (MAID) since the passing of Bill C-14 in 2016. This means at least 400,000 Canadians have been directly impacted by MAID as close friends or family members. In most cases, these family caregivers bear both the logistical and emotional burden of these decisions made by their loved ones, even when receiving support from the healthcare system. Furthermore, the current cultural conversation concerning MAID is polarized, leading to fear, uncertainty, and experiences of stigmatization. Psychosocial Oncology professionals are well positioned to help family caregivers with the physical, mental, and emotional strain of caring for a loved one who chooses MAID.

### 112.2. Methodology or Methods

This presentation will begin by providing a very brief overview of the Digital Storytelling process and the SSHRC funded study that supported the creation of 12 digital stories with MAID Family Caregivers. It will then screen two digital stories with each followed by audience discussion about what psychosocial oncology professionals could take away from the stories and apply to their own clinical practice.

### 112.3. Impact on Practice or Results

Attendees will leave with a greater understanding of the lived experiences of MAID family caregivers and have direct access to digital stories that could be utilized in their own clinical practice with this population.

### 112.4. Discussion or Conclusions

Supporting family caregivers of people with cancer is a core role of psychosocial oncology professionals so that entire family systems can live well with, through, and beyond the cancer experience. This includes family caregivers of cancer patients who choose MAID. As the number of MAID family caregivers will only increase in the future, and the experience of supporting a loved one through MAID is logistically, mentally, and emotionally complex, psychosocial oncology professionals need to develop a deeper understanding of this population’s lived experience so that they can provide the most meaningful and effective care.

**174**

## 113. Conceptualizing Quality of Life for Adolescents Living with Advanced Cancer

Andrea Johnson ^1,2^, Kimberley Widger ^1,2^

1. University of Toronto, Toronto, Canada.
2. The Hospital for Sick Children, Toronto, Canada.

### 113.1. Background/Rationale or Objectives/Purpose

Approximately 25% of adolescents diagnosed with cancer will live with advanced cancer that is unlikely to be cured. Although Quality of Life (QoL) is considered at different times in the cancer trajectory for adolescents, it becomes even more significant when adolescents are living with advanced cancer and accessing palliative care. The dominant goal of palliative care is to enhance QoL however, QoL has been poorly explored for adolescents living with advanced cancer and there is no established conceptual framework of QoL guiding their clinical care and interventions. The purpose of this research is to develop a conceptual framework of QoL that is valid and meaningful to adolescents with advanced cancer.

### 113.2. Methodology or Methods

This study has two phases. Initially a qualitative study guided by the methodological principles of Interpretive Description was conducted with social media data created by adolescents living with advanced cancer. TikTok and other social media sources were searched to identify relevant posts using search strings such as: adolescent + terminal cancer. All posts created by adolescents, ages 13–19, who self-identified as living with advanced cancer, were included in the analysis. Data was coded and iteratively analyzed into proposed conceptual domains of QoL. The second phase of this study involved the validation of our proposed conceptual QoL domains and development of a conceptual framework. A second qualitative study was conducted using interviews with adolescents living with advanced cancer who offered insights and feedback on our proposed domains of QoL and more broadly shared their experiences living with advanced cancer and how they considered their QoL. Interview data was analyzed in accordance with Interpretive Description and used to refine our proposed domains of QoL. Throughout both study phases, we consulted with an adolescent young adult (AYA) Research Advisory group of AYAs recently treated for cancer that we developed as part of this research.

### 113.3. Impact on Practice or Results

In the initial study phase, social media data from 14 adolescents living with advanced cancer were included and 235 posts that they created were analyzed. This sample included adolescents with a variety of cancers and had diverse geographical representation. The second study phase involved 10 adolescents also with a variety of cancer diagnoses and were from Toronto and Vancouver. Our analyses from both phases generated a conceptual framework of QoL for adolescents with advanced cancer including the following domains: (1) Perceived Health; (2) The Lived Body; (3) Emotional Wellbeing; (4) Normalcy; (5) Purpose and Direction; (6) Re-Orientation.

### 113.4. Discussion or Conclusions

Enhancing QoL is a dominant goal of the clinical care of adolescents with cancer when curative treatment has not been successful and a valid conceptual framework of QoL is an essential precursor to this goal. Our research has generated a conceptual framework of QoL meaningful to adolescents with advanced cancer informed by their voices and experiences. This framework contains conceptual domains markedly different from those of QoL frameworks traditionally used with adolescents with advanced cancer but not validated with them. This reflects a significant gap between what is usually measured for QoL of adolescents with advanced cancer and what is important to them from their perspectives. Our findings offer a valid conceptualization of QoL that can be used to guide clinical care and as the basis for development of a tool to assess QoL of adolescents with advanced cancer.

**182**

## 114. The Wish to Hasten Death Is Distinct from the Desire for Medical Assistance in Dying (MAiD)

Eugenia Ubeira Amaral ^1^, Stefan Aguiar ^1,2^, Aliza Panjwani ^1,2^, Gilla Shapiro ^1,2,3^, Anne Rydall ^1^, Emma Hagopian ^1^, Roberta Yael Klein ^1^, Anne Barbeau ^1^, Gary Rodin ^1,2,3^, Madeline Li ^1,2^

1. Princess Margaret Cancer Centre, Toronto, Canada.
2. University of Toronto, Toronto, Canada.
3. Global Institute of Psychosocial, Palliative and End-of-Life Care, Toronto, Canada.

### 114.1. Background/Rationale or Objectives/Purpose

The prevalence of the wish to hasten death (WTHD) in patients with advanced cancer ranges from 2–28%. While the desire for MAiD ranges between 2.5–10%, there is little evidence regarding the prevalence of the WTHD in patients with advanced cancer who request MAiD, this study aims to describe the prevalence of the WTHD in patients with advanced cancer, focusing on those who requested MAiD.

### 114.2. Methodology or Methods

We conducted a prospective, longitudinal study of MAiD in patients with advanced cancer. Patients were classified according to whether they would consider requesting/completing MAiD. The WTHD was measured by the Schedule of Attitudes toward Hastened Death (SAHD-A), with a cutoff-value of ≥3 signifying a high vs. low WTHD.

### 114.3. Impact on Practice or Results

There were 317 patients at baseline with a mean SAHD-A score of 0.67 (SD = 1.03), 14 of whom reported an SAHD-A ≥ 3 (4.4%). SAHD-A scores were positively associated with MAiD interest, from patients who would not consider MAiD (M = 0.38, SD = 0.53), patients who were unsure (M = 0.46, SD = 0.76), to patients who would consider MAiD (M = 1.02, SD = 1.31). 35 patients (11.3%) subsequently requested MAiD during follow-up, with 12 (3.8%) completions to date. Mean SAHD-A scores were 1.30 (SD = 1.63) and1.45 (SD = 1.81), respectively. 5/35 MAiD requests (14.3%) and 2/12 MAiD completions (16.7%) reported high SAHD-A scores.

### 114.4. Discussion or Conclusions

SAHD-A scores are higher in patients with interest in MAiD, but the WTHD remains low in most patients who request and complete MAiD. This suggests that there are many psychological states within the WTHD potentially amenable to interventions other than MAiD.

**184**

## 115. Death Anxiety in Patients with Advanced Cancer Requesting MAiD

Stefan Aguiar ^1,2^, Eugenia Amaral ^1^, Aliza. A Panjwani ^1,2^, Gilla K. Shapiro ^1,2,3^, Emma Hagopian ^1^, Robin Graham ^1^, Roberta Y. Klein ^1^, Anne Barbeau ^1^, Anne Rydall ^1^, Gary Rodin ^1,2,3^, Madeline Li ^1,2^

1. Princess Margaret Cancer Centre—University Health Network, Toronto, Canada.
2. University of Toronto, Toronto, Canada.
3. Global Institute of Psychosocial, Palliative and End-of-Life Care (GIPPEC), Toronto, Canada.

### 115.1. Background/Rationale or Objectives/Purpose

Advanced cancer is the most common condition underlying requests for medical assistance in dying (MAiD), but the impact of psychological factors such as death anxiety has not been previously explored. This study describes the prevalence of death anxiety and its relationships to other distress variables.

### 115.2. Methodology or Methods

Descriptive data are presented from a longitudinal study on the desire for MAiD in patients with advanced cancer. The sample comprised 279 patients at baseline and 35 that requested MAiD, of which 12 received MAiD. Pearson correlations were calculated between death anxiety (Death and Dying Distress Scale [DADDS]) and self-perceived burden (Self Perceived Burden Scale [SPBS]), depressive symptoms (Patient Health Questionnaire-9, [PHQ-9]), and symptom burden (Edmonton Symptom Assessment Scale-revised [ESAS-r]).

### 115.3. Impact on Practice or Results

The prevalence of mild, moderate and severe death anxiety across groups were 67%, 28%, and 5% at baseline; 55%, 42%, and 3% at MAiD request; and 73%, 27%, and 0% among the subset of MAiD recipients. The DADDS was significantly positively correlated (*p* ≤ 0.01) with SPBS (r = 0.425), PHQ-9 (r = 0.527), and ESAS-r (r = 0.547) in the baseline sample. Among MAiD requestors, DADDS correlated with only SPBS (r = 0.572).

### 115.4. Discussion or Conclusions

Moderate to severe death anxiety is higher in patients who request MAiD, but lower among MAiD recipients. Death anxiety is associated with various distress outcomes among individuals with advanced cancer and those requesting MAiD, but not among those who receive MAiD. Findings may assist clinicians in recognizing psychological suffering in patients considering MAiD to support informed decision-making.

**195**

## 116. Communication Is Key: A Qualitative Study on the Experience of Medical Assistance in Dying (MAiD) Communication for Patients with Advanced Cancer

Olivia Mazzurco ^1,2^, Anne Barbeau ^2^, Anne Rydall ^2^, Emma Hagopian ^2^, Gary Rodin ^1,2^, Rinat Nissim ^2,1^, Roberta Y. Klein ^2^, Madeline Li ^2,1^

1. University of Toronto, Toronto, Canada.
2. Princess Margaret Cancer Centre, University Health Network, Toronto, Canada.
3. Global Institute of Psychosocial, Palliative and End-of-Life Care (GIPPEC), University of Toronto, Toronto, Canada.

### 116.1. Background/Rationale or Objectives/Purpose

Canada’s legalization of Medical Assistance in Dying (MAiD) offered a new end-of-life option. Yet, clinical communication guidelines are largely not evidence-based, and patient conversations throughout the MAiD decision-making trajectory remain relatively unknown. This study aimed to better understand the patient experience of MAiD communication with their healthcare providers (HCPs) and family members and conceptualize the nature of these conversations.

### 116.2. Methodology or Methods

Twenty patients with advanced cancer from a Canadian comprehensive cancer centre, of which 8 had applied for MAiD, participated via virtual or phone semi-structured interviews. Qualitative interviews included patients across a range of attitudes towards MAiD. Open-ended questions focused on MAiD understanding, communication, and the decision-making process, along with access to supportive care services and healthcare team relationships. Interview transcripts were independently coded by two researchers, then reconciled and analyzed using constructivist grounded theory methodology.

### 116.3. Impact on Practice or Results

The core category “Experiencing the complexities and ambiguities of MAiD communication” symbolizes its dynamic and uncertain nature, where patients are questioning and planning MAiD conversations. The experience is detailed across four subcategories representing the Four W’s: WHAT are the end-of-life options, WHO to discuss MAiD with, WHEN are MAiD conversations initiated with HCPs, and WHY discuss a patient’s reasons for MAiD.

### 116.4. Discussion or Conclusions

MAiD is one part of end-of-life communication, requiring information and understanding. HCP-initiated communication with patients is to involve informative, personalized and nuanced therapeutic conversations to psychologically unpack everything that goes into an end-of-life decision. Findings may inform future MAiD communication protocols and support optimal end-of-life care for patients.

## 117. Final Category: K. Pandemics and Cancer Care Issues

**6**

## 118. Healthcare Providers’ Knowledge, Needs, and Experiences with Self-Management Support for Cancer-Related Cognitive Impairment: An Ongoing Qualitative Study

Sitara Sharma, Jennifer Brunet

University of Ottawa, Ottawa, Canada

### 118.1. Background/Rationale or Objectives/Purpose

In the absence of established treatment options for cancer-related cognitive impairment (CRCI), self-management (SM) becomes crucial. Healthcare providers (HCPs) are well-positioned to facilitate SM, but insufficient CRCI training can limit their ability to support patients experiencing cognitive challenges. Within a larger research program, this qualitative study aims to explore HCPs’: (1) knowledge and perceptions of CRCI guidelines, treatment gaps, and emerging SM strategies (e.g., physical activity), (2) needs for delivering effective CRCI SM support, and (3) barriers/facilitators to adopting SM support interventions.

### 118.2. Methodology or Methods

We will recruit 10–15 Ontario-based oncology HCPs via purposive and snowball sampling until data saturation. Participants will complete an online survey and semi-structured interview guided by the *Theoretical Domains Framework* (TDF) to explore their knowledge, perceptions, needs, barriers, and facilitators related to CRCI SM support. Descriptive statistics will be generated for survey data; interviews will be audio-recorded, transcribed, and analyzed using hybrid deductive-inductive content analysis to code data within TDF domains and identify emergent themes, respectively. Preliminary themes will be reviewed by an Advisory Council comprising HCP and patient representatives and refined accordingly. We welcome feedback/tips on: (1) determining data saturation/trustworthiness, (2) analyzing/visualizing data using the TDF, and (3) meaningfully engaging our Advisory Council throughout/beyond this study.

### 118.3. Impact on Practice or Results

N/A—protocol

### 118.4. Discussion or Conclusions

Insights into HCPs’ CRCI knowledge gaps and SM support delivery needs, barriers, and facilitators will shape future CRCI-focused educational resources/interventions for HCPs. Specifically, findings will pinpoint essential topics for inclusion, guide optimal resource/intervention design, and illuminate which behaviour change techniques should be targeted.

**102**

## 119. Exploring the Psychological Health of Parents of Children with Cancer During the COVID-19 Pandemic: A Mixed Methods Study

Alex Pizzo ^1^, Claire R. Galvin ^1^, Kelly Mazzocca ^1^, Elham Hashemi ^2^, Maxime Caru ^3^, Thomas Hadjistavropoulos ^4^, Paul C. Nathan ^2^, Serge Sultan ^5^, Lindsay Jibb ^2^, Nicole M. Alberts ^1^

1. Concordia University, Montréal, Canada.
2. Hospital for Sick Children, Toronto, Canada.
3. Penn State College of Medicine, Hershey, USA.
4. University of Regina, Regina, Canada.
5. Université de Montréal, Montréal, Canada.

### 119.1. Background/Rationale or Objectives/Purpose

The COVID-19 pandemic caused unprecedented challenges for parents in the general population, which may have been compounded in families managing childhood cancer. Despite this, few studies have examined the psychological health of parents of children with cancer within the context of the pandemic.

### 119.2. Methodology or Methods

Canadian parents of youth on cancer treatment during the pandemic (N = 30; age = 41.3 years, 86.7% mothers) and now off treatment, were recruited from the community. Parents completed measures of anxiety (Generalized Anxiety Disorder-7) and depressive symptoms (Patient Health Questionnaire-8) as well as a semi-structured interview regarding their perceptions of psychological health and services offered during the pandemic. Interviews were transcribed and analyzed using thematic analysis. The proportion of parents with elevated levels (score ≥ 10) of anxiety, depressive symptoms, or both were calculated.

### 119.3. Impact on Practice or Results

We derived five themes: (1) uncertainty around the primary cause of distress (pandemic vs. cancer), (2) exacerbation of negative psychological effects of cancer treatment, (3) reduction in already limited mental health supports, (4) positive and negative experiences of virtual psychological services, and (5) resilience against the long-term impact of the pandemic on psychological health. Moreover, 26.7%, 36.7%, and 23.3% endorsed elevated levels of anxiety, depressive symptoms, and both, respectively.

### 119.4. Discussion or Conclusions

Parents described the pandemic as amplifying distress associated with their child’s cancer, and experiencing limited access to support services. Study findings provide insights into service needs and delivery mode preferences for those involved in the psychosocial care of families impacted by cancer, and can improve readiness for future pandemics or major incidents.

**142**

## 120. Impact of Hospital Visitor Restrictions During the COVID-19 Pandemic on Quality of Death and Bereavement Outcomes

Rinat Nissim ^1^, Paige Chu ^1^, Sally Bean ^2^, Breffni Hannon ^1^, Elie Isenberg-Grzeda ^2^, Madeline Li ^1^, Debbie Selby ^2^, Camilla Zimmermann ^1^, Sarah Hales ^1^

1. Princess Margaret Cancer Centre, Toronto, Canada.
2. Sunnybrook Health Sciences Centre, Toronto, Canada.

### 120.1. Background/Rationale or Objectives/Purpose

The COVID-19 pandemic and institutional control measures have changed how people die and how their families grieve. This presentation will report on quality of death (QOD) outcomes of individuals dying in hospital during the pandemic; bereavement outcomes of their family caregivers; and the impact of pandemic control measures on outcomes.

### 120.2. Methodology or Methods

Sample and setting: **Retrospective lists of patient deaths between March 2020 and May 2022 in two large urban** hospitals in Toronto, Ontario, Canada were used to identify and recruit bereaved family members.

Procedures: Bereaved caregivers were approached for recruitment at least six months following patient death. Consenting participants completed self-report questionnaires via REDCap, including the PG-13 and TRIG (complicated grief symptoms), IES-R (PTSD symptoms), PHQ-9 (depressive symptoms), FAMCARE (satisfaction with care), and a survey on perceived pandemic restrictions (pre- and post-death). QOD was assessed using the QODD questionnaire, administered via video or phone interviews.

### 120.3. Impact on Practice or Results

A total of 286 bereaved family members completed study measures (mean age 62.3 years; 66% women; 59% spousal caregivers). Higher levels of pre-death hospital visitor restrictions were significantly associated with lower quality of death (QOD) and satisfaction with care (*p* < 0.001). Both pre- and post-death visitor restrictions showed significant positive correlations with symptoms of depression, post-traumatic stress disorder (PTSD), and complicated grief (all *p* < 0.001).

### 120.4. Discussion or Conclusions

These findings highlight the profound effects of the pandemic and its containment measures on end-of-life and bereavement outcomes. They provide evidence to inform interventions, research, and policy development to better support families during pandemics and similar crises.

**146**

## 121. Bereaved Caregiver Perspectives on COVID-19 Restrictions: A Thematic Analysis of Open-Ended Survey Responses

Karley Wulf ^1^, Paige Chu ^1^, Sally Bean ^2^, Breffni Hannon ^1^, Elie Isenberg-Grzeda ^2^, Madeline Li ^1^, Debbie Selby ^2^, Camilla Zimmermann ^1^, Sarah Hales ^1^, Rinat Nissim ^1^

1. Princess Margaret Cancer Centre, Toronto, Canada.
2. Sunnybrook Health Sciences Centre, Toronto, Canada.

### 121.1. Background/Rationale or Objectives/Purpose

This study explored bereaved caregivers’ perspectives on COVID-19 restrictions, focusing on how these policies influenced their caregiving and grief experiences during and after a family member’s end-of-life hospitalization.

### 121.2. Methodology or Methods

Sample and Setting: Data were collected from 286 bereaved caregivers of patients who died in one of two large hospitals in Toronto, Canada, between March 2020 and May 2022. Participants were asked to rate their perceived level of restrictions. In addition, two open-ended questions invited participants to share their thoughts on COVID-19 restrictions and provide suggestions for improving care during the pandemic.

Procedures: Of the 286 participants, 244 responded to the open-ended questions. The responses were analyzed using reflexive thematic analysis.

### 121.3. Impact on Practice or Results

The analysis identified key challenges and suggestions for improvement across several areas: Communication of Hospital Restrictions, Emotional Support, Enforcement of Policies, Physical Care and Advocacy, Last Moments and Farewells, Autopsy and Aftercare Transparency, Disruptions to Culturally and Spiritually Significant Grief Rituals, and Post-Death Support.

### 121.4. Discussion or Conclusions

Caregivers reported a profound impact of COVID-19 policies on their experience during end-of-life care and bereavement. The study’s findings can inform the development of more caregiver-centered policies and practices, particularly during public health crises. Additionally, insights can guide future survey designs to better capture the experiences and needs of bereaved caregivers.

**149**

## 122. Selfless Caregivers: A Qualitative Study of Woman Caregivers with Young Children Supporting a Family Member at the End of Life During the COVID-19 Pandemic

Junhee Baek ^1^, Faye Ajmera ^1^, Sally Bean ^2^, Breffni Hannon ^3^, Elie Isenberg-Grzeda ^2^, Madeline Li ^3^, Debbie Selby ^2^, Camilla Zimmermann ^3^, Sarah Hales ^3^, Rinat Nissim ^3^

1. University of Toronto, Toronto, Canada.
2. Sunnybrook Health Sciences Centre, Toronto, Canada.
3. Princess Margaret Cancer Centre, Toronto, Canada.

### 122.1. Background/Rationale or Objectives/Purpose

More than half of women in Canada provide care to both children and care-dependent adults. During the COVID-19 pandemic, the challenges of this dual caregiving role were heightened for many women, compounded further by the loss of loved ones and the experience of grieving in isolation due to social distancing measures. This qualitative study aims to understand the lived experience of woman caregivers who cared for their children and family members dying in hospitals during the pandemic.

### 122.2. Methodology or Methods

Sample and Setting: Using purposive sampling, 11 transcripts from woman caregivers with children under the age of 18 were identified from a large mixed-method study with 286 bereaved caregivers of patients who died in one of two large hospitals in Toronto, Canada, between March 2020 and May 2022.

Procedures: Transcripts were analyzed using grounded theory. Categories were created by conceptualizing meaning in the transcripts and repeated comparison with new and existing data.

### 122.3. Impact on Practice or Results

We identified a core category: selfless caregiver. Six subcategories emerged: being “pulled in many different directions”; self-care as “homework”; lacking a “shoulder to cry on”; the impact of the death on children as “a difficult pill to swallow”; prioritizing others’ needs over self; and “saved” by children from feelings of grief. COVID-19 as an added burden was identified as a cross-cutting theme.

### 122.4. Discussion or Conclusions

The findings emphasize the need for family-centered clinical practices and policies supporting dual caregiving role and tailored bereavement programs. Recognizing the intersection of caregiving and parenting is essential to developing equitable and effective support systems for families, particularly during public health crises.

**181**

## 123. Impact of Loneliness on Cancer Mortality: A Systematic Review and Meta-Analysis

Samantha Cheng ^1^, Joshua Yi ^1^, Keiran Pace ^2^, Jane Jomy ^1^, Srinivas Raman ^3^

1. University of Toronto, Toronto, Canada.
2. Toronto, Toronto, Canada.
3. BC Cancer, Vancouver, Canada.

### 123.1. Background/Rationale or Objectives/Purpose

This study aims to evaluate the association between loneliness and social isolation on mortality among cancer patients.

### 123.2. Methodology or Methods

We searched MEDLINE, Embase, and PsycINFO from inception to 13 September 2024 for articles that report on the impact of loneliness and/or social isolation on all-cause and cancer-related mortality among cancer patients. Two pairs of reviewers independently screened articles resolving discrepancies by consensus or involvement of a third reviewer. Following a data calibration exercise, data on study characteristics, loneliness measures, and mortality outcomes were extracted using a standardized form. Risk of bias was assessed using a tool by the CLARITY Group at McMaster University. We plan to conduct random-effects meta-analyses to synthesize the impact of loneliness and social isolation on mortality, supplemented by subgroup analyses for risk of bias where possible. Quality of evidence will be appraised using the GRADE approach.

### 123.3. Impact on Practice or Results

Of 12,602 citations, 17 publications were included and reported on over 80,000 patients with various oncologic diagnoses including colorectal, head and neck, breast, prostate, and lung cancers. Of these, most articles focused on the concepts of social isolation, integration, and deprivation, rather than loneliness. Many articles establish a statistically significant association between social isolation and all-cause mortality or cancer-related mortality. All formal meta-analyses are currently underway, and the results will be made available in advance of CAPO 2025.

### 123.4. Discussion or Conclusions

This review aims to inform clinicians and policymakers about the implications of loneliness for cancer outcomes, promoting interventions to mitigate its effects on survival.

## 124. Final Category: L. Patient Oriented Research Approaches

**1**

## 125. Culturally Responsive Policies and Impact of Cancer on Health-Related Quality of Life of Adults Living with Cancer in British Columbia

Melba D’Souza ^1^, Ruby Gidda ^2^, Ashwin Nairy ^3^

1. Thompson Rivers University, British Columbia, Canada.
2. BC Cancer Abbotsford & Provincial Professional Practice, British Columbia, Canada.
3. University of British Columbia, British Columbia, Canada.

### 125.1. Background/Rationale or Objectives/Purpose

Cancer impacts the economic stability and health-related quality of life of adults in British Columbia, particularly in rural areas.

### 125.2. Methodology or Methods

The goal of this research is to understand the economic burden of cancer and its effect on the quality of life among adults living with cancer in British Columbia’s interior region. A quantitative descriptive cross-sectional research design was used for the study.

### 125.3. Impact on Practice or Results

The findings reveal that gender and cancer type demonstrate significant impacts on both the economic burden of cancer (EBC) and cancer treatment barriers (CTB) across all tests, featuring their prominent roles in patient outcomes. Age, ethnicity, education, residential location, employment and time since diagnosis also showed significant effects on CTB, with chi-square tests indicating disparities in treatment access. ANOVA Tests revealed that being a middle-aged, male, ethnic minority, college graduate, residing in a remote area, employed part-time, having a type of cancer other than breast cancer, and having been diagnosed for less than five years are associated with higher barriers in cancer treatment.

### 125.4. Discussion or Conclusions

This study highlights the complexity of factors affecting cancer care and economic outcomes, suggesting that interventions should be customized to address the distinct needs of different groups. This study highlights the need for targeted, culturally responsive policies that address the unique challenges faced by people treated with cancer in British Columbia, especially in rural communities. Enhancing supportive care services and addressing socioeconomic disparities is crucial for improving outcomes and quality of life. The insights gained from this research can guide future interventions to better support cancer across diverse demographic and socioeconomic backgrounds.

**9**

## 126. Assessing Quality-of-Life in Young People with Advanced Cancer: Usability and Social Validity of an Electronic Self-Report Questionnaire

Lye-Ann Robichaud ^1,2^, Nikita Guarascio ^1,2^, Michel Duval ^3,4^, Bruno Michon ^5^, Marianne Olivier-D’Avignon ^6^, Mathias Tyo-Gomez ^7^, Émélie Rondeau ^2,7^, Marc-Antoine Marquis ^3,8^, Serge Sultan ^1,2,3^

1. Department of Psychology, Université de Montréal, Montreal, Canada.
2. Azrieli Research Centre, CHU Sainte-Justine, Montreal, Canada.
3. Department of Pediatrics, Université de Montréal, Montreal, Canada.
4. Department of Hematology-Oncology, CHU Sainte-Justine, Montreal, Canada.
5. Centre mère-enfant Soleil, CHU de Québec-Université Laval, Quebec, Canada.
6. School of Social Work, Université Laval, Quebec, Canada.
7. Psycho-Oncology Center (CPO), CHU Sainte-Justine, Montreal, Canada.
8. Department of General Pediatrics, CHU Sainte-Justine, Montreal, Canada.

### 126.1. Background/Rationale or Objectives/Purpose

Self-reported questionnaires assessing quality of life (QoL) are essential for tailoring interventions to young people with advanced cancer. Two paper-and-pencil questionnaires for ages 8–12 and 13–18, covering seven QoL domains (physical, psychological, social, pleasure, autonomy, achievements, and feeling heard) were developed. This study adapted these questionnaires into electronic versions using the REDCap^®^ platform to enhance accessibility and usability.

### 126.2. Methodology or Methods

We recruited 20 French-speaking patients (10 children aged 8–12 and 10 adolescents aged 13–18) from CHU Sainte-Justine and with Leucan, a non-profit organization. Participants, diagnosed with cancer at least three months prior, were undergoing treatment, spoke French, could communicate verbally, and had an Internet-connected device.

Recruitment and data collection occurred in two rounds, with questionnaires’ revisions between each round to ensure problem coverage. Participants tested the electronic questionnaires and evaluated their social validity (acceptability, relevance, and satisfaction) through virtual cognitive interviews. They also completed a short sociodemographic questionnaire. Interviews were analyzed using a method for interpreting cognitive interviews for instrument development.

### 126.3. Impact on Practice or Results

We identified 22 codes from cognitive interviews, organized into five themes: (1) Content-related elements; (2) Format-related elements; (3) Technology-related elements; (4) Positive features; and (5) Children-specific elements. These insights informed modifications to optimize the electronic version.

### 126.4. Discussion or Conclusions

*Advance QoL* tools aim to empower young patients by including their voices in QoL discussions. Overall feedback on the electronic versions was positive, with minor issues addressed via REDCap^®^ functionalities. These tools were found to be accessible, user-friendly, and relevant, offering valuable means to gather self-reported data and helping identify intervention targets.

**13**

## 127. The Public Interest Group on Cancer Research—2025 Update

Sevtap Savas ^1,2^, John King ^3^, Deanna Roy ^1^, Derrick Bishop ^4^, Janine Taylor-Cutting ^5^, Darrell Peddle ^1^, Namiko O’Brien ^6^, Holly Etchegary ^1,2^

1. Public Interest Group on Cancer Research, St. John’s, Canada.
2. Memorial University of Newfoundland, St. John’s, Canada.
3. Public Interest Group on Cancer Research, Clarenville, Canada.
4. Public Interest Group on Cancer Research, CBS, Canada.
5. Public Interest Group on Cancer Research, Grand Falls-Windsor, Canada.
6. Public Interest Group on Cancer Research, Portugal Cove—St. Philips, Canada.

### 127.1. Background/Rationale or Objectives/Purpose

Background and rationale: Public and patient engagement greatly influences clinical care, health literacy, research, and policy-making. Public Interest Group on Cancer Research is a public—scientist partnership focusing on cancer in Newfoundland and Labrador. Created in 2021, this group has been instrumental in identifying patient and family priorities, disseminating public knowledge on cancer, organizing public events, working on a public outreach strategy, and advocacy. Our goal in this presentation is to review and summarize our activities to date.

### 127.2. Methodology or Methods

Methods: We reviewed our meeting minutes, project reports, and other documentation and correspondences related to our work up to November 2024.

### 127.3. Impact on Practice or Results

Results/impact on practice: Since 2021, we have published three academic papers; delivered several conference, webinar, and public presentations; published articles in local media; organized and delivered two public events; instituted a podcast; helped train graduate students; advocated for individuals and families affected by cancer; co-developed projects and obtained funding; and increased our public outreach in several ways. We also received the university’s public engagement award.

### 127.4. Discussion or Conclusions

Discussion: The Public Interest Group on Cancer Research is a productive public—scientist partnership that has initiated and accomplished various academic and public activities about cancer in Newfoundland and Labrador. Strengthened by its members’ dedication, lived experience, creativity, and skills, this group aims to continue to elevate the voices of individuals affected by cancer and improve their circumstances through advocacy, community engagement, and research. This group’s work and accomplishments can inform other patient partnerships in oncology.

**15**

## 128. ACC PAC: A Pan-Atlantic Canada Patient and Family Advisory Committee for Precision Medicine and Cancer

Sevtap Savas ^1,2^, Georgia Skardasi ^1,2^, Aaron Curtis ^1,2^, John King ^3^, Jennifer Coish ^4^, Sharon Jennings ^5^, Marcelo Fabian Martinez ^6^, Cyndi Corbett ^7^, Cara MacInnis ^8^, Judy Donovan Whitty ^6^, Beverly Pausche ^9^, Gilles LeBlanc ^10^, Pauline McIntyre ^11^, Angela Hyde ^1,12^

1.   Atlantic Cancer Consortium Patient Advisory Committee, St. John’s, Canada.
2.   Memorial University of Newfoundland, St. John’s, Canada.
3.   Atlantic Cancer Consortium Patient Advisory Committee, Clarenville, Canada.
4.   Atlantic Cancer Consortium Patient Advisory Committee, Mount Pearl, Canada.
5.   Atlantic Cancer Consortium Patient Advisory Committee, Souris, Canada.
6.   Atlantic Cancer Consortium Patient Advisory Committee, Charlottetown, Canada.
7.   Atlantic Cancer Consortium Patient Advisory Committee, Windsor Forks, Canada.
8.   Atlantic Cancer Consortium Patient Advisory Committee, Coldbrook, Canada.
9.   Atlantic Cancer Consortium Patient Advisory Committee, Gaspereau, Canada.
10. Atlantic Cancer Consortium Patient Advisory Committee, Dieppe, Canada.
11. Atlantic Cancer Consortium Patient Advisory Committee, Fredericton, Canada.
12. Newfoundland and Labrador Health Services, St. John’s, Canada.

### 128.1. Background/Rationale or Objectives/Purpose

Precision Medicine can help improve treatment and survival outcomes of individuals diagnosed with cancer. Here we describe the formation and work of the ACC PAC, a pan-Atlantic Canada Patient Advisory Committee created as part of the Atlantic Cancer Consortium (ACC) in order to advise the ACC researchers on Precision Medicine-related priorities, research and public engagement activities.

### 128.2. Methodology or Methods

The ACC PAC members were recruited in early 2024 using social and mainstream media, institutional communications, and connections. Forty-two applicants completed a demographic survey to help select the final 12 public members with diverse backgrounds. The committee consisting of public members, a cancer scientist, an oncologist, and a coordinator meets virtually; meeting minutes taken were used to summarize the work described here.

### 128.3. Impact on Practice or Results

The ACC PAC members represent patients and family members, several cancers, visible minorities, and all four Atlantic Canada provinces. So far, the ACC PAC identified cancer patient and family priorities in Atlantic Canada. These priorities concentrated on two key topics: (1) how to effectively prevent cancer and provide care for all who are affected by cancer, and (2) how to better understand and access Precision Medicine. The ACC PAC is currently working on organizing a public conference on Precision Medicine for 2025.

### 128.4. Discussion or Conclusions

Integrating voices of individuals affected by cancer improves cancer care, research, public outreach, knowledge dissemination, and academic training. Through its diverse and enthusiastic membership and productive work, the ACC PAC is expected to support improvements in all of these focus areas in Atlantic Canada.

**39**

## 129. Creating Safe Connections: Co-Implementation and Co-Evaluation of an Educational Intervention Designed to Increase Equity in Lung Cancer Prevention and Early Detection

Zeenat Ladak ^1,2^, Tatiana Demarco ^1,2,3^, Salva Niwe ^4^, Angus Pratt ^5^, Bikila Amenu ^6^, Howard Freedman ^6^, Jean-Claude Camus ^7^, Tara Jeji ^8^, Vinesha Ramasamy ^6^, Vanessa Wright ^9^, Ambreen Sayani ^1,2,10^

1.   Women’s College Hospital Institute for Health System Solutions & Virtual Care, Toronto, Canada.
2.   University of Toronto, Toronto, Canada.
3.   FrancoMed, Toronto, Canada.
4.   University of Waterloo, Waterloo, Canada.
5.   Patient Partner, Vancouver, Canada.
6.   Patient Partner, Toronto, Canada.
7.   Patient Partner, Hamilton, Canada.
8.   Patient Partner, Windsor, Canada.
9.   Women’s College Hospital Crossroads Clinic, University of Toronto, Toronto, Canada.
10. Canadian Partnership Against Cancer, Toronto, Canada.

### 129.1. Background/Rationale or Objectives/Purpose

Inequities in smoking rates contribute to a greater risk of lung cancer and mortality. Systemic biases, stigmatizing clinical encounters and a lack of responsive primary-care create further barriers to smoking cessation and lung cancer screening. To fill this gap, we have co-created an educational intervention to build equity-oriented skills and competencies in primary care providers (PCPs). We are now co-implementing and co-evaluating the impact of the intervention.

### 129.2. Methodology or Methods

This is an equity-oriented, patient-partnered, interdisciplinary, implementation science study. The intervention of focus is an e-learning module for PCPs called Creating Safe Connections. Our co-implementation is ongoing and involves a deliberative dialogue methodology to rapidly synthesize data from meetings with patient partners, healthcare providers, and decision-makers. Meeting data is being used to develop an implementation plan. Our co-evaluation over a one-year period will follow explanatory mixed-methods approaches (surveys and interviews) to assess PCPs participation, satisfaction, and knowledge, guided by the RE-AIM framework (Reach, Effectiveness, Adoption, Implementation, Maintenance).

### 129.3. Impact on Practice or Results

Expected findings will include outcomes of the five RE-AIM domains. For example, R: type of PCPs taking the module (i.e., family physicians, nurse practitioners), E: impact on PCPs’ behaviour, A: type of PCP organizations making the module a priority, I: feasibility for PCPs to take the module, M: number of PCPs completing the module long-term (i.e., 1 year post-implementation).

### 129.4. Discussion or Conclusions

This e-learning module will equip PCPs to engage in safer, more effective conversations about smoking cessation and lung cancer screening and enable patients across Canada’s healthcare system to achieve better health outcomes.

**47**

## 130. The Top 10 Priorities for Adolescent and Young Adult Cancer Research in Canada

Perri Tutelman ^1^, Chantale Thurston ^2^, Tamara Rader ^3^, Tristyn Ranger ^1^, Brianna Henry ^1^, Fiona Schulte ^1^, on behalf of the AYA Cancer PSP Team ^1^

1. University of Calgary, Calgary, Canada.
2. AYA CAN—Canadian Cancer Advocacy, Winnipeg, Canada.
3. James Lind Alliance, Southampton, United Kingdom.

### 130.1. Background/Rationale or Objectives/Purpose

Despite the growing interest in adolescent and young adult (AYA) cancer care, little is known about the most important topics for patients and their caregivers. This project aimed to establish the top 10 research priorities for AYA cancer research in Canada according to patients, caregivers, and clinicians.

### 130.2. Methodology or Methods

This project followed the rigorous James Lind Alliance Priority Setting Partnership (JLA PSP) methodology. The steps of a JLA PSP include: (1) convening a steering group and establishing the project scope, (2) gathering initial research uncertainties, (3) processing the uncertainties, forming summary questions, and verifying the uncertainties, (4) interim priority setting, and (5) a final priority setting workshop.

### 130.3. Impact on Practice or Results

In the initial uncertainties survey, 1,947 potential research questions were submitted by 279 patients, caregivers, and clinicians. Following data processing, summary question formation, and the evidence check, 58 questions were put forward for interim prioritization in a second survey. The top 20 questions from the interim prioritization were ranked at the final priority setting workshop attended by a diverse group of 23 patients, caregivers, and clinicians from across Canada. The top 10 priorities reflect topics across the cancer continuum including: delays in diagnosis, screening and early detection, access to healthcare, development and implementation of novel therapies, psychosocial impacts and supports, end of life concerns, and survivorship issues.

### 130.4. Discussion or Conclusions

This project identified the top research priorities for AYA cancer in Canada according to patients, caregivers, and clinicians. These priorities will inform a national patient-oriented research strategy to improve outcomes for AYA patients and their families.

**49**

## 131. Kamnagogy: Do We Need a Specialized Teaching Method for (Cancer) Patients?

Don Desserud

UPEI, Charlottetown, Canada.

### 131.1. Background/Rationale or Objectives/Purpose

Current practices for communicating devastating diagnoses such as cancer do not adequately take into account the initial psychological trauma being experienced by the patient. We know that effective communication between Health Care Providers (HCPs) and patients is essential to patient care and leads to improved outcomes. HCPs are now being trained to simplify how they deliver crucial information. While this is a step in the right direction, the focus of this approach is primarily on one-way communication and passive learning. As well, while there have been advances in patient education such as using adult learning models (andragogy), such approaches are primarily in the area of informing patients about life-style choices or how to continue treatments at home. In any case, cancer patients are not “just like” adult learners. Andragogy assumes self-motivated, self-directed adult learners who are in the classroom because they want to be and can leave any time they so wish. This does not describe cancer patients. Cancer patients do not want to be in their HCP’s office. They do not want to hear what they are being told. Their world is being turned upside down; they are resistant, reluctant, and most important, very scared. We need a new teaching model that takes into account the present and on-going trauma that results from receiving a devastating cancer diagnosis. We are calling this “kamnagogy”, a coined word meaning “teaching the sick”, and we are looking for research partners to collaborate on developing such a model.

### 131.2. Methodology or Methods

This will be a presentation made by a cancer patient advocate who is looking for research partners with expertise in this field

### 131.3. Impact on Practice or Results

We need a new teaching model that takes into account the present and on-going trauma that results from receiving a devastating cancer diagnosis. We are calling this “kamnagogy”, a coined word meaning “teaching the sick”, and we are looking for research partners to collaborate on developing such a model.

### 131.4. Discussion or Conclusions

See above.

**54**

## 132. Person-Centred Navigation in Rare Cancers Optimizes Innovation Implementation: The Case of the Canadian Cholangiocarcinoma Collaborative (C3)

Samar Attieh ^1,2^, Leonard Angka ^1,3^, Christine Lafontaine ^1,3^, Cynthia Mitchell ^4^, Julie Carignan ^5^, Carolina Ilkow ^3^, Simon Turcotte ^6^, Rebecca C. Auer ^1,2,3^, John Bell ^3^, Carmen G. Loiselle ^7,8^

1. Cancer Research Program, Ottawa Hospital Research Institute, Ottawa, Canada.
2. Department of Surgery, Faculty of Medicine, The University of Ottawa, Ottawa, Canada.
3. Department of Biochemistry, Microbiology, and Immunology, Faculty of Medicine, University of Ottawa, Ottawa, Canada.
4. Patient representative, Winnipeg, Canada.
5. Patient representative, Quebec city, Canada.
6. Département de Chirurgie, Service de Transplantation Hépatique et de Chirurgie Hépatopancréatobiliaire, Centre Hospitalier de l’Université de Montréal (CHUM), Montreal, Canada.
7. Segal Cancer Center, Jewish General Hospital, Montreal, Canada.
8. Department of Oncology and Ingram School of Nursing, Faculty of Medicine and Health Sciences, McGill University, Montreal, Canada.

### 132.1. Background/Rationale or Objectives/Purpose

Person-centred navigation (PCN) including professional, peer, and virtual guidance for patients and their significant others, is a well-documented approach to address gaps in accessing care, support, and innovation. PCN is particularly crucial in rare cancers, where affected individuals face significant uncertainty about their diagnosis and treatment, difficulties accessing timely support, and reduced contact with others with similar diagnoses. The Canadian Cholangiocarcinoma Collaborative (C3) is a recent exemplar of a promising initiative within the context of individuals affected by biliary tract cancers (BTC).

### 132.2. Methodology or Methods

As a national partnership among clinicians, researchers, patients, and informal caregivers, C3 is dedicated to enhancing access to BTC research and care. The C3 person-centred navigation model provides personalized guidance and support, facilitating access to molecular testing, clinical trials, and case reviews at national multidisciplinary rounds. C3 also focuses on building a national network of clinical and research experts, establishing a patient registry, creating a centralized biobank, and promoting support and advocacy groups.

### 132.3. Impact on Practice or Results

Through hands-on guidance and co-designed implementation strategies (e.g., assessing evolving cancer-related needs, integrating research early, and addressing treatment gaps), C3 has already served to optimize its processes and outputs. Challenges have included timely participant recruitment from large and diverse geographical areas, silos in clinical and research information databases, resource constraints, and provincial care disparities.

### 132.4. Discussion or Conclusions

C3’s comprehensive approach highlights the crucial roles of PCN in implementation science and practice. Future directions include expanding the C3 network, enhancing patient engagement, and further integrating research into ongoing clinical care.

**61**

## 133. Developing a Patient and Caregiver Engagement Plan to Amplify Diverse Voices in Canadian Cancer Society Policy Positions

Apiramy Jeyapalan, Olivia Kulbak, Elizabeth Holmes, Tracy Torchetti

Canadian Cancer Society, Toronto, Canada.

### 133.1. Background/Rationale or Objectives/Purpose

People with lived experience of cancer, including patients and caregivers, provide valuable insights into cancer control issues. By incorporating patient and caregiver perspectives, we can align policy positions with the real needs and challenges faced by those directly affected by cancer. Developing a structured engagement plan ensures that a diverse range of voices are heard, leading to more comprehensive, equitable and impactful policy positions that truly reflect the needs of people with cancer and their caregivers.

### 133.2. Methodology or Methods

To develop the patient and caregiver engagement plan, we first identified two main goals: to either gather input from patients and caregivers to understand their experiences before developing a policy position or to get feedback on a draft policy position. We outlined key considerations like defining the scope of engagement, timelines, selecting target demographics to engage and establishing strategies to reach a diverse group of patients and caregivers. Lastly, we collaborated with internal stakeholders to refine the plan, creating a clear, adaptable and repeatable process for engagement.

### 133.3. Impact on Practice or Results

Standardizing patient and caregiver engagement ensures a consistent approach to including voices impacted by cancer and understanding their experiences. This approach provides representation across Canada, including people from rural and urban areas, various racial and ethnic backgrounds, gender identities, income levels, and residency statuses.

### 133.4. Discussion or Conclusions

Lessons learned highlight the need for clear, accessible language and flexibility throughout the process. By defining engagement purpose and standardizing steps, this plan offers a repeatable, adaptable framework for effective patient and caregiver engagement.

**63**

## 134. Patients’ and Nurses’ Perspectives on Psychosocial Symptom Management in Outpatient Malignant Hematology Care: A Qualitative Descriptive Study

Kylie Teggart ^1^, Christine Newbold ^2^, Catherine Spence ^2^, John Watson ^3^, Kari Kolm ^4^, Tammy De Gelder ^4^, Denise Bryant-Lukosius ^1^, Sarah Neil-Sztramko ^1^, Cheryl Page ^4^, Rebecca Ganann ^1^

1. McMaster University, Hamilton, Canada.
2. Patient Partner.
3. Caregiver Partner.
4. Hamilton Health Sciences, Hamilton, Canada.

### 134.1. Background/Rationale or Objectives/Purpose

This study aims to (a) describe patients’ and oncology nurses’ experiences with symptom management care in an outpatient malignant hematology unit, and (b) identify the barriers and facilitators to providing evidence-informed symptom management among specialized and advanced oncology nurses.

### 134.2. Methodology or Methods

A qualitative descriptive study was conducted within the context of a larger experience-based co-design project. A Steering Group of patients, caregivers, clinicians, and organizational leaders was engaged to provide strategic oversight, inform study procedures, and help contextualize findings. Patients with hematologic malignancies, registered nurses, and nurse practitioners from an outpatient unit in Hamilton, ON were recruited using maximum variation sampling. Individual, semi-structured interviews were conducted via phone, Zoom, or in person. Qualitative content analysis was performed using a hybrid inductive and deductive approach, informed by the revised Consolidated Framework for Implementation Research.

### 134.3. Impact on Practice or Results

Patients and oncology nurses reported notable gaps in the management of psychosocial symptoms. Fatigue, anxiety, and depression were described as common, distressing, and often unmanaged. The nature of hematologic malignancies and the outpatient care environment itself contributed to patients’ distress. Nurses reported many barriers to assessing and providing evidence-informed psychosocial symptom management, including a lack of time, privacy, specialized knowledge, access to specialty providers, and a perceived lack of psychosocial symptom management strategies.

### 134.4. Discussion or Conclusions

This study identifies opportunities to provide more proactive and evidence-informed psychosocial care in the outpatient malignant hematology setting. The Steering Group is using these findings to identify the top priorities and co-design strategies to improve outpatient symptom management delivery.

**64**

## 135. Evaluating the Effectiveness of a Whiteboard Video in Enhancing Awareness of Support Resources for AYAs with High-Grade Glioma

Kaviya Devarajah ^1,2^, Natalie Pitch ^2,3^, Abha A. Gupta ^2,4^

1. Institute of Medical Science, University of Toronto, Toronto, Canada.
2. Adolescent and Young Adult Program, Department of Supportive Care, Princess Margaret Cancer Centre, University Health Network, Toronto, Canada.
3. Temerty Faculty of Medicine, University of Toronto, Toronto, Canada.
4. Division of Medical Oncology and Hematology, Princess Margaret Cancer Centre, University of Toronto.

### 135.1. Background/Rationale or Objectives/Purpose

Adolescent and young adults (AYAs) with high-grade glioma face unique challenges due to the intersection of cancer’s demands and their developmental stage. Phase 1 of this study identified significant gaps in resources and tools needed to provide holistic care for AYAs navigating this diagnosis. In response, a whiteboard video titled “Navigating High-Grade Glioma: Comprehensive Support for AYAs at the Princess Margaret Cancer Centre” was developed to educate and raise awareness about available resources and support services. This phase 2 study evaluates the effectiveness of the video in addressing these needs.

### 135.2. Methodology or Methods

This study uses a mixed-methods approach, combining surveys and one-on-one interviews. Participants complete demographic and pre-video surveys to assess baseline knowledge, followed by a post-video survey to measure changes in awareness and perceived utility of the video. Descriptive statistics summarize survey data, while Braun and Clarke’s framework for thematic analysis is applied to interview transcripts to explore participants’ experiences and perspectives on the video. Outcomes focus on changes in awareness, understanding, and satisfaction with support resources.

### 135.3. Impact on Practice or Results

This intervention aims to improve the delivery of holistic care by equipping AYAs with essential knowledge about available resources, enhancing their ability to navigate the healthcare system and address their unique needs during treatment and survivorship. Findings from this evaluation will inform future educational tools and interventions for this population.

### 135.4. Discussion or Conclusions

Preliminary results suggest the video increases awareness and facilitates resource accessibility. Future work will refine content based on participant feedback and explore scaling this intervention to other institutions for broader implementation.

**69**

## 136. Community of Practice: Promoting Patient Engagement, Forming Bridges, and Exchanging Best Practices on Patient Engagement in Cancer Research

Sevtap Savas ^1,2^, Don Wood ^3^, Suzanne Bays ^4^, Meera Rayar ^5,6^, Heather Roy ^7,8^, Judy Needham ^9^

1. Community of Practice, Steering Committee and member, St. John’s, Canada.
2. Memorial University of Newfoundland and Labrador, St. John’s, Canada.
3. Community of Practice Secretariat, Canadian Cancer Society, Calgary, Canada.
4. Caregiver Partner; Community of Practice Member; Canadian Cancer Society, Piedmont, Canada.
5. Community of Practice, Steering Committee, Vancouver, Canada.
6. University of British Columbia, Vancouver, Canada.
7. Caregiver Parent; Community of Practice Member, Calgary, Canada.
8. University of Calgary, Calgary, Canada.
9. Patient Partner; Community of Practice, Steering and Founding Member, Kelowna, Canada.

### 136.1. Background/Rationale or Objectives/Purpose

Patient engagement is essential for timely and meaningful patient-oriented studies that address the needs and priorities of individuals affected by cancer. To promote and enhance patient engagement in cancer research and exchange best practices, a virtual Community of Practice (CoP) was initiated in 2022 under the executive sponsorship of the Canadian Cancer Society.

### 136.2. Methodology or Methods

Here we summarize the goals, activities and impact of CoP using CoP documentations and correspondences (such as, meeting minutes, steering committee activities, presentations about CoP), and responses provided to the member satisfaction survey (administered in 2023 and 2024).

### 136.3. Impact on Practice or Results

Key activities of CoP include virtual open meetings that incorporate presentations and conversations on patient engagement, facilitating connections and collaboration between its members and stakeholders (for example, via its meetings and LinkedIn page), and disseminating knowledge about CoP and patient engagement in scholarly and public spheres. CoP membership has been consistently growing since its inception (8 in 2022; 69 in 2023 and 97 in 2024). Responses to membership survey indicate that CoP delivers its mandates, helps members connect, and improves its members’ knowledge and practice in patient engagement.

### 136.4. Discussion or Conclusions

CoP is a successful, pan-Canadian virtual community on patient engagement in cancer research. It aims to continue to grow and evolve based on members’ needs and changing patient engagement practices. We invite all interested individuals to join CoP, promote and enhance patient engagement, and help integrate patients in cancer research in order to reduce cancer’s impact in Canada and globally.

**72**

## 137. The Journey to Formal Patient Engagement Within a Yoga Research Program

Emma McLaughlin ^1^, S. Nicole Culos-Reed ^1,2,3^, Kaitlyn Quinn ^4^, In memory of Lisa Currey ^4^, Maria-Hélèna Pacelli ^4^, Melissa Coombs ^4^, Sundas Shamshad ^4^, Amanda Wurz ^1,5,6^

1. Faculty of Kinesiology, University of Calgary, Calgary, Canada.
2. Department of Oncology, Cumming School of Medicine, University of Calgary, Calgary, Canada.
3. Department of Psychosocial Resources, AE Child Comprehensive Cancer Centre, Calgary, Canada.
4. Patient Advisory Board, Canada.
5. School of Kinesiology, Chilliwack, Canada.
6. Research Institute, BC Children’s Hospital, Vancouver, Canada.

### 137.1. Background/Rationale or Objectives/Purpose

Patient engagement is important to ensure relevance of research findings for end-users and is required by most funding agencies. This presentation provides an overview of the journey to formal patient engagement within our yoga research for young adults affected by cancer (YAs), which may help others wishing to engage patients in their work.

### 137.2. Methodology or Methods

From 05-06/2020, we offered weekly drop-in yoga classes delivered by videoconference. Following this, we gathered input from 7 YAs who participated and co-developed an 8-week yoga intervention that we evaluated in a pilot trial (09/2020-02/2021). Following the pilot trial, we collated YAs’ perspectives (e.g., emails, comments) and gathered qualitative data from 28/30 participants. We integrated feedback, formed a Patient Advisory Board (Board; 5 YAs, 2 researchers) in 12/2022, and are currently testing a refined 12-week yoga intervention in an ongoing trial.

### 137.3. Impact on Practice or Results

The journey from informal to formal patient engagement took 2 years, and engagement is ongoing. From inception, >30 YAs have shaped this research program, from recruitment approaches used, to the questions asked, assessments used, and yoga intervention delivered. Further, the Board has informed dissemination by co-authoring 2 manuscripts and 2 conference presentations.

### 137.4. Discussion or Conclusions

YAs have informed the vision and mission of our yoga research program. We believe the natural evolution, rooted in valuing feedback from YAs from drop-in classes to intervention conceptualization onward, gradually led to more formalized approaches for centering YAs’ voices. Continued efforts to engage YAs will enhance relevancy of research outputs for end-users.

**109**

## 138. Imagine... Reshaping Adolescent and Young Adult Cancer Care Through Immersive Experience

Cheryl Heykoop ^1^, Alice O’Grady ^2^, Kat Dornian ^3^, Tiffany Hill ^4^, Gary Lam ^5^, Genevieve Stonebridge ^6^, Jennifer Wolfe ^7^, Amber Baker ^8^, Shandy Bearman ^9^, Laura Floyd ^8^, Jennifer L ^9^, Jamie MacIver ^8^, Jodi Rethy ^10^, Kaylie Rooke ^11^, Ashley Tremblay ^9^, Roxanne van Velzen ^8^

1.   Anew, Vancouver, Canada.
2.   Co-Lead, University of Leeds, Leeds, United Kingdom.
3.   Production Manager, Anew, Calgary, Canada.
4.   Senior Researcher, Anew, Vancouver, Canada.
5.   Counsellor, Anew, Vancouver, Canada.
6.   Registered Clinical Counsellor, Anew, Victoria, Canada.
7.   Research Program Coordinator, Anew, Vancouver, Canada.
8.   Co-researcher, Victoria, Canada.
9.   Co-researcher, Vancouver, Canada.
10. Co-researcher, Ganges, Canada.
11. Co-researcher, Abbotsford, Canada.

### 138.1. Background/Rationale or Objectives/Purpose

Over an 18-month period we worked with adolescents and young adults (AYA) with lived experiences with cancer to co-design an immersive experience that captured the felt sense of AYA cancer care and support.

### 138.2. Methodology or Methods

We applied a patient-oriented, Participatory Action Research and Performance as Research research methodology to engage a group of AYAs to co-design an immersive experience.

### 138.3. Impact on Practice or Results

In November 2024, we delivered thirteen 40-min immersive experiences to clinicians, care givers, decision makers, and AYAs at the BC Cancer Summit. Over 100 people experienced the immersive experience and directly explored the felt sense of what it means to be an AYA navigating cancer care and support in BC.

### 138.4. Discussion or Conclusions

Through this immersive experience process, we explored how to best support AYAs as co-researchers (including the provision of ongoing counselling support) in a co-design process. Moving forward we intend to explore how this immersive experience can be shared with others and how we can monitor and evaluate the impact with clinicians, care providers, decision makers and AYAs over time.

Note: This workshop will present the immersive experience (in physical or video format), will discuss the process of development (including the process, challenges, and opportunities), and explore implications for future practice.

**124**

## 139. Addressing Fear of Cancer Recurrence in Parents of Pediatric Cancer Survivors: Assessing the Acceptability and Feasibility of Fear of Recurrence Therapy for Parents (Parent-FORT)

Celeste Holy ^1^, Jeff Brinson, Janelle Gibson-Haerle, Lauriane Giguère ^1^, Greg Gulicher ^2^, Christine Maheu ^3^, Wendy Pelletier ^4^, Fiona Schulte ^4^, Héloïse Sirois-Leclerc ^5^, Perri Tutelman ^4^, Lauren Heathcote ^6^, Sophie Lebel ^1^

1. University of Ottawa, Ottawa, Canada.
2. Alberta Children’s Hospital, Calgary, Canada.
3. McGill University, Montreal, Canada.
4. University of Calgary, Calgary, Canada.
5. Children’s Hospital of Eastern Ontario, Ottawa, Canada.
6. King’s College London, London, United Kingdom.

### 139.1. Background/Rationale or Objectives/Purpose

Fear of Cancer Recurrence (FCR) is a significant concern for parents of childhood cancer survivors. Studies show these parents experience equal or greater levels of FCR than their children, which has been linked to lower quality of life and increased distress. FCR can successfully be addressed in cancer survivors with brief interventions. However, none of these interventions have been tested with parents. Therefore, we have adapted a 7-week online group intervention aimed at reducing FCR in parents of childhood cancer survivors (Parent-FORT).

### 139.2. Methodology or Methods

FORT was first adapted with the guidance of ten parents (residing in Canada) recruited to a parent advisory committee. Focus groups were held bi-weekly, and feedback was received from parents on how to best adapt the current workbook to meet their needs. Usability was then assessed by five parents who participated in the 7-week intervention, led by two trained therapists. Parents and therapists completed questionnaires on the acceptability of the content and overall readiness of sessions. Exit interviews will be conducted with parents and therapists to gather additional feedback, which may result in another round of usability testing.

### 139.3. Impact on Practice or Results

FORT modifications included having conversations on FCR, “do’s and dont’s” when your child has a new symptom, and reflections on how life has changed since cancer. Findings from this study will determine if Parent-FORT should be tested in a pilot study to determine feasibility, acceptability and evidence of clinically reduced FCR.

### 139.4. Discussion or Conclusions

This research addresses an important gap in bringing evidence-based FCR care to parents of childhood cancer survivors in Canada.

**135**

## 140. “I Had to Be the Strong One”: Young People with Lived Experience Interpret Data Anomalies in Psychosocial Oncology Research

Chana Korenblum ^1,2,3^, Rachel Taylor ^4^, Rachael Hough ^5^, Bethany Wickramasinghe ^6^, Lorna Fern ^7^

1. Princess Margaret Cancer Centre, Toronto, Canada.
2. The Hospital for Sick Children, Toronto, Canada.
3. Temerty Faculty of Medicine, University of Toronto, Toronto, Canada.
4. Centre for Nurse, Midwife and Allied Health Profession Led Research (CNMAR), University College London Hospitals NHS Foundation Trust and Department of Targeted Interventions, University College London, London, UK.
5. Children and Young Peoples Cancer Service, University College London Hospitals NHS Foundation Trust and Cancer Institute, University College London, London, United Kingdom.
6. Research Fellow, Department of Targeted Intervention, University College London, London, United Kingdom.
7. Cancer Clinical Trials Unit, University College London Hospitals NHS Foundation Trust, London, United Kingdom.

### 140.1. Background/Rationale or Objectives/Purpose

Adolescents and young adults with cancer (AYA) face unique psychological challenges compared to younger children and older adults. There are little published data on symptoms of distress in AYA. In a quantitative study, we identified that distress was negatively impacted by increasing amounts of social support, counterintuitive to expectations. We sought to understand this relationship by consulting our Young Advisory Panel (YAP) with lived experience with a cancer diagnosis, examining their impressions of the results, and identifying future research and clinical implications.

### 140.2. Methodology or Methods

Seven female YAP members, aged 14–20 at diagnosis with typical AYA cancers, attended two 1.5-h online workshops. A presentation of study results was followed by an interactive whiteboard exercise and a focus group discussion. The whiteboard content and workshop transcripts were reviewed independently by three researchers, highlighting key themes.

### 140.3. Impact on Practice or Results

Participants felt mental health in AYA was important, not well acknowledged by their friends and family, and underexplored by their healthcare team. On examination of the relationship between social support and distress, positive and negative impacts were identified. Practical support from family and friends was helpful, however, feeling like a burden and wanting to protect loved ones from further emotional upset increased distress levels.

### 140.4. Discussion or Conclusions

Set in context by young people with lived experience of cancer, study findings about factors affecting psychosocial distress provide a deeper understanding of their mental health. This information can inform the design of individualized, effective screening and interventions to mitigate distress and improve quality of life for AYA.

**201**

## 141. The Contributions of Nurse Navigators to More Positive Cancer Care Experiences

Carmen G. Loiselle ^1,2^, Samar Attieh ^3^

1. Department of Oncology and Ingram School of Nursing, Faculty of Medicine and Health Sciences, McGill University, Montreal, Canada.
2. Segal Cancer Center, Jewish General Hospital, Montreal, Canada.
3. Division of Experimental Medicine, Faculty of Medicine and Health Sciences, McGill University, Montreal, Canada.

### 141.1. Background/Rationale or Objectives/Purpose

Nurse navigators (NNs) play a key role in people-centered care, yet patient-reported evidence is still limited. This study compared patient experiences and satisfaction between those who reported being assigned a NN and those who did not. Patient experiences included six key domains—emotional support, care coordination, respect for preferences, comfort, communication, and access. In addition, four core NN functions (i.e., assessment, information, support, and care coordination) were included in the analysis.

### 141.2. Methodology or Methods

2858 individuals diagnosed with cancer, who received treatment in the last six months at a university-affiliated cancer center in Montréal, Québec, completed the Ambulatory Oncology Patient Satisfaction Survey (AOPSS) and items related to NN assignment. Mean differences for all domains and functions were calculated for the NN group (n = 2003) and the non-NN group (n = 855).

### 141.3. Impact on Practice or Results

Patients in the NN group reported significantly higher satisfaction and more positive cancer care experiences across all six cancer care domains (mean differences ranging from 3.32 to 8.95) with the largest difference observed for emotional support (i.e., 8.95; 95% CI: 7.61, 10.29). All four nursing functions were also rated higher in the NN group (differences ranging from 5.64 to 10.39). 83% of participants rated NN as very to extremely useful (5 or above) on a 7-point scale.

### 141.4. Discussion or Conclusions

This study underscores the key role of nurse navigators in optimizing cancer care. Future work should focus on assessing NN training and development needs, the integration of technology (e.g., AI), and conducting cost-effectiveness/value-based analyses according to various patient navigation models.

## 142. Final Category: M. Primary, Secondary and Tertiary Cancer Prevention

**31**

## 143. Youth and Nicotine Pouches: The Concerning New Trend Examined Through TikTok and Its Implications for Cancer Prevention

Ashmeet Mand, Laura Struik

University of British Columbia- Okanagan, Kelowna, Canada.

### 143.1. Background/Rationale or Objectives/Purpose

Nicotine pouches have been marketed as a less harmful alternative to smoking. They began to gain popularity, particularly among youth, due to their discrete nature and convenience. However, despite claiming reduced harm, these pouches deliver high nicotine concentrations, posing a risk to oral health and cancer development. Social media platforms, like TikTok, play a major role in promoting and normalizing their use, particularly among youth. Little is known about how nicotine pouch content is presented on platforms like TikTok. This study uses a qualitative descriptive design to explore how nicotine pouches (specifically Zyns) are represented on TikTok.

### 143.2. Methodology or Methods

A total of 200 TikToks posts were screened under the #zyn, #zyns, and #nicotinepouch hashtags, and 132 were analyzed. Posts were analyzed using Braun and Clarke’s thematic analysis approach. Collaborative coding ensured reliability and uncovered key themes in the content.

### 143.3. Impact on Practice or Results

Five themes emerged: (1) The Zyn Movement; (2) “Boy Heaven”; (3) “Unintended Negative Consequences”; (4) Product design: “Life doesn’t need to stop”; (5) Physical Benefits: “Its like IcyHot for your Mouth”. Overall, the content heavily condoned nicotine pouch use and normalized it, with males disproportionately represented as the primary users of nicotine pouches.

### 143.4. Discussion or Conclusions

This study highlights TikTok’s influence in shaping youth perceptions of nicotine pouches as something trendy and relatively harmless. This underlines the need for targeted interventions and policies to protect youth from the risks of nicotine pouch use, including cancer.

**34**

## 144. Estimates of HPV Vaccination in Canadian Children: Data from the 2021 Childhood National Immunization Coverage Survey

Gwen Eyre ^1^, Tara Martin ^1^, Julien Robitaille ^1^, Selma Osman ^1^, Shelly Bolotin ^1^, Ramandip Grewal ^1^, Gilla Shapiro ^2^

1. Dalla Lana School of Public Health, Toronto, Canada.
2. Princess Margaret Cancer Centre, Toronto, Canada.

### 144.1. Background/Rationale or Objectives/Purpose

Human papillomavirus (HPV) is responsible for almost 3800 new cancer cases annually in Canada, including cervical, head and neck, and anogenital cancers. School-based HPV vaccination programs were first introduced by Canadian provinces/territories for females (2007–2011) and subsequently became gender-neutral in all jurisdictions (2013–2017). Despite national targets of 90%, HPV vaccine coverage estimates often fall short. Understanding how vaccination differs by sociodemographic factors is key to increasing coverage.

### 144.2. Methodology or Methods

We analyzed data from the 2021 Childhood National Immunization Coverage Survey (cNICS). Conducted by Statistics Canada, cNICS estimates the national immunization coverage rates for childhood vaccines across Canada. Data were collected via self-response electronic questionnaires or telephone interviews based on a representative sample stratified by child’s province/territory of residence and age. Parents/guardians of 14-year-olds were asked about their child’s HPV vaccination status and sociodemographic characteristics. HPV vaccination responses included ‘yes’ (i.e., child is HPV vaccinated), ‘no’ (i.e., child is not HPV vaccinated) or ‘unknown’ for one or more doses. Univariate multinomial logistic regression was used to determine associations between each sociodemographic variable and HPV vaccination, with ‘Vaccinated’ used as the reference.

### 144.3. Impact on Practice or Results

There were 755 parents (weighted n = 413,255) who responded to questions relating to their child’s HPV vaccination status. HPV vaccination was significantly higher in girls compared to boys (*p* < 0.05). Nearly twice as many boys (14.2%) had unknown vaccination status, compared to girls (7.4%). Compared to vaccinated children, children who were unvaccinated were significantly more likely to belong to families with a greater number of children in the home (all *p* < 0.05). Compared to vaccinated children, children who had unknown vaccination status were significantly more likely to be boys and of Indigenous identity, and they were also significantly more likely to have parents who were high school educated and born outside of Canada (all *p* < 0.05).

### 144.4. Discussion or Conclusions

HPV vaccine coverage varied by sociodemographic factors, with all estimates falling short of the 90% target. Our study demonstrates the importance of examining correlates of HPV vaccination to identify subgroups who may require tailored intervention.

**40**

## 145. Social Influences on Youth Experiences with Quitting Vaping: Implications for Caring for the Next Generation

Laura Struik ^1^, Mischa Taylor ^1^, Danielle Rodberg ^1^, Roula Nawara ^1^, Karlee Fonteyne ^1^, Tina Afshar ^2^, Youjin Yang ^1^

1. UBC, Kelowna, Canada.
2. UBC, Vancouver, Canada.

### 145.1. Background/Rationale or Objectives/Purpose

While it is well known that familial and social influences play a major role in e-cigarette uptake among youth, it is less well known how these same influences play a role in quitting e-cigarette use. Understanding this is critical to providing care and support to the next generation and ultimately protecting them from the cancer risk associated with e-cigarette use. Therefore, this research study examines the complex social influences on youth vaping cessation.

### 145.2. Methodology or Methods

Using a qualitative descriptive design, we conducted fifteen semi-structured interviews with youth ages 16 to 24 who had experiences with vaping and cessation. Inductive thematic analysis was employed to extract themes.

### 145.3. Impact on Practice or Results

Four key themes emerged, including (1) limited effect of social networks on decision to quit; (2) vaping/ex-vaping community influences vaping cessation journeys; (3) seeking support from social networks may be contingent on the nature of the relationship; and (4) normalization of vaping cessation could be helpful in vaping cessation journeys.

### 145.4. Discussion or Conclusions

This study highlighted a significant gap in providing Canadian youth with vaping cessation support that considers the social dimensions of quitting. The findings highlight the need for comprehensive programs that normalize cessation, reduce stigma, and promote supportive environments. Multi-sectoral efforts are needed at the policy and intervention levels to ensure that youth have access to resources that empower (vs. isolate) youth in their cessation journeys, ultimately reducing their cancer-related morbidity and mortality risk.

**48**

## 146. Understanding the Psychosocial Needs & Piloting a Psychology Service for Patients with Hereditary Cancer Risk

Kristina Cahill ^1^, Paul D’Alton ^2^, Elaine Lowry ^2^, Sara Scardini ^2^, Claire Coffey ^2^, Sonya Deschênes ^2^, Lisa Cadden ^1^, Louise O’Driscoll ^1^

1. St. Vincent’s University Hospital, Dublin, Ireland.
2. University College Dublin, Dublin, Ireland.

### 146.1. Background/Rationale or Objectives/Purpose

Services in Ireland for patients with hereditary cancer risk lag far behind international best practice. To begin bridging this gap, the Irish Cancer Society funded a 3-year project to undertake the following aims:

- Conduct a nationwide psycho-social needs assessment of patients with hereditary cancer risk.
- Work with healthcare service providers nationally to assess their understanding of patient’s psycho-social needs, current gaps in psycho-social service provision, and how these might be addressed.
- Design, pilot, and evaluate a psychology service for patients with hereditary cancer risk embedded within a high-risk family history clinic in an acute hospital.

### 146.2. Methodology or Methods

Psychosocial needs assessment included a cross-sectional survey of (n = 152) participants. A mixed methods approach was used to explore the psychosocial needs from the perspective of healthcare providers (n = 30). A further study was conducted reviewing patients referred to the specific-psychology service (n = 51), their engagement with interventions, and the outcomes of interventions.

### 146.3. Impact on Practice or Results

Findings from all studies indicate that individuals with hereditary cancer risk often experience heightened anxiety, depression, and uncertainty about the future.

### 146.4. Discussion or Conclusions

This project highlights the importance of psychological support to help individuals manage these challenges. It underscores the need for healthcare providers to consider not only the medical but also the emotional and social aspects of hereditary cancer risk when offering care. By addressing these psychosocial needs, healthcare systems can improve the quality of life and well-being of individuals at increased risk for hereditary cancers, promoting a more holistic approach to healthcare.

**94**

## 147. Periodontal Disease and Its Association with Colorectal Cancers—A Systematic Review

Violet D’Souza ^1,2,3^, Jasmine Mann ^1^, Yuqi Wang ^1^

1. Faculty of Dentistry, Dalhousie University, Halifax, NS, Canada.
2. Department of Surgery, Faculty of Medicine, Dalhousie University, Halifax, NS, Canada.
3. Dept. of Community Health and Epidemiology, Dalhousie University, Halifax, NS, Canada.

### 147.1. Background/Rationale or Objectives/Purpose

Periodontal disease (PD), the second most common oral disease, is associated with colorectal cancers (CRCs). CRCs are the 3rd most common cancer affecting both men and women. The study aimed to examine the relationship between PD and CRCs.

### 147.2. Methodology or Methods

This systematic review was conducted by systematically searching the electronic databases PubMed and Embase and manually searching for relevant scientific articles published on the topic. Original population-based studies that investigated the causal relationship between PD and CRCs were included if they were published in English before December 2022, had moderate to high-quality scores, and had at least 50 individuals with CRC. Two reviewers independently screened studies while resolving the disagreement with consensus. Data were extracted using predetermined data extraction forms, and a quality assessment was performed.

### 147.3. Impact on Practice or Results

Of the identified 4011 reports, 12 met the inclusion criteria and were included in the synthesis. Of them, seven were prospective cohort studies, four were retrospective cohort studies, and one was a case-control study. The studies were of moderate to high quality. In six studies, participants were at least 40 years or older. Three studies reported PD as a significant risk for CRC. One study examined the impact of PD management and reported that those who were treated for PD had a lower risk for CRC. The remaining studies had inconclusive findings.

### 147.4. Discussion or Conclusions

Our results suggest that periodontitis may be a risk factor for CRC, which needs to be confirmed. Also, prevention and management of PD may reduce the burden of CRC.

**114**

## 148. “Make HPV Vaccine Free for Me”: Lessons from a Campaign Aiming to Prevent Cancer by Extending HPV Vaccine Coverage

Gillian Cameron ^1^, Kirsten Krose ^1,2^, Dallas Barnes ^1,3,4^, Shania Bhopa ^1^, Gilla Shapiro ^1,5,6^

1. The HPV Prevention Advocacy Coalition, London, Canada.
2. SWON Public Affairs, London, Canada.
3. Reya Health, London, Canada.
4. Femtech Canada, Hamilton, Canada.
5. Princess Margaret Cancer Centre, Toronto, Canada.
6. University of Toronto, Toronto, Canada.

### 148.1. Background/Rationale or Objectives/Purpose

Human papillomavirus (HPV) vaccination is a critical preventative measure against several cancers including cervical, anal, vaginal, vulvar, penile, and oropharyngeal. The HPV vaccine is underused in Canada. Following the National Advisory Committee on Immunization’s recommendation, some provinces offer the vaccine to those who missed it during school; however, HPV vaccination is not yet publicly available in Ontario and uninsured young adults face challenges accessing this vaccine.

### 148.2. Methodology or Methods

We describe a pro bono, grassroots campaign that seeks to extend the eligibility of HPV vaccination in Ontario. The “Make HPV Vaccine Free for Me” campaign led by SWON Public Affairs and the HPV Prevention Advocacy Coalition seeks to make the HPV vaccine freely accessible to all adults under the age of 26 in Ontario. In this presentation, we discuss the campaign’s development, ongoing activities, challenges faced, and next steps.

### 148.3. Impact on Practice or Results

This campaign, initiated by a young adult with firsthand experience, has collected over 33 thousand signatures on our Change.org petition; been published widely (including in Global News, CBC: The National, and Canada Healthwatch); and has now received the support of leading health organizations (such as HPV Global Action, Action Canada, and Canadian Women’s Foundation). In partnership with MPP Kristyn Wong-Tam, we also championed a motion to expand coverage for key health measures including the HPV vaccine.

### 148.4. Discussion or Conclusions

This campaign supports cancer prevention and a more inclusive healthcare system that benefits all regardless of income or geography. We will continue our advocacy and educational efforts to promote access to HPV vaccines.

**170**

## 149. Informed Choices: HPV-Related Cancer Prevention Using Digital Storytelling

Michael Lang ^1,2,3^, Beverly Milroy ^4^, Lindsay Wodinksi ^4^

1. University of Calgary, Calgary, Canada.
2. Mike Lang Stories, Calgary, Canada.
3. Common Language Digital Storytelling, Calgary, Canada.
4. Alberta Health Services, Edmonton, Canada.

### 149.1. Background/Rationale or Objectives/Purpose

Since the COVID-19 pandemic childhood vaccination rates have been declining around North America. This phenomenon has exacerbated the already low HPV vaccination rates in Alberta, despite significant amounts of data demonstrating the efficacy and safety of this vaccine. New methods of communicating important information to vaccine hesitant populations are needed to increase the uptake of the HPV vaccine and decrease the incidence of head and neck and reproductive cancers associated with HPV. Digital storytelling, in which people create their own first-person, narrated short-film, is a cost-effective, meaningful, and compelling form of communication which has potential to influence parents beliefs, attitudes, and values related to the HPV vaccine.

### 149.2. Methodology or Methods

This presentation will begin by providing a brief overview of the Digital Storytelling HPV prevention strategy being piloted by the Cancer Prevention and Innovation (CPSI) team at Alberta Health Services. It will then screen one digital story created by a parent about their experience with an HPV related-cancer and how it impacted their own decision to immunize their child. This will be followed by a brief audience discussion with the presentation concluding with ideas on how Digital Storytelling can be incorporated into primary, secondary, and tertiary cancer prevention strategies.

### 149.3. Impact on Practice or Results

Attendees will leave with a greater understanding of how utilizing Digital Storytelling in a primary cancer prevention capacity could provide a new and innovative way to talk to vaccine hesitant parents. They will leave with direct access to patient-created digital stories about HPV prevention that they can use in their own practice.

### 149.4. Discussion or Conclusions

As vaccine hesitancy continues to grow, all oncology professionals have a responsibility to discuss prevention strategies with their patients and families. Indeed, psychosocial oncology professionals may have unique opportunities in their work to promote primary cancer prevention strategies. Parents who have cancer and also have young children could be an ideal population for psychosocial professionals to share HPV vaccine information with, and this presentation will provide attendees with both the language and the stories to do this in an effective manner.

## 150. Final Category: N. Novel Interventions and Clinical Trials in PSO

**37**

## 151. Development of a New Group Intervention to Support Parental and Family Emotional Health in Pediatric Oncology

Mélina Rivard ^1^, Élodie Bergeron ^2^, Christine Lefebvre ^1^, Julie Tremblay ^2^, Léandra Desjardins ^3^, Zakaria Mestari ^1^

1. Université du Québec à Montréal, Montréal, Canada.
2. Leucan, Montréal, Canada.
3. Centre Hospitalier Universitaire Ste-Justine, Montréal, Canada.

### 151.1. Background/Rationale or Objectives/Purpose

Mental health support for parents of a child living with cancer is crucial for theirs as well as their child’s well-being. However, it is often overlooked. A general survey, conducted by a community organisation, provided further insight into the psychosocial needs of families dealing with paediatric cancer. The opinions voiced suggested the necessity of an intervention targeting aspects of parenting generally neglected in public health service settings. The community service provider partnered with academics to co-create a group intervention to help parents address these needs.

### 151.2. Methodology or Methods

The modified Delphi method was used. Topics addressed were extracted from the survey results, best practices and theoretical knowledge. These topics were the targeted population, intervention mechanisms, dispensation modalities, expected outcomes, and barriers and facilitators. The 21 experts participating in the Delphi iterations were parents of children living with cancer, clinicians, service managers, and scientists with expertise in pediatric oncology.

### 151.3. Impact on Practice or Results

Analysis of the two mixed questionnaire and three focus groups content was used to generate a logic model. This model informed the contents and modalities of the intervention, an online bi-weekly group meeting of around 8 parents, offered by a parent whose child went through oncology treatments teamed with a mental-health care professional.

### 151.4. Discussion or Conclusions

This intervention was developed in a partnership between paediatric oncology experts—academic, clinical and parents with firsthand experience. We are confident the next phase—real-life testing—will provide health providers with a solid and useful evidence-based intervention.

**38**

## 152. The Development and Evaluation of a Novel Clinical Service Aimed at Addressing Wait-Times for Psychosocial Care in Alberta: The ‘Coping with a New Cancer Diagnosis Class’ Pilot

Dana Male ^1,2^, Stephanie Gauvin ^3^

1. Arthur Child Comprehensive Cancer Centre, Calgary, Canada.
2. University of Calgary, Calgary, Canada.
3. Acadia University, Wolfville, Canada.

### 152.1. Background/Rationale or Objectives/Purpose

It is estimated that 1 in 2 Albertans will receive a diagnosis of cancer in their lifetime (Alberta Health Services, 2021). The period of time between receiving a cancer diagnosis and commencing active treatment is considered one of the most difficult times across the cancer trajectory (de Sousa Barros et al., 2018; Landmark & Wahl, 2002) and is associated with acute distress (Burgess et al., 2005; Mehnert et al., 2014). Psychosocial interventions are efficacious for helping individuals navigate these challenging times (Carlson & Bultz, 2003); however, high wait times for psychosocial oncology services pose a barrier to access. Clinical wait times across many countries/cities saw a significant increase following the shift to virtual care delivery with the onset of COVID-19 (Wiseman et al., 2020, Podubinski et al., 2021, Sud et al., 2020). Between January 2020 (before the implementation of COVID-19 public health measures in Canada) and November 2021, the wait time for a first counseling appointment with a psychosocial clinician at the Tom Baker Cancer Centre (TBCC) increased from 2 weeks to 11 weeks. As the demand for psychosocial oncology services continues to grow without commensurate increases in funding/resources, there is a need for innovative clinical solutions that allow patients and families to be seen at the right time, to mitigate increasing distress that can negatively affect their subsequent adjustment to illness.

### 152.2. Methodology or Methods

Canadian guidelines for the delivery of psychosocial oncology services recommend that interventions be delivered according to a stepped care model (Mackay et al., 2023), first offering group-based programs to meet common needs, with access to individualized and specialized services for those requiring additional care (Mackay et al., 2023). The purpose of this study was to develop and pilot a novel two-hour virtual class for people coping with a new diagnosis of breast or gynecological cancer. The class draws upon evidence-based therapies to provide education about common emotions and reactions, teach coping skills, and review practical tools and community-based resources. The goal was to provide an evidence-based clinical service to patients referred to psychosocial oncology at a reduced wait time (4 weeks or less, based on once-monthly offerings) and free up higher resource-intensive/specialty services by serving a greater number of patients (maximum registration of 20 patients) in a single clinical encounter.

### 152.3. Impact on Practice or Results

Over a 9-month period, 120 participants registered for the ’Coping with a New Cancer Diagnosis’ class and 90 attended (75% attendance rate; M = 10 participants per class). 96% of survey respondents endorsed the class (“would recommend this class to other patients who have received a new diagnosis”. Pre-post data suggests that participants experienced a decrease in their level of distress and a slight increase in their perceived ability to cope after the class. Nine months following participation in the class, 34% of class participants had gone on to access further (individualized or specialized) psychosocial services; by 2 years later, 54% had done so.

### 152.4. Discussion or Conclusions

These findings suggest that a two-hour co-facilitated virtual class aimed at helping reduce distress and bolster coping during the acute stress of receiving a new cancer diagnosis is feasible to recruit for and implement and is acceptable to participants. Preliminary data suggests that the class largely meets the needs of those seeking psychosocial services shortly after receiving a new diagnosis. These results led to the integration of this class into standard psychosocial programming twice monthly at our cancer centre, and increased accessibility for all patients across the province of Alberta with any type of cancer.

**68**

## 153. A Scoping Review of the Use of SMARTs to Evaluate Adaptive Behavioral Interventions

Sydney Wasserman ^1^, Sylvie Lambert ^1,2^, Melanie Bédard ^1^, Erica Moodie ^1^

1. McGill University, Montreal, Canada.
2. St. Mary’s Research Centre, Montreal, Canada.

### 153.1. Background/Rationale or Objectives/Purpose

Traditional randomized controlled trials often adopt a “one size fits all” approach, which is inadequate for developing adaptive interventions tailored to individual needs. Sequential Multiple Assignment Randomized Trials (SMARTs) provide an innovative framework for designing and evaluating adaptive interventions. This scoping review examines the application of SMARTs in behavioral interventions, focusing on design types, intervention components, targeted behaviors, and barriers/facilitators to implementation.

### 153.2. Methodology or Methods

Sample/Setting: The review focuses on behavioral interventions targeting adults with chronic mental (e.g., depression) or physical (e.g., diabetes, cancer) health conditions.

Procedures: A comprehensive literature search was conducted in Ovid-Medline, Ovid-Embase, PsycInfo, and CINAHL using an iterative three-step process beginning in July 2023. Records were managed using the Rayyan QCRI program. Narrative synthesis and deductive content analysis were performed, extracting data based on predefined categories.

### 153.3. Impact on Practice or Results

From 42,117 titles screened, 239 full texts were reviewed, and 14 studies included. A two-stage SMART design was most common, involving initial randomization to treatments followed by re-randomization based on response to tailoring variables and intermediate outcomes. Most interventions followed a stepped-care approach. Tailoring variables were categorized as proximal (mechanistic measures), distal (outcomes), or adherence-related, with most falling into the distal category, addressing symptoms (e.g., pain) and outcomes (e.g., weight loss). Strengths of SMARTs include their rigor and effectiveness in optimizing tailored interventions, while challenges involve their complexity, cost, and recruitment demands.

### 153.4. Discussion or Conclusions

SMARTs hold significant potential to refine adaptive interventions in behavioral health research. Insights from this review can guide the optimization and broader implementation of SMART designs to meet diverse population needs.

**76**

## 154. A Mixed Methods Examination of the Relationship Between Narrative Style, Personal Wisdom, and Improved Affect Following a Single-Session Narrative Care Interview

Lucas Norton ^1^, Karen Fergus ^1^, Iana Ianakieva ^1^, David Olsen ^1^, Erin Gross ^2^, Benjamin Stevenson ^1^, Catriona Buick ^1^

1. York University, Toronto, Canada.
2. Sunnybrook Health Sciences Centre, Toronto, Canada.

### 154.1. Background/Rationale or Objectives/Purpose

Between 2017 and 2024, we tested the effectiveness of a single session narrative care intervention (NCI) for individuals following cancer treatment. Sub-analyses of NCI transcripts found that participants who self-reported the greatest benefit from the NCI also exhibited specific narrative features, including willingness to discuss fundamental life matters and an orientation toward personal growth. These participants were also more inclined to report higher positive affect following the NCI. The goals of this study are (1) to examine the relationship between narrative style and personal wisdom discourses following cancer treatment, and (2) to explore the relationship between narrative style and improvement in affect after participation in the NCI using a mixed methods approach.

### 154.2. Methodology or Methods

Twenty transcripts from the NCI project are being examined—10 from individuals who showed improvement in effect on the PANAS scale and 10 who did not. Selection of transcripts was performed by one researcher, while the coding system was applied by two trained RAs blind to participant group. Transcripts are reviewed and rated on five dimensions: Narrative Openness, Reflexivity, Narrative Complexity, Emotional Engagement, and Narrative Agency.

### 154.3. Impact on Practice or Results

Once the coding process is completed, the results will be examined for consistent patterns to determine if an openness to cultivating personal wisdom correlates with improvements in affect following the NCI.

### 154.4. Discussion or Conclusions

Demonstration of an association between the openness to personal wisdom construct and greater benefit from the NCI will provide a rationale for the development of a screening tool to identify patients most likely to benefit from the NCI.

**80**

## 155. No Soul Left Behind: Building Capacity to Support Complex Trauma in Psychosocial Oncology Interventions

Danielle Petricone-Westwood

DPW Psychology, Toronto, Canada.

### 155.1. Background/Rationale or Objectives/Purpose

Complex trauma (CT) is defined as repeated, interpersonal traumatic experiences, often involving a caregiver or attachment figure in childhood or adolescence. CT is associated with severe emotional disorders like borderline personality disorder, complex post-traumatic stress disorder, and other emotional and interpersonal dysregulation. People with these conditions likely experience additional barriers to psychosocial care due to stigma and perceived unresponsiveness to care among healthcare providers. Oftentimes, such disorders are perceived as outside of the scope of practice or capacity for clinicians in psychosocial oncology, presenting a barrier to care. This workshop will focus on increasing clinical capacity to support people with CT facing cancer.

### 155.2. Methodology or Methods

We will review theoretical models of CT and teach practical tools that can be integrated into commonly used models of care. We will discuss adaptation of Dialectical Behaviour Therapy and psychosocial interventions for people with both CT and cancer. Experiential exercises and vignettes will be used through group discussions to facilitate learning.

### 155.3. Impact on Practice or Results

Learning objectives for participants include:

1. Understanding how CT relates to severe emotional disorders, and cancer-specific considerations;
2. Mechanisms of action that cause emotional dysregulation in people with CT;
3. How to keep clients within a window of tolerance and benefiting from psychotherapeutic interventions;
4. Troubleshooting strategies;
5. Managing self-care and transference concerns for clinicians working with CT.

### 155.4. Discussion or Conclusions

This workshop aims to improve confidence in clinicians when delivering psychotherapeutic support to people affected by CT in cancer care. We aim to equip participants with the skills to better understand and respond effectively to these patients’ needs.

**107**

## 156. Implementation of the Fear of Recurrence Therapy (FORT) Intervention: Preliminary Results of a Mixed Methods Study

Emma Kearns ^1^, Alanna Chu ^1^, Sara Beattie ^2^, Andrea Feldstain ^3^, Sheila Garland ^4^, Cheryl Harris ^5^, Jennifer Jones ^6^, Christine Maheu ^7^, Linda Carlson ^3^, Sophie Lebel ^1^

1. University of Ottawa, Ottawa, Canada.
2. Tom Baker Cancer Center, Calgary, Canada.
3. University of Calgary, Calgary, Canada.
4. Memorial University of Newfoundland, St John’s, Canada.
5. The Ottawa Hospital, Ottawa, Canada.
6. Princess Margaret Cancer Center, Toronto, Canada.
7. McGill University, Montréal, Canada.

### 156.1. Background/Rationale or Objectives/Purpose

Fear of cancer recurrence (FCR) is the “fear, worry or concern relating to the possibility that cancer will come back or progress” and is the most common need among survivors. Fear of Recurrence Therapy (FORT) is a manualized, six-week cognitive-existential group intervention. Following two randomized controlled trials, FORT shows evidential posttreatment FCR reductions in women with breast and gynecological. To bridge clinical knowledge into practice, the implementation of FORT has begun in five large centers across Canada.

### 156.2. Methodology or Methods

A mixed-methods comparative case study employing the Reach Effectiveness Adoption Implementation Maintenance (RE-AIM) framework is underway. Phase I: Implementation facilitators and barriers were evaluated following qualitative interviews (*n* = 20) with key decision-makers and clinicians in five clinical centers and categorized according to the Consolidated Framework for Implementation Research. Phase II: Two FORT groups will be administered at each site (i.e., 10 FORT groups of 6-8 survivors in 12 months). Data collection is underway, and analysis of implementation outcome data will conclude in early 2025.

### 156.3. Impact on Practice or Results

Preliminary results indicate that leadership support and compatibility with organizational priorities and existing clinical workflows (e.g., referral pathways, renumeration for research hours) is critical for successful implementation.

### 156.4. Discussion or Conclusions

Initial site feedback indicates necessity of a tailored plan to support the implementation of novel group interventions in psychosocial oncology. A comprehensive implementation strategy and initial posttreatment outcomes (i.e., T1-T2 FCR, uncertainty), attendance, satisfaction, fidelity and cost will be presented following the conclusion of Phase II.

**111**

## 157. Optimizing Care for Family Caregivers in Oncology

Rinat Nissim

Princess Margaret Cancer Centre, Toronto, Canada.

### 157.1. Background/Rationale or Objectives/Purpose

Family caregivers are essential to the cancer care system, providing physical, emotional, and practical support for individuals with cancer. As cancer patients live longer and require more outpatient care, caregivers’ responsibilities intensify, often leading to psychological, physical, and financial strain. Many caregivers feel unprepared and experience distress levels higher than both the general population and the patients they care for. This highlights the critical need for targeted attention to caregiver needs within the cancer care system.

### 157.2. Methodology or Methods

In response, the Princess Margaret Cancer Centre in Toronto established Canada’s first Caregiver Clinic, serving as a clinical, educational, and research hub for family caregiver support. The clinic’s philosophy emphasizes that caregiver well-being is a shared responsibility between caregivers and the healthcare system, framing caregiver distress as an actionable indicator of cancer care quality. The clinic offers tailored interventions that can be accessed independently of patient involvement.

### 157.3. Impact on Practice or Results

The Caregiver Clinic has pioneered a model that integrates caregiver support into cancer care, fostering broader institutional recognition of caregivers’ psychosocial needs as a core component of cancer treatment.

### 157.4. Discussion or Conclusions

Key lessons include the importance of maintaining separate medical records for caregivers, ensuring privacy and facilitating open communication. The clinic’s sustainability is supported by its integration into existing infrastructure, as well as ongoing philanthropic funding and research initiatives. The clinic model is scalable and can be adapted in other cancer institutions.

**191**

## 158. “Love Is Letting Go of Fear”: An Experiential Workshop Based on the Non-Dual Spiritual Teaching Used in a Cancer Patient Empowerment Program

Robert Rutledge, Gabriela Ilie

Dalhousie University, Halifax, Canada.

### 158.1. Background/Rationale or Objectives/Purpose

The Cancer Patient Empowerment Program is a comprehensive six-month daily home-based health-promotion program tested in a phase 2 trial of 104 adult cancer survivors. Mental distress was significantly improved at six and 12 months of follow up. The program includes daily emails and ‘PEP’ videos teaching and encouraging healthy lifestyle choices: exercise, strength training, dietary advice, relaxation techniques, relationship teaching and prescribed activities, healthy habit formation, and deeper psychological work. Social support via monthly videoconferences, and a buddy system kept the program ‘organic’.

The Cancer Patient Empowerment Program (PC-PEP) is a six-month, home-based, health-promotion intervention aimed at improving the mental, emotional, and physical well-being of cancer survivors. Tested in a Phase 2 clinical trial involving 104 adult cancer survivors, the program demonstrated significant reductions in mental distress at both six- and 12-month follow-ups. PC-PEP integrates evidence-based practices such as physical activity, dietary guidance, stress management, and relationship enhancement, with deeper psychological and spiritual exploration.

A unique component of the program involves weekly five- to eight-minute videos based on *“Love is Letting Go of Fear”* by Gerald Jampolsky, MD, which draws from the non-dual spiritual teachings of *A Course in Miracles*. These videos, presented by the program Leads (an Oncologist and a Research Scientist), introduce principles such as “I choose to see the lamp, not the lampshade”, using clinical and personal examples to encourage participants to apply the teachings in their daily lives.

### 158.2. Methodology or Methods

Participants in PC-PEP received daily emails with ‘PEP’ videos, promoting healthy lifestyle habits alongside the optional Sunday spiritual videos. The program incorporated:

- **Behavioral Components:** Exercise routines, strength training, dietary advice, relaxation techniques, and habit formation.
- **Social Support:** Monthly videoconferences and a buddy system to maintain engagement and foster a sense of community.
- **Spiritual Exploration:** Weekly short videos discussing spiritual principles, with relatable examples from clinical and personal experiences shared by the program Leads.

This workshop replicates the experiential aspect of PC-PEP by combining didactic learning about the program outcomes and principles with group discussions, encouraging participants to explore how these teachings can be applied in their own personal and professional contexts.

### 158.3. Impact on Practice or Results

#### 158.3.1. Results

PC-PEP significantly improved mental distress among participants at six and 12 months. The optional spiritual programming added depth to the intervention, fostering reflection and personal growth. Participants reported feeling supported not only physically but also emotionally and spiritually, highlighting the holistic benefits of the program. Engagement with the spiritual principles further enhanced participants’ abilities to navigate fear, uncertainty, and emotional challenges associated with cancer survivorship.

#### 158.3.2. Impact on Practice

This workshop demonstrates how integrating spiritual teachings within a health-promotion program can enhance mental and emotional well-being in cancer survivors. While secular healthcare systems may perceive challenges in implementing spiritual programming, this experience shows that such content can be delivered in an accessible, inclusive, and non-dogmatic way. Healthcare providers and researchers can explore similar approaches to support patients’ psychological and emotional needs holistically, while maintaining cultural and religious sensitivity. The workshop also offers practical guidance on incorporating these principles into professional and personal practices, fostering resilience, compassion, and deeper connections in caregiving roles.

### 158.4. Discussion or Conclusions

The *“Love is Letting Go of Fear”* component of PC-PEP serves as a powerful example of how spiritual principles can complement evidence-based interventions in cancer care. By addressing the emotional and spiritual dimensions of survivorship, this program provides a replicable model for enhancing quality of life in a holistic manner. Participants in this workshop will leave with actionable insights, tools, and reflections to enrich their personal and professional lives, while considering the broader implications of incorporating spiritual teachings into secular healthcare environments.

## 159. Final Category: O. Survivorship

**14**

## 160. Implementation of Cognitive-Behavioral Therapy for Insomnia in Cancer Care: Preliminary Results of the IMPACT Program

Josée Savard ^1,2,3^, Émilie Godin ^2,3^, Aude Caplette-Gingras ^2,4^, Charles M. Morin ^1,5^, Lynda Bélanger ^4^, Marie-Pierre Gagnon ^1^, Marie-Ève Lamontagne ^1^

1. Université Laval, Québec, Canada.
2. CHU de Québec-Université Laval Research Center, Québec, Canada.
3. Université Laval Cancer Research Center, Québec, Canada.
4. CHU de Québec-Université Laval, Québec, Canada.
5. CERVO Brain Research Centre, Québec, Canada.

### 160.1. Background/Rationale or Objectives/Purpose

The main goals of the IMPACT (Insomnia in Patients with Cancer—Personalized Treatment) program are to assess the feasibility and efficacy of implementing a stepped care cognitive-behavioural therapy for insomnia (CBT-I), combining a web-based intervention and 1-3 booster therapy sessions, in four cancer centers in Quebec City.

### 160.2. Methodology or Methods

The ongoing study uses a stepped wedge cluster non-randomized design implementing the stepped care CBT-I at various time intervals across cancer centers. Patients having a score^3^ 4 on the sleep item of the Edmonton System Assessment System-Revised (ESAS-R-sleep) receive a leaflet explaining the stepped care CBT-I and how to access the web-based program (first step). Patients with residual insomnia symptoms after completing this first step are offered 1-3 booster sessions with a clinical psychologist (second step). Uptake and retention rates are the main variables.

### 160.3. Impact on Practice or Results

Across cancer centers, 11.9–50.8% of patients having a score of 4 or greater on the ESAS-R-sleep item were referred to the IMPACT program. The most common reasons for not referring were: (1) the presence of comorbid anxiety or depression symptoms (they were referred instead to the psychosocial oncology team) and; (2) patients declining help. Registration rates were greater when a brief follow-up phone call was conducted to explain the IMPACT program in more detail (vs. giving only the leaflet). Across cancer centers, 10–54.9% of referred patients registered with the web-based CBT-I, 83.3–90% of them initiated it and 26–42.3% completed it. The most common reasons for not completing the web-based CBT-I were: (1) sleep difficulties improved or remitted (36.2%); and (2) the program did not meet patients’ needs (23.4%). Among completers, 29–56% had residual insomnia and were offered booster sessions. On average, sleep diary data collected during treatment (N = 83) indicated a reduction of sleep-onset latency of 20 min and of wake after sleep onset of 45 min. Sleep efficiency increased from 70 to 85%.

### 160.4. Discussion or Conclusions

Although uptake and retention rates could be improved, these preliminary data suggest that a stepped care CBT-I can be implemented in routine cancer care and is effective.

**33**

## 161. Individual Variability in Psychosocial Outcomes Among Women with Gynecologic Cancer Who Participated in a Co-Created Yoga Program: A Series N-of-1 Trial

Jenson Price ^1^, Soulene Sabir ^2^, Brooklyn Westlake ^3^, Jennifer Brunet ^3^

1. Queen’s University, Kingston, Canada.
2. Western University, London, Canada.
3. University of Ottawa, Ottawa, Canada.

### 161.1. Background/Rationale or Objectives/Purpose

A community partnership yielded a co-created yoga program for women diagnosed with gynecologic cancer with key patient-reported outcomes identified to evaluate upon implementation. This study aimed to investigate individual variability in psychosocial responses to the co-created yoga program.

### 161.2. Methodology or Methods

Twenty women diagnosed with gynecologic cancer (*M*_age(years)_ = 62.4, *M*_years since diagnosis_ = 7.6) were recruited from The Ottawa Hospital (Ontario). A multiple baseline series N-of-1 A_1_BA_2_ trial design was used. Participants completed a 12-week bi-modal Hatha yoga program (morning or evening) with optional group discussions, journals, and pre-recorded videos for at-home practice. Self-report data on quality of life, fatigue, cognitive abilities, body image, sexual distress, and stress were collected prior to (A_1_; 3–5 timepoints), during (B; 3 timepoints), and after the program (A_2_; 3 timepoints).

### 161.3. Impact on Practice or Results

Based on visual analysis, individual variability was present; however, patterns were observable across participants. Quality of life showed stability during A_1_, improved during B, and initially declined at A_2_ before gradually increasing. Cognitive abilities remained steady during A_1_, slightly improved during B, and stabilized during A_2_. Fatigue was stable during A_1_ and B but worsened during A_2_. Sexual distress showed improvement across phases, while negative body image remained stable. Stress was stable across phases for the morning group, while the evening group showed stability during A_1_, improvement during B, and worsened during A_2_.

### 161.4. Discussion or Conclusions

A co-created 12-week Hatha yoga program demonstrated sustained improvements in psychosocial outcomes. The diverse responses highlight the need for personalized approaches to providing supportive care in physical activity settings.

**43**

## 162. Fear of Cancer Recurrence Workshop: Application of Acceptance and Commitment Therapy (ACT) as a Way of Coping with Fear of Cancer Recurrence (FCR)

Chelsea Butler, Simran Chhina, Sandra Peláez

Colorectal Cancer Canada, Montreal, Canada.

### 162.1. Background/Rationale or Objectives/Purpose

Fear of Cancer Recurrence (FCR) is one of the most reported challenges that patients in survivorship face. Currently, there are few approaches that specifically target fear of cancer recurrence, especially colorectal cancer specific. Acceptance and Commitment Therapy (ACT) has been successful in addressing FCR associated with other Cancer types. It has not been evaluated in application to colorectal cancer.

### 162.2. Methodology or Methods

An 8-week intervention support group was created using ACT. The purpose of the intervention is to provide coping strategies and ways to manage FCR to facilitate living a rich meaningful life according to one’s values. Through interactive group discussion and activities, each of the eight sessions explore a specific topic aligned with ACT. These sessions deal with coping strategies, values, defusion, acceptance, observing self, and value-based action. Outcomes aim to increase psychological flexibility and the ability to cope with FCR.

### 162.3. Impact on Practice or Results

Evidence indicates that ACT is shown to empower patients to practice coping strategies and ways of managing FCR which can lead to a higher quality of life. ACT is shown to reduce anxiety, improve psychological flexibility, address uncertainty, and provide a way to self-manage unhelpful thoughts, feelings, emotions, and behavior.

### 162.4. Discussion or Conclusions

The program is in the pilot phase, currently under evaluation. Patients’ and healthcare professionals’ perspectives are being collected to improve the content and delivery. Adjustments will be completed, and the following round will be devoted to exploring the efficacy of ACT when applied to FCR amongst the Colorectal Cancer community.

**65**

## 163. Hot Flash Interference and Insomnia Severity in Women Being Treated for Breast Cancer

Payton Peach ^1,2^, Emily White ^1^, Rachel Lee ^1^, Maria Baker ^1^, Sheila Garland ^1,2^

1. Department of Psychology, Faculty of Science, Memorial University of Newfoundland, St. John’s, Canada.
2. Discipline of Oncology, Faculty of Medicine, Memorial University of Newfoundland, St. John’s, Canada.

### 163.1. Background/Rationale or Objectives/Purpose

Hot flashes are one of the most severe and prevalent menopausal symptoms experienced by women following breast cancer treatment. Many breast cancer survivors report nighttime awakenings and difficulty falling back asleep due to hot flashes. This study investigates the relationship between hot flashes and insomnia symptom severity in women being treated for breast cancer.

### 163.2. Methodology or Methods

This prospective observational cohort study examined 100 women with newly diagnosed breast cancer (26 premenopausal and 74 postmenopausal), recruited prior to receiving chemotherapy, radiation therapy, and hormone therapy. Out of 99 participants that received hormone therapy, 76 completed all questionnaires and were included in the analyses. Hot flash interference and insomnia severity were measured using the Hot Flash Related Daily Interference Scale (HFRDIS) and Insomnia Severity Index (ISI), respectively. A repeated measures ANOVA with post-hoc paired comparisons was performed to examine differences in HFRDIS scores from baseline to each 4-, 8-, and 12-month follow-ups. Correlations were performed to examine whether HFRDIS scores were related to ISI scores at the corresponding time points.

### 163.3. Impact on Practice or Results

Hot flash interference significantly increased from baseline (*M* = 8.80) to 4 months (*M* = 17.43, *p* = 0.001), baseline to 8 months (*M* = 21.70, *p* < 0.001), and baseline to 12 months (*M* = 18.03, *p* < 0.001). HFRDIS and ISI were found to be significantly positively correlated at baseline (*r*(74) = 0.56, *p* < 0.001), 4 months (*r*(74) = 0.55, *p* < 0.001), 8 months (*r*(74) = 0.53, *p* < 0.001), and 12 months (*r*(74) = 0.52, *p* < 0.001).

### 163.4. Discussion or Conclusions

Hot flash interference significantly increased within the first year after breast cancer diagnosis among women receiving hormone therapy. Greater hot flash interference was associated with greater insomnia severity, highlighting the impact of menopausal symptoms on breast cancer survivors’ quality of life.

**66**

## 164. Feasibility and Acceptability of an In-Home Polysomnography Device to Measure Objective Sleep Response in CBT-I

Emily White ^1^, Heath Matheson ^1^, Benjamin Rich Zendel ^2^, Sheila Garland ^1,3^

1. Department of Psychology, Faculty of Science, Memorial University, St. John’s, Canada.
2. Division of Population Health and Applied Health Sciences, Faculty of Medicine, Memorial University, St. John’s, Canada.
3. Discipline of Oncology, Faculty of Medicine, Memorial University, St. John’s, Canada.

### 164.1. Background/Rationale or Objectives/Purpose

Cognitive Behavioural Therapy for Insomnia (CBT-I) is the recommended treatment for insomnia disorder, but there is limited and inconsistent research using home-based polysomnography to assess treatment outcome. This pilot study assessed the feasibility of using the Cerebra Sleep System (CSS; an in-home polysomnography device) to measure objective sleep response to seven weeks of CBT-I.

### 164.2. Methodology or Methods

Women diagnosed with breast cancer residing in the St. John’s area who completed primary treatments and met the diagnostic criteria for insomnia disorder were eligible for the study. Treatment consisted of seven weekly sessions of CBT-I. Before and after treatment, participants used the CSS for one night to objectively measure their sleep. Recruitment, retention, and exit interviews were used to gain more information on the feasibility and acceptability of using the CSS.

### 164.3. Impact on Practice or Results

Out of 49 individuals screened, 19 were deemed eligible. Of those, four did not enroll, six withdrew (four due to medical or family reasons), six completed the study, and three are actively in treatment. The current retention rate is 60%. Out of 20 overnight sleep assessments, two had to be redone. Zero participants contacted for additional assistance in using the device. From exit interviews, participants felt that their sleep improved and themes addressing use of the CSS will be discussed.

### 164.4. Discussion or Conclusions

Objective sleep measures may help to understand mechanisms underlying the benefits of CBT-I but challenges in recruitment and retention remain.

Questions: Given the noted concerns with feasibility, what changes could be implemented for a large scale RCT?

**70**

## 165. An Evaluation of Follow-Up Care Practices for Northern Ontario Adult Childhood Cancer Survivors

Joshua McGonegal, Nancy Lightfoot, Emily Donato, Sandra Hoy

Laurentian University, Sudbury, Canada.

### 165.1. Background/Rationale or Objectives/Purpose

This study will evaluate if and how follow-up care practices could be improved for adult childhood cancer survivors (ACCS) who reside in northern Ontario and access follow-up care through the Pediatric Oncology Group of Ontario (POGO). We will adhere to Cancer Care Ontario’s definition of a follow-up care plan requiring surveillance of signs and symptoms of cancer recurrence, its long-term and late effects, and a person’s developing psychosocial needs.

### 165.2. Methodology or Methods

#### 165.2.1. Sample and Setting

20 ACCS residing in northern Ontario, 19 years or older, will be recruited to discuss their experiences receiving follow-up care delivered by the POGO. The study will also include the perspectives of eight healthcare providers (HCPs) who provide follow-up care to this population.

#### 165.2.2. Procedures

A qualitative descriptive study design, semi-structured interviews, and reflexive thematic analysis will be utilized.

### 165.3. Impact on Practice or Results

The resulting themes will reflect the perspectives of these ACCS and the HCPs responsible for their care through the POGO.

### 165.4. Discussion or Conclusions

We hope our results will improve the delivery and availability of health services for northern Ontario ACCS.

**Questions**

1. The lead author of this study is an adult childhood cancer survivor. What might be done to ensure reflexivity throughout the research process?
2. How might strategies to prepare and conduct interviews with adult childhood cancer survivors differ from those with healthcare providers?
3. Do you have any suggestions on how this study’s results can best be shared with the cancer community?

**79**

## 166. The Creation and Validation of a Perceptual Measure for Medical Assistance in Dying for Cancer Survivors (PMAID): Results from Qualitative Data Analysis

Chloe House ^1,2^, Chris Quinn-Nilas ^1^, Emily Fawcett ^1^, Sheila N. Garland ^1,2,3^

1. Department of Psychology, Faculty of Science, Memorial University of Newfoundland, St. John’s, Canada.
2. Beatrice Hunter Cancer Research Institute, Halifax, Canada.
3. Discipline of Oncology, Faculty of Medicine, Memorial University of Newfoundland, St. John’s, Canada.

### 166.1. Background/Rationale or Objectives/Purpose

Over 13,000 Canadians received MAID in 2022. Of those individuals, 63% had a terminal cancer diagnosis. Opinions of MAID have been collected from many sources, but less is known about how people who are not at end-of-life view MAID. This study proposes the development of a perceptual measure that may facilitate clinical MAID discussions and quantify beliefs and opinions for researchers.

### 166.2. Methodology or Methods

Qualitative interviews with cancer survivors to understand their perceptions of MAID are currently underway. Novel and frequent themes will be added to a concept map that will later be built into an initial item pool. Cancer survivors’ perceptions of MAID will be analyzed with thematic analysis. Prominent or representative codes will be added to a concept map and help build the initial item pool for The Perceptions of MAID (PMAID) instrument.

### 166.3. Impact on Practice or Results

Preliminary findings suggest survivors suggest that (1) survivors feel suffering is worse than death; (2) they may be unsure about choosing MAID if major life/family events are coming; (3) feel comfort seeking MAID knowing they have control to opt out of MAID at any time.

### 166.4. Discussion or Conclusions

PMAID aims to improve the ease of clinical discussions centered around MAID by identifying cancer survivors’ opinions, concerns, and needs and adding quantifiability of perceptions of MAID in research contexts.

How would you see this tool being used, and would you find this tool useful for MAID discussions in your practice?

Would you be comfortable taking or administering this instrument?

**82**

## 167. Assessing the Relationship Between Quality of Life and Parent/Child Symptoms of Posttraumatic Stress, Posttraumatic Growth, and the Benefits/Burdens of Cancer in Survivors of Childhood Cancer

Amanda Button ^1^, Caitlin Forbes ^1^, Michaela Patton ^1^, Kathleen Reynolds ^2,1^, Fiona Schulte ^1,2^

1. University of Calgary, Calgary, Canada.
2. Alberta Health Services, Calgary, Canada.

### 167.1. Background/Rationale or Objectives/Purpose

Survivors of childhood cancer (SCC) and their parents frequently face traumatic experiences throughout their treatment. Our aim was to assess symptoms of posttraumatic stress, posttraumatic growth, and the benefits/burdens of cancer, and examine their relationship with quality of life (QoL) in SCC.

### 167.2. Methodology or Methods

Sample and Setting: SCC were ≥2 years post-treatment or ≥5 years post-diagnosis and currently aged 8–17. (N = 62; *M_Current age_* = 12.76 years, (SD = 2.95); *M_Age at Diagnosis_* = 4.48 years (SD = 3.24); 51.3% female)

Procedures: Participants were recruited from a survivorship clinic, social media, and community organizations. Clinical and demographic variables were collected. Quality of life was measured through the Pediatric Quality of Life (PedsQL) generic-core (self-report). Survivors completed the Child Posttraumatic Stress Scale (CPSS) and the Benefit-Burden Scale for Children (BBSC). Parents completed the self-report Posttraumatic Growth Inventory (PTGI) and PTSD Checklist for DSM-5(PCL-5). Variables that were correlated with QoL were included in a linear regression

### 167.3. Impact on Practice or Results

Self-reported QoL (*M* = 75.30, (SD = 15.64)) was below PedsQL healthy norms (*p* < 0.001) and was correlated with BBSC Burden (r(69) = −0.395, *p* < 0.001), CPSS Severity (r(70) = −0.601, *p* < 0.001), CPSS Impairment (r(70) = −0.402, *p* < 0.001), PCL-5 total (r(64) = −0.258, *p* = 0.040), PCL-5 Cluster D (r(64) = −0.269, *p* = 0.031), and PCL-5 Cluster E (r(63) = −0.257, *p* = 0.042). The regression model was significant (F(6,55) = 3.707, *p* < 0.004, *R*/ = 0.288) with CPSS severity totals emerging as a significant predictor of QoL (*p* = 0.009).

### 167.4. Discussion or Conclusions

The trauma that SCC’s face result in cancer burden, as well as parent and SCC posttraumatic stress. Parent and child mental wellbeing should be prioritized as an intervention target for positive QoL.

**85**

## 168. Fertility Discussions Prior to Treatment Initiation in Adolescents and Young Adults with Cancer: A Retrospective Analysis

Khulood Hasnain ^1^, Sapna Oberoi ^2,3,4^, Madyson Shauman ^5^, Mackenzie Jansen ^5^, Iresha Ratnayake ^6^, Lin Xue ^6^, Oliver Bucher ^6^, Cielle Stapleton ^2,3,4^

1. Rady Faculty of Health Sciences, University of Manitoba, Winnipeg, Canada.
2. Department of Pediatric Hematology/Oncology, CancerCare Manitoba, Winnipeg, Canada.
3. Department of Pediatrics and Child Health, Max Rady College of Medicine, University of Manitoba, Winnipeg, Canada.
4. CancerCare Manitoba Research Institute, CancerCare Manitoba, Winnipeg, Canada.
5. Department of Patient and Family Support Services, CancerCare Manitoba, Winnipeg, Canada.
6. Department of Epidemiology and Cancer Registry, CancerCare Manitoba, Winnipeg, Canada.

### 168.1. Background/Rationale or Objectives/Purpose

Adolescent and Young Adult (AYA) patients with cancer are at risk of infertility due to cancer and its treatment, substantially impacting their quality of life. Our primary objective was to assess the proportion of AYAs with documented fertility discussions before initiating treatment and to identify factors associated with receiving these discussions. Secondary objectives included determining the proportion of patients referred to a fertility clinic and those undergoing fertility preservation procedures.

### 168.2. Methodology or Methods

A retrospective chart review of patients aged 15 to 39 newly diagnosed with cancer at CancerCare Manitoba in 2022 was conducted. Data extracted included sociodemographic characteristics, cancer and treatment details, and fertility outcomes. Descriptive analyses summarized the data. Univariable and multivariable regression analyses determined the factors associated with fertility discussions.

### 168.3. Impact on Practice or Results

Of 259 eligible patients (mean age: 32 years), 65% were female. Solid tumours were predominant (86%), and 31% of patients received systemic cancer treatment. Fertility discussions were documented for 39% of patients before initiating therapy and for 42% at some time in treatment. Of those having fertility discussions, 24% were referred to a fertility clinic, and 14% underwent fertility preservation procedures. On multivariable regression, systemic cancer treatment and consultations with medical or gynecological oncologists were associated with increased odds of fertility discussion before treatment, whereas age, sex, cancer type, residence, and provider sex were not associated.

### 168.4. Discussion or Conclusions

Fertility discussions, documented for 39% of AYAs before treatment and occurring more frequently in medical and gynecology oncology, underscore the need for standardizing fertility discussions across various oncology specialties to ensure equitable oncofertility care.

**86**

## 169. Adaptive Functioning in Survivors of Childhood Cancer and Healthy Controls

Mehak Stokoe, Jenny Duong, Caitlin Forbes, Heide Adham, David Nordstokke, Gabrielle Wilcox, Fiona Schulte

University of Calgary, Calgary, Canada.

### 169.1. Background/Rationale or Objectives/Purpose

Adaptive functioning (AF) is comprised of behaviors that are utilized in daily living such as reading, interpersonal skills, personal care. Survivors of childhood cancer (SCC) may experience treatment-related impacts on their AF due to treatment directed at the central nervous system, which can impact their cognitive development, and subsequently influence the acquisition of AF skills. The objective of this study is to examine group differences in AF among survivors of acute lymphoblastic leukemia (ALL), non-central nervous system solid tumors (non-CNS ST), and brain tumors (BT), and healthy controls (HC).

### 169.2. Methodology or Methods

Survivors of ALL, non-CNS ST and BT, and HC, as well as a parent or caregiver were invited to complete a study visit at the Alberta Children’s Hospital. Parents completed the Adaptive Behavior Assessment System (3rd edition; ABAS-3) as part of the study visit. Group differences between survivors and HC were evaluated through a Multivariate Analysis of Variance (MANOVA).

### 169.3. Impact on Practice or Results

A total of 117 families participated in this study, with 28 survivors of ALL, 21 survivors of ST, 33 survivors of BT, and 35 HC. Mean age at diagnosis for survivors was 4.53 (SD = 3.59) years. Results of the MANOVAs revealed significant main effects for the conceptual, social, and practical domains and significant difference between groups on conceptual (*F*(3, 111) = 6.48, *p* < 0.001), social (*F*(3,111) = 7.36, *p* < 0.001), and practical domain scores (*F*(3,111) = 5.63, *p* < 0.001). Parents rated survivors of BT significantly lower than HC on all domains (*p <* 0.001), and parents rated survivors of ALL significantly lower than HC on the conceptual domain only (*p* = 0.017). No significant differences were found between non-CNS ST and HC.

### 169.4. Discussion or Conclusions

School and healthcare providers should monitor the adaptive skills of survivors of childhood cancer, especially for survivors of BT and ALL. Gaining a deeper understanding of factors influencing AF in these groups of survivors may help inform targeted interventions to improve skills across AF domains.

**93**

## 170. Oral Health Deficits and Frailty in Head and Neck Cancer Survivors—An Interim Analysis

Violet D’Souza ^1,2,3^, Shauna Hachey ^1^, Rebecca Affoo ^1,4^, Yuqi Wang ^1^, Pierre-Luc Michaud ^1^, Debora Matthews ^1^, Mary McNAlly ^1^, Olga Theou ^5^, Kenneth Rockwood ^6,7^, Mark Taylor ^8^

1. Faculty of Dentistry, Dalhousie University, Halifax, Canada.
2. Department of Surgery, Faculty of Medicine, Dalhousie University, Halifax, Canada.
3. Department of Community Health and Epidemiology, Dalhousie University, Halifax, Canada.
4. School of Communication Sciences and Disorders, Faculty of Health, Dalhousie University, Halifax, Canada.
5. School of Physiotherapy, Dalhousie University, Halifax, NS, Canada.
6. Division of Geriatric Medicine, Dalhousie University, Halifax, NS, Canada.
7. School of Health Administration, Dalhousie University, Halifax, NS, Canada.
8. Division of Otolaryngology–Head & Neck Surgery, Dalhousie University, Halifax, NS, Canada.

### 170.1. Background/Rationale or Objectives/Purpose

Head and neck cancer survivors (HNCS) often suffer from long-term sequelae related to their cancer and its treatment. Oral physiology and functions are often altered, affecting their overall well-being. Aim: To assess the oral health status, oral functions, and their impact on the overall well-being of HNCS.

### 170.2. Methodology or Methods

In this cross-sectional study conducted at Dalhousie University, participating HNCS underwent oral exams and were screened for frailty using the Clinical Frailty Scale. Also, their oral health-related quality of life (OHRQoL) was assessed using the OHIP-14, swallowing function using the MD Anderson Dysphagia Inventory, and chewing function using the Chewing Function Questionnaire. Data were analyzed to calculate the prevalence and severity of oral health problems and their association with participants’ overall well-being.

### 170.3. Impact on Practice or Results

This interim analysis includes data from 42 HNCS (74% male). The mean age was 69 years. Approximately 24% had at least one decayed tooth, and 31% had moderate to severe periodontal disease. The mean (SD) number of missing teeth was 9.4 (±9.6), and the mean (SD) number of posterior teeth in function was 4.9 (±2.7). The OHRQoL was poorer in those who received radiation and those who experienced chewing and swallowing difficulty (*p* < 0.001). The mean frailty score was 2.1 (±1.0), indicating mild functional decline but not associated with oral health status or functions.

### 170.4. Discussion or Conclusions

Our study findings suggest that HNCS experience significant oral health problems that affect their well-being. Systematic monitoring of HNCS to identify, prevent, and address oral health problems may improve the overall well-being of HNCS.

**95**

## 171. The Weight of Perfection: How It Shapes Psychological Adjustment to Breast Cancer

Claudia Mc Brearty ^1,2,3^, Catherine Banville ^1,2,3^, Véronique Massicotte ^1,2,3^, Aude Caplette-Gingras ^2,4^, Frédérique Langlois ^5^, Jean-Philippe Gouin ^6^, Gordon Flett ^7^, Danielle S. Molnar ^8^, Julie Lemieux ^2,4^, Josée Savard ^1,2,3^

1. School of Psychology, Université Laval, Québec, Canada.
2. CHU de Québec-Université Laval Research Center, Québec, Canada.
3. Université Laval Cancer Research Center, Québec, Canada.
4. Centre des maladies du sein, Québec, Canada.
5. Department of Psychology, Université du Québec à Trois-Rivières, Trois-Rivières, Canada.
6. Department of Psychology, Concordia University, Montréal, Canada.
7. Faculty of Health, York University, Toronto, Canada.
8. Department of Child and Youth Studies, Brock University, Saint Catharines, Canada.

### 171.1. Background/Rationale or Objectives/Purpose

A cancer diagnosis is associated with significant psychological challenges, which specific personality traits can further exacerbate. Perfectionism is a trait associated with various psychosocial difficulties in the general population. However, the psychological impact of perfectionism on individuals with cancer remains to be assessed. This exploratory qualitative study aimed to examine the role of perfectionism as a vulnerability factor underlying the psychosocial difficulties experienced by women with breast cancer.

### 171.2. Methodology or Methods

Ten women with nonmetastatic breast cancer who self-identified as perfectionists were recruited after completing radiation therapy. Participants participated in either a focus group or an individual interview. Verbatim were transcribed and analyzed using NVivo.

### 171.3. Impact on Practice or Results

An hybrid inductive-deductive thematic analysis revealed the following themes: (1) various dimensions of perfectionism; (2) low self-compassion; (3) control seeking; (4) low self-disclosure; (5) low social support seeking; (6) various coping strategies; (7) relative stability of perfectionism after cancer; (8) need to be a perfect patient; (9) negative psychological impacts; and (10) low interest in an intervention targeting perfectionism.

### 171.4. Discussion or Conclusions

This exploratory study highlights the complex psychosocial issues associated with perfectionism among breast cancer patients, emphasizing both systemic and individual dynamics. While perfectionism gave some patients a sense of control and purpose, it was perceived as being associated with heightened stress, hindered social support, and as contributing to exhaustion. These findings underscore the potential value of targeted psychological interventions, particularly those focusing on self-compassion and psychological flexibility.

**129**

## 172. Short-Term Success, Long-Term Challenges: Piloting an Exercise Program for Women Who Have Completed Breast Cancer Treatment

Jenson Price ^1^, Kaitlyn Kauffeldt ^1^, Amy Latimer-Cheung ^1^, Angela Fong ^2^, Jennifer Tomasone ^1^

1. Queen’s University, Kingston, Canada.
2. University of Michigan, Michigan, USA.

### 172.1. Background/Rationale or Objectives/Purpose

A co-designed 12-week exercise program for women who have completed breast cancer treatment was piloted to evaluate its benefits and suitability for integration into an existing community-based exercise program.

### 172.2. Methodology or Methods

Nine participants (*M*_age_ = 56.98 ± 6.39) enrolled in the exercise program in Kingston, Ontario. Certified exercise professionals delivered the program, which incorporated tailored exercise sessions in a group setting, educational sessions, and behavior change techniques aligned with self-determination theory. A single-arm trial design was used to assess the exercise program. Participants completed a post-program focus group (*n* = 7) to determine whether program features were congruent with their values, expectations, and preferences. The focus group transcript was analyzed using content analysis. Pragmatic discussions were held between the certified exercise professionals, community program coordinator and co-directors to determine program sustainability.

### 172.3. Impact on Practice or Results

Four main themes were identified from the focus group: (1) a tailored journey of personalized support and empowering relationships, (2) reclaiming strength through physical recovery and emotional growth, (3) building momentum with group dynamics and accountability, (4) refining the experience with better communication and accessibility. Pragmatic discussions revealed human, financial, and structural resources as well as research priority alignment with the existing community-based exercise program as challenges to sustainability.

### 172.4. Discussion or Conclusions

While the exercise program showed initial success in engaging participants and improving perceived physical and psychological functioning, long-term sustainability was not achieved. Key challenges and lessons learned will be shared to guide future efforts in designing sustainable community-based interventions for women who have completed breast cancer treatment.

**131**

## 173. An Interpretive Description of Quality of Life After Treatment of Relapsed and Refractory B-Cell Lymphoma

Samantha Mayo ^1,2^, Jeus Cabaluna ^3^, Stacey Morrison ^1^, Shanelle Racine ^1^, Medha Surajpal ^1^, Anca Prica ^1,2^, John Kuruvilla ^1,2^

1. Princess Margaret Cancer Centre, Toronto, Canada.
2. University of Toronto, Toronto, Canada.
3. University Health Network, Toronto, Canada.

### 173.1. Background/Rationale or Objectives/Purpose

People with relapsed or refractory lymphoma (RRL) do poorly with standard chemotherapy. Expanded options with autologous stem cell transplant (ASCT) or chimeric antigen receptor T-cell (CAR-T) therapy offer enhanced survival benefit, but also potential physical and psychosocial impacts. This study explores survivors’ experiences and perceived impacts of RRL treatment on quality of life.

### 173.2. Methodology or Methods

Using an interpretive description approach, qualitative semi-structured interviews are being conducted as part of a mixed methods study of quality of life after RRL treatment. Participants are adults, ≥6 months post-ASCT or CAR-T for RRL, and in remission. Interviews focus on participants’ experiences with RRL treatment and its impact on physical, psychological, and social aspects of life. Common themes will be generated using thematic analysis, with rigor supported by investigator triangulation, an audit trail, and debriefing with patient partners. Anticipated sample size is 15–20 participants.

### 173.3. Impact on Practice or Results

Twelve interviews have been completed to date. Participants, with a median age of 62 years (range 54–76), were predominantly male (66%), white (92%), and treated with ASCT (66%). Mean time since treatment was 1.52 years. Preliminary findings indicate that participants feel fortunate to have received treatment but face functional changes influencing quality of life. Family support, social connections, and one’s sense of resilience are central to their experience.

### 173.4. Discussion or Conclusions

Patients treated for RRL perceive their quality of life positively, largely depending on their capacity to cope with physical and psychosocial challenges. Additional data collected in this ongoing study will expand these findings. Tailored prehabilitation and supportive care interventions may contribute to long-term well-being in this growing group of survivors.

**132**

## 174. Beyond Survival: Mental Health, Executive Functioning, and Pain in Survivors of Acute Lymphoblastic Leukemia and Their Siblings

Emma Houghton ^1^, Brooke Russell ^2^, Caitlin Forbes ^3^, Oluwaseyi Lawal ^3^, Lianne Tomfohr-Madsen ^4^, Fiona Schulte ^2,3^

1. Department of Psychology, University of Calgary, Calgary, Canada.
2. Hematology, Oncology, Transplant Program, Alberta Children’s Hospital, Calgary, Canada.
3. Department of Oncology, Division of Psychosocial Oncology, Cumming School of Medicine, University of Calgary, Calgary, Canada.
4. Department of Educational and Counselling, and Special Education, University of British Columbia, Vancouver, Canada.

### 174.1. Background/Rationale or Objectives/Purpose

Survivors of acute lymphoblastic leukemia (ALL) may experience poorer mental health, executive function, and pain. This study examines these variables by comparing survivors with controls and survivor siblings with control siblings to investigate shared familial impacts and identify intervention targets.

### 174.2. Methodology or Methods

Participants included survivors of ALL (n = 45, mean age = 11.53 [SD = 2.50], 53% male), survivor siblings (n = 25, mean age = 12.96 [SD = 3.34], 60% male), controls (n = 45, mean age = 11.42 [SD = 2.45], 53% male) and control siblings (n = 41, mean age = 11.10 [SD = 2.27], 68% male). Data were collected online via Qualtrics.

Survivor families were recruited from a long-term survivor clinic, and control families via posters/pamphlets/social media. Anxiety and depression were assessed through the Behaviour Assessment System for Children (BASC-2), executive functioning through the Behaviour Rating Inventory of Executive Functioning (BRIEF), and chronic pain through a yes/no item. T-scores > 60 indicate at-risk levels on the BASC and BRIEF. Independent samples t-tests were completed to compare between-group means.

### 174.3. Impact on Practice or Results

Anxiety was significantly higher in survivors (M = 51.02, SD = 9.89), compared to controls (M = 44.93, SD = 9.21), t(83) = 2.94, *p* = 0.002. Similarly, survivors had greater depression (M = 50.02, SD = 8.66) than controls (M = 46.20, SD = 7.83), t(83) = 2.14, *p* = 0.018. Survivors exhibited more executive functioning deficits (M = 51.49, SD = 10.58) than controls (M = 47.40, SD = 11.59). Survivors’ siblings had greater depression (M = 52.42, SD = 11.88) than control siblings (M = 46.03, SD = 8.35), t(57) = 0.269, *p* = 0.010. All other variable comparisons were non-significant.

### 174.4. Discussion or Conclusions

These findings underscore the importance of routine screening and targeted interventions for both survivors and their siblings to address anxiety, depression, and executive functioning deficits.

**134**

## 175. Informed and Empowered: Evaluating a Patient Education Video on Sexual Health (SH) Needs in Adolescents and Young Adults (AYA) with Cancer

Natalie Pitch ^1^, Anjali Sachdeva ^2^, Jennifer Catsburg ^3^, Sheila Gandhi ^1^, Rebecca Cote ^1^, Chana Korenblum ^1^, Jonathan Avery ^3^, Abha A. Gupta ^1^

1. SickKids Hospital, Toronto, Canada.
2. Temerty Faculty of Medicine, Toronto, Canada.
3. Princess Margaret Hospital, Toronto, Canada.

### 175.1. Background/Rationale or Objectives/Purpose

Female AYAs aged 15–39 with cancer may develop SH related challenges. To address the SH care gap, we developed a patient education white-board video explaining SH concerns associated with cancer. Our study aims to evaluate the video by assessing patients’ knowledge pre- and post- video and overarching impressions.

### 175.2. Methodology or Methods

Participants were recruited nationally through hospitals and cancer organizations. Inclusion criteria: female-assigned at birth aged 15–39, minimum one-month post-diagnosis. Participants completed online questionnaires before and after watching the video to assess knowledge, interest in SH, and impressions. Descriptive statistics, mean comparisons, and inductive coding of short answers were conducted.

### 175.3. Impact on Practice or Results

To date, 31 patients have been recruited (median age 28, range 15–39). Most common cancer diagnoses included leukemia (n = 11, 35%) and lymphoma (n = 4, 13%). Seventeen participants (55%) were actively receiving treatment. Sixteen (51%) reported never discussing SH with a healthcare provider; 29 (94%) felt learning about SH was important/very important. Nineteen (61%) reported their SH concerns were inadequately/not at all addressed. Mean knowledge test scores improved from 68% to 90% after watching the video (*p* < 0.01). Feedback of video strengths included the use of visuals and type of information; suggestions for improvement included shortening the length, using more age-appropriate content, and focusing more on psychosocial effects.

### 175.4. Discussion or Conclusions

This study reaffirms existing literature demonstrating an unmet need in supporting AYA’s SH concerns. Our video significantly increased participants’ SH knowledge, underscoring the value of educational videos in cancer care. We aim to increase recruitment to better understand the differences in how younger AYA perceive the video.

**140**

## 176. A Habit-Based Intervention to Reduce Sedentary Behaviour in Cancer Survivors: A Feasibility Study

Deema Khalaf, Linda Trinh

University of Toronto, Toronto, Canada.

### 176.1. Background/Rationale or Objectives/Purpose

Cancer survivors experience long-term treatment-related side effects such as poor quality of life (QoL) and physical function. Reducing sedentary behaviour (SED), low energy waking behaviours performed while seated or lying down, may help improve physical and mental well-being. Using habit formation to reduce SED is successful in other clinical populations, but its effect on cancer survivors is unknown. Habit is a cognitive process where a context triggers a specific behaviour, learned through repetition of the behaviour every time the context is encountered. Therefore, this study examines the feasibility of an 8-week habit-based intervention to reduce SED, and changes in physical function and QoL of cancer survivors.

### 176.2. Methodology or Methods

Cancer survivors will be randomized into an intervention group (weekly, tailored habit-based checklists to reduce SED) or control group (handouts containing movement guidelines and benefits of reducing SED). The intervention group will receive a Fitbit for reminders to break SED, and 1-on-1 remotely delivered, weekly behavioural counselling sessions for tailored tips to reduce SED.

### 176.3. Impact on Practice or Results

Feasibility will be measured through enrolment and measurement completion rates, loss to follow-up, adherence, adverse events, and patient satisfaction. SED will be measured using an inclinometer (ActivPAL). Physical function will be measured using the 30s sit-to-stand test. QoL will be measured using the Functional Assessment of Cancer Therapy–General and Short Form-12 questionnaires. Outcomes will be measured pre- and post-intervention.

### 176.4. Discussion or Conclusions

Study findings will inform larger randomized controlled trials to reduce SED in cancer survivors. This intervention may be a cost-effective and accessible option for cancer survivors to optimize their health.

**167**

## 177. Pain in Young People Treated with Hematopoietic Stem Cell Transplant for Sickle Cell Disease in Childhood: A Qualitative Examination

Simone Garneau ^1^, Hailey Zwicker ^1^, Perri Tutelman ^1^, Caitlin Forbes ^1^, Jenny Duong ^1^, Gregory Guilcher ^1,2^, Aisha Bruce ^3^, Nur El Huda Abu Saris, Fiona Schulte ^1,2^

1. University of Calgary, Calgary, Canada.
2. Alberta Children’s Hospital Research Institute, Calgary, Canada.
3. University of Alberta, Edmonton, Canada.

### 177.1. Background/Rationale or Objectives/Purpose

Objectives/purpose: Sickle cell disease (SCD) is a genetic disorder characterized by severe pain crises and health complications that negatively impact quality of life. Hematopoietic cell transplant (HCT) offers a potential cure; however, the physical and psychosocial challenges associated with HCT, such as pain, remain unknown. This study aimed to explore the pain experiences of youth who underwent HCT during childhood.

### 177.2. Methodology or Methods

Sample and setting: Eleven youth [age range = 13–22 years, female = 72.73%, time since transplant = 6.31 ± 4.24 years] who had undergone HCT for SCD and eleven parents (mothers = 90.91%) were recruited from the Alberta Children’s Hospital Hematology Clinic in Calgary, Alberta.

Procedures: A phenomenological qualitative study design was used. Youth and parents completed separate semi-structured interviews. Data were analyzed using reflexive thematic analysis.

### 177.3. Impact on Practice or Results

Results: Analyses generated three preliminary themes: (1) Pain is a changed experience for youth with SCD after HCT; (2) embracing a new normal; children and families strive to find “normalcy” while managing any lingering pain or late effects after HCT; and (3) experiences of pain after HCT are shaped by pre-transplant expectations.

### 177.4. Discussion or Conclusions

Conclusion and clinical implications: The pain experience for those who underwent HCT as a child is not only changed but intertwined with their journey towards a “new normal”. Additionally, their experiences of pain are influenced by their pre-transplant expectations. The findings of this study emphasize the ongoing presence of pain post-HCT and highlight the need for better support systems for youth and their families throughout, and after, HCT.

**168**

## 178. Living with Fear: A Qualitative Exploration of Fear of Progression in Advanced Lung Cancer Patients Undergoing Immunotherapy or Targeted Therapy

Malick Ouattara ^1^, Alanna Chu ^1^, Anne Leslie ^1^, Sara Moore ^1,2,3^, Cheryl Harris ^2^, Kimberly McMillan ^1,4,5^, Rinat Nissim ^6,7,8^, Christine McPherson ^1^, Sophie Lebel ^1^

1. University of Ottawa, Ottawa, Canada.
2. The Ottawa Hospital, Ottawa, Canada.
3. Ottawa Hospital Research Institute, Ottawa, Canada.
4. Children’s Hospital of Eastern Ontario Research Institute, Ottawa, Canada.
5. Canadian Palliative Care Nursing Association, Ottawa, Canada.
6. University of Toronto, Toronto, Canada.
7. University Health Network, Toronto, Canada.
8. The Princess Margaret Hospital, Toronto, Canada.

### 178.1. Background/Rationale or Objectives/Purpose

A growing number of people with advanced lung cancer are living longer because of immunotherapy (IMT) or targeted therapy (TT). Qualitative studies have revealed that these treatments can lead to significant uncertainty and for some, Fear of Cancer Progression (FoP). Research on FoP has focused mostly on people living with earlier stage disease and little is known about how FoP presents in chronic advanced disease. Thus the goal of the present study is to gain an in-depth understanding of FoP in advanced lung cancer patients receiving IMT and TT.

### 178.2. Methodology or Methods

This study is part of an ongoing mixed methods study, which involves the completion of an online survey on FoP and other psychological constructs (n = 80 out of the proposed 150). A subset of 20–30 participants with significant FoP scores (≥34 on the Fear of Progression Questionnaire) will be selected for qualitative interviews. Diversity factors such as age, racial identity, and gender will be considered in the selection of participants. Semi-structured interviews, lasting 40–90 min, will be conducted in-person or virtually, with the final sample size determined by data saturation.

### 178.3. Impact on Practice or Results

The qualitative data collected in the study will provide deeper insights into FoP and identify potential patterns in the data or disparities that quantitative analyses alone cannot contextualize.

### 178.4. Discussion or Conclusions

This study aims to improve patient-centred care and provide tailored support systems around FoP in advanced cancer patients while enhancing the psychosocial support provided to diverse lung cancer patient populations.

**180**

## 179. Fear of Cancer Progression in Patients with Advanced and Metastatic Lung Cancer Receiving Immunotherapy or Targeted Therapy: Preliminary Results

Anne Leslie ^1^, Alanna Chu ^2^, Cheryl Harris ^1^, Sophie Lebel ^2^, Kimberly McMillan ^2^, Christine McPherson ^2^, Rinat Nissim ^3^, Sara Moore ^4^, Veronika Huta ^2^

1. The Ottawa Hospital Research Institute, Ottawa, Canada.
2. University of Ottawa, Ottawa, Canada.
3. Princess Margaret Cancer Centre, Toronto, Canada.
4. The Ottawa Hospital Cancer Centre, Ottawa, Canada.

### 179.1. Background/Rationale or Objectives/Purpose

Lung cancer is the leading cause of cancer deaths in Canada. Immunotherapies (IO) and targeted therapies (TT) can be highly effective at prolonging survival in individuals with advanced or metastatic lung cancer. Qualitative studies suggest these individuals may experience uncertainty, fear of progression (FOP) and death anxiety due to the non-curative intent and variable response of the treatment. This mixed-methods study investigates these psychological experiences in this population.

### 179.2. Methodology or Methods

This study is recruiting participants (n = 150) with advanced or metastatic lung cancer receiving IO or TT. Participants complete validated quantitative measures of FOP, death anxiety, symptom burden, supportive care needs, and illness uncertainty. Demographic and medical information is also collected. Qualitative interviews (n = 30) explore the relationships between these constructs.

### 179.3. Impact on Practice or Results

Study recruitment is ongoing. Preliminary quantitative results and recruitment will be presented. 240 individuals were invited, 65 participated (50% women; average age = 68.57). 10.53% reported clinical levels of FOP, with a mean score of 24.55 (range = 5–50). 21.82% reported moderate levels of death anxiety, with a mean score of 17.18 (range = 0–40). Preliminary results indicated a positive correlation between death anxiety and FOP.

### 179.4. Discussion or Conclusions

Despite the growing number of individuals living long-term with advanced lung cancer due to IOs and TTs, no studies have used mixed methods to assess prevalence and severity of psychological concerns in this population. This study aims to understand their experiences and improve the scientific conceptualization of FOP. This study will help inform psychosocial interventions for FOP and death anxiety in this emerging population.

**189**

## 180. Banding Together or Drifting Apart After Breast Cancer: A Comparative Thematic Analysis of Women’s Retrospective Accounts

Amanda Piccirilli ^1^, Karen Fergus ^1,2^, Sami Harb ^1^

1. York University, Toronto, Canada.
2. Sunnybrook Odette Cancer Centre, Toronto, Canada.

### 180.1. Background/Rationale or Objectives/Purpose

While the cancer mortality rate has decreased over time, the stressors of surviving cancer carry forward affecting not only the diagnosed person, but also their life partner. One relationship quality that has been proposed to promote couple resilience and partners’ ability to cope collectively and effectively with significant life adversity is the couple’s strength of relationship identity, or “we-ness”. This study investigated the processes by which relationships may become strengthened, or sense of we-ness enhanced, following breast cancer (BC), as well as the processes by which a relationship breaks down in the face of BC.

### 180.2. Methodology or Methods

Sample and setting: Six, heterosexual women with a history of BC were recruited from a Toronto-based cancer centre and the surrounding community. Procedures: In keeping with a Task Analytic methodology (Greenberg, 2007), three women with a history of BC who reported their relationship had strengthened, and three of whom reported relationship weakening or dissolution following cancer –were recruited and interviewed. Interview transcripts were analyzed using reflexive thematic analysis (Braun & Clarke, 2019).

### 180.3. Impact on Practice or Results

Themes emerging from the ‘strengthened’ group were: improved communication, being aligned in life goals, and partners’ creating space to ‘focus’ on their recovery. Themes emerging from the ‘weakened’ group were growing distance between partners, communication breakdown, and feeling uncared for/abandoned.

### 180.4. Discussion or Conclusions

Given the widespread interest for couple-based interventions in cancer care, these findings help to refine theoretical understanding of processes that foster or erode we-ness in order to improve psychosocial interventions for women and their partners coping with BC.

**190**

## 181. Evaluating the Relationship Between Marital Adjustment and Fear of Cancer Recurrence Among Young Women with Breast Cancer Across Three Years Post-Treatment: A National Multicentre Cohort Study

Sami Harb ^1^, Arjunvir Singh ^1^, Susan Isherwood ^2^, May Lynn Quan ^2^, Shira Yufe ^1^, Karen Fergus ^1,3^, RUBY Executive Committee, RUBY Site Investigators

1. York University, Toronto, Canada.
2. University of Calgary, Calgary, Canada.
3. Odette Cancer Centre, Sunnybrook Hospital, Toronto, Canada.

### 181.1. Background/Rationale or Objectives/Purpose

To determine how fear of cancer recurrence (FCR) may change across three years post-treatment in young (<41 years-old at time of diagnosis) breast cancer patients, accounting for possible marginal effects of marital adjustment (MA), motherhood identity, generalized anxiety, depression, and illness intrusiveness.

### 181.2. Methodology or Methods

Data were acquired from a Canadian multicentre prospective cohort study before the start of primary treatment and at three yearly time-points post-treatment. A linear mixed-effects model was employed to analyze change in FCR across time. Each covariate was evaluated as a hypothesized moderator of the FCR-time relationship, and the influence of covariates on FCR trajectories was evaluated.

### 181.3. Impact on Practice or Results

306 participants were included in analysis, with mean age of 42.27 years (*SD* = 3.92), and 175 participants (57%) identified as mothers. The analysis prioritized a more parsimonious main-effects-only-model compared to the hypothesized model as it demonstrated comparable explanatory power. There was a significant positive FCR-time trend, and higher FCR scores were significantly associated with higher anxiety, depression, and illness intrusiveness across time. Despite a significant negative FCR-MA bivariate correlation, this association was suppressed when analyzed within a multivariable linear model that included covariates.

### 181.4. Discussion or Conclusions

Our findings suggest that the FCR-MA relationship might conceptually reflect a meaningful backdrop to cognitive-emotional appraisals of FCR. Although other covariates accounted for the explained variance in FCR that was otherwise attributed to MA in a bivariate context, the nature of this suppressed association remains unclear. This study underscores a possible connection of FCR to relationship perception, thereby highlighting the need for accessible psychosocial care for couples/families.

**202**

## 182. Enhancing Group Support in Young Adult Cancer Care: Adapting Fear of Cancer Recurrence Therapy for Young Adults with Hematological Malignancies

Sharlane Lau ^1^, Norma D’Agostino ^1^, Pamela Mosher ^2^, Marlie Smith ^3^, Jennifer Jones ^2,4^, Vishal Kukreti ^5,6^, Rinat Nissim ^1,2^, Christine Maheu ^7^, Sophie Lebel ^8^, Aliza Panjwani ^1,2^

1. Department of Supportive Care, Princess Margaret Cancer Centre, University Health Network, Toronto, Canada.
2. Department of Psychiatry, University of Toronto, Toronto, Canada.
3. Chelsea and Westminster Hospital NHS Foundation Trust, London, UK.
4. Princess Margaret Cancer Centre, University Health Network, Toronto, Canada.
5. Division of Medical Oncology and Hematology, Princess Margaret Cancer Centre, University Health Network, Toronto, Canada.
6. Department of Medicine, Toronto, Canada.
7. Ingram School of Nursing, McGill University, Toronto, Canada.
8. Faculty of Social Sciences, University of Ottawa, Ottawa, Canada.

### 182.1. Background/Rationale or Objectives/Purpose

Fear of cancer recurrence (FCR) is a critical concern among young adults (YAs). Although tailored interventions for YAs with cancer are scarce, nearly 1 in 2 report a desire for group and online supports. Fear of Cancer Recurrence Therapy (FORT) is a cognitive-existential group intervention shown to reduce FCR in breast and gynecological patients with cancer. Centering voices of patients with lived experience, the purpose of the current study was to adapt FORT for YAs with hematological malignancies (HM) and virtual delivery, with lymphoma as the pilot site.

### 182.2. Methodology or Methods

YAs with experiences of lymphoma and FCR (n = 6, 50% females; age_range_ = 20–37) formed a patient advisory board (PAB). The PAB participated in four virtual meetings to provide feedback on FORT content, language, functionality, and delivery. Meeting transcripts were analyzed through thematic analysis. FORT content and developmental context experts provided feedback integrating modifications suggested by the PAB.

### 182.3. Impact on Practice or Results

YAs found FORT to be relevant and valued its group format for fostering shared understanding and peer connection. They endorsed online delivery due to improved accessibility. YAs provided targeted feedback, including incorporating values aligning with aspects of identity, content on hope and meaning, triggers specific to YAs & HM, softened language in cognitive restructuring, and greater interaction in a virtual format.

### 182.4. Discussion or Conclusions

This study addresses the significant unmet need of FCR among YAs with HM while leveraging the benefits of group support and online format to improve accessibility. FORT-YA shows significant potential for applicability to other YA cancer populations, with next steps including a feasibility trial.

## 183. Final Category: P. Other

**4**

## 184. Fear of Cancer Recurrence in Breast Cancer Survivors Carrying a *BRCA1* or *2* Genetic Mutation: A Cross-Sectional Study

Emma Coughlan ^1,2,3^, Alexandra Michel ^1,2,3^, Michel Dorval ^1,2,4^, Jocelyne Chiquette ^1,2,3^, Josée Savard ^1,2,3^

1. Université Laval, Québec, Canada.
2. CHU de Québec-Université Laval Research Center, Québec, Canada.
3. Université Laval Cancer Research, Québec, Canada.
4. CISSS de Chaudière-Appalaches Research Center, Lévis, Canada.

### 184.1. Background/Rationale or Objectives/Purpose

This cross-sectional study, conducted in women who were treated for breast cancer and carrying a *BRCA1/2* mutation, aimed to: (1) assess the mean level of fear of cancer recurrence (FCR) and estimate the proportion of patients with clinical levels of FCR; (2) examine the relationships between FCR and some psychological variables (e.g., avoidance, intolerance to uncertainty) and quality of life; (3) explore whether FCR levels vary as a function of the past preventive treatment received; and (4) assess the associations between FCR and the presence of decisional conflict or regret regarding preventive surgeries.

### 184.2. Methodology or Methods

89 female participants were recruited in the province of Québec through email using the distribution list of the oncogenetics network. Participants were invited to complete a battery of questionnaires online.

### 184.3. Impact on Practice or Results

70.8% of the participants showed a clinical level of FCR. FCR was significantly associated with higher levels of anxiety and depression, and greater avoidance and intolerance of uncertainty, but not with quality of life. No significant difference was observed on the total FCR score between women who had received preventive surgery (mastectomy and/or salpingo-oophorectomy) and those considering it, and those not considering it. Association was significant between higher FCR scores and greater decisional conflicts and regrets about choosing to undergo preventive surgery.

### 184.4. Discussion or Conclusions

These data suggest that FCR is a significant problem for breast cancer survivors carrying a *BRCA1/2* genetic mutation, even after undergoing a prophylactic surgery. This highlights the importance of providing these women with specific psychological intervention focusing on FCR.

**55**

## 185. Integrating Psychosocial Oncology Advocacy Priorities as Reported by Cancer-Related Organizations: Insights from a Cross-Canada Online Survey

Samar Attieh ^1^, Andreea Constantinescu ^2^, Sevtap Savas ^3^, kimberley Thibodeau ^4^, Carmen G. Loiselle ^2^

1. Department of Experimental Medicine, Faculty of Medicine and Health Sciences, McGill University, Montreal, Canada.
2. Department of Oncology and Ingram School of Nursing, Faculty of Medicine and Health Sciences, McGill University, Montreal, Canada.
3. Division of Biomedical Sciences, Faculty of Medicine, Memorial University, St John’s, Canada.
4. McGill University Health Centre, Montreal, Canada.

### 185.1. Background/Rationale or Objectives/Purpose

The Canadian Association of Psychosocial Oncology (CAPO) sought input from key Canadian organizations advocating for psychosocial oncology (PSO) to create a shared national PSO advocacy strategic plan.

### 185.2. Methodology or Methods

Following the identification of 73 potentially relevant cancer-related organizations, a brief online survey confirmed that 54 were engaged in PSO advocacy. Next, a 27-item follow-up survey served to identify each organization’s PSO priorities and challenges.

### 185.3. Impact on Practice or Results

Of the 54 organizations contacted, 30 responded to the follow-up survey. Sixty-one percent were based in Ontario (n = 11, 35%) or Quebec (n = 8, 26%). Although 18 organizations (40%) reported having a dedicated advocacy committee, only 4 (13%) allocated a separate budget for advocacy activities. Twenty-three organizations (76%) identified lack of resources as their primary challenge, with 22 (73%) relying on donations to fund their PSO advocacy activities. Convergent PSO advocacy priorities across participating organizations included improving access to timely, equitable, age appropriate and tailored PSO support for affected individuals. Additional PSO priorities included raising awareness, fostering partnerships, and advocating for increased funding and research. Divergent priorities centered around advocating for particular cancer groups, complementary therapies, and workplace reintegration and support.

### 185.4. Discussion or Conclusions

Findings offer additional insights into current PSO advocacy efforts by Canadian cancer organizations. These will serve to inform the co-development of a national PSO advocacy strategic plan to strengthen the collective impact of PSO advocacy efforts from coast to coast.

**81**

## 186. The Association of In-Hospital Recreational Oncology Programming and Psychosocial Health in Parents/Caregivers of Pediatric Cancer Patients

Divine Udobor ^1^, Sarah O’Connell ^1^, Shannon Miller ^1^, Lauren Chisholm ^2^, Sarah L West ^1^

1. Trent University, Peterborough, Canada.
2. Campfire Circle, Muskoka, Canada.

### 186.1. Background/Rationale or Objectives/Purpose

Parents and caregivers of pediatric cancer patients experience significant life-altering changes including becoming primary caregivers, decision makers and family maintainers. This places them at risk for high stress and decreased well-being, therefore, opportunities that may reduce the burden of responsibility on parents/caregivers are needed. This study investigated the association of patient participation in in-hospital recreational oncology camp (ROC) with parent/caregiver state stress, depression and anxiety.

### 186.2. Methodology or Methods

Parents/caregivers were recruited from ROC registration list (Campfire Circle) and were placed into groups: (1) child currently in-hospital/on treatment and accessing ROC; (2) child currently in-hospital/on treatment and previously, but not currently, accessing ROC; (3) child not currently in-hospital/on treatment and previously accessed ROC; and (4) child not currently in-hospital/on treatment and never accessed ROC programs. Parents/caregivers completed a survey including the Depression Anxiety Stress Scale and the Perceived Stress Questionnaire and answered open-ended questions about their experiences with ROC. T-tests and Mann-Whitney U tests compared quantitative outcomes, and thematic analysis examined qualitative responses.

### 186.3. Impact on Practice or Results

Quantitative analysis included 159 parent/caregivers (42.5 ± 11.1 years old), with 131 participants (42.6 ± 6.8 years old) included in the thematic analysis. There were no differences in state stress, depression and anxiety between parents/caregivers whose child had access to the in-hospital ROC and those who did not. However, thematic analysis revealed four main themes with ROC participation: positive emotions, appreciation for the ROC, reassurance of their child’s well-being, and the ability to relax. Parents/caregivers also reported that ROC provided a socially engaging environment and distraction for their children.

### 186.4. Discussion or Conclusions

While in-hospital ROC participation was not associated with a difference in parent/caregiver state stress, depression and anxiety, parents/caregivers described great appreciation for ROC and that their children had a socially inclusive environment allowing them to be distracted from their cancer.

**152**

## 187. Developing Collaborative Practices with Clinical and Psychosocial Teams to Support Patients Experiencing Suicidal Ideation in Cancer Care Alberta

April Wales, Claire Link, Andrea DeIure

Cancer Care Alberta, Calgary, Canada.

### 187.1. Background/Rationale or Objectives/Purpose

A cancer diagnosis can impact a person’s mental health and may be accompanied by increased risk of dying by suicide. Cancer Care Alberta aims to provide patients with specialized support when experiencing suicidal ideation. Providing this support can be complex, and psychosocial teams have expressed a need for clear roles and responsibilities in addressing this particular patient need in the clinical environment.

### 187.2. Methodology or Methods

Surveys were administered to clinical and psychosocial teams to assess competencies in this area and identify knowledge and resource gaps. Responses from 166 clinical staff members and 24 psychosocial care providers highlighted that many staff feel unprepared to effectively assess and address suicide risk. Additionally, there was a general lack of clarity regarding when patients should be formally referred to the psychosocial department, based on their risk level.

### 187.3. Impact on Practice or Results

Drawing on the survey results, CCA is developing a strategy to support clinical and psychosocial staff to provide patients with the right level of support based on their unique circumstances and suicide risk level. The goal is to create collaborative capacity and clear expectations for staff involved in the screening, assessment, and ongoing support of patients experiencing suicidal ideation in Alberta.

### 187.4. Discussion or Conclusions

CCA has identified the need for more collaborative staff practices to appropriately assess and address suicidal ideation experienced by patients. This presentation will use case examples to highlight how multidisciplinary cancer care teams can function cohesively to effectively assess patients’ risk and provide personalized support.

**159**

## 188. The Human Crisis in Cancer: A Lancet Oncology Commission

Hanae Davis ^1^, Nirmala Bhoo-Pathy ^2^, Karla Unger ^3^, Mac Skelton ^4^, Dario Trapani ^5^, Elizabeth Smyth ^6^, Naveen Salins ^7^, Madeline Li ^1^, Richard Sullivan ^8^, Gary Rodin ^1^

1. Princess Margaret Cancer Centre, Toronto, Canada.
2. University of Malaya, Kuala Lumpur, Malaysia.
3. National Institute of Cancerology, Mexico City, Mexico.
4. The American University of Iraq Sulaimani, Sulaymaniyah Governorate, Iraq.
5. University of Milan, Milan, Italy.
6. Oxford University Hospitals, Oxford, UK.
7. Kasturba Medical College, Manipal, India.
8. King’s College London, London, United Kingdom.

### 188.1. Background/Rationale or Objectives/Purpose

Over 19 million cancer cases were diagnosed globally in 2020 and that is projected to increase by 75% over the next three decades. This growth will be most significant in low and middle-income countries, where there is the least preparation and resilience in health systems capacity. While the human cost is enormous, there is an imbalance in the attention and resources allocated to the physical and psychological well-being, quality of life, and social circumstances of patients and families compared to the biomedical dimensions of the disease. This has resulted in a global crisis of avoidable human suffering that is largely ignored and under-resourced.

### 188.2. Methodology or Methods

The Lancet Oncology invited Drs. Gary Rodin and Richard Sullivan to lead a commission on the human crisis in cancer. Evidence for the crisis, factors that are driving it, and global solutions will be elucidated through novel data, meta-analytic reviews, and case studies in the following areas: fragmented care systems, insufficient availability and access to mental health and palliative care, increased financial toxicity, limited training on the human aspects in medical education, and insufficient consideration to the sociocultural factors that influence care, research, and policy.

### 188.3. Impact on Practice or Results

Scheduled to launch in 2025, the Commission will put a spotlight on this crisis at a critical tipping point, and aims to change the global narrative around person-centred cancer care.

### 188.4. Discussion or Conclusions

Preliminary solutions to be discussed include: expand humanistic competencies in oncology curricula, incentivize embedding of psychological and palliative care in health systems, and explore innovative health financing models to fund patient-centred interventions.

**172**

## 189. Scoping Review Protocol: Inventory of Tested Quality of Life Measurements for Caregivers of Solid-Tumour Patients

Ala Refai ^1,2^, Aliaa Sabry ^2,3^, Margaret Fitch ^4,5^, Jill I Cameron ^4,6^, Andrew McPartlin ^7^, Rosemary Martino ^1,2,8^

1. Rehabilitation Sciences Institute, Department of Speech-Language Pathology, and the Swallowing Lab, University of Toronto, Toronto, Canada.
2. Krembil Research Institute, University Health Network, Toronto, Canada.
3. The Swallowing Lab, University of Toronto, Toronto, Canada.
4. Rehabilitation Sciences Institute, University of Toronto, Toronto, Canada.
5. Bloomberg Faculty of Nursing, University of Toronto, Toronto, Canada.
6. Department of Occupational Science and Occupational Therapy, Temerty Faculty of Medicine, University of Toronto, Toronto, Canada.
7. Department of Radiation Oncology, University Health Network, Toronto, Canada.
8. Department of Otalaryngology-Head and Neck Surgery, University of Toronto, Toronto, Canada.

### 189.1. Background/Rationale or Objectives/Purpose

Assessing the quality of life (QoL) of caregivers is critical to address the negative consequences of caregiving such as risks of poor psychological health. There is no inventory of caregiver QoL measurements that researchers and clinicians can use to inform selection of tools that have (a) psychometric validation, (b) well-defined QoL, and (c) a focus on solid tumour cancers. This scoping review protocol outlines the methods to derive such an inventory.

### 189.2. Methodology or Methods

The databases used will be MEDLINE, EMBASE, CINAHL, EBM Reviews, PsycINFO, Health Technology Assessment, and Health and Psychosocial Instruments.

This scoping review will adhere to the Joanna Briggs Institute guidelines. Screening of abstracts and then full articles of accepted abstracts will abide by apriori criteria, which includes validated QoL tools used in adult informal caregivers of solid tumour cancer patients. A second reviewer will screen abstracts, checking interrater reliability on a random selection of 20% of abstracts (threshold of Cohen’s *k* = ≥0.80). If reliability is suboptimal, reviewer training will repeat. Discrepancies will be resolved by a third rater. The first reviewer will extract data related to: cancer type, QoL domains targeted, and psychometric properties. A second reviewer will check the extracted data for 20% of articles.

### 189.3. Impact on Practice or Results

Databases generated 11,005 abstracts, with 2300 accepted for full-text screening.

### 189.4. Discussion or Conclusions

This will be the first inventory of validated/reliable QoL tools for caregivers catalogued by solid tumour type and QoL domains. It will serve as an essential resource to inform researchers and healthcare providers on the suitability of tools in each population.

**175**

## 190. Qualitative Study Protocol: Exploring Quality of Life in Caregivers of Head and Neck Cancer Patients with Dysphagia

Ala Refai ^1,2^, Margaret Fitch ^3,4^, Jill I Cameron ^3,5^, Andrew McPartlin ^6^, Rosemary Martino ^1,2,7^

1. Rehabilitation Sciences Institute, Department of Speech-Language Pathology, and the Swallowing Lab, University of Toronto, Toronto, Canada.
2. Krembil Research Institute, University Health Network, Toronto, Canada.
3. Rehabilitation Sciences Institute, University of Toronto, Toronto, Canada.
4. Bloomberg Faculty of Nursing, University of Toronto, Toronto, Canada.
5. Department of Occupational Science and Occupational Therapy, Temerty Faculty of Medicine, University of Toronto, Toronto, Canada.
6. Department of Radiation Oncology, University Health Network, Toronto, Canada.
7. Department of Otolaryngology-Head and Neck Surgery, University of Toronto, Toronto, Canada.

### 190.1. Background/Rationale or Objectives/Purpose

Few studies examine caregiver quality of life (QoL) in head and neck cancer (HNC) in relation to dysphagia. Notably, these studies either use QoL and other terms interchangeably or do not comprehensively cover the domains of QoL. Further, none have investigated this caregiver QoL with the consideration of cancer treatments and dysphagia treatment. There is a need to explore the QoL of informal caregivers of HNC patients receiving various cancer treatments, within the context of dysphagia and dysphagia intervention.

### 190.2. Methodology or Methods

This is a protocol for a study that will include adult caregivers of HNC patients treated for dysphagia who previously received radiotherapy or chemoradiation or surgery. This project follows a constructivist grounded theory approach. Approximately 40–60 caregivers will individually participate in a one-hour semi-structured interview either in-person or virtually. Interviews will be transcribed and analyzed using a constant comparative analysis.

Each consenting participant will first complete a demographic survey, identifying characteristics known to be associated with QoL (e.g., income, education, etc.). Next, they interview will explore key topics regarding their perceptions on QoL in reference to the cancer and dysphagia impacted over time (i.e., from patient diagnosis up to the day of the interview). The guide was developed and structured in accordance with the six QoL domains outlined by the World Health Organization.

### 190.3. Impact on Practice or Results

N/A

### 190.4. Discussion or Conclusions

These studies will be the first of their kind to gather a deeper understanding of the dysphagia-related impact on HNC caregiver QoL with the consideration of cancer treatment modality and dysphagia treatment.

**176**

## 191. Exploring Family Caregiver Distress and Burden in Psychosocial Oncology

Andréa Maria Laizner ^1,2,3^, Camilo Sierra-Herrera ^1,2^, Anita Mehta ^3,4^

1. McGill University Health Centre, Montreal, Canada.
2. Research Institute of the McGill University Health Centre, Montreal, Canada.
3. McGill University, Montreal, Canada.
4. Montreal Institute for Palliative Care/Branch of the Teresa-Dellar Palliative Care Residence, Kirkland, Canada.

### 191.1. Background/Rationale or Objectives/Purpose

Family members have multiple roles and responsibilities when caring for someone diagnosed with cancer. The distress associated with these roles can result in family members experiencing anxiety and depression, as well as caregiver burden. Previous research developed an English version of the Family Member Problem Checklist (FMPC), identifying multiple domains contributing to family members’ distress. **Purpose:** To explore family caregiver distress (contributing domains; anxiety and depression) and burden among caregivers of patients treated at a university hospital.

### 191.2. Methodology or Methods

Family caregivers were informed of the study via the psychosocial oncology clinic, pamphlets, posters and app notifications. With informed consent, caregivers rated their distress using Distress Thermometer and FMPC, Hospital Anxiety and Depression Scale, and Burden using self-report questionnaires in English or French. These were completed online or paper.

### 191.3. Impact on Practice or Results

19 of the 21 recruited caregivers completed all questionnaires. The mean age was 55 (SD 14), and 67% of the respondents were women. Patients’ cancer diagnosis varied (e.g., breast, lung, prostate, bladder, renal, sarcoma, leukemia, pancreas and multiple myeloma). 62% were living with advanced disease. Caregivers reported median distress of 6/10 (IQR 5–7.5). HADs: Anxiety Mean 10.8 (SD 5), Depression 8.3 (SD 5).

### 191.4. Discussion or Conclusions

Elevated caregiver distress is concerning as it can be higher than that of patients with cancer. This presentation will provide more details about caregiver distress, contributing domains, and burden. Nurses and social workers are well-placed to screen for caregiver distress, provide initial support, and refer those with moderate to severe distress and burden for supportive interventions to psychosocial oncology or other services.

**183**

## 192. What Else Do You Want to Know?—Identifying Educational Needs of Professionals Providing Psychosocial Oncology Care

Gregory Gooding ^1^, Jochen Ernst ^2^, Andrea Laizner ^1^, Celestina Martopullo ^3^, Lisa Roelfsema ^4,5^, Melba D’Souza ^6^, Annett Körner ^1^

1. McGill University, Montreal, Canada.
2. Medical Center of the University of Leipzig, Leipzig, Germany.
3. University of Calgary, Calgary, Canada.
4. Ontario Health—Cancer Care Ontario, Ontario, Canada.
5. Trillium Health Partners—Credit Valley Hospital, Ontario, Canada.
6. Thompson Rivers University, Kamloops, Canada.

### 192.1. Background/Rationale or Objectives/Purpose

The Education Committee of the Canadian Association of Psychosocial Oncology (CAPO) is committed to promoting best practices in psychological, social, physical and spiritual well-being among individuals affected by cancer through excellence in educational initiatives that enhance knowledge and skills in psychosocial oncology practitioners.

### 192.2. Methodology or Methods

To identify the most pertinent training needs of health care professionals in psycho-oncology, CAPO’s 2023 survey among CAPO members included questions about the most important educational training needs as well as the preferred delivery modalities.

### 192.3. Impact on Practice or Results

The most highly endorsed topic domain was: “Specific psychological interventions”—rated as very or extremely important by 79% of the 58 survey respondents. However, to not overburden participants, a multitude of interventions was grouped into this survey question, e.g., CBT, ACT, Motivational Interviewing, Dignity therapy, and more. Thus, the current data do not allow determining which evidence-based interventions practitioners are most interested in learning about.

Live webinars were the first choice of delivery modality, but pre-recorded, self-paced online education, a combination of virtual and in-person learning, as well as exclusively in-person training were frequently endorsed.

### 192.4. Discussion or Conclusions

Beyond reporting the current findings regarding training needs and delivery modality, our presentation aims at collecting more specific data via QR code leading to a brief follow-up survey. Conference participants can conveniently use their smart phone to indicate their most pertinent training needs in real-time. Those data will inform the work of CAPO’s education committee and help tailor future educational opportunities to the most pressing training needs of professionals providing psychosocial oncology care.

**187**

## 193. Are Humanistic Dimensions of Cancer Being Considered in National Cancer Control Plans? A Global Review of 156 Countries

Emily Lau ^1,2^, Gilla Shapiro ^1^, Nirmala Bhoo-Pathy ^3^, Karla Unger ^4^, Mac Skelton ^5^, Dario Trapani ^6^, Elizabeth Smyth ^7^, Naveen Salins ^8^, Richard Sullivan ^9^, Gary Rodin ^1,2^, Madeline Li ^1^

1. Princess Margaret Cancer Centre, Toronto, Canada.
2. Dalla Lana School of Public Health, University of Toronto, Toronto, Canada.
3. University Malay, Malaysia.
4. National Institute of Cancerology, Mexico City, Mexico.
5. Institute of Regional & Intl. Studies, The American University of Iraq Sulaimani, Iraq.
6. Department of Oncology and Hematology, University of Milan, Milan, Italy.
7. Oxford University Hospitals, UK
8. Kasturba Medical College of Manipal, Manipal Academy of Higher Education, India.
9. King’s College London, London, United Kingdom.

### 193.1. Background/Rationale or Objectives/Purpose

National Cancer Control Plans (NCCPs) are policy documents describing a country’s prioritized actions for cancer control. To support the Lancet Oncology Commission on the Human Crisis of Cancer which addresses the relative imbalance between biomedical cancer care compared to psychosocial care, we examined how common humanistic dimensions of cancer care were included in global cancer plans.

### 193.2. Methodology or Methods

The dataset from the 2023 global review of National Cancer Control Plans conducted by the International Cancer Control Partnership (ICCP) includes a standardized 99-item assessment on 98 NCCPs and 58 Non-Communicable Disease (NCD) plans across 156 countries as of November 2023. Questions relevant to humanistic aspects of cancer care were extracted. A descriptive analysis was conducted, stratified by WHO region and World Bank Index levels: Upper (UIC), upper middle (UMIC), lower middle (LMIC) and lower (LIC) income levels.

### 193.3. Impact on Practice or Results

Overall, 21.2% (22/99) of the questions in the dataset were relevant to the humanistic dimensions of cancer care. The proportion of questions relating to the themes of the *Commission*, included systems of care (45.5%), sociocultural factors (13.6%), mental health (13.6%), palliative care (13.6%), economic factors (9.1%), and medical education (4.5%). Among countries answering any of the 22 questions, humanistic dimensions of cancer care were most common in the European Region (26.9%) and least common in SEARO (7.1%). By income level, they were most common in UIC (32.3%), followed by UMIC (27.1%), LMIC (26.5%), and LIC (14.2%).

### 193.4. Discussion or Conclusions

Significant disparities remain in cancer plans regarding attention to the humanistic dimensions of cancer care, particularly in SEARO and LICs. Addressing such disparities is vital for equitable access to high-quality cancer care.

**196**

## 194. “We’re the Silent People in the Situation”: A Qualitative Examination of the Lived Experiences of Partners of Individuals Diagnosed with Testicular Cancer

Emily Memedovich ^1^, Perri Tutelman ^1,2^, Caitlin Forbes ^1^, Brian Kelly ^1,3^, Zeev Rosberger ^4,5,6^, Cindy Railton ^2,7^, Oneball Charitable Cancer Organization ^8^, Igor Stukalin ^1,8^, Barry D. Bultz ^1,2,3^, Fiona Schulte ^1,9^

1. Cumming School of Medicine, University of Calgary, Calgary, Canada.
2. Tom Baker Cancer Centre, Calgary, Canada.
3. School of Medicine and Public Health, University of Newcastle, Newcastle, Australia.
4. Lady Davis Institute for Medical Research, Jewish General Hospital, Montreal, Canada.
5. Departments of Psychology, Oncology, & Psychiatry, McGill University, Montreal, Canada.
6. Institute of Community and Family Psychiatry, Montreal, Canada.
7. Faculty of Nursing, University of Calgary, Calgary, Canada.
8. Oneball Charitable Cancer Organization, Calgary, Canada.
9. Alberta Children’s Hospital, Calgary, Canada.

### 194.1. Background/Rationale or Objectives/Purpose

Testicular cancer (TC) is one of the most prevalent cancers among adolescent and young adult males. TC can have significant physical and psychosocial impacts on patients and their loved ones, however limited research has focused on the experiences of their partners. Thus, the aim of this study was to explore the lived experiences of partners of individuals diagnosed with TC.

### 194.2. Methodology or Methods

This study used an interpretive phenomenological research design. Three focus groups with 4-5 participants per group were conducted with partners of individuals diagnosed with TC (n = 13; 100% female; mean age = 30.77 years [SD = 6.10]). The majority of partners were married (n = 8), and for eleven (n = 11) the relationship had commenced prior to the TC diagnosis. Data were then analysed using reflexive thematic analysis.

### 194.3. Impact on Practice or Results

Three overarching themes were generated that describe the partners’ experiences: (1) women suffer silently with their partner’s diagnosis, minimizing their own experiences and emotions due to guilt and fear of being a burden; (2) as caregivers, partners must balance various new roles and responsibilities which becomes a large part of their identity; and (3) partners must confront a future that is different than expected as a result of the cancer experience.

### 194.4. Discussion or Conclusions

Women experienced unique emotional and practical challenges when supporting their partner throughout their TC experience. The results of this study highlight the need for improved support and resources tailored to partners of individuals diagnosed with TC.

**198**

## 195. Assessing the Barriers to Psychosocial Oncology Care: Preliminary Results from a Global Survey Study

Juan P. Borda ^1^, Gilla Shapiro ^1^, Chioma Asuzu ^2^, Cristiane D. Bergerot ^3^, Jeffrey C. Dunn ^4^, Loreto del Pilar Fernandez ^5^, Anja Mehnert-Theuerkauf ^6^, William E. Rosa ^7^, Wendy T. Lam ^8^, Madeline Li ^1^

1. Princess Margaret Cancer Centre, Supportive Care Department, Toronto, Canada.
2. University of Ibadan, Counselling & Human Development Studies Department, Ibadan, Nigeria.
3. Grupo Oncoclinicas, Supportive Care Department, Brasilia, DF, Brazil.
4. University of Southern Queensland, Division of Research and Innovation, Toowoomba, Australia.
5. Instituto Oncológico, Fundación Arturo López Perez, Cancer Research Department, Santiago, Chile.
6. University Leipzig, Medical Faculty, Medical Psychology and Medical Sociology Department, Leipzig, Germany.
7. Memorial Sloan Kettering Cancer Center, Counselling & Human Development Studies Department, New York, USA.
8. The University of Hong Kong, School of Public Health, Hong Kong, China.

### 195.1. Background/Rationale or Objectives/Purpose

This study aims to identify barriers to effective psychosocial oncology (PSO) care and to propose solutions for enhancing its integration into anti-cancer therapy.

### 195.2. Methodology or Methods

Two web-based surveys, tailored to patients and healthcare providers, were distributed using a research marketing company to networks of professional organizations, patient advocacy groups, and cancer centers. The patient survey focused on experiences and challenges accessing psychosocial care, while the provider survey collected data on the availability of PSO training, systemic barriers, and the prioritization of PSO. Quantitative data were analyzed using descriptive statistics, while qualitative responses underwent thematic analysis to identify common themes.

### 195.3. Impact on Practice or Results

To date, 108 patients with cancer (median age = 55) and 134 healthcare providers (median age = 44) representing 30 countries in high and low resource settings worldwide completed the survey. Patients identified barriers such as limited access to PSO-trained professionals (66%), financial constraints (48%), and stigma (33%). Providers reported that access challenges included lack of funding (44%), insufficient PSO staff (43%), insufficient training (37%), and low institutional prioritization of PSO care (34%). Key strategies for improvement included expanding PSO training, integrating PSO specialists into oncology teams, and increasing community awareness about PSO care. Over 90% of respondents viewed PSO care as equally or more important than biomedical care.

### 195.4. Discussion or Conclusions

The findings from this survey underscore the need for systemic changes to address barriers in PSO care. Proposed strategies to enhance equitable access to PSO care will inform *The Lancet Oncology* Commission on the Human Crisis of Cancer.


**Author Name**

**Abstract Number**

**Abou Chaar, Emile**
83
**Abu Saris, Nur El Huda**
167
**Adham, Heide**
86, 166
**Affoo, Rebecca**
93
**Afshar, Tina**
40
**Aguiar, Stefan**
160, 182, 184
**Ajmera, Faye**
149
**al Kindy, Aida**
160
**Al-Chami, Mohamad Hamze**
53
**Alberts, Nicole M.**
102
**Alibhai, Shabbir**
157
**Allin, Sara**
123
**Amaral, Eugenia**
184
**Amenu, Bikila**
39
**Angka, Leonard**
54
**Arab, Marianne**
178
**Arbour-Nicitopoulos, Kelly**
50
**Asuzu, Chioma**
198
**Attieh, Samar**
54, 55, 201
**Aubin, Francine**
115
**Audoin, Michelle**
36
**Auer, Rebecca C.**
54
**Avery, Jonathan**
18, 77, 134
**AYA Cancer PSP Team, on behalf of the**
47
**Baek, Junhee**
149, 150
**Baerg, Eden**
67
**Bailly, Greg**
194
**Baird, Malcolm**
192
**Baker, Amber**
109
**Baker, Maria**
65
**Banville, Catherine**
95
**Barbeau, Anne**
182, 184, 195
**Barnard, Kathy**
36
**Barnes, Dallas**
114
**Bates, Alan**
11, 18, 137
**Bays, Suzanne**
69
**Bean, Sally**
142, 146, 149, 150
**Bearman, Shandy**
109
**Beattie, Sara**
107, 126
**Bell, David**
194
**Bell, John**
54
**Bender, Jackie**
28, 73, 78, 151, 200
**Benoit, Maggie**
53
**Bergeron, Élodie**
37
**Bergerot, Cristiane D.**
198
**Bhoo-Pathy, Nirmala**
159, 187
**Bhopa, Shania**
114
**Bilau-Howie, Natasha**
177
**Binder, Louise**
36
**Bishop, Derrick**
13
**Bocangel, Patricia**
148
**Bolotin, Shelly**
34
**Boone, Jennifer**
178
**Borda, Juan P.**
198
**Bowes, David**
194
**Brinson, Jeff**
124
**Bruce, Aisha**
167
**Brunet, Jennifer**
6, 7, 29, 33, 203
**Brunet, Mylène**
83
**Bryant-Lukosius, Denise**
63
**Bucher, Oliver**
85
**Buick, Catriona**
76
**Bultz, Barry D.**
196
**Burgos, Anna**
192
**Butler, Chelsea**
43, 46, 58
**Button, Amanda**
82
**Bédard, Melanie**
68
**Bélanger, Lynda**
14
**Cabaluna, Jeus**
131
**Cadden, Lisa**
48
**Cahill, Kristina**
48
**Calamia, Marianna**
157
**Cameron, Danielle**
51
**Cameron, Gillian**
114
**Cameron, Jill I**
172, 175
**Campbell, Allie**
178
**Campbell, Kristin**
67, 105
**Camus, Jean-Claude**
39
**Cannon, Jocelyn L.**
12, 105
**Caplette-Gingras, Aude**
14, 95
**Capozzi, Lauren**
32, 105
**Carignan, Julie**
54
**Carlson, Linda**
107, 126
**Carmona, Nicole**
89
**Carruyo Soto, Yustine**
130
**Caru, Maxime**
102
**Catsburg, Jennifer**
134
**Catton, Jennifer**
173
**Champagne, Elaine**
83
**Changoor, Alana**
73
**Charitable Cancer Organization, Oneball**
196
**Cheng, Samantha**
181
**Cherblanc, Jacques**
83
**Chhina, Simran**
43
**Chiquette, Jocelyne**
4
**Chisholm, Lauren**
8, 81
**Christensen, Thomas**
105
**Chu, Alanna**
107, 126, 168, 180
**Chu, Paige**
142, 146, 157
**Coffey, Claire**
48
**Coish, Jennifer**
15
**Collaud, Thierry**
83
**Constantinescu, Andreea**
55
**Conte, Ellen**
29
**Coombs, Melissa**
72
**Corbett, Cyndi**
15
**Cote, Rebecca**
98, 134
**Coughlan, Emma**
4
**Cripps, Hannah**
32, 74
**Croke, Jennifer**
62
**Crotty, Pam**
127
**Cuda, Natalie**
50
**Culos-Reed, S. Nicole**
12, 32, 67, 72, 74, 105, 179
**Curnier, Daniel**
5
**Currey, In memory of Lisa**
72
**Curtis, Aaron**
15
**Cuthbert, Colleen**
105
**D’Agostino, Norma**
202
**D’Alton, Paul**
48
**D’Souza, Melba**
1, 183
**D’Souza, Violet**
93, 94
**Dalinda, Alyssa**
112
**Daniels, Sarah**
147
**Danyluk, Jessica**
179
**Das, Anirban**
42
**Daun, Julia T.**
12, 32, 179
**Davis, Hanae**
73, 118, 159, 162
**Dawes, Martin**
36
**De Gelder, Tammy**
63
**de Reland, Mireille**
16
**DeIure, Andrea**
128, 152
**del Pilar Fernandez, Loreto**
198
**Deljoomanesh, Shima**
78, 147, 151
**Demarco, Tatiana**
39
**Desbeaumes-Jodoin, Véronique**
115
**Deschênes, Sonya**
48
**Desjardins, Léandra**
37, 130
**Desserud, Don**
49
**Devarajah, Kaviya**
62, 64
**deVries, Froukje deVries**
160
**Dhani, Neesha**
173
**Dick, Peggy**
53
**Donato, Emily**
70
**Dornian, Kat**
109
**Dorval, Michel**
4
**Doré, Isabelle**
105
**Doyle, Zoe**
160
**Dreger, Julianna**
105
**Dunn, Jeffrey C.**
198
**Dunphy, Colleen**
173
**Duong, Jenny**
42, 86, 167
**Dushinski, John**
179
**Duval, Michel**
9
**Eaton, Geoff**
127
**Elias, Martine**
36
**Embrett, Mark**
178
**Empringham, Brianna**
67
**Ernst, Jochen**
183
**Esack, Samara**
98
**Etchegary, Holly**
13
**Executive Committee, RUBY**
190
**Eyre, Gwen**
34
**Fata, Ladan**
200
**Fawcett, Emily**
79
**Fay-McClymont, Taryn**
166
**Fazelzad, Rouhi**
147
**Feldstain, Andrea**
107, 126
**Fergus, Karen**
76, 89, 189, 190
**Ferguson, Sarah**
78, 147, 151
**Fern, Lorna**
135
**Filatoff, Jamie**
179
**Filiatrault, Megan**
67
**Fitch, Margaret**
172, 175
**Flett, Gordon**
95
**Flinn, Jill**
178
**Floyd, Laura**
109
**Fong, Angela**
129
**Fonteyne, Karlee**
35, 40
**Forbes, Abigail**
173
**Forbes, Caitlin**
42, 82, 86, 132, 166, 167, 196
**Forster, Victoria**
130
**Freedman, Howard**
39
**Gagnon, Marie-Pierre**
14
**Gagnon, Pierre**
83
**Galvin, Claire R.**
102
**Ganann, Rebecca**
63
**Gandhi, Karina**
62
**Gandhi, Sheila**
134
**Gandy, Ellen**
53, 56
**Garland, Sheila**
65, 66, 79, 90, 107, 126, 127
**Garneau, Simone**
167
**Gauthier Desrochers, Laurence**
46, 58
**Gauvin, Stephanie**
38
**George, Megan**
110
**George, Reanna**
127
**Gerl, Lorena**
148
**Gesink, Dionne**
78, 151
**Ghabeli, Gloria**
158, 169
**Ghavami, Baharan**
200
**Gibbins, Paige**
110
**Gibson, Jennifer L.**
123
**Gibson-Haerle, Janelle**
124
**Gidda, Ruby**
1
**Gifford, Wendy**
53, 56
**Giguère, Lauriane**
124
**Ginty, Alexandra**
2
**Glass, Karen**
78, 151
**Godin, Émilie**
14
**Gooding, Gregory**
183
**Gosse, Nancy**
156
**Gouin, Jean-Philippe**
95
**Gourgues, Florence**
126
**Graham, Robin**
184
**Greeley, Krista**
90, 127
**Greene, Malka**
162
**Grewal, Ramandip**
34
**Gross, Erin**
76
**Gruben, Karis**
56
**Guarascio, Nikita**
5, 9
**Guilcher, Gregory**
124, 166, 167
**Gupta, Abha A.**
62, 64, 134
**Ha, Michelle**
50
**Hachey, Shauna**
93
**Hadjistavropoulos, Thomas**
102
**Hagopian, Emma**
182, 184, 195
**Hajj, Aline**
83, 84
**Hales, Sarah**
119, 142, 146, 149, 150, 157
**Hannon, Breffni**
142, 146, 149, 150, 157, 160
**Harb, Sami**
189, 190
**Harb, Sarah**
84
**Harlos, Craig**
148
**Harris, Cheryl**
107, 126, 168, 180
**Hashemi, Elham**
102
**Haslam, Julie**
115
**Hasnain, Khulood**
85
**Heathcote, Lauren**
124
**Henry, Brianna**
47, 130
**Henry, Christophe**
29
**Heykoop, Cheryl**
109
**Hilbers, Daniel**
11
**Hill, Leslie**
178
**Hill, Tiffany**
109
**Hollenhorst, Anahita**
178
**Hollenhorst, Helmut**
178
**Holmes, Elizabeth**
61
**Holmes-Rodman, Paula**
16, 36
**Holy, Celeste**
124
**Horne, Fred**
36
**Hou, Sharon**
67
**Hough, Rachael**
135
**Houghton, Emma**
132
**House, Chloe**
79
**Howard, Fuchsia**
67
**Howes, Janice**
178
**Hoy, Sandra**
70
**Hussien, Julia**
29
**Huta, Veronika**
180
**Hwang, Kyobin**
130
**Hyde, Angela**
15
**Ianakieva, Iana**
76
**Ilie, Gabriela**
185, 186, 188, 191, 194
**Ilkow, Carolina**
54
**Isenberg-Grzeda, Elie**
142, 146, 149, 150
**Isherwood, Susan**
190
**Jacquard, Kenneth**
178
**Jansen, Mackenzie**
85, 148
**Janzen, Anna**
67
**Jean, Nabaasa**
21
**Jeji, Tara**
39
**Jennings, Sharon**
15
**Jeyapalan, Apiramy**
61
**Jibb, Lindsay**
102, 104, 130
**Johnson, Andrea**
174
**Jomy, Jane**
181
**Jones, Jennifer**
107, 126, 202
**Kaal, K. Julia**
178
**Kahn, Rochelle**
73
**Kassam, Alisha**
106
**Kauffeldt, Kaitlyn**
129
**Kearns, Emma**
107, 126
**Keats, Melanie**
105
**Keefe, Zoë**
179
**Kelly, Brian**
196
**Kephart, George**
194
**Keys, Elizabeth**
35
**Khalaf, Deema**
140
**Kilabuk, Tapisa**
56
**Kim, Rachel**
78
**Kim, Soyoun Rachel**
147, 151
**Kim, Yoojung**
19, 20
**King, John**
13, 15
**Kittuppanantharajah, Shay**
173
**Klein, Roberta Y.**
184, 195
**Klein, Roberta Yael**
182
**Kokorovic, Andrea**
194
**Kolm, Kari**
63
**Korenblum, Chana**
98, 134, 135
**Kortes-Miller, Katherine**
171
**Krose, Kirsten**
114
**Krull, Kevin**
166
**Kukreti, Vishal**
202
**Kulbak, Olivia**
61
**Kurup, Simone**
108
**Kuruvilla, John**
131
**Körner, Annett**
183
**L, Jennifer**
109
**Ladak, Zeenat**
39
**Lafay-Cousin, Lucie**
166
**Lafontaine, Christine**
54
**Laframboise, Stephane**
78, 147, 151
**Laizner, Andréa Maria**
176, 183
**Lalani, Yasmin**
51
**Lam, Gary**
109
**Lam, Wendy T.**
198
**Lambert, Sylvie**
68
**Lamontagne, Marie-Ève**
14
**Lang, Michael**
170, 171, 177
**Langelier, David**
32
**Langlois, Frédérique**
95
**Larsen, Lorna**
17
**Latimer-Cheung, Amy**
129
**Lau, Emily**
187
**Lau, Sharlane**
73, 78, 147, 151, 202
**Lauzier, Sophie**
83, 84
**Lawal, Oluwaseyi**
132, 166
**Lawrence, Logan**
178
**Lebel, Sophie**
107, 124, 126, 168, 180, 202
**LeBlanc, Gilles**
15
**Lee Dauz, Rachel**
90
**Lee, Rachel**
65
**Lee, Virginia**
192
**Lefebvre, Christine**
37
**Leigh, Natasha**
73
**Lemieux, Julie**
95
**Leslie, Anne**
168, 180
**Lesser, Iris**
67
**Levesque, Ariane**
5
**Lheureux, Stephanie**
110
**Li, Madeline**
78, 123, 142, 146, 147, 149, 150, 151, 157, 159, 160, 182, 184, 187, 195, 198
**Lightfoot, Nancy**
70
**Lingafelt, Ella**
46
**Link, Claire**
128, 152
**Liu, Chunlin**
119
**Lofters, Aisha**
28
**Loiselle, Carmen**
19, 20, 54, 55, 201
**Longpré-Poirier, Christophe**
115
**Lotocka-Reysner, Hanna**
67
**Lovas, Mike**
173
**Low, Jonathan**
105
**Lowry, Elaine**
48
**M. Morin, Charles**
14
**Mabbott, Donald**
67
**MacDonald, Cody**
194
**Macedo, Alyssa**
173
**MacInnis, Cara**
15
**MacIver, Jamie**
109
**Mack, CindyMarie**
148
**Mackenzie, Kelly**
105
**Maheu, Christine**
107, 124, 126, 202
**Mak, Ernie**
160
**Makarious, Mary**
160
**Male, Dana**
38
**Mand, Ashmeet**
31
**Mann, Jasmine**
94
**Manna, Aditya**
88
**Manoj, Megha**
78, 147, 151
**Marcil, Valérie**
5
**Marquis, Marc-Antoine**
9
**Marr, Kristin**
67
**Martin, Tara**
34
**Martinez, Marcelo Fabian**
15
**Martino, Rosemary**
172, 175
**Martopullo, Celestina**
183
**Mason, Ross**
194
**Massicotte, Véronique**
95
**Matheson, Heath**
66
**Matthews, Debora**
93
**Mayo, Samantha**
112, 131
**Mazzocca, Kelly**
102
**Mazzurco, Olivia**
195
**Mc Brearty, Claudia**
95, 121
**McDonough, Meghan H.**
12
**McGonegal, Joshua**
70
**McIntyre, Pauline**
15
**McIntyre, Roger S.**
160
**McLaughlin, Emma**
67, 72, 74, 105
**McMillan, Kimberly**
168, 180
**McNally, Mary**
93
**McNeely, Margaret**
105
**McPartlin, Andrew**
172, 175
**McPherson, Christine**
168, 180
**McTaggart-Cowan, Helen**
77
**Mehnert-Theuerkauf, Anja**
198
**Mehta, Anita**
176
**Memedovich, Emily**
196
**Merali, Meena**
162, 173
**Mestari, Zakaria**
37
**Michaud, Pierre-Luc**
93
**Michel, Alexandra**
4
**Michon, Bruno**
9
**Milburn, Hayley**
67
**Miller, Shannon**
8, 81
**Milroy, Beverly**
170
**Mitchell, Cynthia**
54
**Moodie, Erica**
68
**Moore, Sara**
168, 180
**Moraru, Camelia**
141
**Morrison, Stacey**
131
**Mosher, Pamela**
202
**Nabi, Hermann**
84
**Nairy, Ashwin**
1
**Nanjappa, Aditi**
19
**Nanos, Stephanie**
104
**Nathan, Paul**
102, 130
**Nawara, Roula**
40
**Needham, Judy**
69
**Neil-Sztramko, Sarah**
63
**Nekain, Navid**
11
**Newbold, Christine**
63
**Nissim, Rinat**
111, 119, 123, 142, 146, 149, 150, 157, 168, 180, 195, 202
**Niwe, Salva**
39
**Nordstokke, David**
86
**Norton, Lucas**
76, 89
**Nunez, John-Jose**
11, 18, 137
**O’Connell, Sarah**
8, 81
**O’Driscoll, Louise**
48
**O’Grady, Alice**
109
**Oberoi, Sapna**
85, 130, 148
**Ogez, David**
5
**Olivier-D’Avignon, Marianne**
9
**Olsen, David**
76
**Oprea, Mara**
141
**Osman, Selma**
34
**Ouattara, Malick**
168
**O’Brien, Namiko**
13
**Pace, Keiran**
181
**Pacelli, Maria-Hélèna**
72
**Page, Cheryl**
63
**Palaez, Sandra**
46
**Panjwani, Aliza**
78, 147, 151, 182, 184, 202
**Papadakos, Janet**
173
**Patel, Zeal**
160
**Patil, Nikhilesh**
194
**Patton, Michaela**
82
**Pausche, Beverly**
15
**Peach, Payton**
65
**Peacock, Stuart**
67, 77
**Peddle, Darrell**
13
**Pelaez, Sandra**
58
**Pelletier, Josee**
36
**Pelletier, Wendy**
124
**Peláez, Sandra**
43
**Perry Brinkert, Judi**
158
**Perry, Sarah**
12
**Petricone-Westwood, Danielle**
80
**Philp, Lauren**
110
**Picard, Juliette**
121
**Piccirilli, Amanda**
189
**Piché, Alexia**
105
**Piedalue, Katherine-Ann**
90
**Pitch, Natalie**
64, 134
**Pitman, Anne**
29, 30
**Pizzo, Alex**
102
**Polskaia, Nadia**
29
**Prabhu, Mani**
177
**Pratt, Angus**
39
**Prica, Anca**
131
**Price, Jenson**
33, 129, 203
**Prudent, América**
126
**Prévost, Nadine**
52
**Puts, Martine**
157
**Qualls, Whitney**
77
**Quan, May Lynn**
190
**Quinn, Kaitlyn**
72
**Quinn-Nilas, Chris**
79
**Racine, Shanelle**
131
**Rader, Tamara**
47
**Railton, Cindy**
196
**Raman, Srinivas**
181
**Ramasamy, Vinesha**
39
**Ramphal, Raveena**
67
**Ranger, Tristyn**
47
**Ratnayake, Iresha**
85
**Ravindran, Thisha**
150
**Rayar, Meera**
69
**Rayner, Jennifer**
36
**Refai, Ala**
172, 175
**Rendon, Ricardo**
194
**Rethy, Jodi**
109
**Reynolds, Kathleen**
82
**Ritacca, Patricia**
162
**Rivard, Mélina**
37
**Rivest, Jacynthe**
115
**Robichaud, Lye-Ann**
9
**Robitaille, Julien**
34
**Rockwood, Kenneth**
93
**Rodberg, Danielle**
40
**Rodin, Gary**
73, 104, 110, 118, 123, 159, 160, 162, 182, 184, 187, 195
**Roelfsema, Lisa**
183
**Rondeau, Émélie**
5, 9
**Rooke, Kaylie**
109
**Rosa, William E.**
198
**Rosberger, Zeev**
196
**Rosenblat, Joshua D**
160
**Ross, Lori**
28
**Rosvold, Amy**
36
**Rousseau, Pascale**
52
**Roy, Deanna**
13
**Roy, Heather**
69
**Russell, Brooke**
132
**Rutledge, Robert**
185, 186, 188, 191, 194
**Rydall, Anne**
182, 184, 195
**S. Molnar, Danielle**
95
**Sabir, Soulene**
33
**Sabiston, Catherine**
50
**Sabry, Aliaa**
172
**Sachdeva, Anjali**
62, 134
**Sahay, Tina**
36
**Salins, Naveen**
159, 187
**Salway, Travis**
77
**Santa Mina, Daniel**
105
**Santiago, Lauryn**
192
**Santos, Susy**
169
**Sauro, Khara**
32
**Savard, Josée**
4, 14, 95, 121
**Savas, Sevtap**
13, 15, 55, 69
**Sawer, Sean**
105
**Sayani, Ambreen**
39
**Scali, Antonella**
36
**Scardini, Sara**
48
**Schulte, Fiona**
42, 47, 74, 82, 86, 124, 130, 132, 166, 167, 196
**Schulz-Quach, Christian**
160
**Scime, Samantha**
106
**Scott, Ian**
148
**Seely, Andrew**
29
**Seely, Dugald**
29
**Seevaratnam, Arjuni**
179
**Selby, Debbie**
142, 146, 149, 150
**Shamloo, Maryam**
148
**Shamshad, Sundas**
72
**Shapiro, Gilla**
34, 73, 114, 118, 123, 182, 184, 187, 198
**Sharbafchi, Mohammadreza**
200
**Sharma, Sitara**
6, 7
**Shauman, Madyson**
85, 148
**Shcherbachuk, Viktoriia**
179
**Sibley, Daniel**
105
**Sidhu, Harsimronjoot**
130
**Sierra-Herrera, Camilo**
176
**Singh, Arjunvir**
190
**Sinisi, Daniela**
52
**Sirois-Leclerc, Héloïse**
124
**Site Investigators, RUBY**
190
**Skardasi, Georgia**
15
**Skelton, Mac**
159, 187
**Small, Will**
77
**Smith, Belinda**
178
**Smith, Marlie**
202
**Smith-Morrison, Barb**
156
**Smylie, Michael**
36
**Smyth, Elizabeth**
159, 187
**Spence, Catherine**
63
**Squires, Lauren**
28
**Srivastava, Richa**
73, 118
**St.John, Laura**
74
**Stapleton, Cielle**
85
**Stephenson, Leah**
36
**Stevenson, Benjamin**
76
**Stewart, Archie**
178
**Stokoe, Mehak**
86, 166
**Stonebridge, Genevieve**
109
**Strahlendorf, Caron**
67
**Struik, Laura**
31, 35, 40
**Stukalin, Igor**
196
**Sullivan, Richard**
159, 187
**Sultan, Serge**
5, 9, 102
**Sung, Lillian**
67
**Surajpal, Medha**
131
**T.Martineau, Joé**
115
**Tarasuk, Joy**
178
**Tavakoli, Camelia**
58
**Taylor, Mark**
93
**Taylor, Mischa**
40
**Taylor, Rachel**
135
**Taylor-Cutting, Janine**
13
**Teggart, Kylie**
63
**Theou, Olga**
93
**Thibodeau, Kimberley**
55
**Thurston, Chantale**
47, 130
**Tomasone, Jennifer**
129
**Tomfohr-Madsen, Lianne**
132
**Tong, Eryn**
119, 123
**Torchetti, Tracy**
61
**Trapani, Dario**
159, 187
**Travis, Taylor**
179
**Tremblay, Ashley**
109
**Tremblay, Julie**
37
**Trinh, Linda**
50, 105, 140
**Tromburg, Courtney**
166
**Truong, Tran**
173
**Tsang, Derek**
42
**Turcotte, Simon**
54
**Turner, Rebecca**
36
**Tutelman, Perri**
47, 124, 167, 196
**Tyo-Gomez, Mathias**
9
**Ubeira Amaral, Eugenia**
182
**Udobor, Divine**
8, 81
**Udrea, Iulia**
141
**Unger, Karla**
159, 187
**Uriarte, Reyna**
56
**van Velzen, Roxanne**
109
**Wagoner, Chad**
105
**Wales, April**
128, 152
**Wang, Yuqi**
93, 94
**Wasserman, Sydney**
68
**Watson, John**
63
**Watson, Linda**
128
**Wazni, Liquaa**
56
**West, Sarah L**
8, 81
**Westlake, Brooklyn**
33, 203
**Wexler, Megan**
73, 118
**White, Emily**
65, 66
**Whitty, Judy Donovan**
15
**Wickramasinghe, Bethany**
135
**Widger, Kimberley**
174
**Wilcox, Gabrielle**
86
**Wilke, Derek**
194
**Wodinksi, Lindsay**
170
**Wolfe, Jennifer**
109
**Wong, Evaon**
116
**Wood, Don**
69
**Woodrow, Caitlin**
148
**Woods, Anne**
137
**Wright, Vanessa**
39
**Wulf, Karley**
146
**Wurz, Amanda**
29, 67, 72, 74
**Xiao, Hui**
50
**Xue, Lin**
85
**Yang, Lin**
179
**Yang, Youjin**
40
**Yeates, Keith**
166
**Yee, Amanda**
123
**Yi, Joshua**
181
**Yu, Jennifer**
147
**Yufe, Shira**
190
**Zammit, Miah**
162
**Zarani, Fariba**
200
**Zendel, Benjamin Rich**
66
**Zimmermann, Camilla**
123, 142, 146, 149, 150, 160
**Zwicker, Hailey**
166, 167

